# *Fusobacterium sphaericum* sp. nov., isolated from a human colon tumor adheres to colonic epithelial cells and induces IL-8 secretion

**DOI:** 10.1080/19490976.2024.2442522

**Published:** 2024-12-25

**Authors:** Martha A. Zepeda-Rivera, Yannick Eisele, Alexander Baryiames, Hanrui Wu, Claudia Mengoni, Gianmarco Piccinno, Elsa F. McMahon, Kaitlyn D. LaCourse, Dakota S. Jones, Hans Hauner, Samuel S. Minot, Nicola Segata, Floyd E. Dewhirst, Christopher D. Johnston, Susan Bullman

**Affiliations:** a Vaccine and Infectious Disease Division, Fred Hutchinson Cancer Center, WA, USA; b Genomic Medicine, The University of Texas MD Anderson Cancer Center, Houston, TX, USA; c School of Medicine and Health, Technical University of Munich, Munich, Germany; d Institute of Nutritional Medicine, School of Medicine and Health, Technical University of Munich, Munich, Germany; e Human Biology Division, Fred Hutchinson Cancer Center, Seattle, WA, USA; f Department of Computational, Cellular and Integrative Biology, University of Trento, Trento, Italy; g Data Core, Shared Resources, Fred Hutchinson Cancer Center, Seattle, Washington, USA; h Department of Microbiology, ADA Forsyth Institute, Cambridge, MA, USA; i Department of Oral Medicine, Infection and Immunity, Harvard School of Dental Medicine, Boston, MA, USA; j Immunology, James P. Allison Institute, The University of Texas MD Anderson Cancer Center, Houston, TX, USA

**Keywords:** *Fusobacterium*, novel species, pangenome, comparative genomics, intestinal microbiota, colorectal cancer

## Abstract

Cancerous tissue is a largely unexplored microbial niche that provides a unique environment for the colonization and growth of specific bacterial communities, and with it, the opportunity to identify novel bacterial species. Here, we report distinct features of a novel *Fusobacterium* species, *F.* *sphaericum* sp. nov. (*Fs*), isolated from primary colon adenocarcinoma tissue. We acquire the complete closed genome and associated methylome of this organism and phylogenetically confirm its classification into the *Fusobacterium* genus, with *F. perfoetens* as its closest neighbor. *Fs* is phenotypically and genetically distinct, with morphological analysis revealing its coccoid shape, that while similar to *F. perfoetens* is rare for most *Fusobacterium* members. *Fs* displays a metabolic profile and antibiotic resistance repertoire consistent with other *Fusobacterium* species. *In vitro, Fs* has adherent and immunomodulatory capabilities, as it intimately associates with human colon cancer epithelial cells and promotes IL-8 secretion. An analysis of the prevalence and abundance of *Fs* in > 20,000 human metagenomic samples shows that it is a rarely detected member within human stool with variable relative abundance, found in both healthy controls and patients with colorectal cancer (CRC). Our study sheds light on a novel bacterial species isolated directly from the human CRC tumor niche and given its *in* *vitro* interaction with cancer epithelial cells suggests that its role in human health and disease warrants further investigation.

## Introduction

In patients with colorectal cancer (CRC), unbiased genomic analyses have revealed an enrichment of *Fusobacterium* in CRC tumors relative to noncancerous colorectal tissue.^1–4^ Previous work by our group and others demonstrated that within patient CRC tumors, *Fusobacterium* colonizes distinct regions with immune and epithelial functions supportive of cancer progression,^5^ that it persists with the primary tumor during metastasis,^6^ and that antimicrobial agents targeting these and other anaerobic bacterial species decrease cancer cell proliferation and tumor growth.^6,7^ Furthermore, high *Fusobacterium* loads are associated with high-grade dysplasia,^1^ advanced disease stage,^8^ and poorer patient prognosis.^9^ As tissue-associated microbiomes are not readily amenable to metagenomic analysis, due to the overabundance of host nucleic acids, characterization of tumor-infiltrating microbial communities is often restricted to amplicon-based sequencing approaches with limited phylogenetic resolution. To overcome this challenge, microbiological culture-based approaches with patient tissue specimens have reemerged as valuable tools to isolate members of tissue-associated microbiomes.

Through a culturing-based approach, we isolated, characterized, and sequenced the genome of a novel *Fusobacterium* species from the resected tumor of a treatment naïve female patient with microsatellite stable (MSS), stage III, right-sided colon cancer. Phylogenetic appraisal of this organism places this bacterium within the *Fusobacterium* genus but demonstrates it is distinct from currently known species. Rather than the typical spindle-like, fusiforme morphologies associated with most *Fusobacterium* species, phenotypic analysis of this novel organism revealed a coccobacilloid shape. Acknowledging this distinct spherical cell-shape, we propose this species as *Fusobacterium sphaericum* sp. nov. (*Fs*). In the present study, we find that while the chromosomal structure and gene content of *Fs* are predominantly unique to this species, its metabolic profile and antibiotic resistance repertoire are consistent with other *Fusobacterium* members. Furthermore, our analyses indicate that its predicted virulence factors are related to metabolism, cell–cell adherence, and immunomodulation. *In vitro* co-culture with human colon cancer epithelial cells confirmed *Fs* adherence and indicated that its presence significantly increases IL-8 secretion. Assessment of the prevalence and abundance of *Fs* in 21,212 human metagenomic samples shows that it is a rare member in human stool with variable relative abundance.

## Results

### *Phylogenetic and morphologic characterization of a novel* Fusobacterium *species*

**Figure 1.**
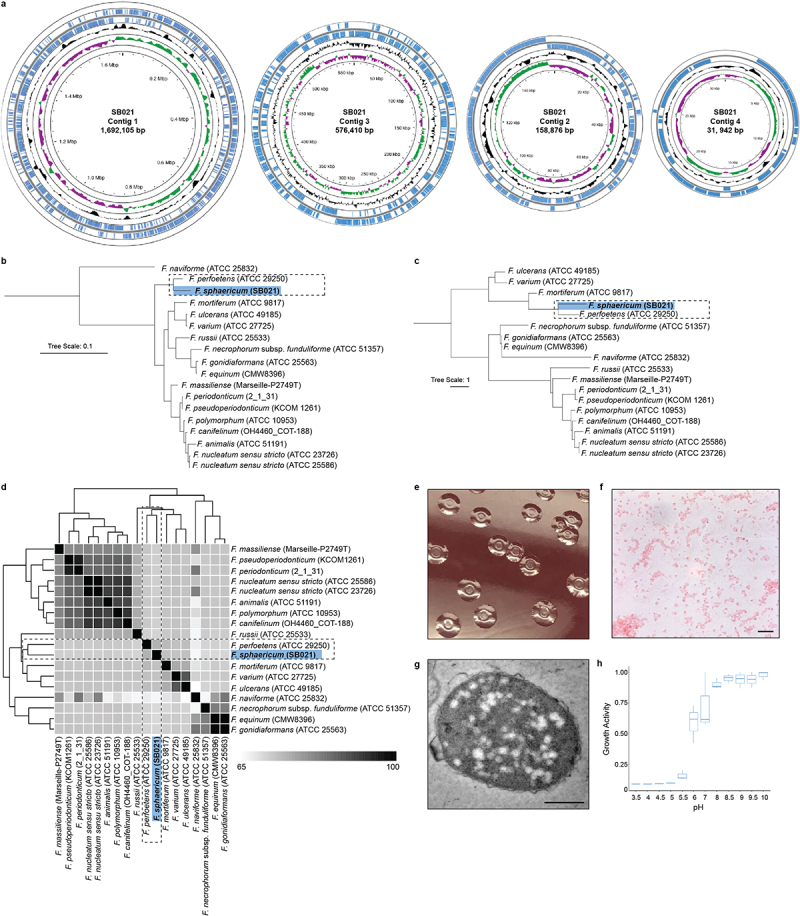
Taxonomic and morphologic analysis of *Fusobacterium sphaericum* sp. nov. (SB021). a) Proksee circular genome architecture of *F. sphaericum* sp. nov. (*Fs*) SB021 contigs. From outwards to inwards, the first and second circles show annotated coding sequences on positive strand and negative strand, respectively. Third circle indicates average GC content, with areas higher than average GC content as outward projections and areas lower than average as inward projections. Fourth circle shows GC skew, with above zero values in green and below zero values in purple. b) A 16S rRNA gene-based dendrogram illustrating the phylogenetic relationship between *Fs* (blue) and other *Fusobacterium* spp. c) kSNP^12^ maximum-likelihood whole-genome phylogenetic tree with *Fs* highlighted in blue. d) Clustered average nucleotide identity (ANI) matrix with *Fs* highlighted in blue. ANI values are reported in Supplementary Table 4. White to black scale indicates an ANI from 65% to 100%, respectively. e) Digital photography of *Fs* SB021 colonies grown on fastidious anaerobe agar enriched with 10% defibrinated horse blood. f) Bright-field microscopy of Gram-stained *Fs* SB021. Images acquired at 100X magnification with oil immersion lens. Image scale bar is 10µm. g) Transmission electron micrographs of an *Fs* SB021 cell. Image scale bar is 500µm. h) Plot indicates growth activity as measured in Biolog PM10 plates for *Fs* SB021. Data is normalized to the maximum value per replicate (*n*=3).

Anaerobic bacterial isolation was performed on a human colorectal cancer (CRC) tumor from a treatment naïve female patient with microsatellite stable (MSS), stage III, right-sided colon cancer. In brief, a piece of fresh tumor tissue (approximately 0.2 cm^3^) was spread across fastidious anaerobic agar and incubated under anaerobic conditions for up to 1 week with growth of bacterial colonies monitored every 24 h (Methods). For bacterial identification, 16S rRNA gene amplification and sequencing was performed on individual bacterial colonies. BLASTn analysis of the 16S rRNA gene sequence of SB021 confirmed its classification into the *Fusobacterium* genus with a top hit against *Fusobacterium perfoetens*, yet all related hits fell below the established 16S rRNA gene species similarity threshold of 98.65%,^10^ suggesting that SB021 could represent a novel *Fusobacterium* species (Supplementary Table S1). Using long-read single-molecule real-time (SMRT)^11^ sequencing, we generated a complete and closed genome and methylome for isolate SB021, identifying two putative chromosomes (1.69 Mb and 0.57 Mb) and two additional extrachromosomal elements (1.59 Kb and 0.32 Kb) (Figure 1a, Supplementary Table S2, Supplementary Table S3). Comparative phylogenetics of SB021 and other *Fusobacterium* spp. genomes (Table 1), both by the 16S rRNA gene and a reference-free whole-genome approach,^12^ showed that SB021 forms a distinct branch within the same clade as *F. perfoetens* (Figure 1b–c). To quantify the relatedness between SB021 and the publicly available *F. perfoetens* ATCC 29250 genome, we calculated the average nucleotide identity (ANI) and found it to be 75.27%, well below the established species threshold of 95–96%,^10^ supporting the classification of SB021 as a novel *Fusobacterium* species (Figure 1d, Supplementary Fig. S1, Supplementary Table S4). GTDB-tk^13^ analysis further supported these observations, as it classified SB021 within the same clade as *F. perfoetens*, as part of “Fusobacterium_B_sp900541465” (Supplementary Table S5). This group is predominantly represented by metagenomically assembled genomes (MAGs) from uncultured samples, with a single uncharacterized isolate from a large-scale cultivation effort of the fecal microbiota in healthy Chinese individuals.^14^

**Table 1.** Genomes used in this study.

Species	Strain	Genome Accession	Genome Length (bp)	GC content	No. of Contigs
*Fusobacterium canifelinum*	OH4460_COT-188	GCA_003859915.1	2224456	27.07	76
*Fusobacterium equinum*	CMW8396	GCA_001546395.1	1797919	32.50	77
*Fusobacterium gonidiaformans*	ATCC 25563	GCA_000158835.2	1698329	32.44	6
*Fusobacterium massiliense*	Marseille_P2749T	GCA_900095705.1	1809170	27.33	6
*Fusobacterium mortiferum*	ATCC 9817	GCA_000158195.2	2671036	29.07	8
*Fusobacterium naviforme*	ATCC 25832	GCF_003014445.1	2400574	52.82	23
*Fusobacterium necrophorum* subspecies *funduliforme*	ATCC 51357	GCA_000262225.1	2110802	34.92	45
*Fusobacterium nucleatum* subspecies *animalis* (suggested name *Fusobacterium animalis*)	ATCC 51191	CP096640	2562868	27.04	1
*Fusobacterium nucleatum* subspecies *nucleatum* (suggested name *Fusobacterium nucleatum sensu stricto*)	ATCC 25586	GCA_003019295.1	2180116	27.14	1
*Fusobacterium nucleatum* subspecies *nucleatum* (suggested name *Fusobacterium nucleatum sensu stricto*)	ATCC 23726	GCA_003019785.1	2299645	27.18	1
*Fusobacterium nucleatum* subspecies *polymorphum* (suggested name *Fusobacterium polymorphum*)	ATCC 10953	GCA_037900625.1	2474009	27.02	3
*Fusobacterium perfoetens*	ATCC 29250	GCA_000622245.1	2103008	25.97	25
*Fusobacterium periodonticum*	2_1_31	GCA_000158215.3	2546312	28.05	6
*Fusobacterium pseudoperiodonticum*	KCOM 1261	GCA_002763625.1	2372880	28.04	1
*Fusobacterium russii*	ATCC 25533	GCA_000381725.1	1941804	28.64	41
*Fusobacterium sphaericum* sp. nov.	SB021	CP055997-CP056000	2459333	30.06	4
*Fusobacterium ulcerans*	ATCC 49185	GCA_000158315.2	3487504	30.38	8
*Fusobacterium varium*	ATCC 27725	GCA_000159915.2	3299801	29.18	11

Table indicates each *Fusobacterium* genome used for comparative analyses in this study with their corresponding genome accession numbers, genome length, GC content, and number of contigs in the assembly noted.

The cellular morphology and pH tolerance of *Fusobacterium* cells is known to vary.^15–17^ Macroscopically, SB021 cells form distinct, vaguely round, raised opaque colonies with central indentations following 48 h of anaerobic culture. The colonies are viscous and malodorous and measure 3–5 mm in diameter (Figure 1e). Microscopically, SB021 cells are short, gram-negative coccobacilli, with an average length of 1.60 μm and an average width of 1.25 μm (Figure 1f), displaying a spherical shape via transmission electron microscopy (Figure 1g), scanning transmission electron microscopy (Supplementary Fig. S2), and confocal laser scanning microscopy (Supplementary Video 1 https://doi.org/10.6084/m9.figshare.27698502.v1). Although most *Fusobacterium* species members tend to form slender filamentous rods with pointed ends, *F. perfoetens* has previously been described as short, ovoid coccobacillus. Our results suggest that members within the *F.* *perfoetens/F.* *sphaericum* sp. nov. clade (Figure 1b–c) share similar cellular morphology. Assessment of preferential growth pH indicated that SB021 is sensitive to pH below 5 and demonstrates a growth preference between pH 8 and pH 10 (Figure 1h). Given the coccobacilloid morphology of SB021 cells, we propose this novel species, part of “Fusobacterium_B_sp900541465”, as *Fusobacterium sphaericum* sp. nov. (*Fs*).

### Fs *genomic attributes predict metabolic and virulence capabilities*

**Figure 2.**
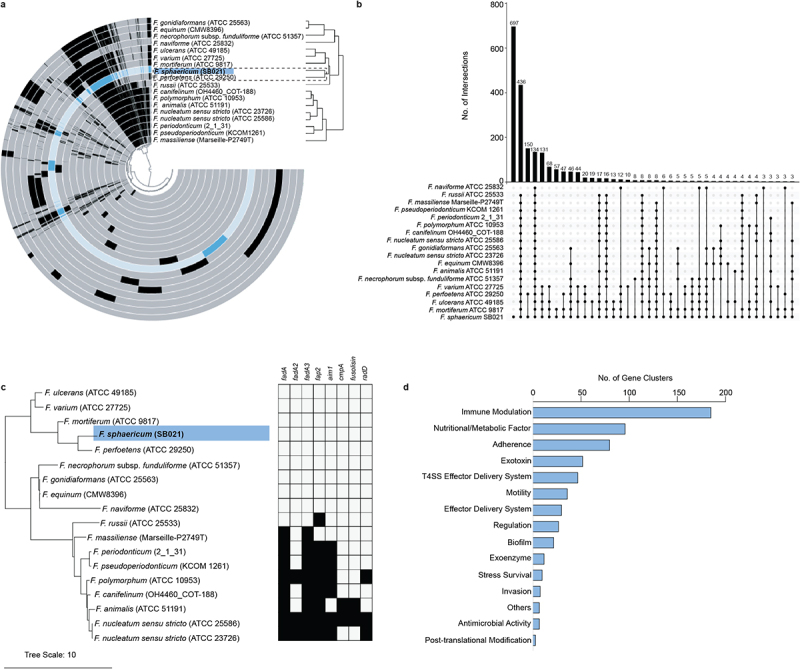
*Fusobacterium sphaericum* sp. nov. (SB021) gene content analysis. a) Anvi’o pangenomic analysis^19^ of *F. sphaericum* sp. nov. (*Fs*) SB021 (blue) as compared to 17 other *Fusobacterium* spp. genomes (Table 1). Each circle represents an individual genome, with identified gene clusters in blue (SB021) or black (other *Fusobacterium* spp.). Genomes are ordered by ANI clustering (Figure 1d). b) Upset plot depicting the presence of gene clusters identified in *Fs* across other *Fusobacterium* spp. genomes. Bar height (top) indicates number of gene clusters shared between combinations of genomes (bottom). c) Presence (black) versus absence (white) plot of canonical virulence factors across *Fusobacterium* spp. genomes, ordered by a kSNP^12^ maximum-likelihood whole-genome phylogenetic tree with *Fs* highlighted in blue. d) *Fs* anvi’o-identified gene clusters were analyzed using the virulence factor database (VFDB).^33^ Graph shows the number of gene clusters in each of the top functional categories of putative virulence factors (Supplementary Table 5).

As significant genomic heterogeneity has been reported within the *Fusobacterium* genus, with genome size, architecture, and content differing between species of varying pathogenic capabilities,^17,18^ we sought to characterize the genomic content of *Fs*. To compare the genetic content of *Fs* to other *Fusobacterium* spp., we implemented the analysis and visualization platform for ‘omics data (Anvi’o) workflow for microbial pangenomics.^19^ This genus-level pangenomic analysis allows us to identify all genes present in the *Fusobacterium* genus (“pangenome”) and discern between gene content that is shared among ≥95% of species (“core genome”), is shared among subsets of species (“accessory genomes”) or is unique to individual members (“singletons”). Across 18 representative *Fusobacterium* genomes (Table 1), only 4.9% of gene clusters compose the core (576/11,710), indicative of the extreme genetic heterogeneity across this genus (Figure 2a). *Fs* harbored 18.7% of identified gene clusters (2,192/11,710), but of these, 31.8% (697/2,192) were unique to *Fs*, while only 6.8% (150/2,192) were shared with its closest phylogenetic neighbor, *F. perfoetens* (Figure 2b).

As the genetic content of *Fs* is predominantly unique to this species, we assessed the 2,192 identified gene clusters. KEGG ortholog analysis^20^ revealed that the mapped gene clusters (50.8%) were predominantly involved in metabolic functions and pathways (Supplementary Table S6). Primary metabolic pathways assessment via gutSMASH^21^ indicated shared predicted metabolic capabilities between *Fs* and other *Fusobacterium* genomes (Supplementary Fig. S3). *In vitro* metabolic profiling via the API 20A Gallery System showed that *Fs* ferments the monosaccharides glucose and mannose, the disaccharide lactose, the trisaccharide raffinose, and the coumarin glucoside esculin (Supplementary Fig. S4a). Glucose, mannose, and raffinose metabolism were also observed via the Biolog Anaerobe Identification Test Panel (AN Plate) (Supplementary Fig. S5). Enzymatic profiling via the API ZYM panel additionally showed positive activity for alkaline phosphatase, C4-esterase, C8-esterase-lipase (weak), acid phosphatase (weak), naphthol-AS-BI-phosphohydrolase (weak), and α-Galactosidase (strong) (Supplementary Fig. S4b). This strong α-Galactosidase activity may be a distinct feature of *Fs*, as activity for this enzyme has only been observed weakly in *F. mortiferum* and *F. necrogenes* .^22^

Notably, *Fs* harbors 19 open reading frames encoding putative multidrug resistance proteins (Supplementary Table S1) and further analysis with the comprehensive antibiotic resistance database (CARD)^23^ identified 183 putative antibiotic resistance genes (Supplementary Table S7), suggesting that *Fs* is resistant to a suite of antibiotics. Our group has previously demonstrated that antibiotic-mediated microbiota modulation, targeting *Fusobacterium* and other anaerobes, decreases cancer cell proliferation and tumor growth.^6^ As such, we sought to characterize the antibiotic response of *Fs. In vitro* antibiotic susceptibility testing demonstrated that *Fs* is susceptible to various classes of antibiotics, including metronidazole (MIC 0.502 μg/ml; nitroimidazole class), colistin (MIC 0.875 μg/ml; polymyxin class), and penicillin (MIC 0.079 μg/ml; beta-lactam class). Consistent with other *Fusobacterium* spp., *Fs* is resistant to erythromycin (macrolide class) and vancomycin (glycopeptide class) antibiotics. Curiously, *Fs* susceptibility to aminoglycosides varied, with susceptibility to gentamycin (MIC 62.2 μg/ml) and resistance to streptomycin and kanamycin (Table 2).

**Table 2.** *F.* *sphaericum* sp. nov. SB021 antibiotic resistance profile.

Antibiotic Agent	MIC in mg/ml	Susceptible (S)/Resistant (R)
Colistin	0.875	S
Erythromycin	-	R
Gentamycin	62.2	R
Kanamycin	-	R
Metronidazole	0.502	S
Penicillin G	0.079	S
Streptomycin	-	R
Vancomyin	-	R

Table shows results for *F.* *sphaericum* sp. nov. susceptibility or resistance to nine tested antibiotic compounds.

Given that *Fs* was isolated directly from a patient's CRC tumor, we next queried the presence of virulence factors important for host-bacterial interactions. We found that *Fs* lacks known *Fusobacterium* virulence factors^24–32^ (Figure 2c) but of the 2,192 identified gene clusters, 28.4% of them are homologous to known virulence factors in other bacterial pathogens^33^ (Supplementary Table 6). Of this subset, the majority function to modulate the immune system (29.7%), to scavenge and metabolize nutritional sources (15.4%), or to adhere to host cells (12.9%) (Figure 2d). This suggests that *Fs* has genetic attributes consistent with facilitating eukaryotic cell interactions and modulation of immune responses.

### Fs *adheres to colon cancer epithelial cells and increases IL-8 secretion*

**Figure 3.**
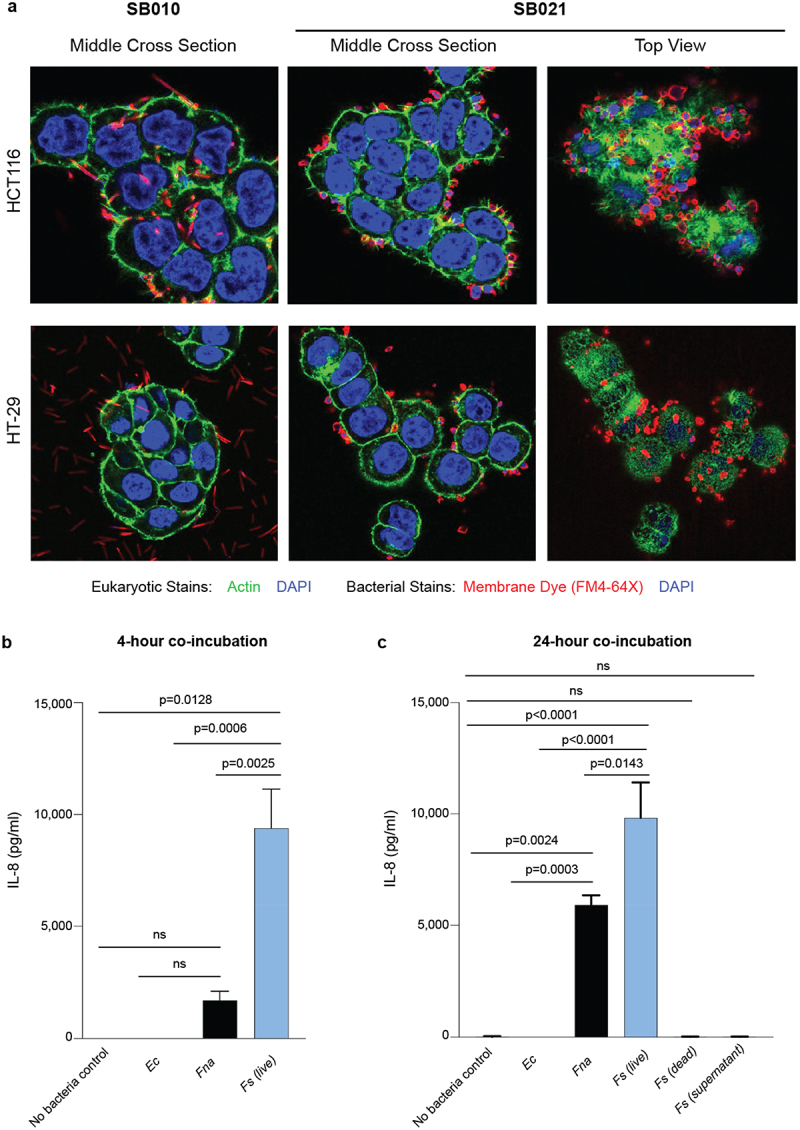
*Fusobacterium sphaericum* sp. nov. adheres to colonic epithelial cells and stimulates IL-8 secretion. Confocal laser scanning microscopy of colon cancer epithelial cell lines, HCT116 and HT-29, co-incubated with *Fusobacterium nucleatum* subsp. *animalis* (*Fna*) SB010 or *F.* *sphaericum* sp. nov. (*Fs*) SB021. Bacterial cell staining: FM4-64X (red) and DAPI (blue). Eukaryotic cell staining: DAPI (blue), actin (green). b–c) Quantitative levels of IL-8 secretion measured via the Single-Analyte ELISArray from HCT116 cells incubated alone or co-incubated with *Escherichia coli* TOP10, SB010, or SB021 at b) 4-hour and c) 24-hour time points. For c) additional co-incubations with dead SB021 and cell-free supernatant were performed. Statistical analysis performed via a one-way ANOVA. For a–c, all co-incubations were at a multiplicity of infection (MOI) of 100.

To evaluate the interaction between *Fs* and colon epithelial cells, we co-cultured *Fs* SB021 with cell lines derived from human colorectal adenocarcinomas, HCT116 and HT-29. As *Fusobacterium nucleatum* subsp. *animalis* (*Fna*) has previously been reported to adhere to and invade cancer epithelial cells,^6^ we used *Fna* SB010 as a positive control. Confocal laser scanning microscopy of fluorescent immunocytochemically stained cells showed a spherical shape for SB021, consistent with our previous observations (Figure 1f–g, Supplementary Fig. S2, Supplementary Video S1) and furthermore demonstrated that SB021 strongly adheres to epithelial cell surfaces in chain-like clusters with both HCT116 and HT-29 (Figure 3a). However, we did not observe intracellular SB021, which suggests that, unlike SB010, SB021 adheres to but does not invade colonic epithelial cells. *In vitro*, *Fs* SB021 did not significantly alter the phosphorylation of markers indicative of G1/S and G2/M cell cycle phases as compared to no bacteria control (Supplementary Fig. S6).

Given that the dominant function assigned to the predicted *Fs* virulence factors is immune modulation (Figure 2d, Supplementary Table 6), we next investigated whether the physical interaction between *Fs* and colonic epithelial cells may affect eukaryotic immune responses by assessing chemokine and cytokine induction in HCT116 cells post co-culturing. In the presence of SB021 cells, we observed an increase in various pro-inflammatory chemokines in two independent Multi-Analyte ELISArray panels, most notably IL-8 (Supplementary Fig. 7). IL-8 expression is elevated in colonic tissue of patients with CRC,^34^ enhances colon cancer cell growth and migration to the liver and lungs,^35,36^ and has previously been reported to be stimulated by the presence of other *Fusobacterium* species including *Fna* and *F.* *nucleatum* subsp. *nucleatum* (*Fnn*).^37^ Quantitative comparison of IL-8 production in the presence of SB021 or SB010 indicated that co-incubation with SB021 stimulated higher IL-8 levels at both 4-h (SB021: average 13,403 pg/ml; SB010: average 1,732 pg/ml) and 24-h (SB021: average 12,004 pg/ml; SB010: average 5,551 pg/ml) time points (Figure 3c).

### Fs *is a low prevalence member in human stool*

**Figure 4.**
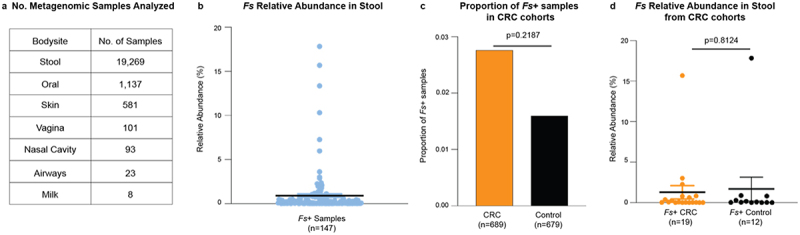
*Fusobacterium sphaericum* sp. nov. is present in human stool. The relative abundance of *F.* *sphaericum* sp. nov. (*Fs*) SB021 was assessed in a) 21,212 metagenomic samples from a various human body sites. b) Graphs show *Fs* percent relative abundance in samples detected. Black line indicates mean. c–d) In datasets comparing patients with CRC to healthy controls (Supplementary Tables 8–9) c) graph shows proportion of samples in which *Fs* was detected. Statistical analysis performed via two-sample Z test. d) Graph shows *Fs* percent relative abundance in samples detected for patients with CRC or healthy controls. Black line indicates mean. Statistical analysis performed via Welch’s T-test.

Given that *Fs* is a novel *Fusobacterium* species with adherent and immunomodulatory capabilities *in* *vitro*, we next sought to determine its prevalence in human specimens from various body sites. The presence of *Fs* SB021 and its nearest phylogenetic neighbor, *F. perfoetens* ATCC 29250, was assessed across 21,212 publicly available human metagenomic samples from the human oral cavity, skin, vagina, airways, breastmilk, nasal passage, and stool (Figure 4a, Supplementary Table 8). Results indicated that SB021 could be detected in stool samples at a low prevalence (147/21,382 = 0.687%), with a range of relative abundances from 0.0005% to 17.829% when detected (Figure 4b). Of the 147 samples in which SB021 could be detected, 31 originated from cohorts comparing patients with CRC to healthy controls (Supplementary Tables 8–9). Nevertheless, no significant difference in SB021 prevalence (*p* = 0.1936, two samples Z-test) (Figure 4c) or abundance (*p* = 0.8124, Welch’s t-test) (Figure 4d) was observed based on disease status in those cohorts. In contrast, ATCC 29250 was detected in only a single stool sample with a low relative abundance of 0.00468% (Supplementary Table 8). Collectively, these findings shed light on a novel bacterial species isolated from the CRC tumor niche, which is also detectable within the human intestinal microbiota.

## Discussion

Due to the abundance of host-associated nucleic acids in complex specimens, tissue-associated microbiome analysis is currently restricted to amplicon-based sequencing. As such, applying traditional culturing-based approaches to tumor specimens provides a valuable opportunity to expand our understanding and classification of tumor-infiltrating microbes.^38^ Here, we report the isolation and characterization of a novel *Fusobacterium* species, *F.* *sphaericum* sp. nov. (*Fs*), from a primary colon adenocarcinoma of a female patient. Phylogenetic and pangenomic analyses reveal that *Fs* is most closely related to *F. perfoetens* (ATCC 29250), but support that *Fs* is a distinct species with an unusual genome architecture, differing from related *Fusobacterium* spp. such as *F. nucleatum sensu lato*, and predominantly unique gene content (Figures 1, 2).

Motivated by the predicted cell–cell adherent and immunomodulatory capabilities of *Fs* genetic content (Figure 2d), we investigated the interactions between *Fs* and colonic epithelial cells in vitro. Transmission electron microscopy and confocal microscopy of fluorescent immunocytochemically stained cells from *Fs* co-culture with human colon epithelial cell lines demonstrated that *Fs* adheres to eukaryotic epithelial cells but does not appear to have invasive capabilities, in contrast to other *Fusobacterium* species, such as *F. nucleatum* subsp. *animalis* (*Fna*) (Figure 3a). *Fs* cells furthermore induced eukaryotic IL-8 secretion from human colon epithelial cells (Figure 3b–c). Quantitative assessment of IL-8 levels indicated that IL-8 secretion from epithelial cells was higher in the presence of *Fs* as compared to *Fna* (Figure 3c). Recent studies have demonstrated increased secretion of IL-8 in colorectal cancer (CRC) tissue and liver metastases^34,35^ with a significant upregulation in the presence of other *Fusobacterium* species including *Fna* and *F. nucleatum* subsp. *nucleatum (Fnn)* .^37^ However, in *Fnn*, induction of IL-8 secretion is dependent on Fap2-mediated adhesion to host cells.^37^ As *Fs* lacks Fap2 (Figure 2c) and was not observed to invade colonic epithelial cells (Figure 3a), further studies to identify the *Fs* factors that induce IL-8 secretion are warranted. Our results demonstrate that *Fs* induces IL-8 secretion and adds a novel species to a growing body of evidence for a distinct role of microbes contributing to a pro-inflammatory environment within human tissues, including tumors.

We demonstrate that in human metagenomic samples (*n* = 21,212) *Fs* is also a member of the human stool microbiota in both healthy individuals and patients with CRC (Figure 4). Although no significant difference in prevalence or abundance based on health status was observed, this could be due to the small number of *Fs*^+^ samples (*n* = 31). Notably, *Fs* was not detected in any oral samples. Previous studies have demonstrated that between *Fusobacterium* species in the oral cavity, *F. nucleatum* can predominate, while other species are present at appreciably lower abundance and prevalence.^39^ However, as *Fs* also lacks the Aim1^28^ and RadD^29^ adhesins, which in *F. nucleatum* mediate interactions with other bacterial species in the oral cavity, it is possible that *Fs* is not a stable member of the human oral microbiome.

The closest phylogenetic neighbor of *Fs* SB021, *F. perfoetens* ATCC 29250, was only detected in a single human stool sample. It is noteworthy that the first described isolate of *F. perfoetens*, acquired in 1900 from the diarrhea of infants, was unfortunately lost to history. As a result, subsequent isolates from a horse cecum (strain CC1) and piglet feces (strain ATCC 29250) were classified as the same species as the original 1900 isolate, but based only on cellular morphology and metabolism, as sequencing technologies for phylogenetic classifications were unavailable at the time.^16^ Although this species underwent various name changes, ATCC 29250 was designated as the type strain for species *F. perfoetens* and remains the only available isolate in existence.^16^ The very low detection of *F. perfoetens* ATCC 29250 across human metagenomic samples is likely reflective of its swine origin and perhaps suggests that the original 1900 human stool isolate was a phylogenetically distinct microbe despite morphological similarities. With its similar coccobacilloid morphology, it is possible that *Fs* SB021, isolated from a human colonic tumor, is a phylogenetic representative of the original 1900 human isolate, distinct from *F. perfoetens* ATCC 29250.

Future genomic studies including *Fs* will be required to reveal its relationship with other microbial members and decipher its impact on the human host. Whether the presence of *Fs* in colorectal patients may contribute to dysbiosis, *in vivo* cancer progression, dysbiosis, or patient prognosis in the context of CRC requires further investigation in animal models and within longitudinal studies of large human cohorts. Testing whether any of the currently established murine models for *F. nucleatum sensu lato* will translate for assessing *Fs*-associated effects *in* *vivo* was not assessed in this study but is of interest for future endeavors.

In conclusion, in this study we have emphasized and demonstrated the importance of reductionist approaches, such as traditional microbial culturing, to isolate and identify members of tissue-associated microbiota which are not yet amenable to metagenomics. Next-generation sequencing techniques, such as long-read SMRT-Sequencing,^11^ allows for the assembly of high-quality, closed, and complete genomes from which accurate phylogenetic characterization, putative functions, and virulent capabilities can be predicted through comparative genomic analyses. Deciphering the role of identified novel microbial species within tumor-associated bacterial communities on disease outcomes remains crucial for informing future preventative and therapeutic interventions.

## Methods

### Bacterial isolation and culturing

Written informed consent was obtained from patient as approved by the Fred Hutchinson Cancer Center Institutional Review Board. Primary adenocarcinoma tumor tissue was collected by hospital staff. Tissue was stored in sterile, chilled RPMI growth medium (Corning, Corning NY, USA) on ice. A small cross section of the tumor was cut from the main tissue mass, lightly scored, and then spread onto a small section on fastidious anaerobe agar (FAA) plates (Oxoid, ThermoFisher Scientific, Waltham, MA, USA) supplemented with 10% defibrinated horse blood (DHB; Lampire Biological Laboratories, Fisher Scientific, Pipersville, PA, USA). Using a sterile loop, the section was spread on the plates for the isolation of single colonies and subsequently, the plates were incubated at 37°C under anaerobic conditions. Every 48 h the plates were checked for new growth, and the identity of colonies was determined by colony PCR as described below. For all subsequent experiments, *F.* *sphaericum* sp. nov SB021 was grown for 48 h on FAA + 10% DHB plates supplemented with josamycin (3 μg/ml), vancomycin (4 μg/ml), and norfloxacin (1 μg/ml) (Sigma Aldrich, USA) and incubated at 37°C under anaerobic conditions (AnaeroGen Gas Generating Systems, Oxoid, ThermoFisher Scientific, USA).

### 16S rRNA bacterial colony PCR

Single colonies were collected with a 1 μl loop and the loop was then suspended in 20 μl of nuclease free water (Promega, Madison WI, USA). The same 1 μl loop was then used to streak a FAA + 10% DHB to subculture and isolate pure colonies. The suspensions were placed in a thermocycler at 98°C for 20 min and then cooled to 4°C. Two microliters of the lysate was subsequently used in a 20 μL PCR reaction (Promega, Madison WI, USA) with a forward primer 342F (5’-CTA CGG GGG GCA GCA G-3’) and reverse primer 1492 R (5’-TAC GGY TAC CTT GTT ACG ACT T-3’) at a final concentration of 1 μM. PCR products were shipped for Sanger Sequencing, and sequencing results were analyzed using NCBI’s BLASTn for species level identification. Identified strains were stocked in 60% TSB with 40% glycerol (v/v) at −80°C.

### High-molecular weight genomic DNA (gDNA) extraction

High molecular weight genomic DNA was extracted using the MasterPure^TM^ Gram Positive DNA Purification Kit (Epicentre, Lucigen, USA). Cells from two plates were re-suspended in 1.5 mL 1X PBS and harvested by centrifugation. Pellets were subjected to manufacturer instructions modified by doubling all reagent volumes and removing vortexing steps to prevent DNA shearing. HMW gDNA was quantified using a Qubit fluorometer (ThermoFisher Scientific, USA).

### PacBio single-molecule real-time sequencing and genome assembly

Single molecule real-time sequencing (SMRT-Seq)^11^ was carried out on a PacBio Sequel instrument (Pacific Biosciences, USA) or a PacBio Sequel II instrument (Pacific Biosciences, USA) at the University of Minnesota Genomics Center. Sequencing reads were processed using Microbial Assembly pipeline within Pacific Biosciences’ SMRTAnalysis pipeline version 9.0.0.92188. Additional assembly was performed using Flye assembler version 2.8 (https://github.com/fenderglass/Flye). Functional predictions for genes were conducted with the Rapid Annotations using Subsystems Technology (RAST)^40,41^ tool (Supplementary Table S1).

### Phylogenetic classification

The full-length 16S rRNA gene sequence for *F.* *sphaericum* sp. nov. SB021 was extracted from its whole-genome sequence and analyzed using NCBI BLASTn (Supplementary Table 3). For available *Fusobacterium* type strains, the 16S rRNA gene sequence was downloaded, and all resulting sequences were aligned via MEGA X^42^ using the MUSCLE clustering algorithm from which a maximum-likelihood dendrogram was generated. kSNP3^12^ with a kmer size of 17, resulting in a fraction of core kmers (FCK) of 0.113, was used to generate a maximum-likelihood whole-genome, reference-free phylogeny of these strains. Final tree were generated using the interactive Tree of Life (iTOL) tool, version 5.^43^ Average nucleotide identity was calculated using the EZ BioCloud ANI calculator.^44^ Further phylogenetic classification was determined using GTDB-tk^13^ (https://github.com/Ecogenomics/GTDBTk.). For *Fusobacterium nucleatum*, ANI thresholds have led to the adoption of different naming schemes based on the recognition of the historical four subspecies as distinct species. In this work, we have implemented the *F. nucleatum sensu lato* nomenclature, referring to each subgroup by their historical subspecies name.

### Pangenomic analysis

Pangenome analysis was performed using the Analysis and Visualization platform for microbial ‘omics (Anvi’o) workflow^19^ with standard thresholds set to a minbit of 0.5 and an MCL of 2. All *F.* *sphaericum* sp. nov. gene clusters were analyzed via KofamKOALA^20^ for KEGG ortholog mapping for their putative functions and analyzed against the Virulence Factor Database (VFDB)^33^ to identify putative virulence-associated genes.

### Bacterial gram staining

Gram staining was performed on *F.* *sphaericum* sp. nov. SB021 uniform bacterial smears after growth at 37°C for 48 h followed by toxic oxygen exposure using a Gram Stain Kit (Remel, Lenexa, KS, USA). The stains were imaged with TissueFAXS microscope system (TissueGnostics, Vienna, Austria) with 100× oil immersion at Fred Hutchinson Cancer Center, Seattle, WA, USA.

### Biolog PM10 phenotype and anaerobe identification test panel microarray plates

*F.* *sphaericum* sp. nov. SB021 and *F. nucleatum* subsp. *animalis* SB010 were grown on FAA plates (Oxoid, Thermo Fisher Scientific) supplemented with 10% DHB (Fisher Scientific) incubated at 37°C in a Concept1000 anaerobic chamber (BakerRuskinn) for 24 h. PM10 and AN plates with corresponding inoculating fluids (IF-0a/IF-10b, and AN-IF, correspondingly) were pre-reduced, either at 4°C overnight (PM10) (AnaeroGen Gas Generating Systems, Oxoid, Thermo Fisher Scientific) or by manufacturer (AN). PM10 and AN plates were brought to room temperature under anaerobic conditions (AnaeroGen Gas Generating Systems, Oxoid, Thermo Fisher Scientific). Bacterial suspensions were made and added to each plate under anaerobic conditions at 37°C (BakerRuskinn). For PM10 plates, bacterial cells were resuspended in 2 ml of pre-reduced IF-0a and normalized across all samples to an optical density at 600 nm (OD_600 nm_) of 0.179 as recommended by Biolog. The final suspension was prepared by combining 0.75 ml of normalized bacterial suspension with 11.25 ml of mix B (100 ml pre-reduced IF-10b with 1.2 ml dye mix D, and 11.18 ml pre-reduced sterile water) to a final volume of 12 ml. For AN plates, bacterial cells were resuspended in 3 ml of pre-reduced AN-IF and normalized across all samples to an OD_600 nm_ of 0.1 as recommended by Biolog. For both PM10 and AN plates, 100 μl of their respective final suspension was added to each well. All plates were then equilibrated to aerobic conditions at room temperature for 10 min and then incubated under anaerobic, hydrogen-free conditions for 24 h at 37°C (AnaeroGen Gas Generating Systems, Oxoid, Thermo Fisher Scientific). All plates were imaged, and absorbance at 590 nm was quantified using a plate reader (Biotek).

### API 20A and API zym metabolic profiling

The metabolic profile of *F.* *sphaericum* sp. nov. SB021 was determined with the API 20A Gallery System (bioMérieux, Marcy-l’Étoile, France) by utilization of colonies after 24 h anaerobic culture. The colonies were resuspended in phosphate-buffered saline (PBS, Corning, Corning, NY, USA) to obtain an optical density at 600 nm (OD_600 nm_) of 0.4–0.5 ( = 3 McFarland) for inoculation of pre-coated capsules on the incubation strip. Results were read after 24 h and additional procedures were carried out as indicated by the manufacturer. The enzymatic profile was investigated similarly with the API Zym Gallery System (bioMérieux, Marcy-l’Étoile, France), except for an OD_600 nm_ of 0.7 ( = 5–6 McFarland) and an incubation period of 4–4.5 h. Both API 20A and API Zym were performed with three replicates.

### Antibiotic sensitivity testing

*In vitro* testing of antibiotic susceptibility of *F.* *sphaericum* sp. nov. SB021 was performed on harvested bacteria after 24-h incubation at 37°C under anaerobic conditions. Cells were resuspended in PBS to an OD_600 nm_ of 0.5 and used to seed bacterial lawns on fastidious anaerobe agar (FAA) plates (Oxoid, ThermoFisher Scientific, Waltham, MA, USA) supplemented with 10% defibrinated horse blood (DHB; Lampire Biological Laboratories, Fisher Scientific, Pipersville, PA, USA). Antibiotic reading strips (MIC test strips; Liofilchem Diagnostics, Waltham, MA, USA) were placed on the lawns with sterile tweezers. All experiments were performed under anaerobic conditions and in triplicates for each antibiotic agent including negative controls (no bacteria). Results were read after 24 h of incubation.

### Mammalian Cell Culture

Mammalian cell culture was performed on the cell lines HCT116 (CCL-247; ATCC, Manassas, VA, USA) and HT-29 (HTB-38; ATCC, Manassas, VA, USA). Mammalian cells were grown in T75 culture flasks using McCoy’s 5A culture medium (Iwakata and Grace Modification) (Corning, Corning NY, USA) with 10% fetal bovine serum (FBS; Sigma-Aldrich, St. Louis, MO, USA), and antibiotics (1× Pen/Strep: penicillin (5000 units/ml), streptomycin (5000 µg/ml), Gibco, Carlsbad, CA, USA) and incubated constantly in a 37°C/5% CO_2_ air humidified incubator. Cells were checked microscopically for monitoring purposes (growth rate, confluency) and split upon reaching confluency (≥80%). Trypsin-EDTA (Gibco, Carlsbad, CA, USA) was added to the flask to dissociate cells from the flask surface, followed by neutralization of the dissociation agent with the growth medium. Subsequently, the cell suspension was centrifuged for 3 min at 302 RCF at room temperature. The cell pellet was resuspended in a sterile medium and seeded into new flasks. Passage numbers were recorded and did not exceed *n* = 10. Prior to co-culture experiments, cells were split as described above and resuspended in an antibiotic-free sterile growth medium, added to 6-well plates to a final concentration of 1.5 × 10^6^ cells per well, and incubated for 24 h before experimental testing.

### Scanning transmission electron microscopy (S/TEM)

*F.* *sphaericum* sp. nov. SB021 was cultured as described above. Cell size of *F.* *sphaericum* sp. nov. SB021 was determined via scanning transmission electron microscopy (S/TEM). We co-incubated HCT116 human colorectal carcinoma cell line (CCL-247; ATCC, Manassas, VA, USA) at low passage (<10) with *F.* *sphaericum* sp. nov. SB021 (OD_600 nm_ = 1.0) for 4 h followed by fixation with ½ strength Karnovsky’s fixative (2.5% glutaraldehyde and 2% paraformaldehyde in 0.1 M cacodylate buffer) and further sample preparation on the same day. Specimens were viewed on a JEOL JEM 1400 transmission electron microscope (JEOL, Tokyo, Japan) with an accelerating voltage of 120kV. Images were acquired with a Gatan Rio 4k × 4k CMOS Camera (Gatan, AMETEK, Berwyn, PA, USA) at Fred Hutchinson Cancer Center, Seattle, WA, USA.

### *Confocal microscopy of* Fusobacterium *co-cultures with human cell lines*

*Fusobacterium* strains, *F.* *sphaericum* sp. nov. SB021 and *F. nucleatum* subsp. *animalis* SB010, were grown for 48 h at 37°C in anaerobic conditions and resuspended in 1 mL of PBS at a final concentration of 5 × 10^8^ cells/mL. Bacterial membranes were stained in this suspension with 50 µl of 100 mg/mL FM 4-64FX (Molecular Probes) and washed twice in PBS by centrifugation for 7,000 × g at RT. Cells from human adenocarcinoma tumor cell lines, HCT116 and HT-29, were grown and passaged as described above and resuspended in 1 mL of McCoys 5A + 10% FBS. 6 × 10^4^ HCT116 or HT-29 cells were seeded with an MOI of 100 bacterial cells in 6-well plates. Bacteria were centrifuged onto HCT116 and HT-29 at 300 × g for 5 min at room temperature (RT), and plates were immediately placed in a 37°C CO_2_ incubator and cultured together for 4 h. Cell lines were then washed 3× with PBS (all washes are 5 min in duration at RT) and then fixed in 4% paraformaldehyde in PBS for 30 min at RT. Following fixation, wells were washed 3× in PBS and then permeabilized with 0.2% (*v/v*) Triton X-100 in PBS for 4 min at RT. Cells were washed 3× in PBS and then stained for 20 min at RT with 2 drops/mL of NucBlue Fixed Cell Strain ReadyProbes (Invitrogen, Carlsbad, CA, USA) and ActinGreen 488 Ready Probes (Invitrogen) to stain DNA and actin, respectively. Cells were washed a final 3× with PBS, and then the coverslips were mounted onto glass slides in ProLong Gold antifade mounting medium (Invitrogen). Samples were viewed with a Leica SP8 confocal laser-scanning microscope (Leica, Wetzlar, Germany) for image acquisition. Representative confocal micrographs of 1024 × 1024 pixels (pixel size: 103.3 nm) were acquired and assembled using Fiji (with Bio-Formats Plugin)^45^.

### Measurement of cell cycle markers in HCT116 and HT-29

To assess whether *F.* *sphaericum* sp. nov. affects the number of human colon epithelial cells in distinct cell cycle phases, we used the Cell Cycle In-Cell ELISA IR Kit from Abcam (Abcam Limited, Cambridge, UK) according to the manufacturer’s instructions. Tests were performed in HCT116 or HT-29 cell lines co-incubated with *F.* *sphaericum* sp. nov. SB021 at MOI 1:100 or a no bacteria control for 24 h. Absorbance readings were obtained with an Odyssey Scanner (LI-COR Biotechnology, Lincoln, NE, USA) at 595 nm, 700 nm, and 800 nm to capture the intensity of Janus Green Stain (whole cell stain), IRDye680 corresponding to Cdk2 (pTyr15) protein signal, and IRDye800 corresponding to Histone H3 (pSer10) protein signal, respectively. To account for uneven cell seeding between wells, background subtracted intensities for IRDye680 and IRDye800 were normalized to the Janus Green Stain value of the corresponding well. This experiment was executed in triplicates. Statistical comparison was calculated by applying a Welch’s t-test using GraphPad Prism v7.0 Software (GraphPad Software).

### Analysis of chemokine and cytokine induction in HCT116

To determine the immunologic impact of *Fusobacterium* presence on HCT116 human colon epithelial cells, we utilized the Multi-Analyte ELISArray kits protocol version 1.4 from SABiosciences (Qiagen, Valencia, CA, USA) for the common human chemokines and inflammatory human cytokines panels according to the manufacturer’s instructions. Tests were performed with the following samples after a 4-h co-incubation with HCT116 (low passage, <10): *F.* *sphaericum* sp. nov. SB021 at MOI of 1:100 (live and oxygen-exposed), *F.* *sphaericum* sp. nov. SB021 cell-free supernatant, *F. nucleatum* subsp. *animalis* SB010 at MOI 1:100 (live), a negative control (HCT116 only), and a positive control (cocktail of antibodies). Supernatants were centrifuged at 1,000 × g for 10 min to remove any particulate material, filter sterilized, and 50 μl of each experimental sample was added to the array with the specific capture antibodies (IL-1α, IL-1β, IL-2, IL-4, IL-6, IL-8, IL-12, IL-17α, IFN-γ, TNF-α and GM CSF; IL-8, MCP-1, RANTES, MIP-1a, IP-10, I-TAC, MIG, Eotaxin, TARC, MDC, GROα), followed by an incubation at room temperature (RT) for 2 h. Subsequently, the wells were buffer washed, and 100 μl of diluted biotinylated detection antibodies were added for a 1 h-incubation at RT in the dark. After another washing procedure, the wells were filled with 100 μl of Avidin-horseradish peroxidase (HRP) and incubated at RT for 30 min, again in the dark. We then added development and stop solutions to detect absorbance changes immediately after termination of the experiment with a microplate reader (Synergy H4 hybrid Reader, BioTek) at 570 nm and 450 nm, visualized with Gen5 Synergy H4 software (Version 3.08, BioTek). Raw data of the absorbance reading were normalized to the cell counts of HCT116 cells incubated without bacteria.

For quantification of immunologic effects, we utilized the Single-Analyte ELISArray kit protocol version 1.4. from SABiosciences (Qiagen, Valencia, CA, USA) for the chemokine IL-8 according to the manufacturer’s instructions. Co-cultures were set up as described above with *F.* *sphaericum* sp. nov. SB021 at MOI of 1:100 (live), *F.* *sphaericum* sp. nov. SB021 cell-free supernatant, *F. nucleatum* subsp. *animalis* SB010 at MOI 1:100 (live), *Escherichia coli* TOP10, or a positive control (HCT116 only) and incubated for 4 h or 24 h. Supernatants were centrifuged at 1000 × g for 10 min and filter sterilized subsequently. A serial dilution of the antigen standard was prepared and 50 μl of each sample was added to the array, followed by an incubation for 2 h at RT. Similar to the Multi-Analyte kit, the array was then incubated with detection antibodies and subsequently Avidin-HRP to determine the attachment of detection antibodies to the chemokine. After the color development, changes in absorbance were read at 570 nm and 450 nm and raw data were equally normalized. This experiment was executed in duplicates. Statistical comparison was calculated by applying a one-way ANOVA using GraphPad Prism v7.0 Software (GraphPad Software).

### Analysis within human metagenomic specimens

Starting from the *Fs* SB021 isolate, we identified the corresponding SGB in the Jan21 markers MetaPhlAn database^46^ using the ‘phylophlan_metagenomic’ subroutine of PhyloPhlAn 3 (v3.0).^47^ The genome was assigned to SGB6028 (MASH distance 3.2%). This is an SGB for which marker genes computed in MetaPhlAn are highly conflicting with markers from three other SGBs in terms of specificity, for this reason the SGB is considered as a group with other three SGBs, and it is called SGB59307_group. *Fusobacterium perfoetens* ATCC 29250 was assigned with the same procedure to SGB29032 (MASH distance 0). Human metagenomic samples were processed with MetaPhlAn 4 (v4.beta.1) using the vJan21 markers database. The detection of SGB59307_group and SGB29032 is used to report, respectively, the presence and relative abundance of *Fs* SB021 and *Fusobacterium perfoetens* ATCC 29250. For samples originating from cohorts comparing patients with colorectal cancer to healthy controls (Supplementary Tables 8–9) in which SGB59307_group was detected, we compared the proportion of samples via a two-sample Z-test and the relative abundance of SGB59307_group using a Welch’s t-test based on patient status.

## Supplementary Material

Supplemental Material

## Data Availability

*F.* *sphaericum* sp. nov. strain SB021 whole-genome sequence is available in GenBank under the accession number SAMN15202580 and methylome is available in the Restriction Enzyme Database (REBASE) under organism number 39739.
