# Supplementary material for: Fusobacterium sphaericum sp. nov., isolated from a human colon tumor adheres to colonic epithelial cells and induces IL-8 secretion
Source: Gut Microbes. 2024 Dec 25;17(1):2442522. doi: 10.1080/19490976.2024.2442522 (PMC12931714; doi:10.1080/19490976.2024.2442522)
Supplement: Supplemental Material [file KGMI_A_2442522_SM5905.zip › Supp figures.docx]

***Supplementary Figures***

***
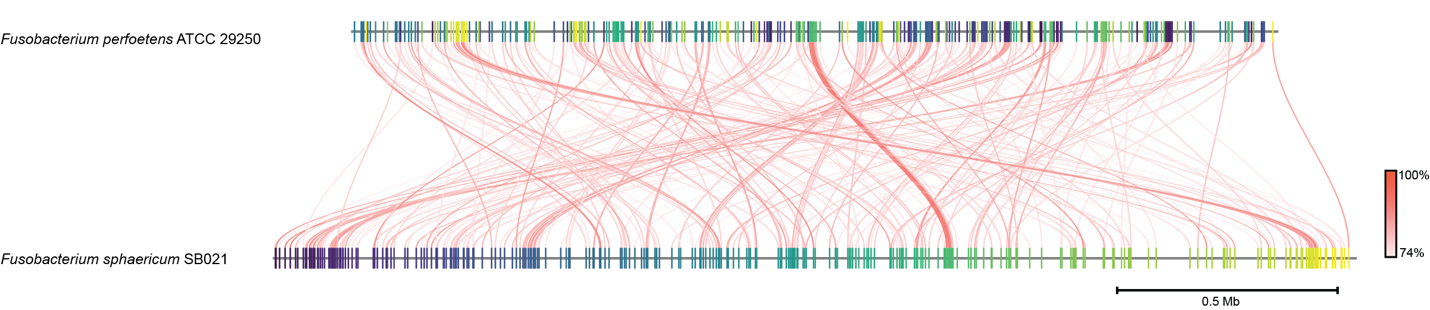
***

**Supplementary Fig. 1: Genomic architecture of *Fusobacterium sphaericum sp. nov.* SB021 as compared to *Fusobacterium perfoetens* ATCC 29250**

Visualization of fastANI alignment between *Fusobacterium sphaericum sp. nov.* SB021 and its nearest phylogenetic neighbor, *Fusobacterium perfoetens* ATCC 29250, where each red line segment denotes a reciprocal mapping of the 275 orthologous matches between the two genomes, indicating evolutionarily conserved regions.


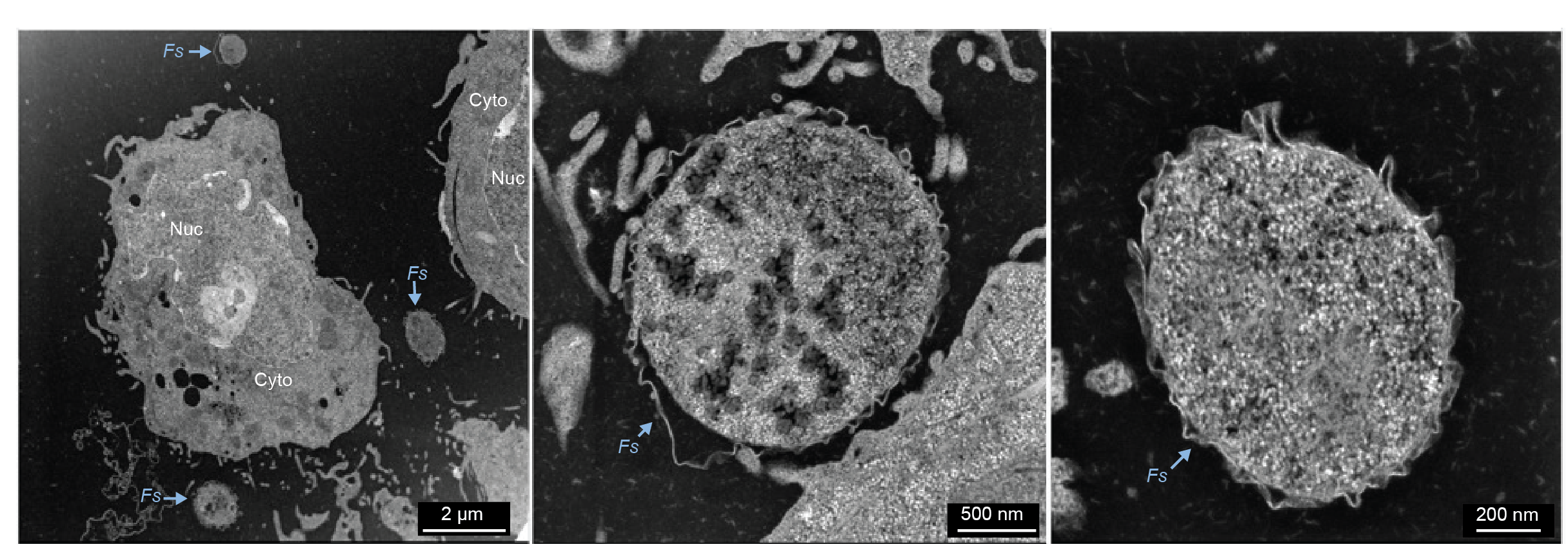


**Supplementary Fig. 2: Scanning Transmission Electron Microscopy of** ***Fusobacterium sphaericum sp. nov.***

Scanning transmission electron microscopy image of *Fusobacterium sphaericum sp. nov.* SB021 in co-culture with human colon cancer epithelial cells, HCT116. Eukaryotic nucleus (Nuc) and cytoplasm (Cyto) are labeled. *Fs* bacterial cells are indicated with blue label and arrow. Scale bars for individual panels are shown in bottom right-hand corner of each.


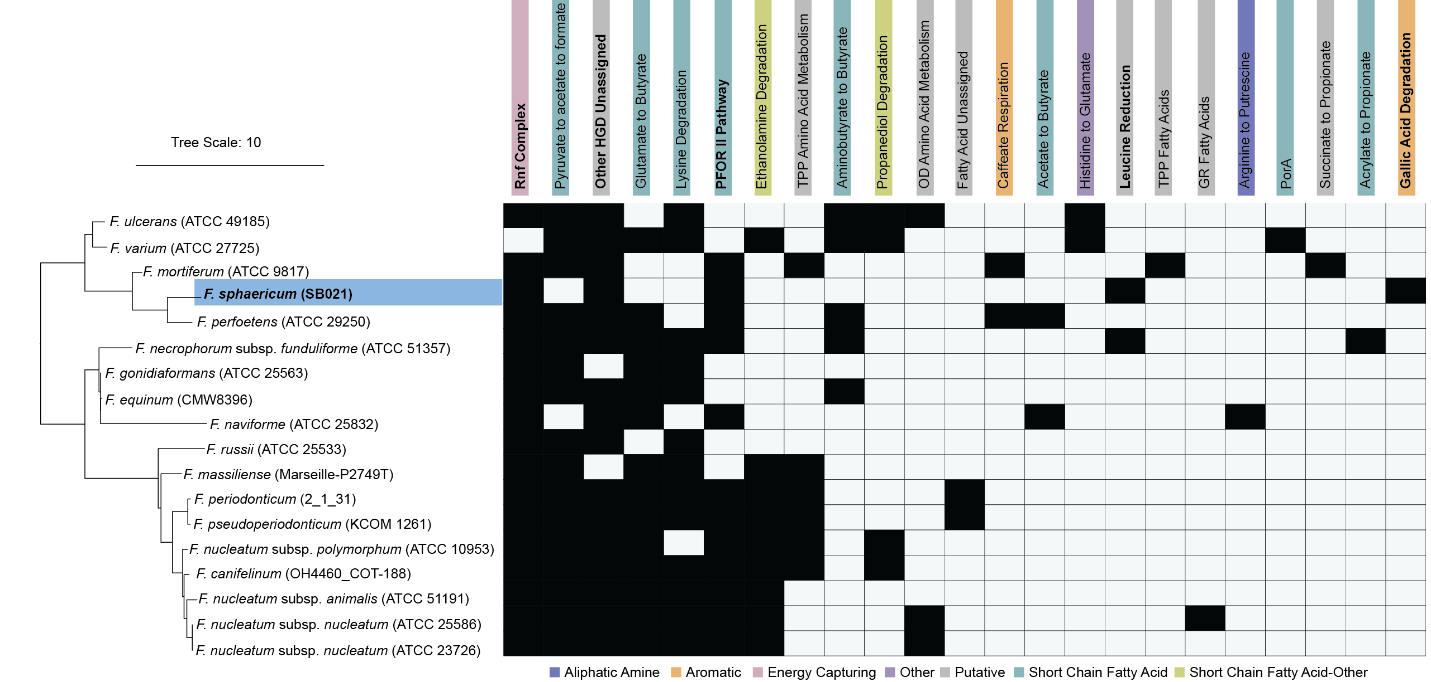


**Supplementary Fig. 3: Primary metabolic pathways in *Fusobacterium sphaericum sp. nov.***

Presence (black) versus absence (white) plot of primary metabolic pathways identified by gutSMASH^21^ across *Fusobacterium spp.* genomes, ordered by a kSNP^12^ maximum-likelihood whole-genome phylogenetic tree with *F. sphaericum sp. nov.* SB021 highlighted in blue.


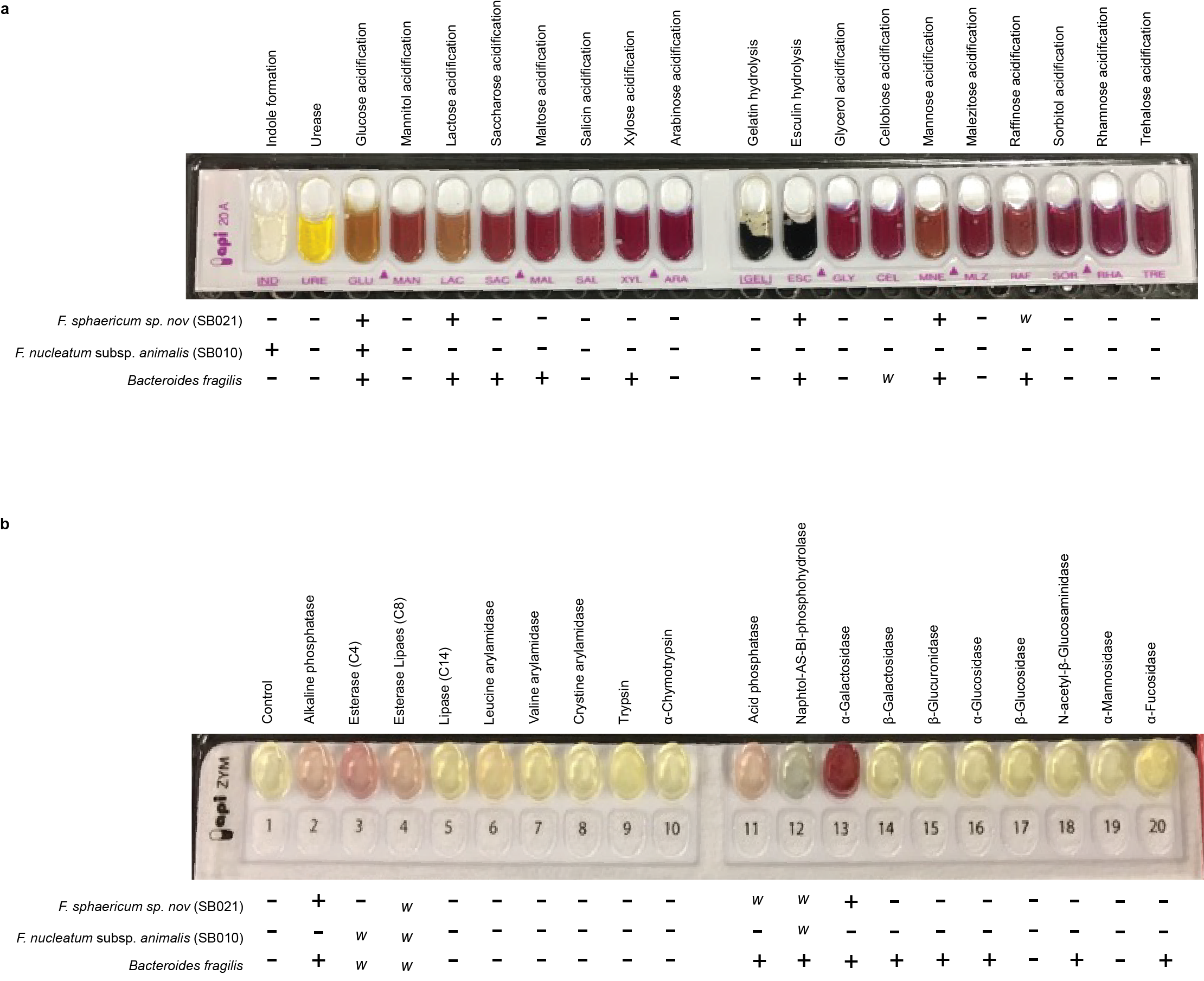


**Supplementary Fig. 4: API metabolic characterization of *Fusobacterium sphaericum* *sp. nov.***

*F. sphaericum sp. nov.* (*Fs*) **a)** metabolic and **b)** chemotaxonomic characteristics assayed by API 20A and API ZYM detection kits, respectively. Images are representative results for *Fs* SB021. Results for *Fs* SB021, *Fusobacterium nucleatum* subsp. *animalis* SB010, and *Bacteroides fragilis* are summarized below each API strip, where (+) indicates a positive result, (-) a negative result, and “*w*” a weakly positive result.

**
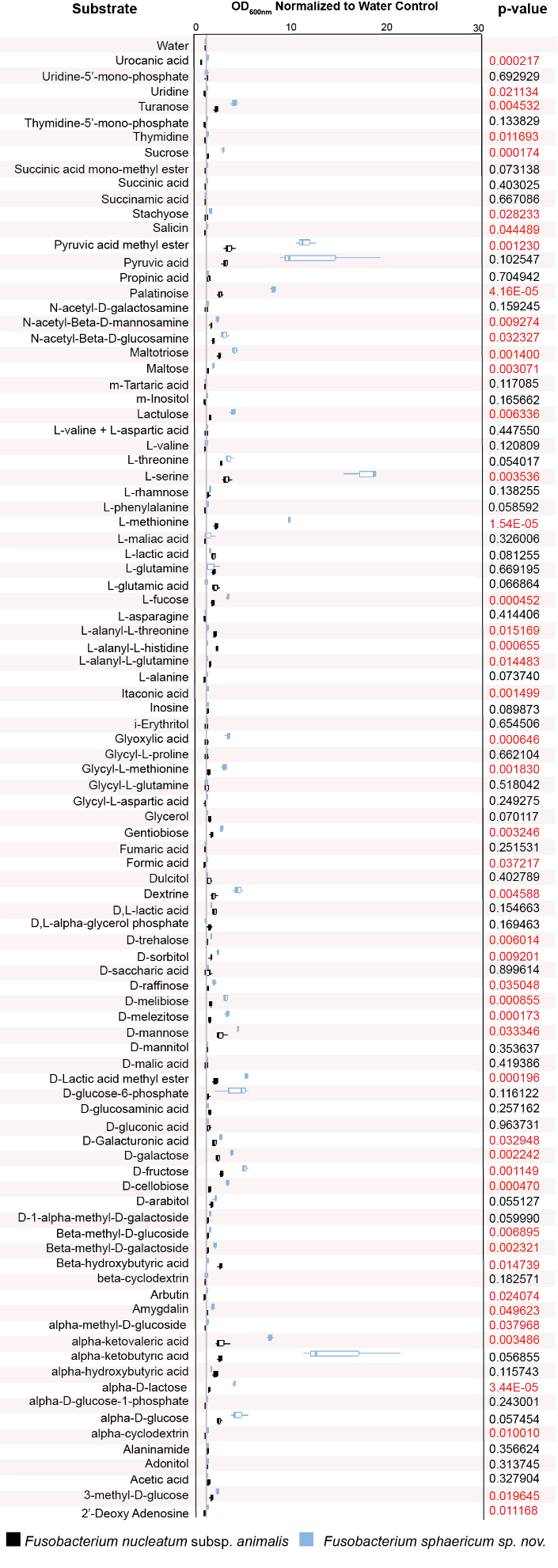
**

**Supplementary Fig. 5: Biolog anaerobe identification test panel characterization of *F. sphaericum sp. nov.* substrate utilization**

*F. sphaericum sp. nov.* substrate utilization assayed by Biolog Anaerobe Identification Test Panel (AN) assay plate. Graphs show results for *F. sphaericum sp. nov.* SB021 (blue) and *Fusobacterium nucleatum* subsp. *animalis* (*Fna*) SB010 (black) normalized to internal water control, in triplicate. Statistical analysis performed via a two-sided T-test. All p-values less than 0.05 are highlighted in red.


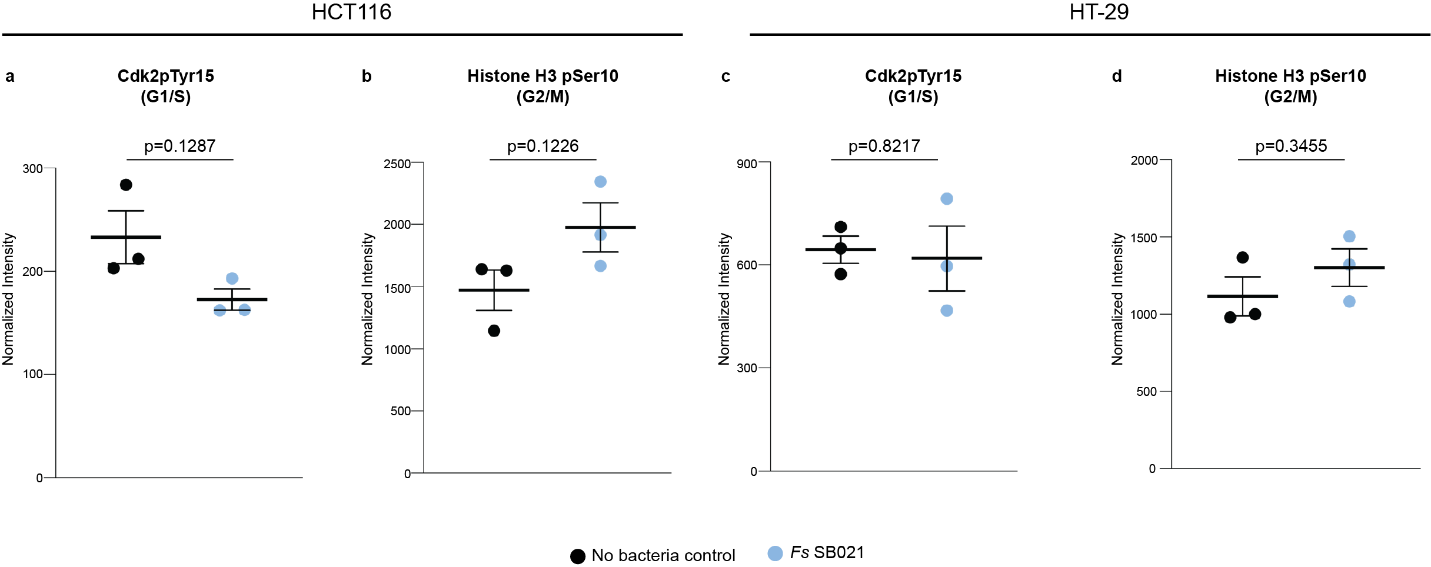


**Supplementary Fig. 6: Normalized number of human colon epithelial cells in distinct cell cycle stages in the presence of *F. sphaericum sp. nov.* SB021**

The in vitro effect of *F. sphaericum sp. nov.* on the number of human colon cancer epithelial cells in distinct cell cycle stages was measured via cell cycle In-Cell ELISA of **a-b)** HCT116 and **c-d)** HT-29 cell lines in the presence of *Fs* SB021 (blue) as compared to a no bacteria control (black). Graphs show the normalized intensity, in triplicate, for **a,c)** phosphorylated Cdk2, a marker elevated in the G1/S cell cycle phase, and **b,d)** phosphorylated histone H3, a marker elevated during the G2/M phase. To account for differences in cell seeding, intensity values for a-d were normalized by total cell count as measured by a Janus Green whole cell stain, per the manufacturer’s instructions. Statistical analysis performed via a Welch’s T-test.


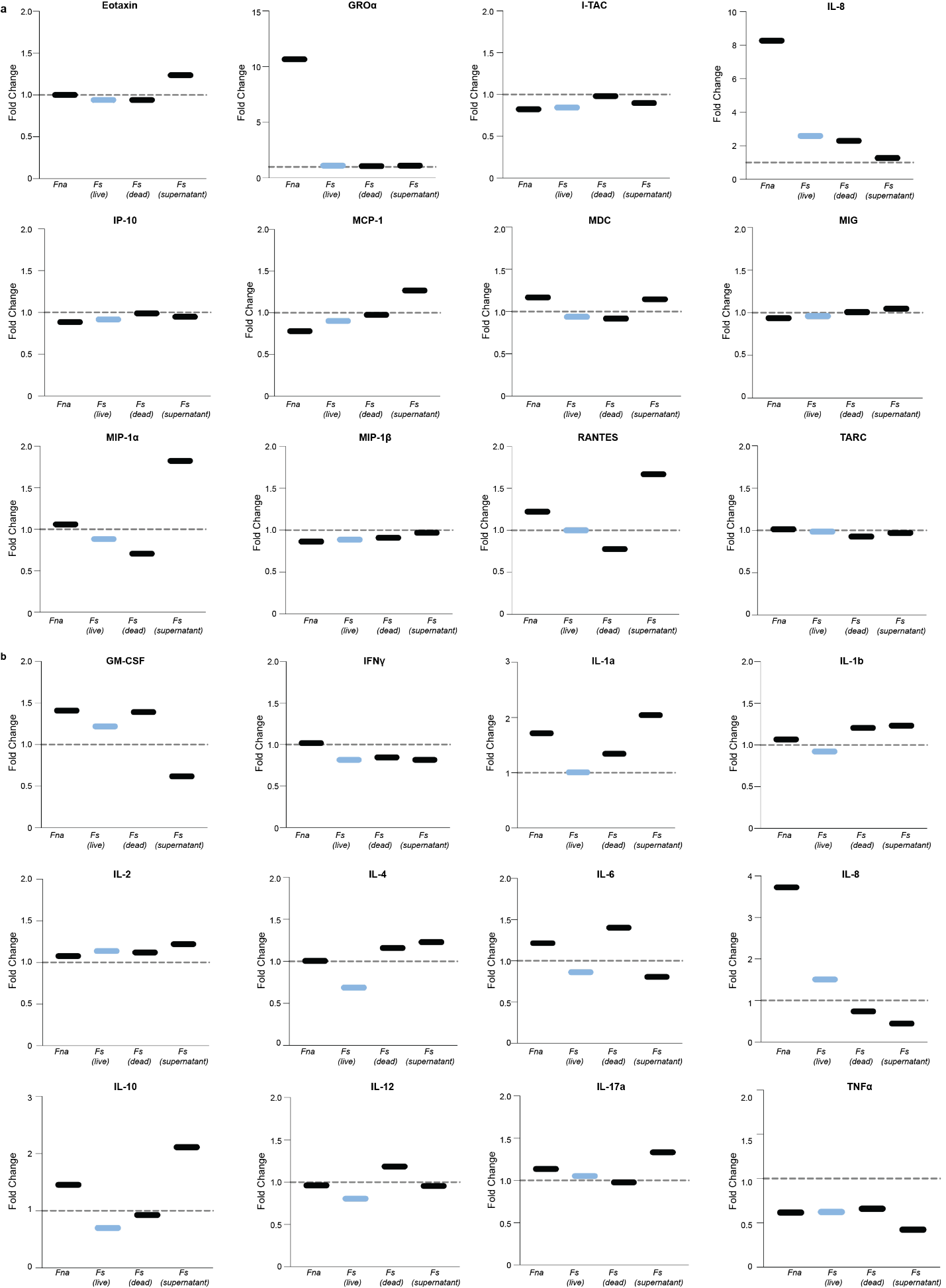


**Supplementary Fig. 7: Cytokine and chemokine induction in HCT116 cells co-incubated with *F. sphaericum sp. nov.* SB021**

Measurements of **a**) common chemokine and **b**) inflammatory cytokine levels measured via the Multi-Analyte ELISArray panels from HCT116 cells co-incubated with different *Fusobacterium* species at a multiplicity of infection (MOI) of 100 or antibody cocktail control, normalized to a no-bacteria HCT116 control. Bacterial species used included *F. nucleatum* subsp. *animalis* (*Fna*) SB010 and *F. sphaericum sp. nov.* (*Fs*) SB021 live cells or oxygen-exposed dead cells. An additional co-incubation with *Fs* SB021 supernatant alone was also performed.

***Supplementary Video Legends***

**Supplementary Video 1: Confocal microscopy imaging of *F. sphaericum sp. nov.* SB021 co-cultured with colon cancer epithelial cells**

Video of the Z-stack from confocal laser scanning microscopy of colon cancer epithelial cells (HCT116) co-incubated with *F. sphaericum sp. nov.* (*Fs*) SB021 in vitro. Bacterial cell staining: FM4-64X (red) and DAPI (blue). Eukaryotic cell staining: DAPI (blue), actin (green). Video is available via figshare at <https://doi.org/10.6084/m9.figshare.27698502.v1>.
