# Supplementary material for: Fusobacterium sphaericum sp. nov., isolated from a human colon tumor adheres to colonic epithelial cells and induces IL-8 secretion
Source: Gut Microbes. 2024 Dec 25;17(1):2442522. doi: 10.1080/19490976.2024.2442522 (PMC12931714; doi:10.1080/19490976.2024.2442522)
Supplement: Supplemental Material [file KGMI_A_2442522_SM5905.zip › 2024_11_18_ST1_ST9 clean.docx]

**Supplementary Table 1: BLASTn analysis of *F. sphaericum sp. nov.* SB021 rRNA gene sequence**

| **Description** | **Scientific Name** | **Max Score** | **Total Score** | **Query Coverage** | **E value** | **Percent**  **Identity** | **Accession Length** | **Accession** |
| --- | --- | --- | --- | --- | --- | --- | --- | --- |
| *Fusobacterium perfoetens* strain DSM 105865 16S ribosomal RNA gene, partial sequence | *Fusobacterium perfoetens* | 2390 | 2390 | 98% | 0 | 96.23 | 1492 | MN537503.1 |
| *Fusobacterium sp.* strain JC048 16S ribosomal RNA gene, partial sequence | *Fusobacterium sp.* | 2399 | 2399 | 99% | 0 | 96.07 | 1477 | MH746937.1 |
| *Fusobacterium sp.* strain IOR10 16S ribosomal RNA gene, partial sequence | *Fusobacterium sp.* | 2268 | 2268 | 94% | 0 | 95.99 | 1396 | MN630848.2 |
| *Fusobacterium sp.* B57-13 16S ribosomal RNA gene, partial sequence | *Fusobacterium sp.* B57-13 | 2146 | 2146 | 89% | 0 | 95.93 | 1324 | KU533800.1 |
| *Fusobacterium perfoetens* strain ATCC 29250 16S ribosomal RNA, partial sequence | *Fusobacterium perfoetens* | 2290 | 2290 | 97% | 0 | 94.55 | 1456 | NR_044688.3 |
| *Fusobacterium perfoetens* strain ATCC 29250 16S ribosomal RNA gene, partial sequence | *Fusobacterium perfoetens* | 2290 | 2290 | 97% | 0 | 94.55 | 1457 | M58684.2 |
| *Fusobacterium mortiferum* ATCC 9817 chromosome, complete genome | *Fusobacterium mortiferum* ATCC 9817 | 2187 | 17489 | 100% | 0 | 93.45 | 2716766 | CP028102.1 |
| [*Clostridium*] rectum gene for 16S ribosomal RNA, partial sequence, strain: JCM 1412 | *Fusobacterium mortiferum* | 2176 | 2176 | 99% | 0 | 93.42 | 1469 | LC053839.1 |
| *Fusobacterium mortiferum* strain DSM 19809 16S ribosomal RNA, partial sequence | *Fusobacterium mortiferum* | 2165 | 2165 | 99% | 0 | 93.39 | 1482 | NR_117734.1 |
| *Fusobacterium mortiferum* strain DSM 108838 16S ribosomal RNA gene, partial sequence | *Fusobacterium mortiferum* | 2158 | 2158 | 98% | 0 | 93.37 | 1456 | MN537550.1 |
| *Fusobacterium hominis* strain NSJ-57 chromosome, complete genome | *Fusobacterium hominis* | 2165 | 19483 | 100% | 0 | 93.18 | 2072767 | CP060637.1 |
| *Fusobacterium sp*. DJF_B100 16S ribosomal RNA gene, partial sequence | *Fusobacterium sp.* DJF_B100 | 2091 | 2091 | 97% | 0 | 93.00 | 1431 | EU728711.1 |
| *Fusobacterium mortiferum* strain NCIMB 10651 16S ribosomal RNA, partial sequence | *Fusobacterium mortiferum* | 2135 | 2135 | 100% | 0 | 92.81 | 1505 | NR_119089.1 |
| *Clostridium* rectum 16S rRNA gene, strain NCIMB 10651 | *Fusobacterium mortiferum* | 2135 | 2135 | 100% | 0 | 92.81 | 1511 | X77850.1 |
| *Fusobacterium varium* strain NCTC10560 genome assembly, chromosome: 1 | *Fusobacterium varium* | 2121 | 14808 | 100% | 0 | 92.65 | 3302398 | LR134390.1 |
| *Fusobacterium ulcerans* strain NCTC12112 genome assembly, chromosome: 1 | *Fusobacterium ulcerans* | 2121 | 14786 | 100% | 0 | 92.65 | 3539037 | LS483487.1 |
| *Fusobacterium varium* ATCC 27725 chromosome, complete genome | *Fusobacterium varium* ATCC 27725 | 2121 | 14841 | 100% | 0 | 92.65 | 3303644 | CP028103.1 |
| *Fusobacterium varium* isolate KR001_HAM_0062 chromosome, complete genome | *Fusobacterium varium* | 2121 | 14841 | 100% | 0 | 92.65 | 3425653 | CP107203.1 |
| *Fusobacterium sp.* P6 Po strain Fusobacterium gastrosuis sp. nov. CDW1, P6 Po genome assembly, chromosome: chr | *Fusobacterium gastrosuis* | 2119 | 10579 | 100% | 0 | 92.64 | 1816688 | LT607734.1 |
| *Fusobacterium gastrosuis* strain CDW1 16S ribosomal RNA, complete sequence | *Fusobacterium gastrosuis* | 2119 | 2119 | 100% | 0 | 92.64 | 1506 | NR_146837.2 |
| *Fusobacterium gastrosuis* partial 16S rRNA gene, type strain CDW1T, isolate P6 Po | *Fusobacterium gastrosuis* | 2119 | 2119 | 100% | 0 | 92.64 | 1512 | LN906797.3 |
| *Fusobacterium varium* strain JCM 6320 16S ribosomal RNA, partial sequence | *Fusobacterium varium* | 2109 | 2109 | 99% | 0 | 92.62 | 1469 | NR_113384.1 |
| *Fusobacterium ulcerans* strain ATCC 49185 chromosome, complete genome | *Fusobacterium ulcerans* | 2115 | 14808 | 100% | 0 | 92.58 | 3537675 | CP028105.1 |
| *Fusobacterium varium* Fv113-g1 DNA, complete genome | *Fusobacterium varium* | 2115 | 14775 | 100% | 0 | 92.58 | 3965155 | AP017968.1 |
| *Fusobacterium varium* gene for 16S ribosomal RNA, partial sequence, strain: JCM 3722 | *Fusobacterium varium* | 2087 | 2087 | 98% | 0 | 92.55 | 1457 | AB595132.1 |
| *Fusobacterium ulcerans* ATCC 49185 isolate Fusobacterium ulcerans 81A6 genome assembly, chromosome: 1 | *Fusobacterium ulcerans* ATCC 49185 | 2109 | 14769 | 100% | 0 | 92.51 | 3839419 | LR215979.1 |
| *Fusobacterium sp.* CSL-7530 16S ribosomal RNA gene, partial sequence; 16S-23S ribosomal RNA intergenic spacer, complete sequence; and 23S ribosomal RNA gene, partial sequence | *Fusobacterium sp.* CSL-7530 | 2073 | 2073 | 98% | 0 | 92.51 | 2163 | EU597748.1 |
| *Fusobacterium sp.* strain B2-O-100 16S ribosomal RNA gene, partial sequence | *Fusobacterium sp.* | 2071 | 2071 | 98% | 0 | 92.51 | 1445 | OK325759.1 |
| *Fusobacterium sp.* strain B2-F-105 16S ribosomal RNA gene, partial sequence | *Fusobacterium sp.* | 2067 | 2067 | 98% | 0 | 92.50 | 1443 | OK271567.1 |
| *Fusobacterium ulcerans* strain PH5-7 16S ribosomal RNA gene, partial sequence | *Fusobacterium ulcerans* | 2076 | 2076 | 98% | 0 | 92.47 | 1451 | KR822462.1 |
| *Fusobacterium varium* strain TW1-2 16S ribosomal RNA gene, partial sequence | *Fusobacterium varium* | 2065 | 2065 | 98% | 0 | 92.44 | 1445 | KR822478.1 |
| *Fusobacterium sp.* Marseille-P3599 partial 16S rRNA gene, strain Marseille-P3599 | *Fusobacterium sp.* Marseille-P3599 | 2047 | 2047 | 97% | 0 | 92.44 | 1430 | LT671592.1 |
| *Fusobacterium ulcerans* strain H_58 16S ribosomal RNA gene, partial sequence | *Fusobacterium ulcerans* | 2098 | 2098 | 100% | 0 | 92.38 | 1472 | MG428853.1 |
| *Fusobacterium ulcerans* strain PM5-10 16S ribosomal RNA gene, partial sequence | *Fusobacterium ulcerans* | 2037 | 2037 | 97% | 0 | 92.36 | 1431 | KR822463.1 |
| *Fusobacterium equinum* strain JCM 11174 16S ribosomal RNA, partial sequence | *Fusobacterium equinum* | 2082 | 2082 | 99% | 0 | 92.28 | 1469 | NR_113379.1 |
| *Fusobacterium sp.* N4-2 gene for 16S ribosomal RNA, partial sequence | *Fusobacterium sp.* N4-2 | 2036 | 2036 | 97% | 0 | 92.24 | 1434 | LC159198.1 |
| *Fusobacterium sp.* TL12 gene for 16S rRNA, partial sequence | *Fusobacterium sp.* | 2036 | 2036 | 97% | 0 | 92.24 | 1433 | KC586280.1 |
| *Fusobacterium pseudoperiodonticum* strain KCOM 1277 chromosome, complete genome | *Fusobacterium pseudoperiodonticum* | 2085 | 10396 | 100% | 0 | 92.23 | 2324076 | CP024701.1 |
| Uncultured *Fusobacterium sp.* clone SL13 16S ribosomal RNA gene, partial sequence | Uncultured *Fusobacterium sp.* | 2085 | 2085 | 99% | 0 | 92.23 | 1471 | JN680680.1 |
| *Fusobacterium sp.* feline oral taxon 120 clone TE006 16S ribosomal RNA gene, partial sequence | *Fusobacterium sp.* feline oral taxon 120 | 2078 | 2078 | 99% | 0 | 92.19 | 1497 | KM461985.1 |
| *Fusobacterium gonidiaformans* ATCC 25563 chromosome, complete genome | *Fusobacterium gonidiaformans* ATCC 25563 | 2082 | 12493 | 100% | 0 | 92.18 | 1678881 | CP028106.1 |
| F*usobacterium pseudoperiodonticum* strain KCOM 1261 chromosome, complete genome | *Fusobacterium pseudoperiodonticum* | 2080 | 10368 | 100% | 0 | 92.18 | 2372880 | CP024699.1 |
| *Fusobacterium pseudoperiodonticum* strain KCOM 2653 chromosome, complete genome | *Fusobacterium pseudoperiodonticum* | 2080 | 10380 | 100% | 0 | 92.17 | 2991059 | CP024705.1 |
| *Fusobacterium pseudoperiodonticum* strain KCOM 2555 chromosome, complete genome | *Fusobacterium pseudoperiodonticum* | 2080 | 10380 | 100% | 0 | 92.17 | 2477385 | CP024704.1 |
| *Fusobacterium pseudoperiodonticum* strain KCOM 2305 chromosome, complete genome | *Fusobacterium pseudoperiodonticum* | 2080 | 10396 | 100% | 0 | 92.17 | 2273832 | CP024703.1 |
| *Fusobacterium pseudoperiodonticum* strain KCOM 1282 chromosome, complete genome | *Fusobacterium pseudoperiodonticum* | 2080 | 10391 | 100% | 0 | 92.17 | 2216236 | CP024702.1 |
| *Fusobacterium pseudoperiodonticum* strain KCOM 1283 chromosome, complete genome | *Fusobacterium pseudoperiodonticum* | 2080 | 10368 | 100% | 0 | 92.17 | 2222370 | CP024698.1 |
| *Fusobacterium equinum* strain VPB 4027 16S ribosomal RNA, partial sequence | *Fusobacterium equinum* | 2080 | 2080 | 99% | 0 | 92.17 | 1472 | NR_028933.1 |
| *Fusobacterium necrophorum* strain FDAARGOS_565 chromosome, complete genome | *Fusobacterium necrophorum* | 2076 | 12410 | 100% | 0 | 92.12 | 2678402 | CP033837.1 |
| *Fusobacterium necrophorum* subsp. *necrophorum* strain ATCC 25286 chromosome, complete genome | *Fusobacterium necrophorum* subsp. *necrophorum* | 2076 | 12416 | 100% | 0 | 92.12 | 2678415 | CP034842.1 |
| *Fusobacterium sp.* canine oral taxon 439 strain OH1383 16S ribosomal RNA gene, partial sequence | *Fusobacterium sp.* canine oral taxon 439 | 2065 | 2065 | 99% | 0 | 92.12 | 1462 | KF030231.1 |
| *Fusobacterium sp.* Marseille-P2749 partial 16S rRNA gene, strain Marseille-P2749 | *Fusobacterium massiliense* | 2073 | 2073 | 99% | 0 | 92.11 | 1469 | LT576389.1 |
| *Fusobacterium massiliense* strain Marseille-P2749 16S ribosomal RNA, partial sequence | *Fusobacterium massiliense* | 2073 | 2073 | 99% | 0 | 92.11 | 1469 | NR_179530.1 |
| *Fusobacterium necrophorum* strain JCM 3718 16S ribosomal RNA, partial sequence | *Fusobacterium necrophorum* | 2065 | 2065 | 99% | 0 | 92.08 | 1469 | NR_114400.1 |
| *Fusobacterium periodonticum* gene for 16S ribosomal RNA, partial sequence | *Fusobacterium periodonticum* | 2063 | 2063 | 99% | 0 | 92.07 | 1467 | AB910749.1 |
| *Fusobacterium necrophorum* subsp. *funduliforme* strain F1260 chromosome, complete genome | *Fusobacterium necrophorum* subsp. *funduliforme* | 2071 | 12371 | 100% | 0 | 92.05 | 2288480 | CP019306.1 |
| *Fusobacterium necrophorum* subsp. *funduliforme* strain F1291 chromosome, complete genome | *Fusobacterium necrophorum* subsp. *funduliforme* | 2071 | 12399 | 100% | 0 | 92.05 | 2135983 | CP018196.1 |
| Uncultured *Fusobacterium sp.* clone VE32H03 16S ribosomal RNA gene, partial sequence | Uncultured *Fusobacterium sp.* | 2034 | 2034 | 98% | 0 | 92.04 | 1446 | GQ179686.1 |
| *Fusobacterium necrophorum* subsp. *funduliforme* strain 1_1_36S chromosome, complete genome | *Fusobacterium necrophorum* subsp. *funduliforme* | 2065 | 12388 | 100% | 0 | 91.98 | 2286018 | CP028107.1 |
| *Fusobacterium periodonticum* strain 2_1_31 chromosome, complete genome | *Fusobacterium periodonticum* | 2063 | 10319 | 100% | 0 | 91.98 | 2541084 | CP028108.1 |
| *Fusobacterium pseudoperiodonticum* strain KCOM 1263 chromosome, complete genome | *Fusobacterium pseudoperiodonticum* | 2063 | 10319 | 100% | 0 | 91.98 | 2651118 | CP024700.1 |
| *Fusobacterium pseudoperiodonticum* strain KCOM 1262 chromosome, complete genome | *Fusobacterium pseudoperiodonticum* | 2063 | 10319 | 100% | 0 | 91.98 | 2643576 | CP024731.1 |
| *Fusobacterium necrophorum* subsp. *necrophorum* gene for 16S ribosomal RNA, partial sequence, strain: JCM 3716 | *Fusobacterium necrophorum* subsp. *necrophorum* | 2054 | 2054 | 99% | 0 | 91.95 | 1469 | AB971800.1 |
| *Fusobacterium necrophorum* subsp. *funduliforme* strain Fn524 16S ribosomal RNA, partial sequence | *Fusobacterium necrophorum* subsp. *funduliforme* | 2054 | 2054 | 99% | 0 | 91.95 | 1469 | NR_104683.1 |
| *Fusobacterium sp.* oral clone EX123 16S ribosomal RNA gene, partial sequence | *Fusobacterium sp.* oral clone EX123 | 2063 | 2063 | 99% | 0 | 91.85 | 1492 | AY134897.1 |
| Uncultured *Fusobacterium sp.* clone M10-6 16S ribosomal RNA gene, partial sequence | Uncultured *Fusobacterium sp.* | 2036 | 2036 | 99% | 0 | 91.84 | 1468 | JF975757.1 |
| Uncultured *Fusobacterium sp.* clone H10-47 16S ribosomal RNA gene, partial sequence | Uncultured *Fusobacterium sp.* | 2036 | 2036 | 99% | 0 | 91.84 | 1468 | JF975740.1 |
| *Fusobacterium necrophorum* strain ATCC 25286 16S ribosomal RNA, partial sequence | *Fusobacterium necrophorum* | 2030 | 2030 | 98% | 0 | 91.83 | 1455 | NR_042365.1 |
| *Fusobacterium nucleatum* E9_12 16S ribosomal RNA gene, partial sequence | *Fusobacterium nucleatum* E9_12 | 2034 | 2034 | 98% | 0 | 91.81 | 1474 | AF481217.1 |
| *Fusobacterium necrophorum* canine oral taxon 190 clone QD026 16S ribosomal RNA gene, partial sequence | *Fusobacterium necrophorum* | 2039 | 2039 | 99% | 0 | 91.80 | 1464 | JN713357.1 |
| Uncultured *Fusobacterium sp.* clone M10-63 16S ribosomal RNA gene, partial sequence | Uncultured *Fusobacterium sp.* | 2030 | 2030 | 99% | 0 | 91.77 | 1468 | JF975766.1 |
| *Fusobacterium hwasookii* ChDC F174, complete genome | *Fusobacterium hwasookii*  ChDC F174 | 2047 | 10164 | 100% | 0 | 91.76 | 2463707 | CP013331.1 |
| *Fusobacterium nucleatum* subsp. *polymorphum* strain KCOM 1275, complete genome | *Fusobacterium nucleatum* subsp. *polymorphum* | 2041 | 10164 | 100% | 0 | 91.70 | 2521394 | CP022123.1 |
| *Fusobacterium nucleatum* subsp. *polymorphum* strain KCOM 1260 (=ChDC F218) genome | *Fusobacterium nucleatum* subsp. *polymorphum* | 2041 | 10180 | 100% | 0 | 91.69 | 2635767 | CP021934.1 |
| *Fusobacterium nucleatum* subsp. *polymorphum* strain ChDC F319 genome | *Fusobacterium nucleatum* subsp. *polymorphum* | 2041 | 10197 | 100% | 0 | 91.69 | 2469901 | CP013328.1 |
| *Fusobacterium hwasookii* ChDC F206, complete genome | *Fusobacterium hwasookii*  ChDC F206 | 2041 | 10175 | 100% | 0 | 91.69 | 2424685 | CP013336.1 |
| *Fusobacterium nucleatum* subsp. *polymorphum* strain THCT15E1 chromosome, complete genome | *Fusobacterium nucleatum* subsp. *polymorphum* | 2041 | 10147 | 100% | 0 | 91.69 | 2515213 | CP071094.1 |
| *Fusobacterium hwasookii* strain THCT14E2 chromosome, complete genome | *Fusobacterium hwasookii* | 2041 | 10197 | 100% | 0 | 91.69 | 2287114 | CP071092.1 |
| *Fusobacterium hwasookii s*train KCOM 1253 chromosome, complete genome | *Fusobacterium hwasookii* | 2041 | 10180 | 100% | 0 | 91.69 | 2351755 | CP060115.1 |
| *Fusobacterium hwasookii* strain KCOM 1249 chromosome, complete genome | *Fusobacterium hwasookii* | 2041 | 10154 | 100% | 0 | 91.69 | 2351860 | CP060112.1 |
| *Fusobacterium sp.* oral clone EX162 16S ribosomal RNA gene, partial sequence | *Fusobacterium sp.* oral clone EX162 | 2039 | 2039 | 99% | 0 | 91.69 | 1481 | AY134900.1 |
| *Fusobacterium necrophorum* subsp. *funduliforme* strain DSM 19678 16S ribosomal RNA, partial sequence | *Fusobacterium necrophorum* subsp. *funduliforme* | 2036 | 2036 | 99% | 0 | 91.69 | 1487 | NR_115077.1 |
| *Fusobacterium hwasookii* ChDC F300, complete genome | *Fusobacterium hwasookii*  ChDC F300 | 2036 | 8478 | 100% | 0 | 91.63 | 2521574 | CP013334.1 |
| *Fusobacterium nucleatum* strain JD-Fn1 chromosome, complete genome | *Fusobacterium nucleatum* | 2036 | 10158 | 100% | 0 | 91.63 | 2470422 | CP116231.1 |
| *Fusobacterium nucleatum* subsp. *polymorphum* genome assembly NCTC10562, chromosome : 1 | *Fusobacterium nucleatum* subsp. *polymorphum* | 2036 | 10130 | 100% | 0 | 91.63 | 2443126 | LN831027.1 |
| *Fusobacterium nucleatum* subsp. *polymorphum* strain THCT7E2 chromosome, complete genome | *Fusobacterium nucleatum* subsp. *polymorphum* | 2036 | 10158 | 100% | 0 | 91.63 | 2547341 | CP071096.1 |
| *Fusobacterium nucleatum* subsp. *polymorphum* strain F0401 16S ribosomal RNA gene, partial sequence | *Fusobacterium nucleatum* subsp. *polymorphum* | 2034 | 2034 | 99% | 0 | 91.62 | 1480 | GU470910.1 |
| Uncultured *Fusobacterium sp.* clone EHFS1_S18a 16S ribosomal RNA gene, partial sequence | Uncultured *Fusobacterium sp.* | 2025 | 2025 | 99% | 0 | 91.61 | 1469 | EU071543.1 |
| Uncultured *Fusobacterium sp.* partial 16S rRNA gene, clone 303E05(oral) | Uncultured *Fusobacterium sp.* | 2041 | 2041 | 99% | 0 | 91.58 | 1482 | AM420105.1 |
| *Fusobacterium nucleatum* strain Fn12230 chromosome, complete genome | *Fusobacterium nucleatum* | 2030 | 10141 | 100% | 0 | 91.56 | 2421837 | CP053468.1 |
| *Fusobacterium nucleatum* strain FNP chromosome, complete genome | *Fusobacterium nucleatum* | 2030 | 10136 | 100% | 0 | 91.56 | 2651846 | CP117825.1 |
| *Fusobacterium nucleatum* subsp. *polymorphum* strain SSMR1 chromosome, complete genome | *Fusobacterium nucleatum* subsp. *polymorphum* | 2030 | 10119 | 100% | 0 | 91.56 | 2443126 | CP100429.1 |
| Uncultured *Fusobacterium sp.* partial 16S rRNA gene, clone 703004(oral) | Uncultured *Fusobacterium sp.* | 2028 | 2028 | 99% | 0 | 91.56 | 1469 | AM420243.1 |
| *Fusobacterium nucleatum* subsp. *polymorphum* strain ChDC F306, complete genome | *Fusobacterium nucleatum* subsp. *polymorphum* | 2030 | 8932 | 100% | 0 | 91.55 | 2600840 | CP013121.1 |
| Uncultured *Fusobacterium sp.* partial 16S rRNA gene, clone 301F01(oral) | Uncultured *Fusobacterium sp.* | 2039 | 2039 | 99% | 0 | 91.53 | 1484 | AM420071.1 |
| *Fusobacterium nucleatum* subsp. *vincentii* strain KCOM 2931 chromosome, complete genome | *Fusobacterium nucleatum* subsp. *vincentii* | 2037 | 10106 | 100% | 0 | 91.52 | 2087706 | CP024749.1 |
| *Fusobacterium sp.* oral clone CY024 16S ribosomal RNA gene, partial sequence | *Fusobacterium sp.* oral clone CY024 | 2030 | 2030 | 99% | 0 | 91.47 | 1512 | AF287809.1 |
| *Fusobacterium nucleatum* subsp. *vincentii* ChDC F8 strain KCOM 1231 chromosome, complete genome | *Fusobacterium nucleatum* subsp. *vincentii* ChDC F8 | 2032 | 8823 | 100% | 0 | 91.46 | 2038535 | CP012714.1 |
| *Fusobacterium nucleatum* strain FNV chromosome, complete genome | *Fusobacterium nucleatum* | 2032 | 10101 | 100% | 0 | 91.45 | 2194806 | CP117525.1 |
| *Fusobacterium nucleatum* subsp. *nucleatum* ATCC 23726 chromosome, complete genome | *Fusobacterium nucleatum* subsp. *nucleatum* ATCC 23726 | 2026 | 10101 | 100% | 0 | 91.39 | 2299539 | CP028109.1 |

Table shows BLASTn top 100 results for the *F. sphaericum sp. nov.* SB021 16S rRNA gene by decreasing percent identity.

**Supplementary Table 2: RAST annotation of the *F. sphaericum sp. nov.* SB021 genome**

| **Contig** | **Locus_tag** | **Feature Type** | **Length (bp)** | **Gene** | **EC number** | **COG** | **Product** |
| --- | --- | --- | --- | --- | --- | --- | --- |
| SB021 contig 1 | LEJJMMNP_00001 | rRNA | 1511 |  |  |  | 16S ribosomal RNA |
| SB021 contig 1 | LEJJMMNP_00002 | rRNA | 2903 |  |  |  | 23S ribosomal RNA |
| SB021 contig 1 | LEJJMMNP_00003 | rRNA | 95 |  |  |  | 5S ribosomal RNA |
| SB021 contig 1 | LEJJMMNP_00004 | CDS | 588 |  |  |  | Hypothetical protein |
| SB021 contig 1 | LEJJMMNP_00005 | CDS | 1200 | *pgk* | 2.7.2.3 |  | Phosphoglycerate kinase |
| SB021 contig 1 | LEJJMMNP_00006 | CDS | 1008 | *gap* | 1.2.1.12 |  | Glyceraldehyde-3-phosphate dehydrogenase |
| SB021 contig 1 | LEJJMMNP_00007 | CDS | 870 | *rluD_1* | 5.4.99.23 | COG0564 | Ribosomal large subunit pseudouridine synthetase D |
| SB021 contig 1 | LEJJMMNP_00008 | CDS | 381 | *yabJ* | 3.5.99.10 | COG0251 | 2-iminobutanoate/2-iminopropanoate deaminase |
| SB021 contig 1 | LEJJMMNP_00009 | CDS | 900 |  |  |  | Hypothetical protein |
| SB021 contig 1 | LEJJMMNP_00010 | CDS | 1767 | *nor_1* |  |  | Anaerobic nitric oxide reductase transcription regulator NorR |
| SB021 contig 1 | LEJJMMNP_00011 | CDS | 1248 | *rpoN* |  | COG1508 | RNA polymerase sigma-54 factor |
| SB021 contig 1 | LEJJMMNP_00012 | CDS | 1530 |  |  |  | Hypothetical protein |
| SB021 contig 1 | LEJJMMNP_00013 | CDS | 2340 |  |  |  | Hypothetical protein |
| SB021 contig 1 | LEJJMMNP_00014 | CDS | 1296 |  |  |  | Nucleobase transporter PlUacP |
| SB021 contig 1 | LEJJMMNP_00015 | CDS | 783 |  |  |  | Hypothetical protein |
| SB021 contig 1 | LEJJMMNP_00016 | CDS | 471 | *hcrC* | 1.3.7.9 |  | 4-hydroxybenzoyl-CoA reductase subunit gamma |
| SB021 contig 1 | LEJJMMNP_00017 | CDS | 2259 | *xdhA* | 1.17.1.4 | COG1529 | Putative xanthine dehydrogenase molybdenum-binding subunit XdhA |
| SB021 contig 1 | LEJJMMNP_00018 | CDS | 1443 | *sdcS* |  |  | Sodium-dependent dicarboxylate transporter SdcS |
| SB021 contig 1 | LEJJMMNP_00019 | CDS | 411 |  |  |  | Hypothetical protein |
| SB021 contig 1 | LEJJMMNP_00020 | CDS | 1911 | *ettA* |  | COG0488 | Energy-dependent translational throttle protein EttA |
| SB021 contig 1 | LEJJMMNP_00021 | CDS | 369 | *rpSL* |  |  | 30S ribosomal protein S12 |
| SB021 contig 1 | LEJJMMNP_00022 | CDS | 471 | *rpsG* |  |  | 30S ribosomal protein S7 |
| SB021 contig 1 | LEJJMMNP_00023 | CDS | 2076 | *fusA_1* |  | COG0480 | Elongation factor G |
| SB021 contig 1 | LEJJMMNP_00024 | CDS | 1185 | *tufA_1* |  | COG0050 | Elongation factor Tu |
| SB021 contig 1 | LEJJMMNP_00025 | CDS | 1479 |  |  |  | IS1182 family transposase ISFnu2 |
| SB021 contig 1 | LEJJMMNP_00026 | CDS | 270 |  |  |  | Hypothetical protein |
| SB021 contig 1 | LEJJMMNP_00027 | CDS | 1359 |  |  |  | Hypothetical protein |
| SB021 contig 1 | LEJJMMNP_00028 | CDS | 318 |  |  |  | Hypothetical protein |
| SB021 contig 1 | LEJJMMNP_00029 | CDS | 816 | *hisK_1* | 3.1.3.15 | COG1387 | Histidinol-phosphatase |
| SB021 contig 1 | LEJJMMNP_00030 | CDS | 1014 | *tsaD* | 2.3.1.234 | COG0533 | tRNA N6-adenosine threonylcarbamoyltransferase |
| SB021 contig 1 | LEJJMMNP_00031 | CDS | 1326 |  |  |  | Hypothetical protein |
| SB021 contig 1 | LEJJMMNP_00032 | CDS | 324 |  |  |  | Hypothetical protein |
| SB021 contig 1 | LEJJMMNP_00033 | tRNA | 88 |  |  |  | tRNA-Leu(taa) |
| SB021 contig 1 | LEJJMMNP_00034 | tRNA | 76 |  |  |  | tRNA-Met(cat) |
| SB021 contig 1 | LEJJMMNP_00035 | tRNA | 76 |  |  |  | tRNA-Gly(tcc) |
| SB021 contig 1 | LEJJMMNP_00036 | tRNA | 76 |  |  |  | tRNA-Lys(ttt) |
| SB021 contig 1 | LEJJMMNP_00037 | tRNA | 77 |  |  |  | tRNA-Arg(tct) |
| SB021 contig 1 | LEJJMMNP_00038 | tRNA | 77 |  |  |  | tRNA-Met(cat) |
| SB021 contig 1 | LEJJMMNP_00039 | tRNA | 75 |  |  |  | tRNA-Glu(ttc) |
| SB021 contig 1 | LEJJMMNP_00040 | tRNA | 84 |  |  |  | tRNA-Ser(tga) |
| SB021 contig 1 | LEJJMMNP_00041 | tRNA | 76 |  |  |  | tRNA-Phe(gaa) |
| SB021 contig 1 | LEJJMMNP_00042 | tRNA | 76 |  |  |  | tRNA-Val(tac) |
| SB021 contig 1 | LEJJMMNP_00043 | tRNA | 77 |  |  |  | tRNA-Asp(gtc) |
| SB021 contig 1 | LEJJMMNP_00044 | CDS | 423 | *yiaC* | 2.3.1.- | COG0454 | Peptidyl-lysine N-acetyltransferase YiaC |
| SB021 contig 1 | LEJJMMNP_00045 | CDS | 1281 |  |  |  | Hypothetical protein |
| SB021 contig 1 | LEJJMMNP_00046 | CDS | 1356 |  |  |  | Hypothetical protein |
| SB021 contig 1 | LEJJMMNP_00047 | CDS | 363 |  |  |  | Hypothetical protein |
| SB021 contig 1 | LEJJMMNP_00048 | CDS | 1251 | *dctA_1* |  | COG1301 | C4-dicarboxylate transport protein |
| SB021 contig 1 | LEJJMMNP_00049 | CDS | 1140 |  |  |  | IS200/IS605 family transposase ISEfa4 |
| SB021 contig 1 | LEJJMMNP_00050 | CDS | 834 |  |  |  | Hypothetical protein |
| SB021 contig 1 | LEJJMMNP_00051 | CDS | 1080 |  |  |  | Hypothetical protein |
| SB021 contig 1 | LEJJMMNP_00052 | CDS | 783 |  |  |  | Hypothetical protein |
| SB021 contig 1 | LEJJMMNP_00053 | CDS | 1014 |  |  |  | Hypothetical protein |
| SB021 contig 1 | LEJJMMNP_00054 | CDS | 1020 | *rfaF* | 2.-.-.- | COG0859 | ADP-heptose—LPS heptosyltransferase 2 |
| SB021 contig 1 | LEJJMMNP_00055 | CDS | 693 |  |  |  | Hypothetical protein |
| SB021 contig 1 | LEJJMMNP_00056 | CDS | 1005 |  |  |  | Hypothetical protein |
| SB021 contig 1 | LEJJMMNP_00057 | CDS | 1080 | *recA* |  | COG0468 | Protein RecA |
| SB021 contig 1 | LEJJMMNP_00058 | CDS | 549 | *recX* |  |  | Regulatory protein RecX |
| SB021 contig 1 | LEJJMMNP_00059 | CDS | 732 |  |  |  | Hypothetical protein |
| SB021 contig 1 | LEJJMMNP_00060 | CDS | 129 | *truA_1* | 5.4.99.12 |  | tRNA pseudouridine synthase A |
| SB021 contig 1 | LEJJMMNP_00061 | CDS | 546 | *truA_2* | 5.4.99.12 | COG0101 | tRNA pseudouridine synthase A |
| SB021 contig 1 | LEJJMMNP_00062 | CDS | 447 |  |  |  | Hypothetical protein |
| SB021 contig 1 | LEJJMMNP_00063 | CDS | 429 | *per* |  | COG0735 | Peroxide operon regulator |
| SB021 contig 1 | LEJJMMNP_00064 | CDS | 390 | *dfx* | 1.15.1.2 | COG2033 | Desulfoferrodoxin |
| SB021 contig 1 | LEJJMMNP_00065 | CDS | 768 |  |  |  | Hypothetical protein |
| SB021 contig 1 | LEJJMMNP_00066 | CDS | 825 |  |  |  | Hypothetical protein |
| SB021 contig 1 | LEJJMMNP_00067 | CDS | 261 |  |  |  | Hypothetical protein |
| SB021 contig 1 | LEJJMMNP_00068 | CDS | 444 |  |  |  | Hypothetical protein |
| SB021 contig 1 | LEJJMMNP_00069 | CDS | 3579 |  |  |  | Hypothetical protein |
| SB021 contig 1 | LEJJMMNP_00070 | CDS | 1479 |  |  |  | IS1182 family transposase ISFnu2 |
| SB021 contig 1 | LEJJMMNP_00071 | CDS | 198 | *cspL* |  | COG1278 | Cold shock protein 2 |
| SB021 contig 1 | LEJJMMNP_00072 | CDS | 1224 | *macB* | 3.6.3.- |  | Macrolide export ATP-binding/permease protein MacB |
| SB021 contig 1 | LEJJMMNP_00073 | CDS | 678 | *yknY* | 3.6.3.- | COG1136 | Putative ABC transporter ATP-binding protein YknY |
| SB021 contig 1 | LEJJMMNP_00074 | CDS | 1095 | *yknX* |  | COG0845 | Putative efflux system component YknX |
| SB021 contig 1 | LEJJMMNP_00075 | CDS | 1248 |  |  |  | Hypothetical protein |
| SB021 contig 1 | LEJJMMNP_00076 | CDS | 1206 | *thlA* | 2.3.1.9 | COG0183 | Acetyl-CoA acetyltransferase |
| SB021 contig 1 | LEJJMMNP_00077 | CDS | 1146 | *bcd_1* | 1.3.8.1 | COG1960 | Acyl-CoA dehydrogenase, short-chain specific |
| SB021 contig 1 | LEJJMMNP_00078 | CDS | 786 | *carD* | 1.3.1.108 | COG2086 | Caffeyl-CoA reductase-Etf complex subunit CarD |
| SB021 contig 1 | LEJJMMNP_00079 | CDS | 1011 | *carE* | 1.3.1.108 | COG2025 | Caffeyl-CoA reductase-Etf complex subunit CarE |
| SB021 contig 1 | LEJJMMNP_00080 | CDS | 1542 | *xylB* | 2.7.1.17 | COG1070 | Xylulose kinase |
| SB021 contig 1 | LEJJMMNP_00081 | CDS | 687 | *yfnB* | 3.-.-.- | COG1011 | Putative HAD-hydrolase YfnB |
| SB021 contig 1 | LEJJMMNP_00082 | CDS | 645 | *yiaD* |  | COG2885 | Putative lipoprotein YiaD |
| SB021 contig 1 | LEJJMMNP_00083 | tRNA | 77 |  |  |  | tRNA-Pro(tgg) |
| SB021 contig 1 | LEJJMMNP_00084 | tRNA | 76 |  |  |  | tRNA-Gly(tcc) |
| SB021 contig 1 | LEJJMMNP_00085 | tRNA | 77 |  |  |  | tRNA-Arg(tct) |
| SB021 contig 1 | LEJJMMNP_00086 | tRNA | 76 |  |  |  | tRNA-His(gtg) |
| SB021 contig 1 | LEJJMMNP_00087 | tRNA | 76 |  |  |  | tRNA-Lys(ttt) |
| SB021 contig 1 | LEJJMMNP_00088 | tRNA | 84 |  |  |  | tRNA-Leu(tag) |
| SB021 contig 1 | LEJJMMNP_00089 | CDS | 1053 |  |  |  | Hypothetical protein |
| SB021 contig 1 | LEJJMMNP_00090 | CDS | 561 |  |  |  | Hypothetical protein |
| SB021 contig 1 | LEJJMMNP_00091 | CDS | 1281 | *murAA* | 2.5.1.7 | COG0766 | UDP-N-acetylglucosamine 1-carboxyvinyltransferase 1 |
| SB021 contig 1 | LEJJMMNP_00092 | CDS | 711 |  | 2.1.1.- |  | Putative TrmH family tRNA/rRNA methyltransferase |
| SB021 contig 1 | LEJJMMNP_00093 | CDS | 573 | *sigH* |  | COG1595 | RNA polymerase sigma-H factor |
| SB021 contig 1 | LEJJMMNP_00094 | CDS | 2610 | *leuS* | 6.1.1.4 | COG0495 | Leucine—tRNA ligase |
| SB021 contig 1 | LEJJMMNP_00095 | CDS | 642 | *adk* | 2.7.4.3 |  | Adenylate kinase |
| SB021 contig 1 | LEJJMMNP_00096 | CDS | 771 | *map* | 3.4.11.18 | COG0024 | Methionine aminopeptidase 1 |
| SB021 contig 1 | LEJJMMNP_00097 | CDS | 219 | *infA* |  |  | Translation initiation factor IF-1 |
| SB021 contig 1 | LEJJMMNP_00098 | CDS | 357 | *rpsM* |  | COG0099 | 30S ribosomal protein S13 |
| SB021 contig 1 | LEJJMMNP_00099 | CDS | 390 | *rpsK* |  |  | 30S ribosomal protein S11 |
| SB021 contig 1 | LEJJMMNP_00100 | CDS | 588 | *rpsD* |  | COG0522 | 30S ribosomal protein S4 |
| SB021 contig 1 | LEJJMMNP_00101 | CDS | 978 | *rpoA* | 2.7.7.6 | COG0202 | DNA-directed RNA polymerase subunit alpha |
| SB021 contig 1 | LEJJMMNP_00102 | CDS | 351 | *rplQ* |  | COG0203 | 50S ribosomal protein L17 |
| SB021 contig 1 | LEJJMMNP_00103 | CDS | 1581 |  |  |  | Hypothetical protein |
| SB021 contig 1 | LEJJMMNP_00104 | CDS | 561 |  |  |  | Hypothetical protein |
| SB021 contig 1 | LEJJMMNP_00105 | CDS | 777 | *rsxB* | 7.-.-.- |  | Ion-translocating oxidoreductase complex subunit B |
| SB021 contig 1 | LEJJMMNP_00106 | CDS | 936 | *ldh* | 1.1.1.27 |  | L-lactate dehydrogenase |
| SB021 contig 1 | LEJJMMNP_00107 | CDS | 1200 | *mnmE_1* | 3.6.-.- |  | tRNA modification GTPase MnmE |
| SB021 contig 1 | LEJJMMNP_00108 | CDS | 618 | *pncA* | 3.5.1.19 | COG1335 | Nicotinamidase |
| SB021 contig 1 | LEJJMMNP_00109 | CDS | 756 | *rpsB* |  | COG0052 | 30S ribosomal protein S2 |
| SB021 contig 1 | LEJJMMNP_00110 | CDS | 888 | *tsf* |  | COG0264 | Elongation factor Ts |
| SB021 contig 1 | LEJJMMNP_00111 | CDS | 717 | *pyrH* | 2.7.4.22 | COG0528 | Uridylate kinase |
| SB021 contig 1 | LEJJMMNP_00112 | CDS | 561 | *frr* |  | COG0233 | Ribosome-recycling factor |
| SB021 contig 1 | LEJJMMNP_00113 | CDS | 201 | *cspLA* |  | COG1278 | Cold shock-like protein CspLA |
| SB021 contig 1 | LEJJMMNP_00114 | CDS | 1413 | *pykF* | 2.7.1.40 | COG0469 | Pyruvate kinase I |
| SB021 contig 1 | LEJJMMNP_00115 | CDS | 1308 | *eno* | 4.2.1.11 | COG0148 | Enolase |
| SB021 contig 1 | LEJJMMNP_00116 | CDS | 702 | *uppS* | 2.5.1.- | COG0020 | Isoprenyl transferase |
| SB021 contig 1 | LEJJMMNP_00117 | CDS | 816 |  |  |  | Hypothetical protein |
| SB021 contig 1 | LEJJMMNP_00118 | CDS | 1152 | *dxr* | 1.1.1.267 | COG0743 | 1-deoxy-D-xylulose 5-phosphate reductoisomerase |
| SB021 contig 1 | LEJJMMNP_00119 | CDS | 681 | *tmk* | 2.7.4.9 | COG0125 | Thymidylate kinase |
| SB021 contig 1 | LEJJMMNP_00120 | CDS | 1017 | *rasP* | 3.4.24.- | COG0750 | Regulator of sigma-W protease RasP |
| SB021 contig 1 | LEJJMMNP_00121 | CDS | 1386 | *zraR* |  | COG2204 | Transcriptional regulatory protein ZraR |
| SB021 contig 1 | LEJJMMNP_00122 | CDS | 1713 | *surA* | 5.2.1.8 |  | Chaperone SurA |
| SB021 contig 1 | LEJJMMNP_00123 | CDS | 1812 | *dnaG* | 2.7.7.- |  | DNA primase |
| SB021 contig 1 | LEJJMMNP_00124 | CDS | 1215 | *sigA_1* |  | COG0568 | RNA polymerase sigma factor SigA |
| SB021 contig 1 | LEJJMMNP_00125 | CDS | 780 |  |  |  | Hypothetical protein |
| SB021 contig 1 | LEJJMMNP_00126 | CDS | 999 |  |  | COG0327 | GTP cyclohydrolase 1 type 2 |
| SB021 contig 1 | LEJJMMNP_00127 | CDS | 498 |  |  |  | Hypothetical protein |
| SB021 contig 1 | LEJJMMNP_00128 | rRNA | 1511 |  |  |  | 16S ribosomal RNA |
| SB021 contig 1 | LEJJMMNP_00129 | rRNA | 2903 |  |  |  | 23S ribosomal RNA |
| SB021 contig 1 | LEJJMMNP_00130 | rRNA | 95 |  |  |  | 5S ribosomal RNA |
| SB021 contig 1 | LEJJMMNP_00131 | CDS | 939 |  |  |  | Hypothetical protein |
| SB021 contig 1 | LEJJMMNP_00132 | CDS | 1152 |  |  |  | Hypothetical protein |
| SB021 contig 1 | LEJJMMNP_00133 | CDS | 153 | *rpmGA* |  |  | 50S ribosomal protein L33 |
| SB021 contig 1 | LEJJMMNP_00134 | tRNA | 76 |  |  |  | tRNA-Trp(cca) |
| SB021 contig 1 | LEJJMMNP_00135 | CDS | 186 |  |  |  | Hypothetical protein |
| SB021 contig 1 | LEJJMMNP_00136 | CDS | 600 | *nusG* |  | COG0250 | Transcription termination/antitermination protein NusG |
| SB021 contig 1 | LEJJMMNP_00137 | CDS | 426 | *rplK* |  | COG0080 | 50S ribosomal protein L11 |
| SB021 contig 1 | LEJJMMNP_00138 | CDS | 711 | *rplA* |  |  | 50S ribosomal protein L1 |
| SB021 contig 1 | LEJJMMNP_00139 | CDS | 507 | *rplJ* |  |  | 50S ribosomal protein L10 |
| SB021 contig 1 | LEJJMMNP_00140 | CDS | 366 | *rplL* |  | COG0222 | 50S ribosomal protein L7/L12 |
| SB021 contig 1 | LEJJMMNP_00141 | CDS | 3513 | *rpoB* | 2.7.7.6 | COG0085 | DNA-directed RNA polymerase subunit beta |
| SB021 contig 1 | LEJJMMNP_00142 | CDS | 3951 | *rpoC* | 2.7.7.6 | COG0086 | DNA-directed RNA polymerase subunit beta’ |
| SB021 contig 1 | LEJJMMNP_00143 | CDS | 873 |  |  |  | Hypothetical protein |
| SB021 contig 1 | LEJJMMNP_00144 | CDS | 564 | *gmk* | 2.7.4.8 | COG0194 | Guanylate kinase |
| SB021 contig 1 | LEJJMMNP_00145 | CDS | 216 | *rpoZ* | 2.7.7.6 |  | DNA-directed RNA polymerase subunit omega |
| SB021 contig 1 | LEJJMMNP_00146 | CDS | 981 | *apbE* | 2.7.1.180 | COG1477 | FAD:protein FMN transferase |
| SB021 contig 1 | LEJJMMNP_00147 | CDS | 2010 | *hppA1* | 7.2.3.- | COG3808 | Putative K(+)-stimulated pyrophosphate-energized sodium pump |
| SB021 contig 1 | LEJJMMNP_00148 | CDS | 828 |  |  |  | Hypothetical protein |
| SB021 contig 1 | LEJJMMNP_00149 | CDS | 591 |  | 3.2.2.- | COG2094 | Putative 3-methyladenine DNA glycosylase |
| SB021 contig 1 | LEJJMMNP_00150 | CDS | 843 |  |  |  | Hypothetical protein |
| SB021 contig 1 | LEJJMMNP_00151 | CDS | 4953 | *yfhM* |  | COG2373 | Alpha-2-macroglobulin |
| SB021 contig 1 | LEJJMMNP_00152 | CDS | 1677 | *pbpC* |  | COG4953 | Penicillin-binding protein 1C |
| SB021 contig 1 | LEJJMMNP_00153 | CDS | 549 |  |  |  | Hypothetical protein |
| SB021 contig 1 | LEJJMMNP_00154 | CDS | 681 | *copR* |  |  | Transcriptional activator protein CopR |
| SB021 contig 1 | LEJJMMNP_00155 | CDS | 1347 | *sasA_1* | 2.7.-.- |  | Adaptive-response sensory-kinase SasA |
| SB021 contig 1 | LEJJMMNP_00156 | CDS | 390 |  |  |  | Hypothetical protein |
| SB021 contig 1 | LEJJMMNP_00157 | CDS | 207 | *rpmE* |  | COG0254 | 50S ribosomal protein L31 |
| SB021 contig 1 | LEJJMMNP_00158 | CDS | 627 | *upp* | 2.4.2.9 |  | Uracil phosphoribosyltransferase |
| SB021 contig 1 | LEJJMMNP_00159 | tRNA | 84 |  |  |  | tRNA-Ser(tga) |
| SB021 contig 1 | LEJJMMNP_00160 | tRNA | 93 |  |  |  | tRNA-Ser(gct) |
| SB021 contig 1 | LEJJMMNP_00161 | tRNA | 77 |  |  |  | tRNA-Arg(acg) |
| SB021 contig 1 | LEJJMMNP_00162 | CDS | 474 |  |  |  | Hypothetical protein |
| SB021 contig 1 | LEJJMMNP_00163 | CDS | 1974 |  |  |  | Hypothetical protein |
| SB021 contig 1 | LEJJMMNP_00164 | CDS | 945 |  |  |  | Hypothetical protein |
| SB021 contig 1 | LEJJMMNP_00165 | CDS | 768 |  |  |  | Hypothetical protein |
| SB021 contig 1 | LEJJMMNP_00166 | CDS | 513 | *infC* |  | COG0290 | Translation initiation factor IF-3 |
| SB021 contig 1 | LEJJMMNP_00167 | CDS | 207 |  |  |  | Hypothetical protein |
| SB021 contig 1 | LEJJMMNP_00168 | CDS | 351 | *rplT* |  | COG0292 | 50S ribosomal protein L20 |
| SB021 contig 1 | LEJJMMNP_00169 | CDS | 180 |  |  |  | Hypothetical protein |
| SB021 contig 1 | LEJJMMNP_00170 | CDS | 723 |  |  |  | Hypothetical protein |
| SB021 contig 1 | LEJJMMNP_00171 | CDS | 1725 |  |  |  | Hypothetical protein |
| SB021 contig 1 | LEJJMMNP_00172 | CDS | 1245 | *hisC_1* | 2.6.1.9 |  | Histidinol-phosphate aminotransferase |
| SB021 contig 1 | LEJJMMNP_00173 | CDS | 252 |  |  |  | Hypothetical protein |
| SB021 contig 1 | LEJJMMNP_00174 | CDS | 1530 |  |  |  | Hypothetical protein |
| SB021 contig 1 | LEJJMMNP_00175 | CDS | 942 | *rluD_2* | 5.4.99.23 | COG0564 | Ribosomal large subunit pseudouridine synthase D |
| SB021 contig 1 | LEJJMMNP_00176 | CDS | 618 | *rnhB* | 3.1.26.4 | COG0164 | Ribonuclease Hll |
| SB021 contig 1 | LEJJMMNP_00177 | CDS | 369 |  |  |  | Hypothetical protein |
| SB021 contig 1 | LEJJMMNP_00178 | CDS | 648 |  |  |  | Hypothetical protein |
| SB021 contig 1 | LEJJMMNP_00179 | CDS | 756 | *tpiA* | 5.3.1.1 |  | Triosephosphate isomerase |
| SB021 contig 1 | LEJJMMNP_00180 | CDS | 1524 | *gpml* | 5.4.2.12 | COG0696 | 2,3-bisphosphoglycerate-independent phosphoglycerate mutase |
| SB021 contig 1 | LEJJMMNP_00181 | CDS | 375 |  |  |  | Hypothetical protein |
| SB021 contig 1 | LEJJMMNP_00182 | CDS | 1329 | *uxaA* | 4.2.1.7 | COG2721 | Altronate dehydratase |
| SB021 contig 1 | LEJJMMNP_00183 | CDS | 1446 | *uxaB* | 1.1.1.58 | COG0246 | Altronate oxidoreductase |
| SB021 contig 1 | LEJJMMNP_00184 | CDS | 1017 | *lgoD* | 1.1.1.414 | COG1063 | L-galactonate-5-dehydrogenase |
| SB021 contig 1 | LEJJMMNP_00185 | CDS | 627 | *rspR* |  | COG1802 | HTH-type transcriptional repressor RspR |
| SB021 contig 1 | LEJJMMNP_00186 | CDS | 1041 |  |  |  | Hypothetical protein |
| SB021 contig 1 | LEJJMMNP_00187 | CDS | 474 |  |  |  | Hypothetical protein |
| SB021 contig 1 | LEJJMMNP_00188 | CDS | 1281 | *siaM* |  | COG1593 | Sialic acid TRAP transporter large permease protein SiaM |
| SB021 contig 1 | LEJJMMNP_00189 | CDS | 1839 |  |  |  | Hypothetical protein |
| SB021 contig 1 | LEJJMMNP_00190 | CDS | 552 | *lemA* |  | COG1704 | Protein LemA |
| SB021 contig 1 | LEJJMMNP_00191 | CDS | 753 | *xynR* |  | COG1414 | HTH-type transcriptional regulator XynR |
| SB021 contig 1 | LEJJMMNP_00192 | CDS | 2832 | *srmB* | 3.6.4.13 |  | ATP-dependent RNA helicase SrmB |
| SB021 contig 1 | LEJJMMNP_00193 | CDS | 1332 |  | 2.8.3.- | COG0427 | Butyryl-CoA:acetate CoA-transferase |
| SB021 contig 1 | LEJJMMNP_00194 | CDS | 1242 |  |  |  | Hypothetical protein |
| SB021 contig 1 | LEJJMMNP_00195 | CDS | 399 | *manX* | 2.7.1.191 | COG2893 | PTS system mannose-specific EIIAB component |
| SB021 contig 1 | LEJJMMNP_00196 | CDS | 486 | *sorB* | 2.7.1.206 | COG3444 | PTS system sorbose-specific EIIB component |
| SB021 contig 1 | LEJJMMNP_00197 | CDS | 750 | *agaC_1* |  | COG3715 | N-acetylgalactosamine permease IIC component 1 |
| SB021 contig 1 | LEJJMMNP_00198 | CDS | 843 | *manZ_1* |  | COG3716 | PTS system mannose-specific EIID component |
| SB021 contig 1 | LEJJMMNP_00199 | CDS | 1104 | *dgaE* | 4.3.1.29 |  | D-glucosaminate-6-phosphate ammonia lyase |
| SB021 contig 1 | LEJJMMNP_00200 | CDS | 750 | *dgaF* | 4.1.2.14 |  | 2-dehydro-3-deoxy-phosphogluconate aldolase |
| SB021 contig 1 | LEJJMMNP_00201 | CDS | 654 |  | 3.1.3.- | COG0637 | Phosphorylated carbohydrates phosphatase |
| SB021 contig 1 | LEJJMMNP_00202 | CDS | 1446 | *trkH* |  | COG0168 | Trk system potassium uptake protein TrkH |
| SB021 contig 1 | LEJJMMNP_00203 | CDS | 1386 | *trkA* |  | COG0569 | Trk system potassium uptake protein TrkA |
| SB021 contig 1 | LEJJMMNP_00204 | CDS | 1194 | *mgl* | 4.4.1.11 | COG0626 | L-methionine gamma-lyase |
| SB021 contig 1 | LEJJMMNP_00205 | CDS | 819 |  |  |  | Hypothetical protein |
| SB021 contig 1 | LEJJMMNP_00206 | CDS | 795 | *metQ* |  | COG1464 | D-methionine-binding lipoprotein MetQ |
| SB021 contig 1 | LEJJMMNP_00207 | CDS | 642 | *metP* |  | COG2011 | Methionine import system permease protein MetP |
| SB021 contig 1 | LEJJMMNP_00208 | CDS | 1011 | *metN* | 3.6.3.- | COG1135 | Methionine import ATP-binding protein MetN |
| SB021 contig 1 | LEJJMMNP_00209 | CDS | 492 | *tadA_1* | 3.5.4.33 |  | tRNA-specific adenosine deaminase |
| SB021 contig 1 | LEJJMMNP_00210 | CDS | 348 |  |  |  | Hypothetical protein |
| SB021 contig 1 | LEJJMMNP_00211 | rRNA | 1511 |  |  |  | 16S ribosomal RNA |
| SB021 contig 1 | LEJJMMNP_00212 | rRNA | 2903 |  |  |  | 23S ribosomal RNA |
| SB021 contig 1 | LEJJMMNP_00213 | rRNA | 95 |  |  |  | 5S ribosomal RNA |
| SB021 contig 1 | LEJJMMNP_00214 | CDS | 867 |  |  |  | Hypothetical protein |
| SB021 contig 1 | LEJJMMNP_00215 | CDS | 573 | *gmhB* | 3.1.3.83 |  | D-glycero-alpha-D-manno-heptose-1,7-bisphosphate 7-phosphatase |
| SB021 contig 1 | LEJJMMNP_00216 | CDS | 1281 | *murG* | 6.3.2.10 |  | UDP-N-acetylmuramoyl-tripeptide--D-alanyl-D-alanine ligase |
| SB021 contig 1 | LEJJMMNP_00217 | CDS | 1089 | *mraY* | 2.7.8.13 | COG0472 | Phospho-N-acetylmuramoyl-pentapeptide-transferase |
| SB021 contig 1 | LEJJMMNP_00218 | CDS | 1323 | *murD* | 6.3.2.9 |  | UDP-N-acetylmuramoylalanine--D-glutamate ligase |
| SB021 contig 1 | LEJJMMNP_00219 | CDS | 1068 | *murG* | 2.4.1.227 | COG0707 | UDP-N-acetylglucosamine--N-acetylmuramyl-(pentapeptide) pyrophosphoryl-undecaprenol N-acetylglucosamine transferase |
| SB021 contig 1 | LEJJMMNP_00220 | CDS | 1335 | *murC* | 6.3.2.8 |  | UDP-N-acetylmuramate--L-alanine ligase |
| SB021 contig 1 | LEJJMMNP_00221 | CDS | 840 | *murB* | 1.3.1.98 | COG0812 | UDP-N-acetylenolpyruvoylglucosamine reductase |
| SB021 contig 1 | LEJJMMNP_00222 | CDS | 870 | *ddl* | 6.3.2.4 | COG1181 | D-alanine--D-alanine ligase |
| SB021 contig 1 | LEJJMMNP_00223 | CDS | 693 | *ftsQ* |  |  | Cell division protein FtsQ |
| SB021 contig 1 | LEJJMMNP_00224 | CDS | 1272 | *ftsA* |  | COG0849 | Cell division protein FtsA |
| SB021 contig 1 | LEJJMMNP_00225 | CDS | 1071 | *ftsZ* |  | COG0206 | Cell division protein FtsZ |
| SB021 contig 1 | LEJJMMNP_00226 | CDS | 285 | *rpsF* |  | COG0360 | 30S ribosomal protein S6 |
| SB021 contig 1 | LEJJMMNP_00227 | CDS | 219 |  |  |  | Hypothetical protein |
| SB021 contig 1 | LEJJMMNP_00228 | CDS | 237 |  |  |  | Hypothetical protein |
| SB021 contig 1 | LEJJMMNP_00229 | CDS | 1308 |  |  |  | Hypothetical protein |
| SB021 contig 1 | LEJJMMNP_00230 | CDS | 1185 | *tufA_2* |  | COG0050 | Elongation factor Tu |
| SB021 contig 1 | LEJJMMNP_00231 | tRNA | 85 |  |  |  | tRNA-Tyr(gta) |
| SB021 contig 1 | LEJJMMNP_00232 | tRNA | 75 |  |  |  | tRNA-Glu(ttc) |
| SB021 contig 1 | LEJJMMNP_00233 | tRNA | 76 |  |  |  | tRNA-Thr(tgt) |
| SB021 contig 1 | LEJJMMNP_00234 | CDS | 831 | *moaA_1* |  |  | GTP 3’,8-cyclase |
| SB021 contig 1 | LEJJMMNP_00235 | CDS | 1332 |  |  | COG2239 | Magnesium transporter MgtE |
| SB021 contig 1 | LEJJMMNP_00236 | CDS | 1479 |  |  |  | IS1182 family transposase ISFnu2 |
| SB021 contig 1 | LEJJMMNP_00237 | CDS | 381 | *ptsG* | 2.7.1.199 | COG1263 | PTS system glucose-specific EIICBA component |
| SB021 contig 1 | LEJJMMNP_00238 | CDS | 912 |  |  |  | Hypothetical protein |
| SB021 contig 1 | LEJJMMNP_00239 | CDS | 1362 | *dtpT* |  | COG3104 | Di-/tripeptide transporter |
| SB021 contig 1 | LEJJMMNP_00240 | CDS | 1401 | *bioB_1* | 2.8.1.6 |  | Biotin synthase |
| SB021 contig 1 | LEJJMMNP_00241 | CDS | 2508 | *mprF* | 2.3.2.3 |  | Phosphatidylglycerol lysyltransferase |
| SB021 contig 1 | LEJJMMNP_00242 | CDS | 2304 | *adiA* | 4.1.1.19 | COG1982 | Biodegradative arginine decarboxylase |
| SB021 contig 1 | LEJJMMNP_00243 | CDS | 1428 | *aaxC* |  | COG0531 | Arginine/agmatine antiporter |
| SB021 contig 1 | LEJJMMNP_00239 | CDS | 1362 | *dtpT* |  | COG3104 | Di-/tripeptide transporter |
| SB021 contig 1 | LEJJMMNP_00240 | CDS | 1401 | *bioB_1* | 2.8.1.6 |  | Biotin synthase |
| SB021 contig 1 | LEJJMMNP_00241 | CDS | 2508 | *mprF* | 2.3.2.3 |  | Phosphatidylglycerol lysyltransferase |
| SB021 contig 1 | LEJJMMNP_00242 | CDS | 2304 | *adiA* | 4.1.1.19 | COG1982 | Biodegradative arginine decarboxylase |
| SB021 contig 1 | LEJJMMNP_00243 | CDS | 1428 | *aaxC* |  | COG0531 | Arginine/agmatine antiporter |
| SB021 contig 1 | LEJJMMNP_00244 | CDS | 2757 | *rapA* | 3.6.4.- |  | RNA polymerase-associated protein RapA |
| SB021 contig 1 | LEJJMMNP_00245 | CDS | 1758 |  |  |  | Hypothetical protein |
| SB021 contig 1 | LEJJMMNP_00246 | CDS | 1011 | *plsX* | 2.3.1.274 | COG0416 | Phosphate acyltransferase |
| SB021 contig 1 | LEJJMMNP_00247 | CDS | 990 | *fabH* | 2.3.1.180 |  | 3-oxoacyl-[acyl-carrier-protein] synthase 3 |
| SB021 contig 1 | LEJJMMNP_00248 | CDS | 912 | *fabD* | 2.3.1.39 |  | Malonyl CoA-acyl carrier protein transacylase |
| SB021 contig 1 | LEJJMMNP_00249 | CDS | 225 | *acpP* |  | COG0236 | Acyl carrier protein |
| SB021 contig 1 | LEJJMMNP_00250 | CDS | 1230 | *fabF* | 2.3.1.179 | COG0304 | 3-oxoacyl-[acyl-carrier-protein] synthase 2 |
| SB021 contig 1 | LEJJMMNP_00251 | CDS | 690 | *rnc* | 3.1.26.3 | COG0571 | Ribonuclease 3 |
| SB021 contig 1 | LEJJMMNP_00252 | CDS | 1038 |  |  |  | Hypothetical protein |
| SB021 contig 1 | LEJJMMNP_00253 | CDS | 1467 | *rng* | 3.1.26.- | COG1530 | Ribonuclease G |
| SB021 contig 1 | LEJJMMNP_00254 | CDS | 498 | *coaD* | 2.7.7.3 | COG0669 | Phosphopantetheine adenylyltransferase |
| SB021 contig 1 | LEJJMMNP_00255 | CDS | 1380 | *radA* | 3.6.4.- | COG1066 | DNA repair protein RadA |
| SB021 contig 1 | LEJJMMNP_00256 | CDS | 1053 | *disA* | 2.7.7.85 |  | DNA integrity scanning protein DisA |
| SB021 contig 1 | LEJJMMNP_00257 | CDS | 2520 | *copA_1* | 7.2.2.8 | COG2217 | Copper-exporting P-type ATPase |
| SB021 contig 1 | LEJJMMNP_00258 | CDS | 267 | *copA_2* | 7.2.2.8 | COG2217 | Copper-exporting P-type ATpase |
| SB021 contig 1 | LEJJMMNP_00259 | CDS | 318 | *csoR* |  | COG1937 | Copper-sensing transcriptional repressor CsoR |
| SB021 contig 1 | LEJJMMNP_00260 | CDS | 801 |  |  |  | Hypothetical protein |
| SB021 contig 1 | LEJJMMNP_00261 | CDS | 435 | *rplM* |  | COG0102 | 50S ribosomal protein L13 |
| SB021 contig 1 | LEJJMMNP_00262 | CDS | 399 | *rpsI* |  | COG0103 | 30S ribosomal protein S9 |
| SB021 contig 1 | LEJJMMNP_00263 | CDS | 168 |  |  |  | Hypothetical protein |
| SB021 contig 1 | LEJJMMNP_00264 | CDS | 606 |  |  |  | Hypothetical protein |
| SB021 contig 1 | LEJJMMNP_00265 | CDS | 591 |  |  |  | Hypothetical protein |
| SB021 contig 1 | LEJJMMNP_00266 | CDS | 1992 |  |  |  | Hypothetical protein |
| SB021 contig 1 | LEJJMMNP_00267 | CDS | 1485 |  | 4.2.2.1 |  | Hyaluronate lyase |
| SB021 contig 1 | LEJJMMNP_00268 | CDS | 504 |  |  |  | Hypothetical protein |
| SB021 contig 1 | LEJJMMNP_00269 | CDS | 783 | *kduD* | 1.1.1.127 |  | 2-dehydro-3-deoxy-D-gluconate 5-dehydrogenase |
| SB021 contig 1 | LEJJMMNP_00270 | CDS | 837 | *kduI* | 5.3.1.17 | COG3717 | 4-deoxy-L-threo-5-hexosulose-uronate ketol-isomerase |
| SB021 contig 1 | LEJJMMNP_00271 | CDS | 1566 |  |  |  | Hypothetical protein |
| SB021 contig 1 | LEJJMMNP_00272 | CDS | 4896 |  |  |  | Hypothetical protein |
| SB021 contig 1 | LEJJMMNP_00273 | CDS | 2421 | *xly* | 4.2.2.12 |  | Xanthan lyase |
| SB021 contig 1 | LEJJMMNP_00274 | CDS | 1410 |  | 3.1.6.- | COG3119 | Endo-4-O-sulfatase |
| SB021 contig 1 | LEJJMMNP_00275 | CDS | 1191 | *ugl* | 3.2.1.180 |  | Unsaturated chondroitin disaccharide hydrolase |
| SB021 contig 1 | LEJJMMNP_00276 | CDS | 1434 | *yjmB* |  | COG2211 | Putative symporter YjmB |
| SB021 contig 1 | LEJJMMNP_00277 | CDS | 639 |  |  |  | Hypothetical protein |
| SB021 contig 1 | LEJJMMNP_00278 | CDS | 576 |  |  |  | Hypothetical protein |
| SB021 contig 1 | LEJJMMNP_00279 | CDS | 2190 |  |  |  | Hypothetical protein |
| SB021 contig 1 | LEJJMMNP_00280 | CDS | 2937 | *chonabc* | 4.2.2.21 |  | Chondroitin sulfate ABC exolyase |
| SB021 contig 1 | LEJJMMNP_00281 | CDS | 468 |  |  |  | Hypothetical protein |
| SB021 contig 1 | LEJJMMNP_00282 | CDS | 1281 | *yeeO* |  | COG0534 | Putative FMN/FAD exporter YeeO |
| SB021 contig 1 | LEJJMMNP_00283 | CDS | 1323 |  |  |  | Hypothetical protein |
| SB021 contig 1 | LEJJMMNP_00284 | CDS | 828 | *rlmA* | 2.1.1.187 | COG0500 | 23S rRNA (guanine(745)-N(1))-methyltransferase |
| SB021 contig 1 | LEJJMMNP_00285 | CDS | 495 |  |  |  | Hypothetical protein |
| SB021 contig 1 | LEJJMMNP_00286 | CDS | 1416 |  |  |  | Hypothetical protein |
| SB021 contig 1 | LEJJMMNP_00287 | CDS | 1125 | *srpA* |  |  | Solvent efflux pump periplasmic linker SrpA |
| SB021 contig 1 | LEJJMMNP_00288 | CDS | 3057 | *mdtB* |  |  | Multidrug resistance protein MdtB |
| SB021 contig 1 | LEJJMMNP_00289 | CDS | 738 |  |  |  | Hypotheticla protein |
| SB021 contig 1 | LEJJMMNP_00290 | CDS | 1401 | *nhaP* |  |  | K(+)/H(+) antiporter NhaP |
| SB021 contig 1 | LEJJMMNP_00291 | CDS | 333 |  |  |  | Hypothetical protein |
| SB021 contig 1 | LEJJMMNP_00292 | CDS | 1320 |  | 3.5.1.47 |  | N-acetyldiaminopimelate deacetylase |
| SB021 contig 1 | LEJJMMNP_00293 | CDS | 465 |  |  |  | Hypothetical protein |
| SB021 contig 1 | LEJJMMNP_00294 | CDS | 819 |  |  |  | Hypothetical protein |
| SB021 contig 1 | LEJJMMNP_00295 | CDS | 1479 |  |  |  | IS1182 family transposase ISFnu2 |
| SB021 contig 1 | LEJJMMNP_00296 | CDS | 1281 |  |  |  | Hypothetical protein |
| SB021 contig 1 | LEJJMMNP_00297 | CDS | 972 | *bioB_2* | 2.8.1.6 | COG0502 | Biotin synthase |
| SB021 contig 1 | LEJJMMNP_00298 | CDS | 1026 |  |  | COG1840 | Putative protein |
| SB021 contig 1 | LEJJMMNP_00299 | CDS | 984 | *cysA* | 7.3.2.3 | COG1118 | Sulfate/thiosulfate import ATP-binding protein CysA |
| SB021 contig 1 | LEJJMMNP_00300 | CDS | 1731 |  |  |  | Hypothetical protein |
| SB021 contig 1 | LEJJMMNP_00301 | CDS | 804 | *phnX* | 3.11.1.1 |  | Phosphonoacetaldehyde hydrolase |
| SB021 contig 1 | LEJJMMNP_00302 | CDS | 1125 | *phnW* | 2.6.1.37 | COG0075 | 2-aminoethylphosphonate--pyruvate transaminase |
| SB021 contig 1 | LEJJMMNP_00303 | CDS | 207 |  |  |  | Hypothetical protein |
| SB021 contig 1 | LEJJMMNP_00304 | CDS | 366 |  |  |  | Hypothetical protein |
| SB021 contig 1 | LEJJMMNP_00305 | CDS | 2613 |  | 7.2.2.10 |  | Calcium-transporting ATPase 1 |
| SB021 contig 1 | LEJJMMNP_00306 | CDS | 1365 |  |  |  | Hypothetical protein |
| SB021 contig 1 | LEJJMMNP_00307 | CDS | 609 |  |  |  | Hypothetical protein |
| SB021 contig 1 | LEJJMMNP_00308 | CDS | 681 |  |  |  | Hypothetical protein |
| SB021 contig 1 | LEJJMMNP_00309 | CDS | 954 |  |  |  | Hypothetical protein |
| SB021 contig 1 | LEJJMMNP_00310 | CDS | 258 | *epsL* | 2.-.-.- | COG2148 | Putative sugar transferase EpsL |
| SB021 contig 1 | LEJJMMNP_00311 | CDS | 1215 |  |  |  | Hypothetical protein |
| SB021 contig 1 | LEJJMMNP_00312 | CDS | 1023 | *capD* | 5.1.3.2 | COG1086 | UDP-glucose 4-epimerase |
| SB021 contig 1 | LEJJMMNP_00313 | CDS | 1173 | *wbjC* | 1.1.1.367 |  | UDP-2-acetamido-2,6-beta-L-arabino-hexul-4-ose reductase |
| SB021 contig 1 | LEJJMMNP_00314 | CDS | 1170 | *wbpI* | 5.1.3.23 | COG0381 | UDP-2,3-diacetamido-2,3-dideoxy-D-glucuronate 2-epimerase |
| SB021 contig 1 | LEJJMMNP_00315 | CDS | 807 |  |  |  | Hypothetical protein |
| SB021 contig 1 | LEJJMMNP_00316 | CDS | 312 |  |  |  | Hypothetical protein |
| SB021 contig 1 | LEJJMMNP_00317 | CDS | 1038 | *gtfA_1* | 2.4.1.- |  | UDP-N-acetylglucosamine--peptide N-acetylglucosaminyltransferase GtfA subunit |
| SB021 contig 1 | LEJJMMNP_00318 | CDS | 1299 |  |  |  | Hypothetical protein |
| SB021 contig 1 | LEJJMMNP_00319 | CDS | 966 |  |  |  | Hypothetical protein |
| SB021 contig 1 | LEJJMMNP_00320 | CDS | 1119 | *mshA* | 2.4.1.250 |  | D-inositol-3-phosphate glycosyltransferase |
| SB021 contig 1 | LEJJMMNP_00321 | CDS | 1089 |  |  |  | Hypothetical protein |
| SB021 contig 1 | LEJJMMNP_00322 | CDS | 1206 |  |  |  | Hypothetical protein |
| SB021 contig 1 | LEJJMMNP_00323 | CDS | 1431 |  |  |  | Hypothetical protein |
| SB021 contig 1 | LEJJMMNP_00324 | CDS | 90 |  |  |  | Hypothetical protein |
| SB021 contig 1 | LEJJMMNP_00325 | CDS | 972 | *mnaA* | 5.1.3.14 | COG0381 | UDP-N-acetylglucosamine 2-epimerase |
| SB021 contig 1 | LEJJMMNP_00326 | CDS | 288 |  |  |  | Hypothetical protein |
| SB021 contig 1 | LEJJMMNP_00327 | CDS | 282 | *ihfA_1* |  |  | Integration host factor subunit alpha |
| SB021 contig 1 | LEJJMMNP_00328 | CDS | 630 | *thiE_1* | 2.5.1.3 |  | Thiamine-phosphate synthase |
| SB021 contig 1 | LEJJMMNP_00329 | CDS | 537 |  |  |  | Hypothetical protein |
| SB021 contig 1 | LEJJMMNP_00330 | CDS | 1107 | *thiH* | 4.1.99.19 | COG1060 | 2-iminoacetate synthase |
| SB021 contig 1 | LEJJMMNP_00331 | CDS | 777 | *thiG* | 2.8.1.10 | COG2022 | Thiazole synthase |
| SB021 contig 1 | LEJJMMNP_00332 | CDS | 213 |  |  |  | Hypothetical protein |
| SB021 contig 1 | LEJJMMNP_00333 | CDS | 678 | *tenA_1* | 3.5.99.2 | COG0819 | Aminopyrimidine aminohydrolase |
| SB021 contig 1 | LEJJMMNP_00334 | CDS | 1347 |  |  |  | Hypothetical protein |
| SB021 contig 1 | LEJJMMNP_00335 | CDS | 567 |  |  |  | Hypothetical protein |
| SB021 contig 1 | LEJJMMNP_00336 | CDS | 936 |  |  | COG0330 | Putative protein |
| SB021 contig 1 | LEJJMMNP_00337 | CDS | 798 |  |  |  | Hypothetical protein |
| SB021 contig 1 | LEJJMMNP_00338 | CDS | 792 |  |  |  | Hypothetical protein |
| SB021 contig 1 | LEJJMMNP_00339 | CDS | 1029 | *hrcA* |  | COG1420 | Heat-inducible transcription repressor HrcA |
| SB021 contig 1 | LEJJMMNP_00340 | CDS | 567 | *grpE* |  | COG0576 | Protein GrpE |
| SB021 contig 1 | LEJJMMNP_00341 | CDS | 1827 | *dnaK* |  | COG0443 | Chaperone protein DnaK |
| SB021 contig 1 | LEJJMMNP_00342 | CDS | 471 | *ogt* | 2.1.1.63 |  | Methylated-DNA—protein-cysteine methyltransferase |
| SB021 contig 1 | LEJJMMNP_00343 | CDS | 879 | *lacX* |  |  | Protein LacX, plasmid |
| SB021 contig 1 | LEJJMMNP_00344 | CDS | 1167 | *dnaJ_1* |  |  | Chaperone protein DnaJ |
| SB021 contig 1 | LEJJMMNP_00345 | CDS | 270 | *yajC* |  | COG1862 | Sec translocon accessory complex subunit YajC |
| SB021 contig 1 | LEJJMMNP_00346 | CDS | 1026 |  |  |  | Hypothetical protein |
| SB021 contig 1 | LEJJMMNP_00347 | CDS | 624 |  |  |  | Hypothetical protein |
| SB021 contig 1 | LEJJMMNP_00348 | CDS | 564 |  |  |  | Hypothetical protein |
| SB021 contig 1 | LEJJMMNP_00349 | CDS | 2130 | *rnr* | 3.1.13.1 | COG0557 | Ribonuclease R |
| SB021 contig 1 | LEJJMMNP_00350 | CDS | 441 | *smpB* |  | COG0691 | SsrA-binding protein |
| SB021 contig 1 | LEJJMMNP_00351 | tmRNA | 350 | *ssrA* |  |  | Transfer-messenger RNA, SsrA |
| SB021 contig 1 | LEJJMMNP_00352 | CDS | 972 | *corA_1* |  | COG0598 | Cobalt/magnesium transport protein CorA |
| SB021 contig 1 | LEJJMMNP_00353 | tRNA | 76 |  |  |  | tRNA-Gly(tcc) |
| SB021 contig 1 | LEJJMMNP_00354 | tRNA | 76 |  |  |  | tRNA-Lys(ctt) |
| SB021 contig 1 | LEJJMMNP_00355 | CDS | 1323 | *der* |  | COG1160 | GTPase Der |
| SB021 contig 1 | LEJJMMNP_00356 | CDS | 1788 |  |  |  | Hypothetical protein |
| SB021 contig 1 | LEJJMMNP_00357 | CDS | 765 | *srlR* |  | COG1349 | Glucitol operon repressor |
| SB021 contig 1 | LEJJMMNP_00358 | CDS | 2355 | *pflD* | 4.2.1.172 | COG1882 | Trans-4-hydroxyl-L-proline dehydratase |
| SB021 contig 1 | LEJJMMNP_00359 | CDS | 912 | *hpdA* | 1.97.1.- | COG1180 | 4-hydroxyphenylacetate decarboxylase activating enzyme |
| SB021 contig 1 | LEJJMMNP_00360 | CDS | 678 | *fsaA* | 4.1.2.- | COG0176 | Fructose-6-phosphate aldolase 1 |
| SB021 contig 1 | LEJJMMNP_00361 | CDS | 1614 |  | 3.6.1.1 |  | Cobalt-dependent inorganic pyrophosphatase |
| SB021 contig 1 | LEJJMMNP_00362 | CDS | 672 |  |  |  | Hypothetical protein |
| SB021 contig 1 | LEJJMMNP_00363 | CDS | 123 |  |  |  | Hypothetical protein |
| SB021 contig 1 | LEJJMMNP_00364 | CDS | 870 |  |  |  | Nucleotide-binding protein |
| SB021 contig 1 | LEJJMMNP_00365 | CDS | 1731 | *uvrC* |  | COG0322 | UvrABC system protein C |
| SB021 contig 1 | LEJJMMNP_00366 | CDS | 951 |  |  |  | Hypothetical protein |
| SB021 contig 1 | LEJJMMNP_00367 | CDS | 1512 |  |  |  | Hypothetical protein |
| SB021 contig 1 | LEJJMMNP_00368 | CDS | 915 |  |  |  | Hypothetical protein |
| SB021 contig 1 | LEJJMMNP_00369 | CDS | 732 | *sfsA* |  | COG1489 | Sugar fermentation stimulation protein A |
| SB021 contig 1 | LEJJMMNP_00370 | CDS | 825 | *apbC* |  | COG0489 | Iron-sulfur cluster carrier protein |
| SB021 contig 1 | LEJJMMNP_00371 | CDS | 1197 | *uctC* | 2.8.3.19 |  | Acetyl-CoA:oxalate CoA-transferase |
| SB021 contig 1 | LEJJMMNP_00372 | CDS | 399 |  |  |  | Hypothetical protein |
| SB021 contig 1 | LEJJMMNP_00373 | CDS | 507 |  |  |  | Hypothetical protein |
| SB021 contig 1 | LEJJMMNP_00374 | CDS | 1626 |  |  |  | Hypothetical protein |
| SB021 contig 1 | LEJJMMNP_00375 | CDS | 498 |  | 2.3.1.- |  | L-methionine sulfoximine/L-methionine sulfone acetyltransferase |
| SB021 contig 1 | LEJJMMNP_00376 | CDS | 690 |  |  |  | Hypothetical protein |
| SB021 contig 1 | LEJJMMNP_00377 | CDS | 993 |  |  |  | Hypothetical protein |
| SB021 contig 1 | LEJJMMNP_00378 | CDS | 645 |  |  |  | Hypothetical protein |
| SB021 contig 1 | LEJJMMNP_00379 | CDS | 642 |  |  |  | Hypothetical protein |
| SB021 contig 1 | LEJJMMNP_00380 | CDS | 747 |  |  |  | Hypothetical protein |
| SB021 contig 1 | LEJJMMNP_00381 | CDS | 885 | *accD* | 2.1.3.15 | COG0777 | Acetyl-coenzyme A carboxylase carboxyl transferase subunit beta |
| SB021 contig 1 | LEJJMMNP_00382 | CDS | 945 | *accA* | 2.1.3.15 | COG0825 | Acetyl-coenzyme A carboxylase carboxyl transferase subunit alpha |
| SB021 contig 1 | LEJJMMNP_00383 | CDS | 966 | *pfkA* | 2.7.1.11 | COG0205 | ATP-dependent 6-phosphofructokinase |
| SB021 contig 1 | LEJJMMNP_00384 | CDS | 1632 | *rqcH* |  | COG1293 | Rqc2 RqcH |
| SB021 contig 1 | LEJJMMNP_00385 | CDS | 345 |  |  |  | Hypothetical protein |
| SB021 contig 1 | LEJJMMNP_00386 | CDS | 336 |  |  |  | Hypothetical protein |
| SB021 contig 1 | LEJJMMNP_00387 | CDS | 642 | *rpe* | 5.1.3.1 |  | Ribulose-phosphate-3-epimerase |
| SB021 contig 1 | LEJJMMNP_00388 | CDS | 894 | *rsgA* | 3.6.1.- | COG1162 | Small ribosomal subunit biogenesis GTPase RsgA |
| SB021 contig 1 | LEJJMMNP_00389 | CDS | 705 | *spk1* | 2.7.11.1 |  | Serine/threonine-protein kinase PK-1 |
| SB021 contig 1 | LEJJMMNP_00390 | CDS | 525 | *hpt* | 2.4.2.8 | COG0634 | Hypoxanthine phosphoribosyltransferase |
| SB021 contig 1 | LEJJMMNP_00391 | CDS | 795 | *rsmA* | 2.1.1.182 |  | Ribosomal RNA small subunit methyltransferase A |
| SB021 contig 1 | LEJJMMNP_00392 | CDS | 240 |  |  |  | Hypothetical protein |
| SB021 contig 1 | LEJJMMNP_00393 | CDS | 240 |  |  |  | Hypothetical protein |
| SB021 contig 1 | LEJJMMNP_00394 | CDS | 537 | *rimM* |  |  | Ribosome maturation factor RimM |
| SB021 contig 1 | LEJJMMNP_00395 | CDS | 729 | *trmD* | 2.1.1.228 |  | tRNA (guanine-N(1)-)-methyltransferase |
| SB021 contig 1 | LEJJMMNP_00396 | CDS | 522 | *yrdA* |  | COG0663 | Protein YrdA |
| SB021 contig 1 | LEJJMMNP_00397 | CDS | 564 |  |  | COG4752 | Putative protein |
| SB021 contig 1 | LEJJMMNP_00398 | CDS | 4326 | *polC* | 2.7.7.7 | COG2176 | DNA polymerase III PolC-type |
| SB021 contig 1 | LEJJMMNP_00399 | CDS | 930 | *impX* |  |  | Riboflavin transporter ImpX |
| SB021 contig 1 | LEJJMMNP_00400 | CDS | 444 |  |  |  | Hypothetical protein |
| SB021 contig 1 | LEJJMMNP_00401 | CDS | 90 |  |  |  | Hypothetical protein |
| SB021 contig 1 | LEJJMMNP_00402 | CDS | 1443 | *opuE_1* |  | COG0591 | Osmoregulated proline transporter OpuE |
| SB021 contig 1 | LEJJMMNP_00403 | CDS | 1443 | *murE* | 6.3.2.13 | COG0769 | UDP-N-acetylmuramoyl-L-alanyl-D-glutamate--2,6-diaminopimelate ligase |
| SB021 contig 1 | LEJJMMNP_00404 | CDS | 843 | *kdsA* | 2.5.1.55 | COG2877 | 2-dehydro-3-deoxyphosphooctonate aldolase |
| SB021 contig 1 | LEJJMMNP_00405 | CDS | 459 |  |  |  | Hypothetical protein |
| SB021 contig 1 | LEJJMMNP_00406 | CDS | 789 | *zupT* |  | COG0428 | Zinc transporter ZupT |
| SB021 contig 1 | LEJJMMNP_00407 | CDS | 984 | *dcyD* | 4.4.1.15 | COG2515 | D-cysteine desulfhydrase |
| SB021 contig 1 | LEJJMMNP_00408 | CDS | 450 |  |  |  | Hypothetical protein |
| SB021 contig 1 | LEJJMMNP_00409 | CDS | 318 |  |  |  | Hypothetical protein |
| SB021 contig 1 | LEJJMMNP_00410 | CDS | 450 | *sigW* |  | COG1595 | ECF RNA polymerase sigma factor SigW |
| SB021 contig 1 | LEJJMMNP_00411 | CDS | 438 |  |  |  | Hypothetical protein |
| SB021 contig 1 | LEJJMMNP_00412 | CDS | 1047 | *ispG* | 1.17.7.3 | COG0821 | 4-hydroxy-3-methylbut-2-en-1-yl diphosphate synthase (flavodoxin) |
| SB021 contig 1 | LEJJMMNP_00413 | CDS | 1029 |  |  |  | Hypothetical protein |
| SB021 contig 1 | LEJJMMNP_00414 | CDS | 1080 |  |  |  | Hypothetical protein |
| SB021 contig 1 | LEJJMMNP_00415 | CDS | 1245 | *rho* | 3.6.4.- | COG1158 | Transcription termination factor Rho |
| SB021 contig 1 | LEJJMMNP_00416 | CDS | 1317 | *miaB* | 2.8.4.3 | COG0621 | tRNA-2-methylthio-N(6)-dimethylallyladenosine synthase |
| SB021 contig 1 | LEJJMMNP_00417 | CDS | 654 | *ogg* |  | COG1059 | Putative N-glycosylase/DNA lyase |
| SB021 contig 1 | LEJJMMNP_00418 | CDS | 882 |  |  |  | Hypothetical protein |
| SB021 contig 1 | LEJJMMNP_00419 | CDS | 195 |  |  |  | Hypothetical protein |
| SB021 contig 1 | LEJJMMNP_00420 | CDS | 1332 |  |  |  | Hypothetical protein |
| SB021 contig 1 | LEJJMMNP_00421 | CDS | 3414 | *dnaE* | 2.7.7.7 | COG0587 | DNA polymerase III subunit alpha |
| SB021 contig 1 | LEJJMMNP_00422 | CDS | 423 |  |  |  | Hypothetical protein |
| SB021 contig 1 | LEJJMMNP_00423 | CDS | 1341 | *accC* | 6.3.4.14 | COG0439 | Biotin carboxylase |
| SB021 contig 1 | LEJJMMNP_00424 | CDS | 366 |  |  | COG1302 | Alkaline shock protein 23 |
| SB021 contig 1 | LEJJMMNP_00425 | CDS | 510 |  |  |  | Hypothetical protein |
| SB021 contig 1 | LEJJMMNP_00426 | CDS | 210 |  |  |  | Hypothetical protein |
| SB021 contig 1 | LEJJMMNP_00427 | CDS | 396 | *nusB* |  | COG0781 | Transcription antitermination protein NusB |
| SB021 contig 1 | LEJJMMNP_00428 | CDS | 540 |  |  |  | Hypothetical protein |
| SB021 contig 1 | LEJJMMNP_00429 | CDS | 321 | *hxlR* |  | COG1733 | HTH-type transcriptional activator HxlR |
| SB021 contig 1 | LEJJMMNP_00430 | CDS | 597 |  |  |  | Hypothetical protein |
| SB021 contig 1 | LEJJMMNP_00431 | CDS | 822 | *aptA_1* | 2.2.1.- | COG3959 | Apulose-4-phosphate transketolase subunit A |
| SB021 contig 1 | LEJJMMNP_00432 | CDS | 927 | *aptB_1* | 2.2.1.- | COG3958 | Apulose-4-phosphate transketolase subunit B |
| SB021 contig 1 | LEJJMMNP_00433 | CDS | 831 |  |  |  | Hypothetical protein |
| SB021 contig 1 | LEJJMMNP_00434 | CDS | 1092 | *envC* |  | COG4942 | Murein hydrolase activator EnvC |
| SB021 contig 1 | LEJJMMNP_00435 | CDS | 813 | *nadK* | 2.7.1.23 | COG0061 | NAD kinase |
| SB021 contig 1 | LEJJMMNP_00436 | CDS | 1407 | *recN* |  | COG0497 | DNA repair protein RecN |
| SB021 contig 1 | LEJJMMNP_00437 | CDS | 189 |  |  |  | Hypothetical protein |
| SB021 contig 1 | LEJJMMNP_00438 | CDS | 237 |  |  |  | Hypothetical protein |
| SB021 contig 1 | LEJJMMNP_00439 | CDS | 351 |  |  |  | Hypothetical protein |
| SB021 contig 1 | LEJJMMNP_00440 | CDS | 894 | *era* |  | COG1159 | GTPase Era |
| SB021 contig 1 | LEJJMMNP_00441 | CDS | 1317 | *pbuG* |  | COG2252 | Guanine/hypoxanthine permease PbuG |
| SB021 contig 1 | LEJJMMNP_00442 | CDS | 492 | *tadA_2* | 3.5.4.33 |  | tRNA-specific adenosine deaminase |
| SB021 contig 1 | LEJJMMNP_00443 | CDS | 981 | *trpS* | 6.1.1.2 | COG0180 | Tryptophan—tRNA ligase |
| SB021 contig 1 | LEJJMMNP_00444 | CDS | 948 | *glcK* | 2.7.1.2 | COG1940 | Glucokinase |
| SB021 contig 1 | LEJJMMNP_00445 | CDS | 660 | *phoP_1* |  | CPG0745 | Alkaline phosphatase synthesis transcriptional regulatory protein PhoP |
| SB021 contig 1 | LEJJMMNP_00446 | CDS | 1347 | *sasA_2* | 2.7.-.- |  | Adaptive-response sensory-kinase SasA |
| SB021 contig 1 | LEJJMMNP_00447 | CDS | 372 |  |  |  | Hypothetical protein |
| SB021 contig 1 | LEJJMMNP_00448 | CDS | 999 |  |  |  | Hypothetical protein |
| SB021 contig 1 | LEJJMMNP_00449 | CDS | 441 |  |  |  | Hypothetical protein |
| SB021 contig 1 | LEJJMMNP_00450 | CDS | 483 |  | 2.7.1- |  | putative phosphotransferase enzyme IIB component |
| SB021 contig 1 | LEJJMMNP_00451 | CDS | 801 | *agaC_2* |  | COG3715 | N-acetylgalactosamine permease IIC component 1 |
| SB021 contig 1 | LEJJMMNP_00452 | CDS | 819 | *manZ_2* |  | COG3716 | PTS system mannose-specific EIID component |
| SB021 contig 1 | LEJJMMNP_00453 | CDS | 1119 | *glmS_1* | 2.6.1.16 |  | Glutamine--fructose-6-phosphate aminotransferase [isomerizing] |
| SB021 contig 1 | LEJJMMNP_00454 | CDS | 714 | *nagR* |  | COG2188 | HTH-type transcriptional repressor NagR |
| SB021 contig 1 | LEJJMMNP_00455 | CDS | 753 |  |  |  | Hypothetical protein |
| SB021 contig 1 | LEJJMMNP_00456 | CDS | 507 | *kdsC* | 3.1.3.45 | COG1778 | 3-deoxy-D-manno-octulosonate 8-phosphate phosphatase KdsC |
| SB021 contig 1 | LEJJMMNP_00457 | CDS | 561 | *cobU* | 2.7.1.156 | COG2087 | Bifunctional adenosylcobalamin biosynthesis protein CobU |
| SB021 contig 1 | LEJJMMNP_00458 | CDS | 831 | *cobS* | 2.7.8.26 | COG0368 | Adenosylcobinamide-GDP ribazoletransferase |
| SB021 contig 1 | LEJJMMNP_00459 | CDS | 591 | *pspA_1* | 3.1.3.3 | COG0406 | Phosphoserine phosphatase 1 |
| SB021 contig 1 | LEJJMMNP_00460 | CDS | 1059 | *cobT* | 2.4.2.21 | COG2038 | Nicotinate-nucleotide--dimethylbenzimidazole phosphoribosyltransferase |
| SB021 contig 1 | LEJJMMNP_00461 | CDS | 672 |  |  |  | Hypothetical protein |
| SB021 contig 1 | LEJJMMNP_00462 | CDS | 954 |  |  |  | Hypothetical protein |
| SB021 contig 1 | LEJJMMNP_00463 | CDS | 489 | *yfiC* | 2.1.1.223 |  | tRNA1(Val) (adenine(37)-N6)-methyltransferase |
| SB021 contig 1 | LEJJMMNP_00464 | CDS | 369 |  |  |  | Hypothetical protein |
| SB021 contig 1 | LEJJMMNP_00465 | CDS | 1989 | *uvrB* |  |  | UvrABC system protein B |
| SB021 contig 1 | LEJJMMNP_00466 | CDS | 1200 | *xseA* | 3.1.11.6 | COG1570 | Exodeoxyribonuclease 7 large subunit |
| SB021 contig 1 | LEJJMMNP_00467 | CDS | 852 |  |  |  | Hypothetical protein |
| SB021 contig 1 | LEJJMMNP_00468 | CDS | 1071 |  |  |  | Hypothetical protein |
| SB021 contig 1 | LEJJMMNP_00469 | CDS | 2310 | *topA* | 5.6.2.1 |  | DNA topoisomerase 1 |
| SB021 contig 1 | LEJJMMNP_00470 | CDS | 882 | *rhaS* |  |  | HTH-type transcriptional activator RhaS |
| SB021 contig 1 | LEJJMMNP_00471 | CDS | 1425 | *melB_1* |  | COG2211 | Melibiose carrier protein |
| SB021 contig 1 | LEJJMMNP_00472 | CDS | 2244 | *agaA* | 3.2.1.22 |  | Alpha-galactosidase AgaA |
| SB021 contig 1 | LEJJMMNP_00473 | CDS | 1401 | *sacA* | 3.2.1.26 |  | Sucrose-6-phosphate hydrolase |
| SB021 contig 1 | LEJJMMNP_00474 | CDS | 417 |  |  |  | Hypothetical protein |
| SB021 contig 1 | LEJJMMNP_00475 | CDS | 822 |  |  |  | Hypothetical protein |
| SB021 contig 1 | LEJJMMNP_00476 | CDS | 567 |  |  |  | Hypothetical protein |
| SB021 contig 1 | LEJJMMNP_00477 | CDS | 213 |  |  |  | Hypothetical protein |
| SB021 contig 1 | LEJJMMNP_00478 | CDS | 963 | *kdsD* | 5.3.1.13 | COG0517 | Arabinose 5-phosphate isomerase KdsD |
| SB021 contig 1 | LEJJMMNP_00479 | CDS | 729 | *rutF* | 1.5.1.42 |  | FMN reductase (NADH) RutF |
| SB021 contig 1 | LEJJMMNP_00480 | CDS | 1209 |  | 2.6.1.1 | COG0436 | Aspartate/prephenate aminotransferase |
| SB021 contig 1 | LEJJMMNP_00481 | CDS | 210 |  |  |  | Hypothetical protein |
| SB021 contig 1 | LEJJMMNP_00482 | CDS | 942 |  |  |  | Hypothetical protein |
| SB021 contig 1 | LEJJMMNP_00483 | CDS | 855 |  |  |  | Hypothetical protein |
| SB021 contig 1 | LEJJMMNP_00484 | CDS | 792 | *potA_1* | 7.6.2.11 | COG3842 | Spermidine/putrescine import ATP-binding protein PotA |
| SB021 contig 1 | LEJJMMNP_00485 | CDS | 840 |  |  |  | Hypothetical protein |
| SB021 contig 1 | LEJJMMNP_00486 | CDS | 789 | *murI* | 5.1.1.3 | COG0796 | Glutamate racemase |
| SB021 contig 1 | LEJJMMNP_00487 | CDS | 1215 | *gltS* |  | COG0786 | Sodium/glutamate symporter |
| SB021 contig 1 | LEJJMMNP_00488 | CDS | 156 |  |  |  | Hypothetical protein |
| SB021 contig 1 | LEJJMMNP_00489 | CDS | 2187 | *feoB* |  | COG0370 | Fe(2+) transporter FeoB |
| SB021 contig 1 | LEJJMMNP_00490 | CDS | 231 |  |  |  | Hypothetical protein |
| SB021 contig 1 | LEJJMMNP_00491 | CDS | 216 |  |  |  | Hypothetical protein |
| SB021 contig 1 | LEJJMMNP_00492 | CDS | 948 | *ttcA_1* | 2.8.1.- |  | tRNA-cytidine(32) 2-sulfurtransferase |
| SB021 contig 1 | LEJJMMNP_00493 | CDS | 822 | *ttcA_2* | 2.8.1.- |  | tRNA-cytidine(32) 2-sulfurtransferase |
| SB021 contig 1 | LEJJMMNP_00494 | CDS | 339 |  |  |  | Hypothetical protein |
| SB021 contig 1 | LEJJMMNP_00495 | CDS | 579 |  |  |  | Hypothetical protein |
| SB021 contig 1 | LEJJMMNP_00496 | CDS | 1341 | *mdtK* |  | COG0534 | Multidrug resistance protein MdtK |
| SB021 contig 1 | LEJJMMNP_00497 | CDS | 882 | *metF* | 1.5.1.20 | COG0685 | 5,10-methylenetetrahydrofolate reductase |
| SB021 contig 1 | LEJJMMNP_00498 | CDS | 3330 | *metH* | 2.1.1.13 | COG0646 | Methionine synthase |
| SB021 contig 1 | LEJJMMNP_00499 | CDS | 462 |  |  |  | Hypothetical protein |
| SB021 contig 1 | LEJJMMNP_00500 | CDS | 486 |  |  |  | Hypothetical protein |
| SB021 contig 1 | LEJJMMNP_00501 | CDS | 852 | *cvfB* |  |  | Conserved virulence factor B |
| SB021 contig 1 | LEJJMMNP_00502 | CDS | 1545 | *abgT* |  | COG2978 | p-aminobenzoyl-glutamate transport protein |
| SB021 contig 1 | LEJJMMNP_00503 | CDS | 1827 | *pepF1* | 3.4.24.- |  | Oligoendopeptidase F, plasmid |
| SB021 contig 1 | LEJJMMNP_00504 | CDS | 873 | *cmpR* |  | COG0583 | HTH-type transcriptional activator CmpR |
| SB021 contig 1 | LEJJMMNP_00505 | CDS | 576 | *gmhA* | 5.3.1.28 | COG0279 | Phosphoheptose isomerase |
| SB021 contig 1 | LEJJMMNP_00506 | CDS | 831 |  | 3.1.-.- |  | Deoxyribonuclease |
| SB021 contig 1 | LEJJMMNP_00507 | CDS | 633 | *thiN* | 2.7.6.2 | COG1564 | Thiamine pyrophosphokinase |
| SB021 contig 1 | LEJJMMNP_00508 | CDS | 1224 |  |  |  | Uracil permease |
| SB021 contig 1 | LEJJMMNP_00509 | CDS | 1146 | *braC_1* |  | COG0683 | Leucine-, isoleucine-, valine-, threonine-, and alanine-binding protein |
| SB021 contig 1 | LEJJMMNP_00510 | CDS | 885 | *livH* |  | COG0559 | High-affinity branched-chain amino acid transport system permease protein LivH |
| SB021 contig 1 | LEJJMMNP_00511 | CDS | 795 |  |  |  | Hypothetical protein |
| SB021 contig 1 | LEJJMMNP_00512 | CDS | 219 |  |  |  | Hypothetical protein |
| SB021 contig 1 | LEJJMMNP_00513 | CDS | 780 | *lptB_1* | 3.6.3.- | COG1137 | Lipopolysaccharide export system ATP-binding protein LptB |
| SB021 contig 1 | LEJJMMNP_00514 | CDS | 729 | *livF* |  | COG0410 | High-affinity branched-chain amino acid transport ATP-binding protein LivF |
| SB021 contig 1 | LEJJMMNP_00515 | CDS | 1302 | *brnQ* |  | COG1114 | Branched-chain amino acid transport system 2 carrier protein |
| SB021 contig 1 | LEJJMMNP_00516 | CDS | 318 |  |  |  | Hypothetical protein |
| SB021 contig 1 | LEJJMMNP_00517 | CDS | 3516 |  |  |  | Hypothetical protein |
| SB021 contig 1 | LEJJMMNP_00518 | CDS | 345 |  |  |  | Hypothetical protein |
| SB021 contig 1 | LEJJMMNP_00519 | CDS | 459 |  |  |  | Hypothetical protein |
| SB021 contig 1 | LEJJMMNP_00520 | CDS | 831 |  |  |  | Hypothetical protein |
| SB021 contig 1 | LEJJMMNP_00521 | CDS | 489 |  |  |  | Hypothetical protein |
| SB021 contig 1 | LEJJMMNP_00522 | CDS | 387 |  |  |  | Hypothetical protein |
| SB021 contig 1 | LEJJMMNP_00523 | CDS | 735 |  |  |  | Hypothetical protein |
| SB021 contig 1 | LEJJMMNP_00524 | CDS | 1122 |  |  |  | Hypothetical protein |
| SB021 contig 1 | LEJJMMNP_00525 | CDS | 1776 |  |  |  | Hypothetical protein |
| SB021 contig 1 | LEJJMMNP_00526 | CDS | 1515 | *comM* |  | COG0606 | Competence protein ComM |
| SB021 contig 1 | LEJJMMNP_00527 | CDS | 795 | *fabM* | 5.3.3.14 | COG1024 | Trans-2-decenoyl-[acyl-carrier-protein] isomerase |
| SB021 contig 1 | LEJJMMNP_00528 | CDS | 720 | *atoD* | 2.8.3.8 | COG1788 | Acetate CoA-transferase subunit alpha |
| SB021 contig 1 | LEJJMMNP_00529 | CDS | 660 | *atoA* | 2.8.3.8 | COG2057 | Acetate CoA-transferase subunit beta |
| SB021 contig 1 | LEJJMMNP_00530 | CDS | 1320 |  |  |  | Hypothetical protein |
| SB021 contig 1 | LEJJMMNP_00531 | CDS | 1311 | *mepA_1* |  |  | Multidrug export protein MepA |
| SB021 contig 1 | LEJJMMNP_00532 | CDS | 474 | *levE* | 2.7.1.202 | COG3444 | PTS system fructose-specific EIIB component |
| SB021 contig 1 | LEJJMMNP_00533 | CDS | 783 | *agaC_2* |  | COG3715 | N-acetylgalactosamine permease IIC component 1 |
| SB021 contig 1 | LEJJMMNP_00534 | CDS | 810 | *manZ_3* |  | COG3716 | PTS system mannose-specific EIID component |
| SB021 contig 1 | LEJJMMNP_00535 | CDS | 429 | *levD* |  | COG2893 | PTS system fructose-specific EIIA component |
| SB021 contig 1 | LEJJMMNP_00536 | CDS | 285 | *rpmA* |  |  | 50S ribosomal protein L27 |
| SB021 contig 1 | LEJJMMNP_00537 | CDS | 336 |  |  |  | Hypothetical protein |
| SB021 contig 1 | LEJJMMNP_00538 | CDS | 312 | *rplU* |  | COG0261 | 50S ribosomal protein L21 |
| SB021 contig 1 | LEJJMMNP_00539 | CDS | 525 |  |  |  | Hypothetical protein |
| SB021 contig 1 | LEJJMMNP_00540 | CDS | 504 | *isiB* |  |  | Flavodoxin |
| SB021 contig 1 | LEJJMMNP_00541 | CDS | 1329 | *stet* |  | COG0531 | Serine/threonine exchanger SteT |
| SB021 contig 1 | LEJJMMNP_00542 | CDS | 1311 | *lysA* | 4.1.1.20 | COG0019 | Diaminopimelate decarboxylase |
| SB021 contig 1 | LEJJMMNP_00543 | CDS | 843 | *dapF* | 5.1.1.7 | COG0253 | Diaminopimelate epimerase |
| SB021 contig 1 | LEJJMMNP_00544 | CDS | 1212 | *ftsW* | 2.4.1.129 | COG0772 | putative peptidoglycan glycosyltransferase FtsW |
| SB021 contig 1 | LEJJMMNP_00545 | CDS | 198 |  |  |  | Hypothetical protein |
| SB021 contig 1 | LEJJMMNP_00546 | CDS | 1392 | *asnS* | 6.1.1.22 | COG0017 | Asparagine—tRNA ligase |
| SB021 contig 1 | LEJJMMNP_00547 | CDS | 1026 |  |  |  | Hypothetical protein |
| SB021 contig 1 | LEJJMMNP_00548 | CDS | 306 |  |  |  | Hypothetical protein |
| SB021 contig 1 | LEJJMMNP_00549 | CDS | 1806 |  |  |  | Hypothetical protein |
| SB021 contig 1 | LEJJMMNP_00550 | CDS | 747 | *mipA* |  | COG3713 | MltA-interacting protein |
| SB021 contig 1 | LEJJMMNP_00551 | CDS | 1155 | *metK* | 2.5.1.6 |  | S-adenosylmethionine synthase |
| SB021 contig 1 | LEJJMMNP_00552 | CDS | 393 | *rimP* |  |  | Ribosome maturation factor RimP |
| SB021 contig 1 | LEJJMMNP_00553 | CDS | 1104 | *nusA* |  | COG0195 | Transcription termination/antitermination protein NusA |
| SB021 contig 1 | LEJJMMNP_00554 | CDS | 525 |  |  |  | Hypothetical protein |
| SB021 contig 1 | LEJJMMNP_00555 | CDS | 2103 | *infB* |  |  | Translation initiation factor IF-2 |
| SB021 contig 1 | LEJJMMNP_00556 | CDS | 366 | *rbfA* |  |  | Ribosome-binding factor A |
| SB021 contig 1 | LEJJMMNP_00557 | CDS | 1686 | *recJ* | 3.1.-.- | COG0608 | Single-stranded-DNA-specific exonuclease RecJ |
| SB021 contig 1 | LEJJMMNP_00558 | CDS | 1290 | *tig* | 5.2.1.8 | COG0544 | Trigger factor |
| SB021 contig 1 | LEJJMMNP_00559 | CDS | 234 | *clpP_1* | 3.4.21.92 | COG0740 | ATP-dependent Clp protease proteolytic subunit |
| SB021 contig 1 | LEJJMMNP_00560 | CDS | 258 | *clpP_2* | 3.4.21.92 | COG0740 | ATP-dependent Clp protease proteolytic subunit |
| SB021 contig 1 | LEJJMMNP_00561 | CDS | 174 |  |  |  | Hypothetical protein |
| SB021 contig 1 | LEJJMMNP_00562 | CDS | 975 | *clpX* |  | COG1219 | ATP-dependent Clp protease ATP-binding subunit ClpX |
| SB021 contig 1 | LEJJMMNP_00563 | CDS | 2316 | *lon* | 3.4.21.53 | COG0466 | Lon protease |
| SB021 contig 1 | LEJJMMNP_00564 | CDS | 609 | *engB* |  | COG0218 | putative GTP-binding protein EngB |
| SB021 contig 1 | LEJJMMNP_00565 | CDS | 2661 | *valS* | 6.1.1.9 | COG0525 | Valine—tRNA ligase |
| SB021 contig 1 | LEJJMMNP_00566 | CDS | 486 | *purE* | 5.4.99.18 | COG0041 | N5-carboxyaminoimidazole ribonucleotide mutase |
| SB021 contig 1 | LEJJMMNP_00567 | CDS | 1110 | *purK* | 6.3.4.18 |  | N5-carboxyaminoimidazole ribonucleotide synthase |
| SB021 contig 1 | LEJJMMNP_00568 | CDS | 3693 | *purL* | 6.3.5.3 |  | Phosphoribosylformylglycinamidine synthase |
| SB021 contig 1 | LEJJMMNP_00569 | CDS | 1410 | *purF* | 2.4.2.14 | COG0034 | Amidophosphoribosyltransferase |
| SB021 contig 1 | LEJJMMNP_00570 | CDS | 1032 | *purM* | 6.3.3.1 |  | Phosphoribosylformylglycinamidine cyclo-ligase |
| SB021 contig 1 | LEJJMMNP_00571 | CDS | 588 | *purN* | 2.1.2.2 |  | Phosphoribosylglycinamide formyltransferase |
| SB021 contig 1 | LEJJMMNP_00572 | CDS | 1533 | *purH* |  |  | Bifunctional purine biosynthesis protein PurH |
| SB021 contig 1 | LEJJMMNP_00573 | CDS | 1236 | *purD* | 6.3.4.13 | COG0151 | Phosphoribosylamine--glycine ligase |
| SB021 contig 1 | LEJJMMNP_00574 | CDS | 126 | *guaB_1* | 1.1.1.205 | COG0516 | Inosine-5'-monophosphate dehydrogenase |
| SB021 contig 1 | LEJJMMNP_00575 | CDS | 1329 | *guaB_2* | 1.1.1.205 | COG0516 | Inosine-5'-monophosphate dehydrogenase |
| SB021 contig 1 | LEJJMMNP_00576 | CDS | 567 |  |  |  | Hypothetical protein |
| SB021 contig 1 | LEJJMMNP_00577 | CDS | 1479 |  |  |  | IS1182 family transposase ISFnu2 |
| SB021 contig 1 | LEJJMMNP_00578 | CDS | 882 | *dagK* | 2.7.1.107 | COG1597 | Diacylglycerol kinase |
| SB021 contig 1 | LEJJMMNP_00579 | CDS | 1668 | *eptC* | 2.7.-.- | COG2194 | Phosphoethanolamine transferase EptC |
| SB021 contig 1 | LEJJMMNP_00580 | CDS | 1113 | *rfaQ* | 2.-.-.- | COG0859 | Lipopolysaccharide core heptosyltransferase RfaQ |
| SB021 contig 1 | LEJJMMNP_00581 | CDS | 1173 | *gtfA_2* | 2.4.1.- |  | UDP-N-acetylglucosamine--peptide N-acetylglucosaminyltransferase GtfA subunit |
| SB021 contig 1 | LEJJMMNP_00582 | CDS | 1095 |  |  |  | Hypothetical protein |
| SB021 contig 1 | LEJJMMNP_00583 | CDS | 1185 | *pglH* | 2.4.1.292 | COG0438 | GalNAc-alpha-(1->4)-GalNAc-alpha-(1->3)-diNAcBac-PP-undecaprenol alpha-1,4-N-acetyl-D-galactosaminyltransferase |
| SB021 contig 1 | LEJJMMNP_00584 | CDS | 753 |  |  |  | Hypothetical protein |
| SB021 contig 1 | LEJJMMNP_00585 | CDS | 951 |  |  |  | Hypothetical protein |
| SB021 contig 1 | LEJJMMNP_00586 | CDS | 1701 |  |  |  | Hypothetical protein |
| SB021 contig 1 | LEJJMMNP_00587 | CDS | 585 |  |  |  | Hypothetical protein |
| SB021 contig 1 | LEJJMMNP_00588 | CDS | 582 |  |  |  | Hypothetical protein |
| SB021 contig 1 | LEJJMMNP_00589 | CDS | 870 | *rfbA_1* | 2.7.7.24 | COG1209 | Glucose-1-phosphate thymidylyltransferase 1 |
| SB021 contig 1 | LEJJMMNP_00590 | CDS | 576 | *rfbC_1* | 5.1.3.13 | COG1898 | dTDP-4-dehydrorhamnose 3,5-epimerase |
| SB021 contig 1 | LEJJMMNP_00591 | CDS | 882 | *rfbD_1* | 1.1.1.133 | COG1091 | dTDP-4-dehydrorhamnose reductase |
| SB021 contig 1 | LEJJMMNP_00592 | CDS | 621 | *dapH_1* | 2.3.1.89 |  | 2,3,4,5-tetrahydropyridine-2,6-dicarboxylate N-acetyltransferase |
| SB021 contig 1 | LEJJMMNP_00593 | CDS | 1194 | *rffG_1* | 4.2.1.46 | COG1088 | dTDP-glucose 4,6-dehydratase 2 |
| SB021 contig 1 | LEJJMMNP_00594 | CDS | 1125 | *pglJ* | 2.4.1.291 | COG0438 | N-acetylgalactosamine-N,N'-diacetylbacillosaminyl-diphospho-undecaprenol 4-alpha-N-acetylgalactosaminyltransferase |
| SB021 contig 1 | LEJJMMNP_00595 | CDS | 972 |  |  |  | Hypothetical protein |
| SB021 contig 1 | LEJJMMNP_00596 | CDS | 639 | *dapH_2* | 2.3.1.89 |  | 2,3,4,5-tetrahydropyridine-2,6-dicarboxylate N-acetyltransferase |
| SB021 contig 1 | LEJJMMNP_00597 | CDS | 1065 | *corA_2* |  | COG0598 | Cobalt/magnesium transport protein CorA |
| SB021 contig 1 | LEJJMMNP_00598 | CDS | 1614 | *sasA_3* | 2.7.-.- |  | Adaptive-response sensory-kinase SasA |
| SB021 contig 1 | LEJJMMNP_00599 | CDS | 657 | *srrA* |  | COG0745 | Transcriptional regulatory protein SrrA |
| SB021 contig 1 | LEJJMMNP_00600 | CDS | 1440 | *gatB* | 6.3.5.- | COG0064 | Aspartyl/glutamyl-tRNA(Asn/Gln) amidotransferase subunit B |
| SB021 contig 1 | LEJJMMNP_00601 | CDS | 1464 | *gatA* | 6.3.5.7 | COG0154 | Glutamyl-tRNA(Gln) amidotransferase subunit A |
| SB021 contig 1 | LEJJMMNP_00602 | CDS | 294 | *gatC* | 6.3.5.- | COG0721 | Glutamyl-tRNA(Gln) amidotransferase subunit C |
| SB021 contig 1 | LEJJMMNP_00603 | CDS | 711 | *rluB* | 5.4.99.22 | COG1187 | Ribosomal large subunit pseudouridine synthase B |
| SB021 contig 1 | LEJJMMNP_00604 | CDS | 531 | *scpB* |  |  | Segregation and condensation protein B |
| SB021 contig 1 | LEJJMMNP_00605 | CDS | 1053 | *mreB_1* |  | COG1077 | Cell shape-determining protein MreB |
| SB021 contig 1 | LEJJMMNP_00606 | CDS | 321 | *maf* | 3.6.1.9 | COG0424 | dTTP/UTP pyrophosphatase |
| SB021 contig 1 | LEJJMMNP_00607 | CDS | 162 |  | 3.6.1.9 |  | dTTP/UTP pyrophosphatase |
| SB021 contig 1 | LEJJMMNP_00608 | CDS | 771 |  |  |  | Hypothetical protein |
| SB021 contig 1 | LEJJMMNP_00609 | CDS | 444 |  |  |  | Hypothetical protein |
| SB021 contig 1 | LEJJMMNP_00610 | CDS | 462 |  |  |  | Hypothetical protein |
| SB021 contig 1 | LEJJMMNP_00611 | CDS | 270 |  |  |  | Hypothetical protein |
| SB021 contig 1 | LEJJMMNP_00612 | rRNA | 97 |  |  |  | 5S ribosomal RNA |
| SB021 contig 1 | LEJJMMNP_00613 | CDS | 480 |  |  |  | Hypothetical protein |
| SB021 contig 1 | LEJJMMNP_00614 | CDS | 825 | *thyA1* | 2.1.1.45 | COG0207 | Thymidylate synthase 1 |
| SB021 contig 1 | LEJJMMNP_00615 | CDS | 795 | *ycdX* | 3.1.3.- | COG1387 | Putative phosphatase YcdX |
| SB021 contig 1 | LEJJMMNP_00616 | CDS | 948 |  | 1.6.5.11 | COG2070 | NADH:quinone reductase |
| SB021 contig 1 | LEJJMMNP_00617 | CDS | 1365 | *alsT_1* |  | COG1115 | Amino-acid carrier protein AlsT |
| SB021 contig 1 | LEJJMMNP_00618 | CDS | 1230 |  | 3.4.11.- |  | Aminopeptidase 2 |
| SB021 contig 1 | LEJJMMNP_00619 | CDS | 1113 | *potA_2* | 7.6.2.11 | COG3842 | Spermidine/putrescine import ATP-binding protein PotA |
| SB021 contig 1 | LEJJMMNP_00620 | CDS | 840 | *potB* |  | COG1176 | Spermidine/putrescine transport system permease protein PotB |
| SB021 contig 1 | LEJJMMNP_00621 | CDS | 774 | *ydcV* |  |  | Inner membrane ABC transporter permease protein YdcV |
| SB021 contig 1 | LEJJMMNP_00622 | CDS | 846 |  |  |  | Hypothetical protein |
| SB021 contig 1 | LEJJMMNP_00623 | CDS | 543 | *yajL* | 3.1.2.- | COG0693 | Protein/nucleic acid deglycase 3 |
| SB021 contig 1 | LEJJMMNP_00624 | CDS | 1170 | *nanK* | 2.7.1.60 |  | N-acetylmannosamine kinase |
| SB021 contig 1 | LEJJMMNP_00625 | CDS | 966 |  |  |  | Hypothetical protein |
| SB021 contig 1 | LEJJMMNP_00626 | CDS | 681 | *purC* | 6.3.2.6 | COG0152 | Phosphoribosylaminoimidazole-succinocarboxamide synthase |
| SB021 contig 1 | LEJJMMNP_00627 | CDS | 258 |  |  |  | Hypothetical protein |
| SB021 contig 1 | LEJJMMNP_00628 | CDS | 1017 | *hmo* | 1.1.3.46 |  | 4-hydroxymandelate oxidase |
| SB021 contig 1 | LEJJMMNP_00629 | CDS | 453 | *dtd* | 3.1.1.96 | COG1490 | D-aminoacyl-tRNA deacylase |
| SB021 contig 1 | LEJJMMNP_00630 | CDS | 1521 | *pncB* | 6.3.4.21 |  | Nicotinate phosphoribosyltransferase |
| SB021 contig 1 | LEJJMMNP_00631 | CDS | 570 | *xpt* | 2.4.2.22 |  | Xanthine phosphoribosyltransferase |
| SB021 contig 1 | LEJJMMNP_00632 | CDS | 786 | *focA* |  | COG2116 | Putative formate transporter 1 |
| SB021 contig 1 | LEJJMMNP_00633 | CDS | 1488 | *udk_1* | 2.7.1.48 |  | Uridine kinase |
| SB021 contig 1 | LEJJMMNP_00634 | CDS | 1749 | *clpP_3* | 3.4.21.92 |  | ATP-dependent Clp protease proteolytic subunit |
| SB021 contig 1 | LEJJMMNP_00635 | CDS | 351 |  |  | COG0718 | Nucleoid-associated protein |
| SB021 contig 1 | LEJJMMNP_00636 | CDS | 132 |  |  |  | Hypothetical protein |
| SB021 contig 1 | LEJJMMNP_00637 | CDS | 282 |  |  |  | Hypothetical protein |
| SB021 contig 1 | LEJJMMNP_00638 | CDS | 957 |  |  |  | Hypothetical protein |
| SB021 contig 1 | LEJJMMNP_00639 | CDS | 1455 |  |  |  | Hypothetical protein |
| SB021 contig 1 | LEJJMMNP_00640 | CDS | 3129 | *recB* | 3.1.11.5 |  | RecBCD enzyme subunit RecB |
| SB021 contig 1 | LEJJMMNP_00641 | CDS | 567 |  |  |  | Hypothetical protein |
| SB021 contig 1 | LEJJMMNP_00642 | CDS | 774 |  |  |  | Hypothetical protein |
| SB021 contig 1 | LEJJMMNP_00643 | CDS | 1296 |  |  |  | Hypothetical protein |
| SB021 contig 1 | LEJJMMNP_00644 | CDS | 1233 | *fief* |  |  | Ferrous-iron efflux pump FieF |
| SB021 contig 1 | LEJJMMNP_00645 | CDS | 2199 | *pcrA* | 3.6.4.12 |  | ATP-dependent DNA helicase PcrA |
| SB021 contig 1 | LEJJMMNP_00646 | CDS | 828 | *lpxC* | 3.5.1.108 | COG0774 | UDP-3-O-acyl-N-acetylglucosamine deacetylase |
| SB021 contig 1 | LEJJMMNP_00647 | CDS | 438 | *fabZ* | 4.2.1.59 | COG0764 | 3-hydroxyacyl-[acyl-carrier-protein] dehydratase FabZ |
| SB021 contig 1 | LEJJMMNP_00648 | CDS | 774 | *lpxA* | 2.3.1.129 | COG1043 | Acyl-[acyl-carrier-protein]--UDP-N-acetylglucosamine O-acyltransferase |
| SB021 contig 1 | LEJJMMNP_00649 | CDS | 810 | *lpxI* | 3.6.1.54 | COG3494 | UDP-2,3-diacylglucosamine pyrophosphatase LpxI |
| SB021 contig 1 | LEJJMMNP_00650 | CDS | 1074 | *lpxB* | 2.4.1.182 | COG0763 | Lipid-A-disaccharide synthase |
| SB021 contig 1 | LEJJMMNP_00651 | CDS | 1791 |  | 3.6.3.- | COG1132 | Putative multidrug export ATP-binding/permease protein |
| SB021 contig 1 | LEJJMMNP_00652 | CDS | 759 | *rph* | 2.7.7.56 | COG0689 | Ribonuclease PH |
| SB021 contig 1 | LEJJMMNP_00653 | CDS | 585 |  | 3.6.1.66 |  | dITP/XTP pyrophosphatase |
| SB021 contig 1 | LEJJMMNP_00654 | CDS | 1329 | *hslU* |  | COG1220 | ATP-dependent protease ATPase subunit HslU |
| SB021 contig 1 | LEJJMMNP_00655 | CDS | 1743 | *smc_1* |  | COG1196 | Chromosome partition protein Smc |
| SB021 contig 1 | LEJJMMNP_00656 | CDS | 1743 | *smc_2* |  | COG1196 | Chromosome partition protein Smc |
| SB021 contig 1 | LEJJMMNP_00657 | CDS | 1011 | *lpxK* | 2.7.1.130 | COG1663 | Tetraacyldisaccharide 4'-kinase |
| SB021 contig 1 | LEJJMMNP_00658 | CDS | 822 |  |  |  | Hypothetical protein |
| SB021 contig 1 | LEJJMMNP_00659 | CDS | 456 | *manP_1* |  | COG1299 | PTS system mannose-specific EIIBCA component |
| SB021 contig 1 | LEJJMMNP_00660 | CDS | 609 | *nadD* | 2.7.7.18 |  | Putative nicotinate-nucleotide adenylyltransferase |
| SB021 contig 1 | LEJJMMNP_00661 | CDS | 1479 |  |  |  | IS1182 family transposase ISFnu2 |
| SB021 contig 1 | LEJJMMNP_00662 | CDS | 1383 | *murJ_1* |  |  | Lipid II flippase MurJ |
| SB021 contig 1 | LEJJMMNP_00663 | CDS | 618 | *pspA_2* | 3.1.3.3 | COG0406 | Phosphoserine phosphatase 1 |
| SB021 contig 1 | LEJJMMNP_00664 | CDS | 738 | *kdsB* | 2.7.7.38 | COG1212 | 3-deoxy-manno-octulosonate cytidylyltransferase |
| SB021 contig 1 | LEJJMMNP_00665 | CDS | 1548 |  |  |  | Hypothetical protein |
| SB021 contig 1 | LEJJMMNP_00666 | CDS | 1398 | *norR_2* |  |  | Anaerobic nitric oxide reductase transcription regulator NorR |
| SB021 contig 1 | LEJJMMNP_00667 | CDS | 138 |  |  |  | Hypothetical protein |
| SB021 contig 1 | LEJJMMNP_00668 | CDS | 1185 |  |  |  | Hypothetical protein |
| SB021 contig 1 | LEJJMMNP_00669 | CDS | 777 |  | 4.2.1.150 | COG1024 | Crotonyl-CoA hydratase |
| SB021 contig 1 | LEJJMMNP_00670 | CDS | 1554 | *carA_1* | 2.8.3.23 | COG4670 | Caffeate CoA-transferase |
| SB021 contig 1 | LEJJMMNP_00671 | CDS | 1152 | *bcd_2* | 1.3.8.1 | COG1960 | Acyl-CoA dehydrogenase, short-chain specific |
| SB021 contig 1 | LEJJMMNP_00672 | CDS | 729 |  |  |  | Hypothetical protein |
| SB021 contig 1 | LEJJMMNP_00673 | CDS | 963 | *etfA* |  | COG2025 | Electron transfer flavoprotein subunit alpha |
| SB021 contig 1 | LEJJMMNP_00674 | CDS | 1728 |  |  |  | Hypothetical protein |
| SB021 contig 1 | LEJJMMNP_00675 | CDS | 1251 |  |  |  | Hypothetical protein |
| SB021 contig 1 | LEJJMMNP_00676 | CDS | 777 |  |  |  | Hypothetical protein |
| SB021 contig 1 | LEJJMMNP_00677 | CDS | 1092 | *epsF_1* | 2.4.-.- | COG0438 | Putative glycosyltransferase EpsF |
| SB021 contig 1 | LEJJMMNP_00678 | CDS | 1185 |  |  |  | Hypothetical protein |
| SB021 contig 1 | LEJJMMNP_00679 | CDS | 1110 | *pglA* | 2.4.1.290 |  | N,N'-diacetylbacillosaminyl-diphospho-undecaprenol alpha-1,3-N-acetylgalactosaminyltransferase |
| SB021 contig 1 | LEJJMMNP_00680 | CDS | 987 |  |  |  | Hypothetical protein |
| SB021 contig 1 | LEJJMMNP_00681 | CDS | 996 |  |  |  | Hypothetical protein |
| SB021 contig 1 | LEJJMMNP_00682 | CDS | 1407 |  |  |  | Hypothetical protein |
| SB021 contig 1 | LEJJMMNP_00683 | CDS | 636 |  |  |  | Hypothetical protein |
| SB021 contig 1 | LEJJMMNP_00684 | CDS | 1308 |  |  |  | Hypothetical protein |
| SB021 contig 1 | LEJJMMNP_00685 | CDS | 1095 | *mdtA* |  |  | Multidrug resistance protein MdtA |
| SB021 contig 1 | LEJJMMNP_00686 | CDS | 3081 | *swrC* |  | COG0841 | Swarming motility protein SwrC |
| SB021 contig 1 | LEJJMMNP_00687 | CDS | 393 |  |  |  | Hypothetical protein |
| SB021 contig 1 | LEJJMMNP_00688 | CDS | 2205 |  |  |  | Hypothetical protein |
| SB021 contig 1 | LEJJMMNP_00689 | CDS | 1197 | *htpG_1* |  | COG0326 | Chaperone protein HtpG |
| SB021 contig 1 | LEJJMMNP_00690 | CDS | 627 | *htpG_2* |  | COG0326 | Chaperone protein HtpG |
| SB021 contig 1 | LEJJMMNP_00691 | CDS | 216 |  |  |  | Hypothetical protein |
| SB021 contig 1 | LEJJMMNP_00692 | CDS | 1050 | *rsmB* | 2.1.1.176 | COG0144 | Ribosomal RNA small subunit methyltransferase B |
| SB021 contig 1 | LEJJMMNP_00693 | CDS | 633 | *trmR* | 2.1.1.- | COG4122 | tRNA 5-hydroxyuridine methyltransferase |
| SB021 contig 1 | LEJJMMNP_00694 | CDS | 1083 | *cshA* | 3.6.4.13 | COG0513 | ATP-dependent RNA helicase CshA |
| SB021 contig 1 | LEJJMMNP_00695 | CDS | 510 | *deaD* | 3.6.4.13 |  | ATP-dependent RNA helicase DeaD |
| SB021 contig 1 | LEJJMMNP_00696 | CDS | 945 | *mltG* | 4.2.2.- | COG1559 | Endolytic murein transglycosylase |
| SB021 contig 1 | LEJJMMNP_00697 | CDS | 858 | *bchE* | 1.21.98.3 |  | Anaerobic magnesium-protoporphyrin IX monomethyl ester cyclase |
| SB021 contig 1 | LEJJMMNP_00698 | CDS | 789 |  |  |  | Hypothetical protein |
| SB021 contig 1 | LEJJMMNP_00699 | CDS | 1344 | *tilS* | 6.3.4.19 | COG0037 | tRNA(Ile)-lysidine synthase |
| SB021 contig 1 | LEJJMMNP_00700 | CDS | 2232 | *ftsH* | 3.4.24.- |  | ATP-dependent zinc metalloprotease FtsH |
| SB021 contig 1 | LEJJMMNP_00701 | CDS | 258 | *rpsO* |  |  | 30S ribosomal protein S15 |
| SB021 contig 1 | LEJJMMNP_00702 | CDS | 711 |  |  |  | Queuosine precursor transporter |
| SB021 contig 1 | LEJJMMNP_00703 | CDS | 705 |  |  |  | Hypothetical protein |
| SB021 contig 1 | LEJJMMNP_00704 | CDS | 363 |  |  |  | Hypothetical protein |
| SB021 contig 1 | LEJJMMNP_00705 | CDS | 3078 |  |  |  | Hypothetical protein |
| SB021 contig 1 | LEJJMMNP_00706 | CDS | 990 |  |  |  | Hypothetical protein |
| SB021 contig 1 | LEJJMMNP_00707 | CDS | 1482 | *add* | 3.5.4.4 |  | Adenosine deaminase |
| SB021 contig 1 | LEJJMMNP_00708 | CDS | 594 | *recR* |  | COG0353 | Recombination protein RecR |
| SB021 contig 1 | LEJJMMNP_00709 | CDS | 306 |  |  |  | Hypothetical protein |
| SB021 contig 1 | LEJJMMNP_00710 | CDS | 90 |  |  |  | Hypothetical protein |
| SB021 contig 1 | LEJJMMNP_00711 | CDS | 1041 | *ftsY* |  | COG0552 | Signal recognition particle receptor FtsY |
| SB021 contig 1 | LEJJMMNP_00712 | CDS | 1014 | *eutD* |  | COG0280 | Ethanolamine utilization protein EutD |
| SB021 contig 1 | LEJJMMNP_00713 | CDS | 1203 | *ackA* | 2.7.2.1 | COG0282 | Acetate kinase |
| SB021 contig 1 | LEJJMMNP_00714 | CDS | 3567 |  | 1.2.7.1 | COG0674 | Pyruvate:ferredoxin oxidoreductase |
| SB021 contig 1 | LEJJMMNP_00715 | CDS | 1362 | *mepA_2* |  | COG0534 | Multidrug export protein MepA |
| SB021 contig 1 | LEJJMMNP_00716 | CDS | 462 |  |  |  | Hypothetical protein |
| SB021 contig 1 | LEJJMMNP_00717 | CDS | 2100 | *spoVD* |  | COG0768 | Stage V sporulation protein D |
| SB021 contig 1 | LEJJMMNP_00718 | CDS | 1044 | *priA_1* | 3.6.4.- | COG1198 | Primosomal protein N' |
| SB021 contig 1 | LEJJMMNP_00719 | CDS | 1158 | *priA_2* | 3.6.4.- | COG1198 | Primosomal protein N' |
| SB021 contig 1 | LEJJMMNP_00720 | CDS | 522 | *def* | 3.5.1.88 | COG0242 | Peptide deformylase |
| SB021 contig 1 | LEJJMMNP_00721 | CDS | 273 |  |  |  | Hypothetical protein |
| SB021 contig 1 | LEJJMMNP_00722 | CDS | 1050 | *glpX* | 3.1.3.11 | COG1494 | Fructose-1,6-bisphosphatase 1 class 2 |
| SB021 contig 1 | LEJJMMNP_00723 | CDS | 489 |  |  |  | Hypothetical protein |
| SB021 contig 1 | LEJJMMNP_00724 | CDS | 1038 | *ItaE* | 4.1.2.48 |  | Low specificity L-threonine aldolase |
| SB021 contig 1 | LEJJMMNP_00725 | CDS | 189 |  |  |  | Hypothetical protein |
| SB021 contig 1 | LEJJMMNP_00726 | CDS | 162 |  |  |  | Hypothetical protein |
| SB021 contig 1 | LEJJMMNP_00727 | CDS | 840 |  |  |  | Hypothetical protein |
| SB021 contig 1 | LEJJMMNP_00728 | CDS | 486 |  |  |  | Hypothetical protein |
| SB021 contig 1 | LEJJMMNP_00729 | CDS | 1170 | *bdhA* | 1.1.1.- | COG1979 | NADH-dependent butanol dehydrogenase A |
| SB021 contig 1 | LEJJMMNP_00730 | CDS | 543 | *yfcE* | 3.1.4.- | COG0622 | Phosphodiesterase YfcE |
| SB021 contig 1 | LEJJMMNP_00731 | tRNA | 77 |  |  |  | tRNA-Met(cat) |
| SB021 contig 1 | LEJJMMNP_00732 | tRNA | 77 |  |  |  | tRNA-Ala(tgc) |
| SB021 contig 1 | LEJJMMNP_00733 | tRNA | 77 |  |  |  | tRNA-Met(cat) |
| SB021 contig 1 | LEJJMMNP_00734 | tRNA | 77 |  |  |  | tRNA-Ala(tgc) |
| SB021 contig 1 | LEJJMMNP_00735 | tRNA | 77 |  |  |  | tRNA-Met(cat) |
| SB021 contig 1 | LEJJMMNP_00736 | tRNA | 77 |  |  |  | tRNA-Ala(tgc) |
| SB021 contig 1 | LEJJMMNP_00737 | CDS | 1164 | *csd* | 2.8.1.7 |  | Putative cysteine desulfurase |
| SB021 contig 1 | LEJJMMNP_00738 | CDS | 276 | *hup_1* |  |  | DNA-binding protein HU |
| SB021 contig 1 | LEJJMMNP_00739 | CDS | 2070 | *fusA_2* |  | COG0480 | Elongation factor G |
| SB021 contig 1 | LEJJMMNP_00740 | CDS | 186 |  |  |  | Hypothetical protein |
| SB021 contig 1 | LEJJMMNP_00741 | CDS | 1149 | *fucO* | 1.1.1.77 | COG1454 | Lactaldehyde reductase |
| SB021 contig 1 | LEJJMMNP_00742 | CDS | 1092 | *dnaN_1* |  |  | Beta sliding clamp |
| SB021 contig 1 | LEJJMMNP_00743 | CDS | 1080 | *potD* |  | COG0687 | Spermidine/putrescine-binding periplasmic protein |
| SB021 contig 1 | LEJJMMNP_00744 | CDS | 768 | *trmH* | 2.1.1.34 |  | tRNA (guanosine(18)-2'-O)-methyltransferase |
| SB021 contig 1 | LEJJMMNP_00745 | CDS | 159 |  |  |  | Rubredoxin |
| SB021 contig 1 | LEJJMMNP_00746 | CDS | 306 |  |  |  | Hypothetical protein |
| SB021 contig 1 | LEJJMMNP_00747 | CDS | 183 |  |  |  | Hypothetical protein |
| SB021 contig 1 | LEJJMMNP_00748 | CDS | 252 |  |  |  | Hypothetical protein |
| SB021 contig 1 | LEJJMMNP_00749 | CDS | 177 |  |  |  | Hypothetical protein |
| SB021 contig 1 | LEJJMMNP_00750 | CDS | 243 |  |  |  | Hypothetical protein |
| SB021 contig 1 | LEJJMMNP_00751 | CDS | 906 |  |  |  | Hypothetical protein |
| SB021 contig 1 | LEJJMMNP_00752 | CDS | 1326 |  |  |  | Hypothetical protein |
| SB021 contig 1 | LEJJMMNP_00753 | CDS | 201 |  |  |  | Hypothetical protein |
| SB021 contig 1 | LEJJMMNP_00754 | CDS | 678 | *nfo* | 3.1.21.2 | COG0648 | Endonuclease 4 |
| SB021 contig 1 | LEJJMMNP_00755 | CDS | 1260 | *esiB* |  |  | Secretory immunoglobulin A-binding protein EsiB |
| SB021 contig 1 | LEJJMMNP_00756 | CDS | 522 | *fbiB* | 6.3.2.34 |  | Bifunctional F420 biosynthesis protein FbiB |
| SB021 contig 1 | LEJJMMNP_00757 | CDS | 549 |  |  |  | Hypothetical protein |
| SB021 contig 1 | LEJJMMNP_00758 | CDS | 681 | *rsuA* | 5.4.99.19 | COG1187 | Ribosomal small subunit pseudouridine synthase A |
| SB021 contig 1 | LEJJMMNP_00759 | CDS | 876 | *xerC_1* |  | COG4974 | Tyrosine recombinase XerC |
| SB021 contig 1 | LEJJMMNP_00760 | CDS | 666 |  |  |  | Hypothetical protein |
| SB021 contig 1 | LEJJMMNP_00761 | CDS | 1197 |  |  |  | Hypothetical protein |
| SB021 contig 1 | LEJJMMNP_00762 | CDS | 756 |  |  |  | Hypothetical protein |
| SB021 contig 1 | LEJJMMNP_00763 | CDS | 762 |  |  |  | Hypothetical protein |
| SB021 contig 1 | LEJJMMNP_00764 | CDS | 831 | *nagB* | 3.5.99.6 | COG0363 | Glucosamine-6-phosphate deaminase |
| SB021 contig 1 | LEJJMMNP_00765 | CDS | 855 | *murR* |  | COG1737 | HTH-type transcriptional regulator MurR |
| SB021 contig 1 | LEJJMMNP_00766 | CDS | 930 |  |  |  | Hypothetical protein |
| SB021 contig 1 | LEJJMMNP_00767 | CDS | 177 |  |  |  | Hypothetical protein |
| SB021 contig 1 | LEJJMMNP_00768 | CDS | 645 | *queH* | 1.17.99.6 |  | Epoxyqueuosine reductase QueH |
| SB021 contig 1 | LEJJMMNP_00769 | CDS | 1158 |  |  |  | Hypothetical protein |
| SB021 contig 1 | LEJJMMNP_00770 | CDS | 483 |  |  |  | Hypothetical protein |
| SB021 contig 1 | LEJJMMNP_00771 | CDS | 2466 | *bepA* | 3.4.-.- |  | Beta-barrel assembly-enhancing protease |
| SB021 contig 1 | LEJJMMNP_00772 | CDS | 1071 | *ysdC* | 3.4.11.- | COG1363 | Putative aminopeptidase YsdC |
| SB021 contig 1 | LEJJMMNP_00773 | CDS | 435 |  |  |  | Hypothetical protein |
| SB021 contig 1 | LEJJMMNP_00774 | CDS | 498 | *msrC* | 1.8.4.14 | COG1956 | Free methionine-R-sulfoxide reductase |
| SB021 contig 1 | LEJJMMNP_00775 | CDS | 495 | *crr* |  | COG2190 | PTS system glucose-specific EIIA component |
| SB021 contig 1 | LEJJMMNP_00776 | CDS | 2571 | *clpB1* |  | COG0542 | Chaperone protein ClpB 1 |
| SB021 contig 1 | LEJJMMNP_00777 | CDS | 1614 |  |  |  | Hypothetical protein |
| SB021 contig 1 | LEJJMMNP_00778 | CDS | 858 |  |  |  | Hypothetical protein |
| SB021 contig 1 | LEJJMMNP_00779 | CDS | 558 | *rnmV* | 3.1.26.8 | COG1658 | Ribonuclease M5 |
| SB021 contig 1 | LEJJMMNP_00780 | CDS | 285 | *ycnE* | 1.-.-.- | COG1359 | Putative monooxygenase YcnE |
| SB021 contig 1 | LEJJMMNP_00781 | CDS | 1206 |  |  |  | Hypothetical protein |
| SB021 contig 1 | LEJJMMNP_00782 | CDS | 828 | *bmrR* |  | COG0789 | Multidrug-efflux transporter 1 regulator |
| SB021 contig 1 | LEJJMMNP_00783 | CDS | 444 | *sirC* | 1.3.1.76 |  | Precorrin-2 dehydrogenase |
| SB021 contig 1 | LEJJMMNP_00784 | CDS | 1353 | *mepA_3* |  |  | Multidrug export protein MepA |
| SB021 contig 1 | LEJJMMNP_00785 | CDS | 462 | *ribH* | 2.5.1.78 | COG0054 | 6,7-dimethyl-8-ribityllumazine synthase |
| SB021 contig 1 | LEJJMMNP_00786 | CDS | 1074 | *ribD* |  | COG0117 | Riboflavin biosynthesis protein RibD |
| SB021 contig 1 | LEJJMMNP_00787 | CDS | 633 | *ribE* | 2.5.1.9 | COG0307 | Riboflavin synthase |
| SB021 contig 1 | LEJJMMNP_00788 | CDS | 1227 | *ribBA* |  | COG0108 | Riboflavin biosynthesis protein RibBA |
| SB021 contig 1 | LEJJMMNP_00789 | CDS | 834 |  |  |  | Hypothetical protein |
| SB021 contig 1 | LEJJMMNP_00790 | CDS | 1251 | *glyA* | 2.1.2.1 | COG0112 | Serine hydroxymethyltransferase |
| SB021 contig 1 | LEJJMMNP_00791 | CDS | 714 | *cobB* | 2.3.1.286 | COG0846 | NAD-dependent protein deacetylase |
| SB021 contig 1 | LEJJMMNP_00792 | CDS | 1122 | *bcd_3* | 1.3.8.1 | COG1960 | Acyl-CoA dehydrogenase, short-chain specific |
| SB021 contig 1 | LEJJMMNP_00793 | CDS | 1305 | *pcnB* | 2.7.7.19 |  | Poly(A) polymerase I |
| SB021 contig 1 | LEJJMMNP_00794 | CDS | 2307 |  |  |  | Hypothetical protein |
| SB021 contig 1 | LEJJMMNP_00795 | CDS | 1728 |  | 3.6.3.- | COG1132 | Putative multidrug export ATP-binding/permease protein |
| SB021 contig 1 | LEJJMMNP_00796 | CDS | 714 | *sapB* |  | COG1285 | Protein SapB |
| SB021 contig 1 | LEJJMMNP_00797 | CDS | 1161 | *scmP* | 3.5.1.- | COG1473 | N-acetylcysteine deacetylase |
| SB021 contig 1 | LEJJMMNP_00798 | CDS | 1392 |  |  |  | Hypothetical protein |
| SB021 contig 1 | LEJJMMNP_00799 | CDS | 498 | *gltC_1* |  |  | HTH-type transcriptional regulator GltC |
| SB021 contig 1 | LEJJMMNP_00800 | CDS | 336 |  |  |  | Hypothetical protein |
| SB021 contig 1 | LEJJMMNP_00801 | CDS | 942 |  |  |  | Hypothetical protein |
| SB021 contig 1 | LEJJMMNP_00802 | CDS | 309 |  |  |  | Hypothetical protein |
| SB021 contig 1 | LEJJMMNP_00803 | CDS | 993 |  |  |  | Hypothetical protein |
| SB021 contig 1 | LEJJMMNP_00804 | CDS | 726 |  |  |  | Hypothetical protein |
| SB021 contig 1 | LEJJMMNP_00805 | CDS | 1296 |  |  |  | Hypothetical protein |
| SB021 contig 1 | LEJJMMNP_00806 | CDS | 123 |  |  |  | Hypothetical protein |
| SB021 contig 1 | LEJJMMNP_00807 | CDS | 2115 |  |  |  | IS1595 family transposase ISCco3 |
| SB021 contig 1 | LEJJMMNP_00808 | CDS | 900 |  |  |  | Hypothetical protein |
| SB021 contig 1 | LEJJMMNP_00809 | CDS | 417 |  |  |  | Hypothetical protein |
| SB021 contig 1 | LEJJMMNP_00810 | CDS | 618 |  |  |  | Hypothetical protein |
| SB021 contig 1 | LEJJMMNP_00811 | CDS | 627 |  |  |  | Hypothetical protein |
| SB021 contig 1 | LEJJMMNP_00812 | CDS | 195 |  |  |  | Hypothetical protein |
| SB021 contig 1 | LEJJMMNP_00813 | CDS | 1266 | *xerC_2* |  |  | Tyrosine recombinase XerC |
| SB021 contig 1 | LEJJMMNP_00814 | tRNA | 87 |  |  |  | tRNA-Leu(caa) |
| SB021 contig 1 | LEJJMMNP_00815 | CDS | 1128 | *alaS_1* | 6.1.1.7 | COG0013 | Alanine--tRNA ligase |
| SB021 contig 1 | LEJJMMNP_00816 | CDS | 1050 | *rlmN* | 2.1.1.192 |  | Putative dual-specificity RNA methyltransferase RlmN |
| SB021 contig 1 | LEJJMMNP_00817 | CDS | 2049 | *mtgA* | 2.4.1.129 |  | Biosynthetic peptidoglycan transglycosylase |
| SB021 contig 1 | LEJJMMNP_00818 | CDS | 327 | *ifhB* |  |  | Integration host factor subunit beta |
| SB021 contig 1 | LEJJMMNP_00819 | CDS | 990 | *fba* |  |  | Fructose-bisphosphate aldolase |
| SB021 contig 1 | LEJJMMNP_00820 | CDS | 468 |  |  |  | Hypothetical protein |
| SB021 contig 1 | LEJJMMNP_00821 | CDS | 1272 | *serS* | 6.1.1.11 | COG0172 | Serine—tRNA ligase |
| SB021 contig 1 | LEJJMMNP_00822 | CDS | 597 |  |  |  | Hypothetical protein |
| SB021 contig 1 | LEJJMMNP_00823 | CDS | 1005 | *lpxD* | 2.3.1.- | COG1044 | UDP-3-O-acylglucosamine N-acyltransferase |
| SB021 contig 1 | LEJJMMNP_00824 | CDS | 477 |  |  |  | Hypothetical protein |
| SB021 contig 1 | LEJJMMNP_00825 | CDS | 2079 | *bamA* |  |  | Outer membrane protein assembly factor BamA |
| SB021 contig 1 | LEJJMMNP_00826 | CDS | 4362 |  |  |  | Hypothetical protein |
| SB021 contig 1 | LEJJMMNP_00827 | CDS | 702 | *rsmG* | 2.1.1.- | COG0357 | Ribosomal RNA small subunit methyltransferase G |
| SB021 contig 1 | LEJJMMNP_00828 | CDS | 1890 | *mnmG_1* |  |  | tRNA uridine 5-carboxymethylaminomethyl modification enzyme MnmG |
| SB021 contig 1 | LEJJMMNP_00829 | CDS | 432 | *ktrA* |  | COG0569 | Ktr system potassium uptake protein A |
| SB021 contig 1 | LEJJMMNP_00830 | CDS | 90 |  |  |  | Hypothetical protein |
| SB021 contig 1 | LEJJMMNP_00831 | CDS | 1338 | *ktrB* |  | COG0168 | Ktr system potassium uptake protein B |
| SB021 contig 1 | LEJJMMNP_00832 | CDS | 1179 |  |  |  | Hypothetical protein |
| SB021 contig 1 | LEJJMMNP_00833 | CDS | 624 | *udk_2* | 2.7.1.48 | COG0572 | Uridine kinase |
| SB021 contig 1 | LEJJMMNP_00834 | CDS | 1044 | *adh* | 1.1.1.1 | COG1063 | Alcohol dehydrogenase |
| SB021 contig 1 | LEJJMMNP_00835 | CDS | 624 | *sgcG* | 1.3.99.24 |  | 2-amino-4-deoxychorismate dehydrogenase |
| SB021 contig 1 | LEJJMMNP_00836 | CDS | 1941 |  |  |  | Hypothetical protein |
| SB021 contig 1 | LEJJMMNP_00837 | CDS | 132 |  |  |  | Hypothetical protein |
| SB021 contig 1 | LEJJMMNP_00838 | CDS | 1251 |  |  |  | Hypothetical protein |
| SB021 contig 1 | LEJJMMNP_00839 | CDS | 1338 | *dnaC* | 3.6.4.12 | COG0305 | Replicative DNA helicase |
| SB021 contig 1 | LEJJMMNP_00840 | CDS | 447 | *rplI* |  |  | 50S ribosomal protein L9 |
| SB021 contig 1 | LEJJMMNP_00841 | CDS | 900 |  |  |  | Hypothetical protein |
| SB021 contig 1 | LEJJMMNP_00842 | CDS | 1476 |  |  |  | Hypothetical protein |
| SB021 contig 1 | LEJJMMNP_00843 | CDS | 1395 | *glnG* |  | COG2204 | DNA-binding transcriptional regulator NtrC |
| SB021 contig 1 | LEJJMMNP_00844 | CDS | 561 |  |  |  | Hypothetical protein |
| SB021 contig 1 | LEJJMMNP_00845 | CDS | 273 |  |  |  | Hypothetical protein |
| SB021 contig 1 | LEJJMMNP_00846 | CDS | 438 | *tolR* |  |  | Tol-Pal system protein TolR |
| SB021 contig 1 | LEJJMMNP_00847 | CDS | 612 | *tolQ* |  |  | Tol-Pal system protein TolQ |
| SB021 contig 1 | LEJJMMNP_00848 | CDS | 339 |  |  |  | Hypothetical protein |
| SB021 contig 1 | LEJJMMNP_00849 | CDS | 2898 | *cpoB_1* |  |  | Cell division coordinator CpoB |
| SB021 contig 1 | LEJJMMNP_00850 | CDS | 576 |  |  |  | Hypothetical protein |
| SB021 contig 1 | LEJJMMNP_00851 | CDS | 1128 | *dnaN_2* |  | COG0592 | Beta sliding clamp |
| SB021 contig 1 | LEJJMMNP_00852 | CDS | 828 | *sigA_2* |  | COG0568 | RNA polymerase sigma factor SigA |
| SB021 contig 1 | LEJJMMNP_00853 | CDS | 1008 | *gpsA* | 1.1.1.94 |  | Glycerol-3-phosphate dehydrogenase [NAD(P)+] |
| SB021 contig 1 | LEJJMMNP_00854 | CDS | 615 | *plsY* | 2.3.1.275 | COG0344 | Glycerol-3-phosphate acyltransferase |
| SB021 contig 1 | LEJJMMNP_00855 | CDS | 147 |  |  |  | Hypothetical protein |
| SB021 contig 1 | LEJJMMNP_00856 | CDS | 1137 | *braC_2* |  | COG0683 | Leucine-, isoleucine-, valine-, threonine-, and alanine-binding protein |
| SB021 contig 1 | LEJJMMNP_00857 | CDS | 1335 |  |  |  | Hypothetical protein |
| SB021 contig 1 | LEJJMMNP_00858 | CDS | 1272 | *ydiN* |  |  | Inner membrane transport protein YdiN |
| SB021 contig 1 | LEJJMMNP_00859 | CDS | 894 | *ydiB* | 1.1.1.282 | COG0169 | Quinate/shikimate dehydrogenase |
| SB021 contig 1 | LEJJMMNP_00860 | CDS | 1926 | *baiH* | 1.3.1.116 | COG0446 | 7-beta-hydroxy-3-oxochol-24-oyl-CoA 4-desaturase |
| SB021 contig 1 | LEJJMMNP_00861 | CDS | 846 |  |  |  | Hypothetical protein |
| SB021 contig 1 | LEJJMMNP_00862 | CDS | 894 | *gltC_2* |  |  | HTH-type transcriptional regulator GltC |
| SB021 contig 1 | LEJJMMNP_00863 | CDS | 873 | *gltC_3* |  |  | HTH-type transcriptional regulator GltC |
| SB021 contig 1 | LEJJMMNP_00864 | CDS | 765 | *aroD* | 4.2.1.10 | COG0710 | 3-dehydroquinate dehydratase |
| SB021 contig 1 | LEJJMMNP_00865 | CDS | 873 | *aroE* | 1.1.1.25 | COG0169 | Shikimate dehydrogenase (NADP(+)) |
| SB021 contig 1 | LEJJMMNP_00866 | CDS | 1149 |  | 1.-.-.- |  | NADH oxidase |
| SB021 contig 1 | LEJJMMNP_00867 | CDS | 744 |  | 1.-.-.- |  | NADH oxidase |
| SB021 contig 1 | LEJJMMNP_00868 | CDS | 1020 |  | 3.5.1.47 |  | N-acetyldiaminopimelate deacetylase |
| SB021 contig 1 | LEJJMMNP_00869 | CDS | 1401 |  |  |  | Hypothetical protein |
| SB021 contig 1 | LEJJMMNP_00870 | CDS | 444 | *elaA* |  |  | Protein ElaA |
| SB021 contig 1 | LEJJMMNP_00871 | CDS | 783 | *ydfG* | 1.1.1.381 | COG4221 | NADP-dependent 3-hydroxy acid dehydrogenase YdfG |
| SB021 contig 1 | LEJJMMNP_00872 | CDS | 564 | *aroK* | 2.7.1.71 | COG0703 | Shikimate kinase 1 |
| SB021 contig 1 | LEJJMMNP_00873 | CDS | 1353 |  |  |  | Hypothetical protein |
| SB021 contig 1 | LEJJMMNP_00874 | CDS | 1161 | *tgt* | 2.4.2.29 |  | Queuine tRNA-ribosyltransferase |
| SB021 contig 1 | LEJJMMNP_00875 | CDS | 2178 | *relA* | 2.7.6.5 | COG0317 | GTP pyrophosphokinase |
| SB021 contig 1 | LEJJMMNP_00876 | CDS | 516 | *apt* | 2.4.2.7 | COG0503 | Adenine phosphoribosyltransferase |
| SB021 contig 1 | LEJJMMNP_00877 | CDS | 858 |  |  |  | Hypothetical protein |
| SB021 contig 1 | LEJJMMNP_00878 | CDS | 663 |  |  |  | Hypothetical protein |
| SB021 contig 1 | LEJJMMNP_00879 | CDS | 1302 |  |  |  | Hypothetical protein |
| SB021 contig 1 | LEJJMMNP_00880 | CDS | 438 |  |  |  | Hypothetical protein |
| SB021 contig 1 | LEJJMMNP_00881 | CDS | 456 | *accB* |  | COG0511 | Biotin carboxyl carrier protein of acetyl-CoA carboxylase |
| SB021 contig 1 | LEJJMMNP_00882 | CDS | 474 |  |  |  | Hypothetical protein |
| SB021 contig 1 | LEJJMMNP_00883 | CDS | 858 | *fold* |  | COG0190 | Bifunctional protein FolD protein |
| SB021 contig 1 | LEJJMMNP_00884 | CDS | 708 | *fmt_1* | 2.1.2.9 | COG0223 | Methionyl-tRNA formyltransferase |
| SB021 contig 1 | LEJJMMNP_00885 | CDS | 126 | *fmt_2* | 2.1.2.9 |  | Methionyl-tRNA formyltransferase |
| SB021 contig 1 | LEJJMMNP_00886 | CDS | 459 | *nrdR* |  | COG1327 | Transcriptional repressor NrdR |
| SB021 contig 1 | LEJJMMNP_00887 | CDS | 471 | *fruA* |  | COG1299 | PTS system fructose-specific EIIABC component |
| SB021 contig 1 | LEJJMMNP_00888 | CDS | 708 | *recO* |  |  | DNA repair protein RecO |
| SB021 contig 1 | LEJJMMNP_00889 | CDS | 558 |  |  |  | Hypothetical protein |
| SB021 contig 1 | LEJJMMNP_00890 | CDS | 804 |  |  |  | Hypothetical protein |
| SB021 contig 1 | LEJJMMNP_00891 | CDS | 861 | *cntI* |  |  | Pseudopaline exporter CntI |
| SB021 contig 1 | LEJJMMNP_00892 | CDS | 1011 |  |  |  | Hypothetical protein |
| SB021 contig 1 | LEJJMMNP_00893 | CDS | 1449 | *patA* | 2.3.1.- | COG1696 | Peptidoglycan O-acetyltransferase |
| SB021 contig 1 | LEJJMMNP_00894 | CDS | 1194 | *rffG_2* | 4.2.1.46 | COG1088 | dTDP-glucose 4,6-dehydratase 2 |
| SB021 contig 1 | LEJJMMNP_00895 | CDS | 1050 |  |  |  | Hypothetical protein |
| SB021 contig 1 | LEJJMMNP_00896 | CDS | 1818 | *hisC_2* | 2.6.1.9 |  | Histidinol-phosphate aminotransferase |
| SB021 contig 1 | LEJJMMNP_00897 | CDS | 1467 |  |  |  | Hypothetical protein |
| SB021 contig 1 | LEJJMMNP_00898 | CDS | 555 |  |  |  | Hypothetical protein |
| SB021 contig 1 | LEJJMMNP_00899 | CDS | 837 | *rfbD_2* | 1.1.1.133 | COG1091 | dTDP-4-dehydrorhamnose reductase |
| SB021 contig 1 | LEJJMMNP_00900 | CDS | 576 | *rfbC_2* | 5.1.3.13 | COG1898 | dTDP-4-dehydrorhamnose 3,5-epimerase |
| SB021 contig 1 | LEJJMMNP_00901 | CDS | 870 | *rfbA_2* | 2.7.7.24 | COG1209 | Glucose-1-phosphate thymidylyltransferase 1 |
| SB021 contig 1 | LEJJMMNP_00902 | CDS | 1236 |  |  |  | Hypothetical protein |
| SB021 contig 1 | LEJJMMNP_00903 | CDS | 843 |  |  |  | Hypothetical protein |
| SB021 contig 1 | LEJJMMNP_00904 | CDS | 1098 |  |  |  | Hypothetical protein |
| SB021 contig 1 | LEJJMMNP_00905 | CDS | 555 | *dapH_2* | 2.3.1.89 |  | 2,3,4,5-tetrahydropyridine-2,6-dicarboxylate N-acetyltransferase |
| SB021 contig 1 | LEJJMMNP_00906 | CDS | 1002 |  |  |  | Hypothetical protein |
| SB021 contig 1 | LEJJMMNP_00907 | CDS | 1131 | *wbiB* | 5.1.3.25 |  | dTDP-L-rhamnose 4-epimerase |
| SB021 contig 1 | LEJJMMNP_00908 | CDS | 1362 |  |  |  | Hypothetical protein |
| SB021 contig 1 | LEJJMMNP_00909 | CDS | 1188 |  |  |  | Hypothetical protein |
| SB021 contig 1 | LEJJMMNP_00910 | CDS | 978 | *thiY_1* |  | COG0715 | Formylaminopyrimidine-binding protein |
| SB021 contig 1 | LEJJMMNP_00911 | CDS | 1023 | *thiY_2* |  | COG0715 | Formylaminopyrimidine-binding protein |
| SB021 contig 1 | LEJJMMNP_00912 | CDS | 741 | *ribX* |  |  | Riboflavin transport system permease protein RibX |
| SB021 contig 1 | LEJJMMNP_00913 | CDS | 708 | *cmpD* | 3.6.3.- |  | Bicarbonate transport ATP-binding protein CmpD |
| SB021 contig 1 | LEJJMMNP_00914 | CDS | 657 | *tenA_2* | 3.5.99.2 | COG0819 | Aminopyrimidine aminohydrolase |
| SB021 contig 1 | LEJJMMNP_00915 | CDS | 1224 | *sbcD* |  | COG0420 | Nuclease SbcCD subunit D |
| SB021 contig 1 | LEJJMMNP_00916 | CDS | 3006 | *sbcC* |  |  | Nuclease SbcCD subunit C |
| SB021 contig 1 | LEJJMMNP_00917 | CDS | 702 |  |  |  | Hypothetical protein |
| SB021 contig 1 | LEJJMMNP_00918 | CDS | 1821 | *glmS_2* | 2.6.1.16 | COG0449 | Glutamine--fructose-6-phosphate aminotransferase [isomerizing] |
| SB021 contig 1 | LEJJMMNP_00919 | CDS | 2082 |  |  |  | Hypothetical protein |
| SB021 contig 1 | LEJJMMNP_00920 | CDS | 696 |  |  |  | Hypothetical protein |
| SB021 contig 1 | LEJJMMNP_00921 | CDS | 1080 | *mnmA* | 2.8.1.13 |  | tRNA-specific 2-thiouridylase MnmA |
| SB021 contig 1 | LEJJMMNP_00922 | CDS | 615 |  |  |  | Hypothetical protein |
| SB021 contig 1 | LEJJMMNP_00923 | CDS | 1806 | *lepA* | 3.6.5.- | COG0481 | Elongation factor 4 |
| SB021 contig 1 | LEJJMMNP_00924 | tRNA | 77 |  |  |  | tRNA-Arg(cct) |
| SB021 contig 1 | LEJJMMNP_00925 | CDS | 747 |  |  |  | Hypothetical protein |
| SB021 contig 1 | LEJJMMNP_00926 | CDS | 1455 | *clsA* | 2.7.8.- | COG1502 | Major cardiolipin synthase ClsA |
| SB021 contig 1 | LEJJMMNP_00927 | CDS | 834 |  |  |  | Hypothetical protein |
| SB021 contig 1 | LEJJMMNP_00928 | CDS | 1293 |  |  |  | Hypothetical protein |
| SB021 contig 1 | LEJJMMNP_00929 | CDS | 771 |  |  |  | Hypothetical protein |
| SB021 contig 1 | LEJJMMNP_00930 | CDS | 606 | *glpE* | 2.8.1.1 |  | Thiosulfate sulfurtransferase GlpE |
| SB021 contig 1 | LEJJMMNP_00931 | CDS | 1695 | *cdr* | 1.8.1.14 |  | Coenzyme A disulfide reductase |
| SB021 contig 1 | LEJJMMNP_00932 | CDS | 816 | *yigL* | 3.1.3.74 | COG0561 | Pyridoxal phosphate phosphatase YigL |
| SB021 contig 1 | LEJJMMNP_00933 | tRNA | 75 |  |  |  | tRNA-Gln(ttg) |
| SB021 contig 1 | LEJJMMNP_00934 | CDS | 1194 |  |  |  | Hypothetical protein |
| SB021 contig 1 | LEJJMMNP_00935 | CDS | 2070 | *glyS* | 6.1.1.14 | COG0751 | Glycine—tRNA ligase beta subunit |
| SB021 contig 1 | LEJJMMNP_00936 | CDS | 870 | *glyQ* | 6.1.1.14 | COG0752 | Glycine—tRNA ligase alpha subunit |
| SB021 contig 1 | LEJJMMNP_00937 | CDS | 453 | *lspA* | 3.4.23.36 | COG0597 | Lipoprotein signal peptidase |
| SB021 contig 1 | LEJJMMNP_00938 | CDS | 2418 | *sasA_4* | 2.7.-.- |  | Adaptive-response sensory-kinase SasA |
| SB021 contig 1 | LEJJMMNP_00939 | CDS | 2166 | *yhgF* |  | COG2183 | Protein YhgF |
| SB021 contig 1 | LEJJMMNP_00940 | CDS | 3114 | *mupB* |  |  | Mupirocin-resistant isoleucine—tRNA ligase MupB |
| SB021 contig 1 | LEJJMMNP_00941 | CDS | 264 | *rpsP* |  | COG0228 | 30S ribosomal protein S16 |
| SB021 contig 1 | LEJJMMNP_00942 | CDS | 1377 | *ffh* |  | COG0541 | Signal recognition particle protein |
| SB021 contig 1 | LEJJMMNP_00943 | CDS | 297 |  |  |  | Hypothetical protein |
| SB021 contig 1 | LEJJMMNP_00944 | CDS | 666 | *nudL* | 3.6.1.- |  | Putative Nudix hydrolase NudL |
| SB021 contig 1 | LEJJMMNP_00945 | CDS | 558 |  |  |  | Hypothetical protein |
| SB021 contig 1 | LEJJMMNP_00946 | CDS | 1278 | *purA* | 6.3.4.4 | COG0104 | Adenylosuccinate synthetase |
| SB021 contig 1 | LEJJMMNP_00947 | CDS | 702 | *trmB* | 2.1.1.33 |  | tRNA (guanine-N(7)-)-methyltransferase |
| SB021 contig 1 | LEJJMMNP_00948 | CDS | 1230 |  |  |  | Hypothetical protein |
| SB021 contig 1 | LEJJMMNP_00949 | CDS | 663 | *cmk* | 2.7.4.25 | COG0283 | Cytidylate kinase |
| SB021 contig 1 | LEJJMMNP_00950 | CDS | 930 | *prmC_1* | 2.1.1.297 |  | Release factor glutamine methyltransferase |
| SB021 contig 1 | LEJJMMNP_00951 | CDS | 795 | *ymdB* | 3.1.4.16 | COG1692 | 2',3'-cyclic-nucleotide 2'-phosphodiesterase |
| SB021 contig 1 | LEJJMMNP_00952 | CDS | 663 |  |  |  | Hypothetical protein |
| SB021 contig 1 | LEJJMMNP_00953 | CDS | 639 |  |  |  | Hypothetical protein |
| SB021 contig 1 | LEJJMMNP_00954 | CDS | 1134 | *rlmL* | 2.1.1.173 | COG0116 | Ribosomal RNA large subunit methyltransferase L |
| SB021 contig 1 | LEJJMMNP_00955 | CDS | 1113 | *yhhT* |  | COG0628 | Putative transport protein YhhT |
| SB021 contig 1 | LEJJMMNP_00956 | CDS | 396 |  |  |  | Hypothetical protein |
| SB021 contig 1 | LEJJMMNP_00957 | CDS | 900 | *psd* | 4.1.1.65 | COG0688 | Phosphatidylserine decarboxylase proenzyme |
| SB021 contig 1 | LEJJMMNP_00958 | CDS | 387 | *mgsA* | 4.2.3.3 | COG1803 | Methylglyoxal synthase |
| SB021 contig 1 | LEJJMMNP_00959 | CDS | 918 |  |  |  | Hypothetical protein |
| SB021 contig 1 | LEJJMMNP_00960 | CDS | 1098 | *ppaT* | 2.6.1.30 | COG0075 | Pyridoxamine—pyruvate transaminase |
| SB021 contig 1 | LEJJMMNP_00961 | CDS | 564 | *efp* |  | COG0231 | Elongation factor P |
| SB021 contig 1 | LEJJMMNP_00962 | CDS | 1164 |  |  |  | Hypothetical protein |
| SB021 contig 1 | LEJJMMNP_00963 | CDS | 969 |  | 1.-.-.- |  | NADH oxidase |
| SB021 contig 1 | LEJJMMNP_00964 | CDS | 321 |  |  |  | Hypothetical protein |
| SB021 contig 1 | LEJJMMNP_00965 | CDS | 1614 | *ybiT* |  | COG0488 | Putative ABC transporter ATP-binding protein YbiT |
| SB021 contig 1 | LEJJMMNP_00966 | CDS | 1092 |  |  |  | Hypothetical protein |
| SB021 contig 1 | LEJJMMNP_00967 | CDS | 1374 | *rlmCD* | 2.1.1.189 | COG2265 | 23S rRNA (uracil-C(5))-methyltransferase RlmCD |
| SB021 contig 1 | LEJJMMNP_00968 | CDS | 198 |  |  |  | Hypothetical protein |
| SB021 contig 1 | LEJJMMNP_00969 | CDS | 963 | *rsmH* | 2.1.1.199 |  | Ribosomal RNA small subunit methyltransferase H |
| SB021 contig 1 | LEJJMMNP_00970 | CDS | 258 |  |  |  | Hypothetical protein |
| SB021 contig 1 | LEJJMMNP_00971 | CDS | 564 | *pgsA* | 2.7.8.5 |  | CDP-diacylglycerol--glycerol-3-phosphate 3-phosphatidyltransferase |
| SB021 contig 1 | LEJJMMNP_00972 | CDS | 1338 | *rimO* | 2.8.4.4 | COG0621 | Ribosomal protein S12 methylthiotransferase RimO |
| SB021 contig 1 | LEJJMMNP_00973 | CDS | 2112 | *pnp* | 2.7.7.8 | COG1185 | Polyribonucleotide nucleotidyltransferase |
| SB021 contig 1 | LEJJMMNP_00974 | CDS | 1947 | *fbp* | 3.1.3.11 |  | Fructose-1,6-bisphosphatase class 3 |
| SB021 contig 1 | LEJJMMNP_00975 | CDS | 1566 | *rny* | 3.1.-.- | COG1418 | Ribonuclease Y |
| SB021 contig 1 | LEJJMMNP_00976 | CDS | 354 | *rsbV* |  | COG1366 | Anti-sigma-B factor antagonist |
| SB021 contig 1 | LEJJMMNP_00977 | CDS | 396 | *spoIIAB* | 2.7.11.1 |  | Anti-sigma F factor |
| SB021 contig 1 | LEJJMMNP_00978 | CDS | 510 |  |  |  | Hypothetical protein |
| SB021 contig 1 | LEJJMMNP_00979 | CDS | 924 | *miaA* | 2.5.1.75 | COG0324 | tRNA dimethylallyltransferase |
| SB021 contig 1 | LEJJMMNP_00980 | CDS | 1287 | *obg* | 3.6.5.- |  | GTPase Obg |
| SB021 contig 1 | LEJJMMNP_00981 | CDS | 549 | *hpf* |  | COG1544 | Ribosome hibernation promotion factor |
| SB021 contig 1 | LEJJMMNP_00982 | CDS | 684 | *lolD* | 3.6.3.- | COG1136 | Lipoprotein-releasing system ATP-binding protein LolD |
| SB021 contig 1 | LEJJMMNP_00983 | CDS | 1167 | *lolC* |  | COG4591 | Lipoprotein-releasing system transmembrane protein LolC |
| SB021 contig 1 | LEJJMMNP_00984 | CDS | 615 |  |  |  | Hypothetical protein |
| SB021 contig 1 | LEJJMMNP_00985 | CDS | 2766 |  |  |  | Hypothetical protein |
| SB021 contig 1 | LEJJMMNP_00986 | CDS | 1629 |  |  |  | Hypothetical protein |
| SB021 contig 1 | LEJJMMNP_00987 | CDS | 858 | *truB* | 5.4.99.25 |  | tRNA pseudouridine synthase B |
| SB021 contig 1 | LEJJMMNP_00988 | CDS | 1812 | *typA* |  | COG1217 | GTP-binding protein TypA/BipA |
| SB021 contig 1 | LEJJMMNP_00989 | CDS | 267 | *ptsH_1* |  |  | Phosphocarrier protein HPr |
| SB021 contig 1 | LEJJMMNP_00990 | CDS | 1731 | *ptsI_1* | 2.7.3.9 | COG1080 | Phosphoenolpyruvate-protein phosphotransferase |
| SB021 contig 1 | LEJJMMNP_00991 | CDS | 1500 | *cobQ_1* |  |  | Cobyric acid synthase |
| SB021 contig 1 | LEJJMMNP_00992 | CDS | 216 |  |  |  | Hypothetical protein |
| SB021 contig 1 | LEJJMMNP_00993 | CDS | 678 | *lexA* | 3.4.21.88 |  | LexA repressor |
| SB021 contig 1 | LEJJMMNP_00994 | CDS | 423 |  |  |  | Hypothetical protein |
| SB021 contig 1 | LEJJMMNP_00995 | CDS | 741 |  |  |  | Hypothetical protein |
| SB021 contig 1 | LEJJMMNP_00996 | CDS | 2820 | *rep* | 3.6.4.12 |  | ATP-dependent DNA helicase Rep |
| SB021 contig 1 | LEJJMMNP_00997 | CDS | 549 |  |  |  | Hypothetical protein |
| SB021 contig 1 | LEJJMMNP_00998 | CDS | 555 |  |  |  | Hypothetical protein |
| SB021 contig 1 | LEJJMMNP_00999 | CDS | 174 | *fdxA* |  |  | 4Fe-4S ferredoxin FdxA |
| SB021 contig 1 | LEJJMMNP_01000 | CDS | 597 |  |  |  | Hypothetical protein |
| SB021 contig 1 | LEJJMMNP_01001 | CDS | 1917 | *recQ* | 3.6.4.12 | COG0514 | ATP-dependent DNA helicase RecQ |
| SB021 contig 1 | LEJJMMNP_01002 | CDS | 1041 | *selU* | 2.5.1.- | COG2603 | tRNA 2-selenouridine synthase |
| SB021 contig 1 | LEJJMMNP_01003 | CDS | 603 | *nrdZ* | 1.17.4.1 |  | Vitamin B12-dependent ribonucleoside-diphosphate reductase |
| SB021 contig 1 | LEJJMMNP_01004 | CDS | 1671 |  |  |  | Hypothetical protein |
| SB021 contig 1 | LEJJMMNP_01005 | CDS | 744 | *artP* |  | COG0834 | Arginine-binding extracellular protein ArtP |
| SB021 contig 1 | LEJJMMNP_01006 | CDS | 1179 | *abgB* | 3.5.1.- | COG1473 | p-aminobenzoyl-glutamate hydrolase subunit B |
| SB021 contig 1 | LEJJMMNP_01007 | CDS | 666 |  |  |  | Hypothetical protein |
| SB021 contig 1 | LEJJMMNP_01008 | CDS | 567 |  |  |  | Hypothetical protein |
| SB021 contig 1 | LEJJMMNP_01009 | CDS | 1125 | *bioF* | 2.3.1.47 | COG0156 | 8-amino-7-oxononanoate synthase |
| SB021 contig 1 | LEJJMMNP_01010 | CDS | 1377 | *bioA* | 2.6.1.62 |  | Adenosylmethionine-8-amino-7-oxononanoate aminotransferase |
| SB021 contig 1 | LEJJMMNP_01011 | CDS | 681 | *bioD1* | 6.3.3.3 | COG0132 | ATP-dependent dethiobiotin synthetase BioD 1 |
| SB021 contig 1 | LEJJMMNP_01012 | CDS | 345 |  |  |  | Hypothetical protein |
| SB021 contig 1 | LEJJMMNP_01013 | CDS | 1260 |  | 1.18.1.2 |  | Ferredoxin--NADP reductase |
| SB021 contig 1 | LEJJMMNP_01014 | CDS | 1434 | *lhgD* | 1.1.5.- |  | L-2-hydroxyglutarate dehydrogenase |
| SB021 contig 1 | LEJJMMNP_01015 | CDS | 1506 | *glpK* | 2.7.1.30 | COG0554 | Glycerol kinase |
| SB021 contig 1 | LEJJMMNP_01016 | CDS | 738 | *glpF* |  | COG0580 | Glycerol uptake facilitator protein |
| SB021 contig 1 | LEJJMMNP_01017 | CDS | 690 |  |  |  | Hypothetical protein |
| SB021 contig 1 | LEJJMMNP_01018 | CDS | 735 | *glpQ* | 3.1.4.46 | COG0584 | Glycerophosphodiester phosphodiesterase |
| SB021 contig 1 | LEJJMMNP_01019 | CDS | 561 | *ygcP* |  | COG1954 | Putative protein YgcP |
| SB021 contig 1 | LEJJMMNP_01020 | CDS | 1149 | *msmX* | 7.5.2.- | COG3839 | Oligosaccharides import ATP-binding protein MsmX |
| SB021 contig 1 | LEJJMMNP_01021 | CDS | 1302 | *ugpB* |  | COG1653 | sn-glycerol-3-phosphate-binding periplasmic protein UgpB |
| SB021 contig 1 | LEJJMMNP_01022 | CDS | 813 | *lacG* |  |  | Lactose transport system permease protein LacG |
| SB021 contig 1 | LEJJMMNP_01023 | CDS | 873 | *ugpA* |  | COG1175 | sn-glycerol-3-phosphate transport system permease protein UgpA |
| SB021 contig 1 | LEJJMMNP_01024 | CDS | 852 | *licT* |  | COG3711 | Transcription antiterminator LicT |
| SB021 contig 1 | LEJJMMNP_01025 | CDS | 1482 | *bglfF_1* |  | COG1263 | PTS system beta-glucoside-specific EIIBCA component |
| SB021 contig 1 | LEJJMMNP_01026 | CDS | 480 | *bglF_2* |  | COG1263 | PTS system beta-glucoside-specific EIIBCA component |
| SB021 contig 1 | LEJJMMNP_01027 | CDS | 1428 | *bglH* | 3.2.1.86 | COG2723 | Aryl-phospho-beta-D-glucosidase BglH |
| SB021 contig 1 | LEJJMMNP_01028 | CDS | 1419 | *alsT_2* |  | COG1115 | Amino-acid carrier protein AlsT |
| SB021 contig 1 | LEJJMMNP_01029 | CDS | 897 | *hslO* |  |  | 33 kDa chaperonin |
| SB021 contig 1 | LEJJMMNP_01030 | CDS | 492 |  |  |  | Hypothetical protein |
| SB021 contig 1 | LEJJMMNP_01031 | CDS | 960 | *hemZ* | 1.3.99.- | COG0635 | Oxygen-independent coproporphyrinogen-III oxidase-like protein HemZ |
| SB021 contig 1 | LEJJMMNP_01032 | CDS | 435 |  |  |  | Hypothetical protein |
| SB021 contig 1 | LEJJMMNP_01033 | CDS | 1047 | *pilT* |  | COG2805 | Twitching mobility protein |
| SB021 contig 1 | LEJJMMNP_01034 | CDS | 804 | *uppP* | 3.6.1.27 |  | Undecaprenyl-diphosphatase |
| SB021 contig 1 | LEJJMMNP_01035 | CDS | 999 | *hldD* | 5.1.3.20 |  | ADP-L-glycero-D-manno-heptose-6-epimerase |
| SB021 contig 1 | LEJJMMNP_01036 | CDS | 855 | *yciV* | 3.1.13.- | COG0613 | 5'-3' exoribonuclease |
| SB021 contig 1 | LEJJMMNP_01037 | CDS | 720 |  |  |  | Hypothetical protein |
| SB021 contig 1 | LEJJMMNP_01038 | CDS | 2610 | *secA* |  | COG0653 | Protein translocase subunit SecA |
| SB021 contig 1 | LEJJMMNP_01039 | CDS | 2019 | *ligA* | 6.5.1.2 |  | DNA ligase |
| SB021 contig 1 | LEJJMMNP_01040 | CDS | 1563 |  |  |  | Hypothetical protein |
| SB021 contig 1 | LEJJMMNP_01041 | CDS | 1461 | *murJ_2* |  | COG0728 | Putative lipid II flippase MurJ |
| SB021 contig 1 | LEJJMMNP_01042 | CDS | 747 | *scpA* |  |  | Segregation and condensation protein A |
| SB021 contig 1 | LEJJMMNP_01043 | CDS | 933 | *ribF* |  |  | Bifunctional riboflavin kinase/FMN adenylyltransferase |
| SB021 contig 1 | LEJJMMNP_01044 | CDS | 912 | *whiA* |  | COG1481 | Putative cell division protein WhiA |
| SB021 contig 1 | LEJJMMNP_01045 | CDS | 2733 | *polA* | 2.7.7.7 |  | DNA polymerase I |
| SB021 contig 1 | LEJJMMNP_01046 | CDS | 426 |  |  |  | Single-stranded DNA-binding protein |
| SB021 contig 1 | LEJJMMNP_01047 | CDS | 1539 | *guaA* | 6.3.5.2 |  | GMP synthase [glutamine-hydrolyzing] |
| SB021 contig 1 | LEJJMMNP_01048 | CDS | 1083 | *xerC_3* |  |  | Tyrosine recombinase XerC |
| SB021 contig 1 | LEJJMMNP_01049 | CDS | 123 |  |  |  | Hypothetical protein |
| SB021 contig 1 | LEJJMMNP_01050 | CDS | 1851 |  |  |  | Hypothetical protein |
| SB021 contig 1 | LEJJMMNP_01051 | CDS | 606 |  |  |  | Hypothetical protein |
| SB021 contig 1 | LEJJMMNP_01052 | CDS | 90 |  |  |  | Hypothetical protein |
| SB021 contig 1 | LEJJMMNP_01053 | CDS | 1803 |  |  |  | Hypothetical protein |
| SB021 contig 1 | LEJJMMNP_01054 | CDS | 504 |  |  |  | Hypothetical protein |
| SB021 contig 1 | LEJJMMNP_01055 | CDS | 114 |  |  |  | Hypothetical protein |
| SB021 contig 1 | LEJJMMNP_01056 | CDS | 840 |  |  |  | Hypothetical protein |
| SB021 contig 1 | LEJJMMNP_01057 | CDS | 294 |  |  |  | Hypothetical protein |
| SB021 contig 1 | LEJJMMNP_01058 | CDS | 636 |  |  |  | Hypothetical protein |
| SB021 contig 1 | LEJJMMNP_01059 | CDS | 276 |  |  |  | Hypothetical protein |
| SB021 contig 1 | LEJJMMNP_01060 | CDS | 516 |  |  |  | Hypothetical protein |
| SB021 contig 1 | LEJJMMNP_01061 | CDS | 102 |  |  |  | Hypothetical protein |
| SB021 contig 1 | LEJJMMNP_01062 | CDS | 360 |  |  |  | Hypothetical protein |
| SB021 contig 1 | LEJJMMNP_01063 | CDS | 231 |  |  |  | Hypothetical protein |
| SB021 contig 1 | LEJJMMNP_01064 | CDS | 912 | *htpX* | 3.4.24.- |  | Protease HtpX |
| SB021 contig 1 | LEJJMMNP_01065 | CDS | 1845 | *dnaJ_2* |  |  | Chaperone protein DnaJ |
| SB021 contig 1 | LEJJMMNP_01066 | CDS | 1371 | *dnaJ_3* |  |  | Chaperone protein DnaJ |
| SB021 contig 1 | LEJJMMNP_01067 | CDS | 672 |  |  |  | Hypothetical protein |
| SB021 contig 1 | LEJJMMNP_01068 | CDS | 168 |  |  |  | Hypothetical protein |
| SB021 contig 1 | LEJJMMNP_01069 | CDS | 1098 |  |  |  | Hypothetical protein |
| SB021 contig 1 | LEJJMMNP_01070 | CDS | 735 |  |  |  | Hypothetical protein |
| SB021 contig 1 | LEJJMMNP_01071 | CDS | 1299 |  |  |  | Hypothetical protein |
| SB021 contig 1 | LEJJMMNP_01072 | CDS | 1311 |  |  |  | Hypothetical protein |
| SB021 contig 1 | LEJJMMNP_01073 | CDS | 1641 |  |  |  | Hypothetical protein |
| SB021 contig 1 | LEJJMMNP_01074 | CDS | 1035 |  | 3.6.1.- | COG1106 | ATP/GTP phosphatase |
| SB021 contig 1 | LEJJMMNP_01075 | CDS | 774 |  |  |  | Hypothetical protein |
| SB021 contig 1 | LEJJMMNP_01076 | CDS | 1626 |  |  |  | Hypothetical protein |
| SB021 contig 1 | LEJJMMNP_01077 | CDS | 561 |  |  |  | Hypothetical protein |
| SB021 contig 1 | LEJJMMNP_01078 | CDS | 597 |  |  |  | Hypothetical protein |
| SB021 contig 1 | LEJJMMNP_01079 | CDS | 2019 |  |  |  | Hypothetical protein |
| SB021 contig 1 | LEJJMMNP_01080 | CDS | 2514 |  |  |  | Hypothetical protein |
| SB021 contig 1 | LEJJMMNP_01081 | CDS | 3618 |  |  |  | Hypothetical protein |
| SB021 contig 1 | LEJJMMNP_01082 | CDS | 3735 |  |  |  | Hypothetical protein |
| SB021 contig 1 | LEJJMMNP_01083 | CDS | 204 |  |  |  | Hypothetical protein |
| SB021 contig 1 | LEJJMMNP_01084 | tRNA | 76 |  |  |  | tRNA-Asn(gtt) |
| SB021 contig 1 | LEJJMMNP_01085 | rRNA | 95 |  |  |  | 5S ribosomal RNA |
| SB021 contig 1 | LEJJMMNP_01086 | rRNA | 2903 |  |  |  | 23S ribosomal RNA |
| SB021 contig 1 | LEJJMMNP_01087 | rRNA | 1511 |  |  |  | 16A ribosomal RNA |
| SB021 contig 1 | LEJJMMNP_01088 | CDS | 648 |  |  |  | Hypothetical protein |
| SB021 contig 1 | LEJJMMNP_01089 | tRNA | 93 |  |  |  | tRNA-SeC(tca) |
| SB021 contig 1 | LEJJMMNP_01090 | CDS | 426 | *pepV* | 3.4.13.- |  | Beta-Ala-Xaa dipeptidase |
| SB021 contig 1 | LEJJMMNP_01091 | CDS | 1017 |  | 3.4.13.- |  | Putative dipeptidase |
| SB021 contig 1 | LEJJMMNP_01092 | CDS | 726 | *fabG* | 1.1.1.100 |  | 3-oxoacyl-[acyl-carrier-protein] reductase FabG |
| SB021 contig 1 | LEJJMMNP_01093 | CDS | 633 | *pyre* | 2.4.2.10 | COG0461 | Orotate phosphoribosyltransferase |
| SB021 contig 1 | LEJJMMNP_01094 | CDS | 1197 | *pyrC* | 3.5.2.3 | COG0044 | Dihydroorotase |
| SB021 contig 1 | LEJJMMNP_01095 | CDS | 729 | *pyrF* | 4.1.1.23 | COG0284 | Orotidine 5'-phosphate decarboxylase |
| SB021 contig 1 | LEJJMMNP_01096 | CDS | 936 | *pyrD* | 1.3.1.14 | COG0167 | Dihydroorotate dehydrogenase B (NAD(+)), catalytic subunit |
| SB021 contig 1 | LEJJMMNP_01097 | CDS | 804 | *pyrK* |  | COG0543 | Dihydroorotate dehydrogenase B (NAD(+)), electron transfer subunit |
| SB021 contig 1 | LEJJMMNP_01098 | CDS | 1065 | *pyrB* | 2.1.3.2 | COG0540 | Aspartate carbamoyltransferase catalytic subunit |
| SB021 contig 1 | LEJJMMNP_01099 | CDS | 1791 | *hflX* |  |  | GTPase HflX |
| SB021 contig 1 | LEJJMMNP_01100 | CDS | 1173 | *sstT_1* |  |  | Serine/threonine transporter SstT |
| SB021 contig 1 | LEJJMMNP_01101 | CDS | 1485 |  |  |  | Hypothetical protein |
| SB021 contig 1 | LEJJMMNP_01102 | CDS | 141 |  |  |  | Hypothetical protein |
| SB021 contig 1 | LEJJMMNP_01103 | CDS | 699 | *ybhL* |  | COG0670 | Inner membrane protein YbhL |
| SB021 contig 1 | LEJJMMNP_01104 | CDS | 582 | *chrA* |  |  | Chromate transport protein |
| SB021 contig 1 | LEJJMMNP_01105 | CDS | 558 | *srpC* |  | COG2059 | Putative chromate transport protein |
| SB021 contig 1 | LEJJMMNP_01106 | CDS | 387 |  |  |  | Hypothetical protein |
| SB021 contig 1 | LEJJMMNP_01107 | CDS | 3207 | *carB* | 6.3.5.5 |  | Carbamoyl-phosphate synthase large chain |
| SB021 contig 1 | LEJJMMNP_01108 | CDS | 1035 | *carA_2* | 6.3.5.5 | COG0505 | Carbamoyl-phosphate synthase small chain |
| SB021 contig 1 | LEJJMMNP_01109 | CDS | 378 |  |  |  | Hypothetical protein |
| SB021 contig 1 | LEJJMMNP_01110 | CDS | 657 |  |  |  | Hypothetical protein |
| SB021 contig 1 | LEJJMMNP_01111 | CDS | 2466 | *menE* | 6.2.1.26 |  | 2-succinylbenzoate--CoA ligase |
| SB021 contig 1 | LEJJMMNP_01112 | CDS | 600 | *lysO* |  | COG2431 | Lysine exporter LysO |
| SB021 contig 1 | LEJJMMNP_01113 | CDS | 285 |  |  |  | Hypothetical protein |
| SB021 contig 1 | LEJJMMNP_01114 | CDS | 924 | *trxB* | 1.8.1.9 | COG0492 | Thioredoxin reductase |
| SB021 contig 1 | LEJJMMNP_01115 | CDS | 627 | *gloC* | 3.1.2.6 | COG0491 | Hydroxyacylglutathione hydrolase GloC |
| SB021 contig 1 | LEJJMMNP_01116 | CDS | 465 | *trmL* | 2.1.1.207 | COG0219 | tRNA (cytidine(34)-2'-O)-methyltransferase |
| SB021 contig 1 | LEJJMMNP_01117 | CDS | 1398 |  |  |  | Hypothetical protein |
| SB021 contig 1 | LEJJMMNP_01118 | CDS | 585 |  |  |  | Hypothetical protein |
| SB021 contig 1 | LEJJMMNP_01119 | CDS | 966 |  |  |  | Hypothetical protein |
| SB021 contig 1 | LEJJMMNP_01120 | CDS | 636 | *artM* |  | COG1126 | Arginine transport ATP-binding protein ArtM |
| SB021 contig 1 | LEJJMMNP_01121 | CDS | 630 | *yecS* |  | COG0765 | L-cystine transport system permease protein YecS |
| SB021 contig 1 | LEJJMMNP_01122 | CDS | 771 | *fliY* |  | COG0834 | L-cystine-binding protein FliY |
| SB021 contig 1 | LEJJMMNP_01123 | CDS | 615 | *cutC* |  | COG3142 | Copper homeostasis protein CutC |
| SB021 contig 1 | LEJJMMNP_01124 | CDS | 1566 | *clcA* |  | COG0038 | H(+)/Cl(-) exchange transporter ClcA |
| SB021 contig 1 | LEJJMMNP_01125 | CDS | 2559 |  |  |  | Hypothetical protein |
| SB021 contig 1 | LEJJMMNP_01126 | CDS | 1968 | *mutL* |  |  | DNA mismatch repair protein MutL |
| SB021 contig 1 | LEJJMMNP_01127 | CDS | 468 | *rlmH* | 2.1.1.177 | COG1576 | Ribosomal RNA large subunit methyltransferase H |
| SB021 contig 1 | LEJJMMNP_01128 | CDS | 321 |  |  |  | Hypothetical protein |
| SB021 contig 1 | LEJJMMNP_01129 | CDS | 369 |  |  |  | Hypothetical protein |
| SB021 contig 1 | LEJJMMNP_01130 | CDS | 1401 | *cpoB_2* |  |  | Cell division coordinator CpoB |
| SB021 contig 1 | LEJJMMNP_01131 | CDS | 1488 | *lysS* | 6.1.1.6 |  | Lysine--tRNA ligase |
| SB021 contig 1 | LEJJMMNP_01132 | CDS | 711 |  |  |  | Hypothetical protein |
| SB021 contig 1 | LEJJMMNP_01133 | CDS | 840 |  |  |  | Hypothetical protein |
| SB021 contig 1 | LEJJMMNP_01134 | CDS | 450 |  |  |  | Hypothetical protein |
| SB021 contig 1 | LEJJMMNP_01135 | CDS | 1281 | *mtaD* | 3.5.4.28 | COG0402 | 5-methylthioadenosine/S-adenosylhomocysteine deaminase |
| SB021 contig 1 | LEJJMMNP_01136 | CDS | 726 |  |  |  | Hypothetical protein |
| SB021 contig 1 | LEJJMMNP_01137 | CDS | 1959 | *mdoB* | 2.7.8.20 |  | Phosphoglycerol transferase I |
| SB021 contig 1 | LEJJMMNP_01138 | CDS | 348 | *lrgA* |  | COG1380 | Antiholin-like protein LrgA |
| SB021 contig 1 | LEJJMMNP_01139 | CDS | 696 | *yohK* |  | COG1346 | Inner membrane protein YohK |
| SB021 contig 1 | LEJJMMNP_01140 | CDS | 258 |  |  |  | Hypothetical protein |
| SB021 contig 1 | LEJJMMNP_01141 | CDS | 549 | *rbr3A* | 1.11.1.1 | COG1592 | Reverse rubrerythrin-1 |
| SB021 contig 1 | LEJJMMNP_01142 | CDS | 438 | *nsrR* |  |  | HTH-type transcriptional repressor NsrR |
| SB021 contig 1 | LEJJMMNP_01143 | CDS | 438 |  |  |  | Hypothetical protein |
| SB021 contig 1 | LEJJMMNP_01144 | CDS | 633 | *ywlC* | 2.7.7.87 | COG0009 | Threonylcarbamoyl-AMP synthase |
| SB021 contig 1 | LEJJMMNP_01145 | CDS | 972 | *prs* | 2.7.6.1 | COG0462 | Ribose-phosphate pyrophosphokinase |
| SB021 contig 1 | LEJJMMNP_01146 | CDS | 1347 | *glmU* |  | COG1207 | Bifunctional protein GlmU |
| SB021 contig 1 | LEJJMMNP_01147 | CDS | 549 | *dnaJ_4* |  |  | Chaperone protein DnaJ |
| SB021 contig 1 | LEJJMMNP_01148 | CDS | 570 |  |  |  | Hypothetical protein |
| SB021 contig 1 | LEJJMMNP_01149 | CDS | 693 |  |  |  | Hypothetical protein |
| SB021 contig 1 | LEJJMMNP_01150 | CDS | 888 |  | 2.5.1.10 |  | Farnesyl diphosphate synthase |
| SB021 contig 1 | LEJJMMNP_01151 | CDS | 222 | *xseB* | 3.1.11.6 |  | Exodeoxyribonuclease 7 small subunit |
| SB021 contig 1 | LEJJMMNP_01152 | CDS | 552 | *rsmD* | 2.1.1.171 | COG0742 | Ribosomal RNA small subunit methyltransferase D |
| SB021 contig 1 | LEJJMMNP_01153 | CDS | 1032 | *queA* | 2.4.99.17 |  | S-adenosylmethionine:tRNA ribosyltransferase-isomerase |
| SB021 contig 1 | LEJJMMNP_01154 | CDS | 1143 | *prmC_2* | 2.1.1.297 |  | Release factor glutamine methyltransferase |
| SB021 contig 1 | LEJJMMNP_01155 | CDS | 1077 | *prfA* |  | COG0216 | Peptide chain release factor 1 |
| SB021 contig 1 | LEJJMMNP_01156 | CDS | 1299 |  |  |  | Hypothetical protein |
| SB021 contig 1 | LEJJMMNP_01157 | CDS | 387 |  |  |  | Hypothetical protein |
| SB021 contig 1 | LEJJMMNP_01158 | CDS | 183 |  |  |  | Hypothetical protein |
| SB021 contig 1 | LEJJMMNP_01159 | CDS | 282 | *ihfA_2* |  |  | Integration host factor subunit alpha |
| SB021 contig 1 | LEJJMMNP_01160 | CDS | 276 | *ihfA_3* |  |  | Integration host factor subunit alpha |
| SB021 contig 1 | LEJJMMNP_01161 | CDS | 1506 | *murJ_3* |  |  | Lipid II flippase MurJ |
| SB021 contig 1 | LEJJMMNP_01162 | CDS | 1170 |  |  |  | Hypothetical protein |
| SB021 contig 1 | LEJJMMNP_01163 | CDS | 1071 |  |  |  | Hypothetical protein |
| SB021 contig 1 | LEJJMMNP_01164 | CDS | 954 | *arnC* | 2.4.2.53 |  | Undecaprenyl-phosphate 4-deoxy-4-formamido-L-arabinose transferase |
| SB021 contig 1 | LEJJMMNP_01165 | CDS | 600 |  |  |  | Hypothetical protein |
| SB021 contig 1 | LEJJMMNP_01166 | CDS | 405 | *glyG* |  |  | Glycosyltransferase GlyG |
| SB021 contig 1 | LEJJMMNP_01167 | CDS | 1098 | *glf* | 5.4.99.9 | COG0562 | UDP-galactopyranose mutase |
| SB021 contig 1 | LEJJMMNP_01168 | CDS | 780 |  |  |  | Hypothetical protein |
| SB021 contig 1 | LEJJMMNP_01169 | CDS | 702 |  |  |  | Hypothetical protein |
| SB021 contig 1 | LEJJMMNP_01170 | CDS | 1335 |  |  |  | Hypothetical protein |
| SB021 contig 1 | LEJJMMNP_01171 | CDS | 1716 | *proS* | 6.1.1.15 | COG0442 | Proline—tRNA ligase |
| SB021 contig 1 | LEJJMMNP_01172 | CDS | 2061 | *recG* | 3.6.4.12 |  | ATP-dependent DNA helicase RecG |
| SB021 contig 1 | LEJJMMNP_01173 | CDS | 753 | *pmpR* |  | COG0217 | Transcriptional regulatory protein PmpR |
| SB021 contig 1 | LEJJMMNP_01174 | CDS | 579 | *yqgN* |  | COG0212 | Putative protein YqgN |
| SB021 contig 1 | LEJJMMNP_01175 | CDS | 597 | *udg* | 3.2.2.27 | COG1573 | Type-4 uracil-DNA glycosylase |
| SB021 contig 1 | LEJJMMNP_01176 | CDS | 783 | *yycJ* | 3.-.-.- | COG1235 | Putative metallo-hydrolase YycJ |
| SB021 contig 1 | LEJJMMNP_01177 | CDS | 798 |  |  |  | Hypothetical protein |
| SB021 contig 1 | LEJJMMNP_01178 | CDS | 1272 |  |  |  | Hypothetical protein |
| SB021 contig 1 | LEJJMMNP_01179 | CDS | 1209 | *tdcB* | 4.3.1.19 | COG1171 | L-threonine ammonia-lyase |
| SB021 contig 1 | LEJJMMNP_01180 | CDS | 1473 | *cysG* | 4.99.1.4 |  | Siroheme synthase |
| SB021 contig 1 | LEJJMMNP_01181 | CDS | 903 | *hemC* | 2.5.1.61 |  | Porphobilinogen deaminase |
| SB021 contig 1 | LEJJMMNP_01182 | CDS | 768 | *cbiJ* | 1.3.1.106 | COG2099 | Cobalt-precorrin-6A reductase |
| SB021 contig 1 | LEJJMMNP_01183 | CDS | 738 |  | 2.1.1.272 | COG1010 | Cobalt-factor III methyltransferase |
| SB021 contig 1 | LEJJMMNP_01184 | CDS | 873 | *cbiG* | 3.7.1.12 | COG2073 | Cobalt-precorrin-5A hydrolase |
| SB021 contig 1 | LEJJMMNP_01185 | CDS | 150 |  |  |  | Hypothetical protein |
| SB021 contig 1 | LEJJMMNP_01186 | CDS | 501 | *cbiF_1* | 2.1.1.271 | COG2875 | Cobalt-precorrin-4 C(11)-methyltransferase |
| SB021 contig 1 | LEJJMMNP_01187 | CDS | 222 | *cbiF_2* | 2.1.1.271 | COG2875 | Cobalt-precorrin-4 C(11)-methyltransferase |
| SB021 contig 1 | LEJJMMNP_01188 | CDS | 720 | *cbiL* | 2.1.1.151 | COG2243 | Cobalt-precorrin-2 C(20)-methyltransferase |
| SB021 contig 1 | LEJJMMNP_01189 | CDS | 579 | *cbiT* | 2.1.1.196 | COG2242 | Cobalt-precorrin-6B C(15)-methyltransferase (decarboxylating) |
| SB021 contig 1 | LEJJMMNP_01190 | CDS | 636 | *cbiE* | 2.1.1.289 | COG2241 | Cobalt-precorrin-7 C(5)-methyltransferase |
| SB021 contig 1 | LEJJMMNP_01191 | CDS | 1140 | *cbiD* | 2.1.1.195 | COG1903 | Cobalt-precorrin-5B C(1)-methyltransferase |
| SB021 contig 1 | LEJJMMNP_01192 | CDS | 648 | *cbiC* | 5.4.99.60 | COG2082 | Cobalt-precorrin-8 methylmutase |
| SB021 contig 1 | LEJJMMNP_01193 | CDS | 1341 | *cbiA* | 6.3.5.11 | COG1797 | Cobyrinate a,c-diamide synthase |
| SB021 contig 1 | LEJJMMNP_01194 | CDS | 1068 | *cobD_1* | 4.1.1.81 | COG0079 | Threonine-phosphate decarboxylase |
| SB021 contig 1 | LEJJMMNP_01195 | CDS | 948 | *cobD_2* |  | COG1270 | Cobalamin biosynthesis protein CobD |
| SB021 contig 1 | LEJJMMNP_01196 | CDS | 927 | *cobQ_2* |  | COG1492 | Cobyric acid synthase |
| SB021 contig 1 | LEJJMMNP_01197 | CDS | 963 | *iolU* | 1.1.1.371 | COG0673 | scyllo-inositol 2-dehydrogenase (NADP(+)) IolU |
| SB021 contig 1 | LEJJMMNP_01198 | CDS | 807 | *thiD* | 2.7.1.49 | COG0351 | Hydroxymethylpyrimidine/phosphomethylpyrimidine kinase |
| SB021 contig 1 | LEJJMMNP_01199 | CDS | 624 | *thiE_2* | 2.5.1.3 |  | Thiamine-phosphate synthase |
| SB021 contig 1 | LEJJMMNP_01200 | CDS | 819 | *thiM* | 2.7.1.50 | COG2145 | Hydroxyethylthiazole kinase |
| SB021 contig 1 | LEJJMMNP_01201 | CDS | 1488 |  |  |  | Hypothetical protein |
| SB021 contig 1 | LEJJMMNP_01202 | CDS | 1140 |  |  |  | Hypothetical protein |
| SB021 contig 1 | LEJJMMNP_01203 | CDS | 1476 |  |  |  | Hypothetical protein |
| SB021 contig 1 | LEJJMMNP_01204 | CDS | 300 |  |  |  | Hypothetical protein |
| SB021 contig 1 | LEJJMMNP_01205 | CDS | 297 |  |  |  | Hypothetical protein |
| SB021 contig 1 | LEJJMMNP_01206 | CDS | 1635 |  | 1.18.1.2 |  | Ferredoxin--NADP reductase |
| SB021 contig 1 | LEJJMMNP_01207 | CDS | 561 | *ahpC* | 1.11.1.15 | COG0450 | Alkyl hydroperoxide reductase C |
| SB021 contig 1 | LEJJMMNP_01208 | tRNA | 86 |  |  |  | tRNA-Leu(cag) |
| SB021 contig 1 | LEJJMMNP_01209 | CDS | 1395 |  |  |  | Hypothetical protein |
| SB021 contig 1 | LEJJMMNP_01210 | CDS | 486 |  |  |  | Hypothetical protein |
| SB021 contig 1 | LEJJMMNP_01211 | CDS | 1116 | *yqeH* |  | COG1161 | Putative protein YqeH |
| SB021 contig 1 | LEJJMMNP_01212 | CDS | 534 | *hslV* | 3.4.25.2 | COG5405 | ATP-dependent protease subunit HslV |
| SB021 contig 1 | LEJJMMNP_01213 | CDS | 900 | *xerD* |  |  | Tyrosine recombinase XerD |
| SB021 contig 1 | LEJJMMNP_01214 | CDS | 1302 | *trmFO* | 2.1.1.74 | COG1206 | Methylenetetrahydrofolate--tRNA-(uracil-5-)-methyltransferase TrmFO |
| SB021 contig 1 | LEJJMMNP_01215 | CDS | 414 |  |  |  | Hypothetical protein |
| SB021 contig 1 | LEJJMMNP_01216 | CDS | 351 |  |  |  | Hypothetical protein |
| SB021 contig 1 | LEJJMMNP_01217 | CDS | 858 |  |  |  | Hypothetical protein |
| SB021 contig 1 | LEJJMMNP_01218 | CDS | 1611 | *rpsA* |  | COG0539 | 30S ribosomal protein S1 |
| SB021 contig 1 | LEJJMMNP_01219 | CDS | 543 | *ispH_1* | 1.17.7.4 | COG0761 | 4-hydroxy-3-methylbut-2-enyl diphosphate reductase |
| SB021 contig 1 | LEJJMMNP_01220 | CDS | 231 | *ispH_2* | 1.17.7.4 |  | 4-hydroxy-3-methylbut-2-enyl diphosphate reductase |
| SB021 contig 1 | LEJJMMNP_01221 | CDS | 270 | *ptsH_2* |  | COG1925 | Phosphocarrier protein HPr |
| SB021 contig 1 | LEJJMMNP_01222 | CDS | 1731 | *ptsI_2* | 2.7.3.9 | COG1080 | Phosphoenolpyruvate-protein phosphotransferase |
| SB021 contig 1 | LEJJMMNP_01223 | CDS | 2118 | *glnA* | 6.3.1.2 |  | Glutamine synthetase |
| SB021 contig 1 | LEJJMMNP_01224 | CDS | 546 | *yodC* | 1.-.-.- | COG0778 | Putative NAD(P)H nitroreductase YodC |
| SB021 contig 1 | LEJJMMNP_01225 | CDS | 1305 |  |  |  | Hypothetical protein |
| SB021 contig 1 | LEJJMMNP_01226 | CDS | 600 | *ruvA* | 3.6.4.12 | COG0632 | Holliday junction ATP-dependent DNA helicase RuvA |
| SB021 contig 1 | LEJJMMNP_01227 | CDS | 2826 | *uvrA* |  |  | UvrABC system protein A |
| SB021 contig 1 | LEJJMMNP_01228 | CDS | 573 | *ruvC* | 3.1.22.4 |  | Crossover junction endodeoxyribonuclease RuvC |
| SB021 contig 1 | LEJJMMNP_01229 | CDS | 300 | *hupB* |  | COG0776 | DNA-binding protein HU-beta |
| SB021 contig 1 | LEJJMMNP_01230 | CDS | 1410 | *tpl* | 4.1.99.2 |  | Tyrosine phenol-lyase |
| SB021 contig 1 | LEJJMMNP_01231 | CDS | 1497 | *alsT_2* |  | COG1115 | Amino-acid carrier protein AlsT |
| SB021 contig 1 | LEJJMMNP_01232 | CDS | 1086 |  | 1.8.-.- | COG0502 | [FeFe] hydrogenase maturase subunit HydE |
| SB021 contig 1 | LEJJMMNP_01233 | CDS | 2388 | *kdpB* | 7.2.2.6 |  | Potassium-transporting ATPase ATP-binding subunit |
| SB021 contig 1 | LEJJMMNP_01234 | CDS | 1506 |  |  |  | Hypothetical protein |
| SB021 contig 1 | LEJJMMNP_01235 | CDS | 879 |  |  |  | Hypothetical protein |
| SB021 contig 1 | LEJJMMNP_01236 | CDS | 1014 | *gale* | 5.1.3.2 | COG1087 | UDP-glucose 4-epimerase |
| SB021 contig 1 | LEJJMMNP_01237 | CDS | 1386 | *phoB* | 3.1.3.1 | COG1785 | Alkaline phosphatase 3 |
| SB021 contig 1 | LEJJMMNP_01238 | CDS | 1122 |  |  |  | Hypothetical protein |
| SB021 contig 1 | LEJJMMNP_01239 | CDS | 1110 | *dthadh* | 4.3.1.27 |  | D-threo-3-hydroxyaspartate dehydratase |
| SB021 contig 1 | LEJJMMNP_01240 | CDS | 1329 | *dsdA* | 4.3.1.18 | COG3048 | D-serine dehydratase |
| SB021 contig 1 | LEJJMMNP_01241 | CDS | 1359 | *dsdX* |  |  | D-serine transporter DsdX |
| SB021 contig 1 | LEJJMMNP_01242 | CDS | 303 |  |  |  | Hypothetical protein |
| SB021 contig 1 | LEJJMMNP_01243 | CDS | 300 |  |  |  | Hypothetical protein |
| SB021 contig 1 | LEJJMMNP_01244 | CDS | 675 | *ung* | 3.2.2.27 | COG0692 | Uracil-DNA glycosylase |
| SB021 contig 1 | LEJJMMNP_01245 | CDS | 1767 |  |  |  | Hypothetical protein |
| SB021 contig 1 | LEJJMMNP_01246 | CDS | 423 |  |  |  | Hypothetical protein |
| SB021 contig 1 | LEJJMMNP_01247 | CDS | 849 | *truA_3* | 5.4.99.12 |  | tRNA pseudouridine synthase A |
| SB021 contig 1 | LEJJMMNP_01248 | CDS | 1239 | *dctA_2* |  | COG1301 | C4-dicarboxylate transport protein |
| SB021 contig 1 | LEJJMMNP_01249 | CDS | 261 |  |  |  | Hypothetical protein |
| SB021 contig 1 | LEJJMMNP_01250 | CDS | 2061 | *norR_3* |  |  | Anaerobic nitric oxide reductase transcription regulator NorR |
| SB021 contig 1 | LEJJMMNP_01251 | CDS | 1278 |  |  |  | Hypothetical protein |
| SB021 contig 1 | LEJJMMNP_01252 | CDS | 1185 | *patB_1* | 4.4.1.13 | COG1168 | Cystathionine beta-lyase PatB |
| SB021 contig 1 | LEJJMMNP_01253 | CDS | 1518 |  |  |  | Hypothetical protein |
| SB021 contig 1 | LEJJMMNP_01254 | CDS | 468 | *ybeY* | 3.1.-.- |  | Endoribonuclease YbeY |
| SB021 contig 1 | LEJJMMNP_01255 | CDS | 2073 |  |  |  | Hypothetical protein |
| SB021 contig 1 | LEJJMMNP_01256 | CDS | 2445 | *yoaA* | 3.6.4.12 | COG1199 | Putative ATP-dependent DNA helicase YoaA |
| SB021 contig 1 | LEJJMMNP_01257 | CDS | 261 | *rpsT* |  | COG0268 | 30S ribosomal protein S20 |
| SB021 contig 1 | LEJJMMNP_01258 | CDS | 741 |  |  |  | Hypothetical protein |
| SB021 contig 1 | LEJJMMNP_01259 | CDS | 1041 | *ldhA* | 1.1.1.28 | COG1052 | D-lactate dehydrogenase |
| SB021 contig 1 | LEJJMMNP_01260 | CDS | 1464 | *nhaC* |  | COG1757 | Na(+)/H(+) antiporter NhaC |
| SB021 contig 1 | LEJJMMNP_01261 | CDS | 339 |  | 3.9.1.- | COG0537 | Purine nucleoside phosphoramidase |
| SB021 contig 1 | LEJJMMNP_01262 | CDS | 429 | *rpiB* | 5.3.1.6 | COG0698 | Ribose-5-phosphate isomerase B |
| SB021 contig 1 | LEJJMMNP_01263 | CDS | 480 | *slyD* | 5.2.1.8 | COG1047 | FKBP-type peptidyl-prolyl cis-trans isomerase SlyD |
| SB021 contig 1 | LEJJMMNP_01264 | CDS | 648 |  |  |  | Hypothetical protein |
| SB021 contig 1 | LEJJMMNP_01265 | CDS | 2352 | *hutG* | 3.5.3.8 |  | Formimidoylglutamase |
| SB021 contig 1 | LEJJMMNP_01266 | CDS | 852 |  |  |  | Hypothetical protein |
| SB021 contig 1 | LEJJMMNP_01267 | CDS | 831 |  |  |  | Hypothetical protein |
| SB021 contig 1 | LEJJMMNP_01268 | CDS | 261 |  |  |  | Hypothetical protein |
| SB021 contig 1 | LEJJMMNP_01269 | CDS | 444 |  |  |  | Hypothetical protein |
| SB021 contig 1 | LEJJMMNP_01270 | CDS | 3069 |  |  |  | Hypothetical protein |
| SB021 contig 1 | LEJJMMNP_01271 | CDS | 591 |  |  |  | Hypothetical protein |
| SB021 contig 1 | LEJJMMNP_01272 | CDS | 228 |  |  |  | Hypothetical protein |
| SB021 contig 1 | LEJJMMNP_01273 | CDS | 1239 | *rarA* |  | COG2256 | Replication-associated recombination protein A |
| SB021 contig 1 | LEJJMMNP_01274 | CDS | 1242 | *hisS* | 6.1.1.21 | COG0124 | Histidine—tRNA ligase |
| SB021 contig 1 | LEJJMMNP_01275 | CDS | 1779 | *aspS* | 6.1.1.12 |  | Aspartate—tRNA ligase |
| SB021 contig 1 | LEJJMMNP_01276 | CDS | 1716 | *argS* | 6.1.1.19 |  | Arginine—tRNA ligase |
| SB021 contig 1 | LEJJMMNP_01277 | CDS | 1005 | *btuD* | 7.6.2.8 |  | Vitamin B12 import ATP-binding protein BtuD |
| SB021 contig 1 | LEJJMMNP_01278 | CDS | 801 |  |  |  | Hypothetical protein |
| SB021 contig 1 | LEJJMMNP_01279 | CDS | 852 |  |  |  | Hypothetical protein |
| SB021 contig 1 | LEJJMMNP_01280 | CDS | 822 |  |  |  | Hypothetical protein |
| SB021 contig 1 | LEJJMMNP_01281 | CDS | 1083 |  |  |  | Hypothetical protein |
| SB021 contig 1 | LEJJMMNP_01282 | CDS | 813 | *hisK_2* | 3.1.3.15 | COG1387 | Histidinol-phosphatase |
| SB021 contig 1 | LEJJMMNP_01283 | CDS | 408 |  |  |  | Hypothetical protein |
| SB021 contig 1 | LEJJMMNP_01284 | CDS | 405 |  |  |  | Hypothetical protein |
| SB021 contig 1 | LEJJMMNP_01285 | CDS | 837 | *nadE* | 6.3.1.5 | COG0171 | NH(3)-dependent NAD(+) synthetase |
| SB021 contig 1 | LEJJMMNP_01286 | CDS | 552 | *rbgA_1* |  | COG1161 | Ribosome biogenesis GTPase A |
| SB021 contig 1 | LEJJMMNP_01287 | CDS | 348 | *rbgA_2* |  | COG1161 | Ribosome biogenesis GTPase A |
| SB021 contig 1 | LEJJMMNP_01288 | CDS | 741 | *rsmI* | 2.1.1.198 | COG0313 | Ribosomal RNA small subunit methyltransferase I |
| SB021 contig 1 | LEJJMMNP_01289 | CDS | 1284 |  |  |  | Hypothetical protein |
| SB021 contig 1 | LEJJMMNP_01290 | CDS | 816 | *tlyA* |  | COG1189 | Hemolysin A |
| SB021 contig 1 | LEJJMMNP_01291 | CDS | 852 |  |  |  | Hypothetical protein |
| SB021 contig 1 | LEJJMMNP_01292 | CDS | 1806 | *dxs* | 2.2.1.7 | COG1154 | 1-deoxy-D-xylulose-5-phosphate synthase |
| SB021 contig 1 | LEJJMMNP_01293 | CDS | 198 |  |  |  | Hypothetical protein |
| SB021 contig 1 | LEJJMMNP_01294 | CDS | 267 |  |  |  | Hypothetical protein |
| SB021 contig 1 | LEJJMMNP_01295 | CDS | 303 | *yhbY* |  | COG1534 | RNA-binding protein YhbY |
| SB021 contig 1 | LEJJMMNP_01296 | CDS | 1656 | *rnjA* | 3.1.-.- | COG0595 | Ribonuclease J1 |
| SB021 contig 1 | LEJJMMNP_01297 | CDS | 1803 | *mrdA* | 3.4.16.4 | COG0768 | Peptidoglycan D,D-transpeptidase MrdA |
| SB021 contig 1 | LEJJMMNP_01298 | CDS | 420 |  |  |  | Hypothetical protein |
| SB021 contig 1 | LEJJMMNP_01299 | CDS | 990 |  |  |  | Hypothetical protein |
| SB021 contig 1 | LEJJMMNP_01300 | CDS | 1323 | *mtaB* | 2.8.4.5 | COG0621 | Threonylcarbamoyladenosine tRNA methylthiotransferase MtaB |
| SB021 contig 1 | LEJJMMNP_01301 | CDS | 717 | *rsmE* | 2.1.1.193 | COG1385 | Ribosomal RNA small subunit methyltransferase E |
| SB021 contig 1 | LEJJMMNP_01302 | CDS | 423 | *cymR* |  |  | HTH-type transcriptional regulator CymR |
| SB021 contig 1 | LEJJMMNP_01303 | CDS | 990 | *ruvB* | 3.6.4.12 | COG2255 | Holliday junction ATP-dependent DNA helicase RuvB |
| SB021 contig 1 | LEJJMMNP_01304 | CDS | 603 |  |  |  | Hypothetical protein |
| SB021 contig 1 | LEJJMMNP_01305 | CDS | 954 |  |  |  | Hypothetical protein |
| SB021 contig 1 | LEJJMMNP_01306 | CDS | 858 |  |  |  | Hypothetical protein |
| SB021 contig 1 | LEJJMMNP_01307 | CDS | 231 |  |  |  | Hypothetical protein |
| SB021 contig 1 | LEJJMMNP_01308 | CDS | 324 |  |  |  | Hypothetical protein |
| SB021 contig 1 | LEJJMMNP_01309 | CDS | 717 | *ygaZ* |  | COG1296 | Inner membrane protein YgaZ |
| SB021 contig 1 | LEJJMMNP_01310 | CDS | 1344 | *gdh* | 1.4.1.2 | COG0334 | NAD-specific glutamate dehydrogenase |
| SB021 contig 1 | LEJJMMNP_01311 | CDS | 1017 | *birA_1* | 6.3.4.15 | COG0340 | Bifunctional ligase/repressor BirA |
| SB021 contig 1 | LEJJMMNP_01312 | CDS | 1317 | *opuE_2* |  | COG0591 | Osmoregulated proline transporter OpuE |
| SB021 contig 1 | LEJJMMNP_01313 | CDS | 240 |  |  |  | Hypothetical protein |
| SB021 contig 1 | LEJJMMNP_01314 | CDS | 663 |  |  |  | Hypothetical protein |
| SB021 contig 1 | LEJJMMNP_01315 | CDS | 303 |  |  |  | Hypothetical protein |
| SB021 contig 1 | LEJJMMNP_01316 | CDS | 510 |  |  |  | Hypothetical protein |
| SB021 contig 1 | LEJJMMNP_01317 | CDS | 978 |  | 3.4.11.- | COG3191 | Putative aminopeptidase |
| SB021 contig 1 | LEJJMMNP_01318 | CDS | 1215 |  | 3.-.-.- | COG0624 | Putative hydrolase |
| SB021 contig 1 | LEJJMMNP_01319 | CDS | 1236 | *sstT_2* |  | COG3633 | Serine/threonine transporter SstT |
| SB021 contig 1 | LEJJMMNP_01320 | CDS | 438 |  |  |  | Hypothetical protein |
| SB021 contig 1 | LEJJMMNP_01321 | CDS | 675 | *deoC* | 4.1.2.4 |  | Deoxyribose-phosphate aldolase |
| SB021 contig 1 | LEJJMMNP_01322 | CDS | 1191 | *deoB* | 5.4.2.7 | COG1015 | Phosphopentomutase |
| SB021 contig 1 | LEJJMMNP_01323 | CDS | 711 | *deoD* | 2.4.2.1 |  | Purine nucleoside phosphorylase DeoD-type |
| SB021 contig 1 | LEJJMMNP_01324 | CDS | 399 | *cdd* | 3.5.4.5 | COG0295 | Cytidine deaminase |
| SB021 contig 1 | LEJJMMNP_01325 | CDS | 1224 | *nupX* |  | COG1972 | Putative nucleoside permease NupX |
| SB021 contig 1 | LEJJMMNP_01326 | CDS | 780 | *udp* | 2.4.2.3 | COG2820 | Uridine phosphorylase |
| SB021 contig 1 | LEJJMMNP_01327 | CDS | 747 | *fadR* |  |  | Fatty acid metabolism regulator protein |
| SB021 contig 1 | LEJJMMNP_01328 | CDS | 1356 | *mepA_4* |  |  | Multidrug export protein MepA |
| SB021 contig 1 | LEJJMMNP_01329 | CDS | 789 | *coax* | 2.7.1.33 | COG1521 | Type III pantothenate kinase |
| SB021 contig 1 | LEJJMMNP_01330 | CDS | 1173 | *tmcAL* | 6.3.4.- | COG1323 | Type III pantothenate kinase |
| SB021 contig 1 | LEJJMMNP_01331 | CDS | 1293 |  |  |  | Hypothetical protein |
| SB021 contig 1 | LEJJMMNP_01332 | CDS | 1311 |  |  |  | Hypothetical protein |
| SB021 contig 1 | LEJJMMNP_01333 | CDS | 732 |  |  |  | Hypothetical protein |
| SB021 contig 1 | LEJJMMNP_01334 | CDS | 579 | *yigZ* |  | COG1739 | IMPACT family member YigZ |
| SB021 contig 1 | LEJJMMNP_01335 | CDS | 1416 | *ttgC* |  | COG1538 | Putative efflux pump outer membrane protein TtgC |
| SB021 contig 1 | LEJJMMNP_01336 | CDS | 1182 | *emrA* |  | COG1566 | Multidrug export protein EmrA |
| SB021 contig 1 | LEJJMMNP_01337 | CDS | 1485 | *emrB* |  |  | Multidrug export protein EmrB |
| SB021 contig 1 | LEJJMMNP_01338 | CDS | 318 |  |  |  | Hypothetical protein |
| SB021 contig 1 | LEJJMMNP_01339 | tRNA | 74 |  |  |  | tRNA-Cys(gca) |
| SB021 contig 1 | LEJJMMNP_01340 | tRNA | 76 |  |  |  | tRNA-Phe(gaa) |
| SB021 contig 1 | LEJJMMNP_01341 | tRNA | 77 |  |  |  | tRNA-Asp(gtc) |
| SB021 contig 1 | LEJJMMNP_01342 | tRNA | 76 |  |  |  | tRNA-Val(tac) |
| SB021 contig 1 | LEJJMMNP_01343 | CDS | 918 |  |  |  | Hypothetical protein |
| SB021 contig 1 | LEJJMMNP_01344 | CDS | 1032 | *mreB_2* |  | COG1077 | Cell shape-determining protein MreB |
| SB021 contig 1 | LEJJMMNP_01345 | CDS | 384 | *mrnCL* |  | COG1939 | Mini-ribonuclease 3-like protein |
| SB021 contig 1 | LEJJMMNP_01346 | CDS | 1425 | *cysS* | 6.1.1.16 | COG0215 | Cysteine--tRNA ligase |
| SB021 contig 1 | LEJJMMNP_01347 | CDS | 1182 |  |  |  | Hypothetical protein |
| SB021 contig 1 | LEJJMMNP_01348 | CDS | 804 | *ramA* | 3.5.1.100 |  | (R)-stereoselective amidase |
| SB021 contig 1 | LEJJMMNP_01349 | CDS | 705 | *ispD* | 2.7.7.60 | COG1211 | 2-C-methyl-D-erythritol 4-phosphate cytidylyltransferase |
| SB021 contig 1 | LEJJMMNP_01350 | CDS | 2340 | *mutS2* | 3.1.-.- |  | Endonuclease MutS2 |
| SB021 contig 1 | LEJJMMNP_01351 | CDS | 258 |  |  |  | Hypothetical protein |
| SB021 contig 1 | LEJJMMNP_01352 | CDS | 495 |  |  | COG0716 | Flavodoxin 1 |
| SB021 contig 1 | LEJJMMNP_01353 | CDS | 993 |  |  |  | Hypothetical protein |
| SB021 contig 1 | LEJJMMNP_01354 | CDS | 924 | *cbiK* | 4.99.1.3 | COG4822 | Sirohydrochlorin cobaltochelatase |
| SB021 contig 1 | LEJJMMNP_01355 | CDS | 621 |  |  |  | Hypothetical protein |
| SB021 contig 1 | LEJJMMNP_01356 | CDS | 141 |  |  |  | Hypothetical protein |
| SB021 contig 1 | LEJJMMNP_01357 | CDS | 1140 | *braC_3* |  | COG0683 | Leucine-, isoleucine-, valine-, threonine-, and alanine-binding protein |
| SB021 contig 1 | LEJJMMNP_01358 | CDS | 888 | *rluC* | 5.4.99.24 | COG0564 | Ribosomal large subunit pseudouridine synthase C |
| SB021 contig 1 | LEJJMMNP_01359 | CDS | 1113 | *mrdB* | 2.4.1.129 | COG0772 | Peptidoglycan glycosyltransferase MrdB |
| SB021 contig 1 | LEJJMMNP_01360 | CDS | 441 | *dut* | 3.6.1.23 |  | Deoxyuridine 5'-triphosphate nucleotidohydrolase |
| SB021 contig 1 | LEJJMMNP_01361 | CDS | 1224 |  | 3.4.24.- | COG0612 | Putative zinc protease |
| SB021 contig 1 | LEJJMMNP_01362 | CDS | 1095 |  |  |  | Hypothetical protein |
| SB021 contig 1 | LEJJMMNP_01363 | CDS | 1080 |  |  |  | Hypothetical protein |
| SB021 contig 1 | LEJJMMNP_01364 | CDS | 531 |  |  |  | Hypothetical protein |
| SB021 contig 1 | LEJJMMNP_01365 | CDS | 1176 | *rlmI* | 2.1.1.191 | COG1092 | Ribosomal RNA large subunit methyltransferase I |
| SB021 contig 1 | LEJJMMNP_01366 | CDS | 1083 | *alr2* | 5.1.1.1 | COG0787 | Alanine racemase 2 |
| SB021 contig 1 | LEJJMMNP_01367 | CDS | 942 | *secF* |  | COG0341 | Protein translocase subunit SecF |
| SB021 contig 1 | LEJJMMNP_01368 | CDS | 1224 | *decDF* |  | COG0341 | Protein translocase subunit SecDF |
| SB021 contig 1 | LEJJMMNP_01369 | CDS | 420 |  | 3.1.-.- | COG0816 | Putative pre-16S rRNA nuclease |
| SB021 contig 1 | LEJJMMNP_01370 | CDS | 2610 | *alaS_2* | 6.1.1.7 | COG0013 | Alanine—tRNA ligase |
| SB021 contig 1 | LEJJMMNP_01371 | CDS | 726 | *lptB_2* | 3.6.3.- | COG1137 | Lipopolysaccharide export system ATP-binding protein LptB |
| SB021 contig 1 | LEJJMMNP_01372 | CDS | 2715 | *lptD* |  |  | LPS-assembly protein LptD |
| SB021 contig 1 | LEJJMMNP_01373 | CDS | 2613 | *mutS* |  | COG0249 | DNA mismatch repair protein MutS |
| SB021 contig 1 | LEJJMMNP_01374 | CDS | 933 | *dusB* | 1.3.1.- | COG0042 | tRNA-dihydrouridine synthase B |
| SB021 contig 1 | LEJJMMNP_01375 | CDS | 951 | *aptB_2* | 2.2.1.- | COG3958 | Apulose-4-phosphate transketolase subunit B |
| SB021 contig 1 | LEJJMMNP_01376 | CDS | 822 | *aptA_2* | 2.2.1.- | COG3959 | Apulose-4-phosphate transketolase subunit A |
| SB021 contig 1 | LEJJMMNP_01377 | CDS | 1341 | *ulaA* |  | COG3037 | Ascorbate-specific PTS system EIIC component |
| SB021 contig 1 | LEJJMMNP_01378 | CDS | 276 |  |  |  | Hypothetical protein |
| SB021 contig 1 | LEJJMMNP_01379 | CDS | 426 | *ulaC* |  | COG1762 | Ascorbate-specific PTS system EIIA component |
| SB021 contig 1 | LEJJMMNP_01380 | CDS | 2061 | *manR* |  | COG1762 | Transcriptional regulator ManR |
| SB021 contig 1 | LEJJMMNP_01381 | CDS | 1464 |  |  |  | Hypothetical protein |
| SB021 contig 1 | LEJJMMNP_01382 | CDS | 1224 |  |  |  | Hypothetical protein |
| SB021 contig 1 | LEJJMMNP_01383 | CDS | 2379 |  |  |  | Hypothetical protein |
| SB021 contig 1 | LEJJMMNP_01384 | CDS | 345 |  |  |  | Hypothetical protein |
| SB021 contig 1 | LEJJMMNP_01385 | CDS | 459 |  |  |  | Hypothetical protein |
| SB021 contig 1 | LEJJMMNP_01386 | CDS | 831 |  |  |  | Hypothetical protein |
| SB021 contig 1 | LEJJMMNP_01387 | CDS | 489 |  |  |  | Hypothetical protein |
| SB021 contig 1 | LEJJMMNP_01388 | CDS | 387 |  |  |  | Hypothetical protein |
| SB021 contig 1 | LEJJMMNP_01389 | CDS | 1443 |  |  |  | Hypothetical protein |
| SB021 contig 1 | LEJJMMNP_01390 | CDS | 186 | *rpmF* |  | COG0333 | 50S ribosomal protein L32 |
| SB021 contig 1 | LEJJMMNP_01391 | CDS | 492 |  |  |  | Hypothetical protein |
| SB021 contig 1 | LEJJMMNP_01392 | CDS | 1095 | *ychF* |  | COG0012 | Ribosome-binding ATPase YchF |
| SB021 contig 1 | LEJJMMNP_01393 | CDS | 1296 |  |  |  | Hypothetical protein |
| SB021 contig 1 | LEJJMMNP_01394 | CDS | 759 | *ycfH* | 3.1.-.- | COG0084 | Putative metal-dependent hydrolase YcfH |
| SB021 contig 1 | LEJJMMNP_01395 | CDS | 375 | *acpS* | 2.7.8.7 | COG0736 | Holo-[acyl-carrier-protein] synthase |
| SB021 contig 1 | LEJJMMNP_01396 | CDS | 1098 | *prfB* |  |  | Peptide chain release factor 2 |
| SB021 contig 1 | LEJJMMNP_01397 | CDS | 1512 | *gltX* | 6.1.1.17 | COG0008 | Glutamate—tRNA ligase |
| SB021 contig 1 | LEJJMMNP_01398 | CDS | 666 |  |  |  | Hypothetical protein |
| SB021 contig 1 | LEJJMMNP_01399 | CDS | 312 | *trxA* |  | COG0526 | Thioredoxin |
| SB021 contig 1 | LEJJMMNP_01400 | CDS | 1041 | *kdgK* | 2.7.1.45 |  | 2-dehydro-3-deoxygluconokinase |
| SB021 contig 1 | LEJJMMNP_01401 | CDS | 636 | *kdgA* |  | COG0800 | KHG/KDPG aldolase |
| SB021 contig 1 | LEJJMMNP_01402 | CDS | 1362 |  |  |  | Hypothetical protein |
| SB021 contig 1 | LEJJMMNP_01403 | CDS | 1188 | *patB_2* | 4.4.1.13 | COG1168 | Cystathionine beta-lyase PatB |
| SB021 contig 1 | LEJJMMNP_01404 | CDS | 1197 |  |  |  | Hypothetical protein |
| SB021 contig 1 | LEJJMMNP_01405 | CDS | 738 | *prpR* |  | COG1221 | Propionate catabolism operon regulatory protein |
| SB021 contig 1 | LEJJMMNP_01406 | CDS | 108 |  |  |  | Hypothetical protein |
| SB021 contig 1 | LEJJMMNP_01407 | CDS | 672 |  |  |  | Hypothetical protein |
| SB021 contig 1 | LEJJMMNP_01408 | CDS | 369 |  |  |  | Hypothetical protein |
| SB021 contig 1 | LEJJMMNP_01409 | CDS | 831 |  |  |  | Iron-sulfur cluster carrier protein |
| SB021 contig 1 | LEJJMMNP_01410 | CDS | 846 |  |  |  | Iron-sulfur cluster carrier protein |
| SB021 contig 1 | LEJJMMNP_01411 | CDS | 1914 | *thrZ* | 6.1.1.3 | COG0441 | Threonine--tRNA ligase 2 |
| SB021 contig 1 | LEJJMMNP_01412 | CDS | 3663 |  |  |  | Hypothetical protein |
| SB021 contig 1 | LEJJMMNP_01413 | CDS | 318 |  |  |  | Thioredoxin C-1 |
| SB021 contig 1 | LEJJMMNP_01414 | CDS | 681 | *tsaB* |  | COG1214 | tRNA threonylcarbamoyladenosine biosynthesis protein TsaB |
| SB021 contig 1 | LEJJMMNP_01415 | CDS | 465 | *tsaE* |  | COG0802 | tRNA threonylcarbamoyladenosine biosynthesis protein TsaE |
| SB021 contig 1 | LEJJMMNP_01416 | CDS | 462 |  | 2.7.7.70 | COG2870 | D-beta-D-heptose 1-phosphate adenylyltransferase |
| SB021 contig 1 | LEJJMMNP_01417 | CDS | 876 |  |  |  | Hypothetical protein |
| SB021 contig 1 | LEJJMMNP_01418 | CDS | 318 | *manP_2* |  | COG1299 | PTS system mannose-specific EIIBCA component |
| SB021 contig 1 | LEJJMMNP_01419 | CDS | 1059 | *manP_2* |  | COG1299 | PTS system mannose-specific EIIBCA component |
| SB021 contig 1 | LEJJMMNP_01420 | CDS | 441 | *mngA* |  | COG1299 | PTS system 2-O-alpha-mannosyl-D-glycerate-specific EIIABC component |
| SB021 contig 1 | LEJJMMNP_01421 | CDS | 2058 |  |  |  | Hypothetical protein |
| SB021 contig 1 | LEJJMMNP_01422 | CDS | 861 | *mscS* |  | COG0668 | Small-conductance mechanosensitive channel |
| SB021 contig 1 | LEJJMMNP_01423 | tRNA | 77 |  |  |  | tRNA-Asp(gtc) |
| SB021 contig 1 | LEJJMMNP_01424 | tRNA | 76 |  |  |  | tRNA-Val(tac) |
| SB021 contig 1 | LEJJMMNP_01425 | tRNA | 85 |  |  |  | tRNA-Tyr(gta) |
| SB021 contig 1 | LEJJMMNP_01426 | tRNA | 75 |  |  |  | tRNA-Glu(ttc) |
| SB021 contig 1 | LEJJMMNP_01427 | tRNA | 76 |  |  |  | tRNA-Thr(tgt) |
| SB021 contig 1 | LEJJMMNP_01428 | CDS | 792 | *minD* |  | COG2894 | Septum site-determining protein MinD |
| SB021 contig 1 | LEJJMMNP_01429 | CDS | 678 | *minC* |  | COG0850 | Septum site-determining protein MinC |
| SB021 contig 1 | LEJJMMNP_01430 | CDS | 1047 | *melR* |  | COG1609 | HTH-type transcriptional repressor MelR |
| SB021 contig 1 | LEJJMMNP_01431 | CDS | 1368 | *melB_2* |  | COG2211 | Melibiose carrier protein |
| SB021 contig 1 | LEJJMMNP_01432 | CDS | 3027 | *cbgA* | 3.2.1.23 |  | Beta-galactosidase |
| SB021 contig 1 | LEJJMMNP_01433 | CDS | 360 | *mgsR* |  | COG1393 | Regulatory protein MgsR |
| SB021 contig 1 | LEJJMMNP_01434 | CDS | 1332 | *gltP* |  | COG1301 | Proton/glutamate-aspartate symporter |
| SB021 contig 1 | LEJJMMNP_01435 | CDS | 1359 | *alsT_4* |  | COG1115 | Amino-acid carrier protein AlsT |
| SB021 contig 1 | LEJJMMNP_01436 | CDS | 717 | *birA_2* | 6.3.4.15 | COG0340 | Bifunctional ligase/repressor BirA |
| SB021 contig 1 | LEJJMMNP_01437 | CDS | 783 |  |  |  | Hypothetical protein |
| SB021 contig 1 | LEJJMMNP_01438 | CDS | 390 | *panD* | 4.1.1.11 | COG0853 | Aspartate 1-decarboxylase |
| SB021 contig 1 | LEJJMMNP_01439 | CDS | 840 | *panC* | 6.3.2.1 | COG0414 | Pantothenate synthetase |
| SB021 contig 1 | LEJJMMNP_01440 | CDS | 930 | *panS* |  | COG0385 | Pantothenate precursors transporter PanS |
| SB021 contig 1 | LEJJMMNP_01441 | CDS | 1284 |  |  | COG1055 | Putative transporter |
| SB021 contig 1 | LEJJMMNP_01442 | CDS | 930 |  |  |  | Hypothetical protein |
| SB021 contig 1 | LEJJMMNP_01443 | CDS | 1278 |  |  | COG1055 | Putative transporter |
| SB021 contig 1 | LEJJMMNP_01444 | CDS | 2517 |  |  |  | Hypothetical protein |
| SB021 contig 1 | LEJJMMNP_01445 | CDS | 549 | *puuR_1* |  |  | HTH-type transcriptional regulator PuuR |
| SB021 contig 1 | LEJJMMNP_01446 | CDS | 1203 | *pncC* | 3.5.1.42 | COG1058 | Nicotinamide-nucleotide amidohydrolase PncC |
| SB021 contig 1 | LEJJMMNP_01447 | CDS | 504 | *pgpA* | 3.1.3.27 | COG1267 | Phosphatidylglycerophosphatase A |
| SB021 contig 1 | LEJJMMNP_01448 | CDS | 2166 |  |  |  | Hypothetical protein |
| SB021 contig 1 | LEJJMMNP_01449 | CDS | 585 | *coaE* | 2.7.1.24 | COG0237 | Dephospho-CoA kinase |
| SB021 contig 1 | LEJJMMNP_01450 | tRNA | 76 |  |  |  | tRNA-Asn(gtt) |
| SB021 contig 1 | LEJJMMNP_01451 | rRNA | 95 |  |  |  | 5S ribosomal RNA |
| SB021 contig 1 | LEJJMMNP_01452 | rRNA | 2903 |  |  |  | 23S ribosomal RNA |
| SB021 contig 1 | LEJJMMNP_01453 | rRNA | 1511 |  |  |  | 16S ribosomal RNA |
| SB021 contig 1 | LEJJMMNP_01454 | CDS | 1143 | *dacF* | 3.4.16.4 | COG1686 | D-alanyl-D-alanine carboxypeptidase DacF |
| SB021 contig 1 | LEJJMMNP_01455 | CDS | 375 | *iscU* |  | COG0822 | Iron-sulfur cluster assembly scaffold protein IscU |
| SB021 contig 1 | LEJJMMNP_01456 | CDS | 1167 | *nifS* | 2.8.1.7 | COG1104 | Cysteine desulfurase NifS |
| SB021 contig 1 | LEJJMMNP_01457 | CDS | 648 | *nth* | 4.2.99.18 | COG0177 | Endonuclease III |
| SB021 contig 1 | LEJJMMNP_01458 | CDS | 465 |  |  |  | Hypothetical protein |
| SB021 contig 1 | LEJJMMNP_01459 | CDS | 1221 | *tyrS* | 6.1.1.1 |  | Tyrosine—tRNA ligase |
| SB021 contig 1 | LEJJMMNP_01460 | CDS | 870 |  |  |  | Hypothetical protein |
| SB021 contig 1 | LEJJMMNP_01461 | CDS | 1281 | *secY* |  | COG0201 | Protein translocase subunit SecY |
| SB021 contig 1 | LEJJMMNP_01462 | CDS | 480 | *rplO* |  | COG0200 | 50S ribosomal protein L15 |
| SB021 contig 1 | LEJJMMNP_01463 | CDS | 183 | *rpmD* |  | COG1841 | 50S ribosomal protein L30 |
| SB021 contig 1 | LEJJMMNP_01464 | CDS | 504 | *rpsE* |  | COG0098 | 30S ribosomal protein S5 |
| SB021 contig 1 | LEJJMMNP_01465 | CDS | 369 | *rplR* |  |  | 50S ribosomal protein L18 |
| SB021 contig 1 | LEJJMMNP_01466 | CDS | 534 | *rplF* |  |  | 50S ribosomal protein L6 |
| SB021 contig 1 | LEJJMMNP_01467 | CDS | 396 | *rpsH* |  |  | 30S ribosomal protein S8 |
| SB021 contig 1 | LEJJMMNP_01468 | CDS | 288 | *rpsN* |  | COG0199 | 30S ribosomal protein S14 |
| SB021 contig 1 | LEJJMMNP_01469 | CDS | 552 | *rplE* |  |  | 50S ribosomal protein L5 |
| SB021 contig 1 | LEJJMMNP_01470 | CDS | 342 | *rplX* |  |  | 50S ribosomal protein L24 |
| SB021 contig 1 | LEJJMMNP_01471 | CDS | 369 | *rplN* |  | COG0093 | 50S ribosomal protein L14 |
| SB021 contig 1 | LEJJMMNP_01472 | CDS | 252 | *rpsQ* |  | COG0186 | 30S ribosomal protein S17 |
| SB021 contig 1 | LEJJMMNP_01473 | CDS | 183 | *rpmC* |  |  | 50S ribosomal protein L29 |
| SB021 contig 1 | LEJJMMNP_01474 | CDS | 423 | *rplP* |  | COG0197 | 50S ribosomal protein L16 |
| SB021 contig 1 | LEJJMMNP_01475 | CDS | 657 | *rpsC* |  | COG0092 | 30S ribosomal protein S3 |
| SB021 contig 1 | LEJJMMNP_01476 | CDS | 333 | *rplV* |  | COG0091 | 50S ribosomal protein L22 |
| SB021 contig 1 | LEJJMMNP_01477 | CDS | 273 | *rpsS* |  | COG0185 | 30S ribosomal protein S19 |
| SB021 contig 1 | LEJJMMNP_01478 | CDS | 831 | *rplB* |  |  | 50S ribosomal protein L2 |
| SB021 contig 1 | LEJJMMNP_01479 | CDS | 285 | *rplW* |  |  | 50S ribosomal protein L23 |
| SB021 contig 1 | LEJJMMNP_01480 | CDS | 633 | *rplD* |  |  | 50S ribosomal protein L4 |
| SB021 contig 1 | LEJJMMNP_01481 | CDS | 627 | *rplC* |  | COG0087 | 50S ribosomal protein L3 |
| SB021 contig 1 | LEJJMMNP_01482 | CDS | 309 | *rpsJ* |  | COG0051 | 30S ribosomal protein S10 |
| SB021 contig 1 | LEJJMMNP_01483 | CDS | 993 | *rnfB* | 7.2.1.2 | COG2878 | Na(+)-translocating ferredoxin:NAD(+) oxidoreductase complex subunit B |
| SB021 contig 1 | LEJJMMNP_01484 | CDS | 585 | *rnfA* | 7.2.1.2 | COG4657 | Na(+)-translocating ferredoxin:NAD(+) oxidoreductase complex subunit A |
| SB021 contig 1 | LEJJMMNP_01485 | CDS | 603 | *rnfE* | 7.2.1.2 | COG4660 | Na(+)-translocating ferredoxin:NAD(+) oxidoreductase complex subunit E |
| SB021 contig 1 | LEJJMMNP_01486 | CDS | 537 | *rnfG* | 7.2.1.2 | COG4659 | Na(+)-translocating ferredoxin:NAD(+) oxidoreductase complex subunit G |
| SB021 contig 1 | LEJJMMNP_01487 | CDS | 942 | *rnfD* | 7.1.1.- | COG4658 | Proton-translocating ferredoxin:NAD(+) oxidoreductase complex subunit D |
| SB021 contig 1 | LEJJMMNP_01488 | CDS | 1308 | *rnfC* | 7.1.1.- | COG4656 | Proton-translocating ferredoxin:NAD(+) oxidoreductase complex subunit C |
| SB021 contig 1 | LEJJMMNP_01489 | CDS | 570 | *pth* | 3.1.1.29 |  | Peptidyl-tRNA hydrolase |
| SB021 contig 1 | LEJJMMNP_01490 | CDS | 1206 | *sdaA* | 4.3.1.17 | COG1760 | L-serine dehydratase 1 |
| SB021 contig 1 | LEJJMMNP_01491 | CDS | 2385 | *pheT* | 6.1.1.20 | COG0072 | Phenylalanine--tRNA ligase beta subunit |
| SB021 contig 1 | LEJJMMNP_01492 | CDS | 1020 | *pheS* | 6.1.1.20 | COG0016 | Phenylalanine--tRNA ligase alpha subunit |
| SB021 contig 1 | LEJJMMNP_01493 | CDS | 495 |  | 3.1.4- |  | Putative metallophosphoesterase MG207 |
| SB021 contig 1 | LEJJMMNP_01494 | CDS | 798 |  |  |  | Hypothetical protein |
| SB021 contig 1 | LEJJMMNP_01495 | CDS | 2604 | *gyrA* | 5.6.2.2 | COG0188 | DNA gyrase subunit A |
| SB021 contig 1 | LEJJMMNP_01496 | CDS | 1182 | *gyrB_1* | 5.6.2.2 | COG0187 | DNA gyrase subunit B |
| SB021 contig 1 | LEJJMMNP_01497 | CDS | 360 | *gyrB_2* | 5.6.2.2 | COG0187 | DNA gyrase subunit B |
| SB021 contig 1 | LEJJMMNP_01498 | CDS | 543 |  |  |  | Hypothetical protein |
| SB021 contig 1 | LEJJMMNP_01499 | CDS | 759 | *recF_1* |  | COG1195 | DNA replication and repair protein RecF |
| SB021 contig 1 | LEJJMMNP_01500 | CDS | 330 | *recF_2* |  |  | DNA replication and repair protein RecF |
| SB021 contig 1 | LEJJMMNP_01501 | CDS | 207 |  |  |  | Hypothetical protein |
| SB021 contig 1 | LEJJMMNP_01502 | CDS | 393 |  |  |  | Hypothetical protein |
| SB021 contig 1 | LEJJMMNP_01503 | CDS | 423 |  |  |  | Hypothetical protein |
| SB021 contig 1 | LEJJMMNP_01504 | CDS | 984 |  |  |  | Hypothetical protein |
| SB021 contig 1 | LEJJMMNP_01505 | CDS | 846 |  |  |  | Hypothetical protein |
| SB021 contig 1 | LEJJMMNP_01506 | CDS | 777 | *opuBA* |  | COG1125 | Choline transport ATP-binding protein OpuBA |
| SB021 contig 1 | LEJJMMNP_01507 | CDS | 1341 |  |  |  | Hypothetical protein |
| SB021 contig 1 | LEJJMMNP_01508 | CDS | 543 | *ywqN* | 1.-.-.- | COG0655 | Putative NAD(P)H-dependent FMN-containing oxidoreductase YwqN |
| SB021 contig 1 | LEJJMMNP_01509 | CDS | 897 | *pdxY* | 2.7.1.35 | COG2240 | Pyridoxal kinase PdxY |
| SB021 contig 1 | LEJJMMNP_01510 | CDS | 1428 | *hisC_3* | 2.6.1.9 |  | Histidinol-phosphate aminotransferase |
| SB021 contig 1 | LEJJMMNP_01511 | CDS | 1380 |  |  |  | Hypothetical protein |
| SB021 contig 1 | LEJJMMNP_01512 | CDS | 795 | *mtnN* | 3.2.2.9 |  | 5'-methylthioadenosine/S-adenosylhomocysteine nucleosidase |
| SB021 contig 1 | LEJJMMNP_01513 | CDS | 1194 |  | 4.4.1.11 | COG0626 | L-methionine gamma-lyase |
| SB021 contig 1 | LEJJMMNP_01514 | CDS | 1107 | *drdK* | 2.7.1.- |  | 5-deoxyribose kinase |
| SB021 contig 1 | LEJJMMNP_01515 | CDS | 96 |  |  |  | Hypothetical protein |
| SB021 contig 1 | LEJJMMNP_01516 | CDS | 861 | *mtnA* | 5.3.1.23 | COG0182 | Methylthioribose-1-phosphate isomerase |
| SB021 contig 1 | LEJJMMNP_01517 | CDS | 1296 | *mleN* |  | COG1757 | Malate-2H(+)/Na(+)-lactate antiporter |
| SB021 contig 1 | LEJJMMNP_01518 | CDS | 666 | *drdA* | 4.1.2.- |  | 5-deoxy-D-ribulose 1-phosphate aldolase |
| SB021 contig 1 | LEJJMMNP_01519 | CDS | 1170 | *adh2* | 1.1.1.192 | COG1979 | Long-chain-alcohol dehydrogenase 2 |
| SB021 contig 1 | LEJJMMNP_01520 | CDS | 765 | *ydjF* |  | COG1349 | Putative HTH-type transcriptional regulator YdjF |
| SB021 contig 1 | LEJJMMNP_01521 | CDS | 633 |  |  |  | Hypothetical protein |
| SB021 contig 1 | LEJJMMNP_01522 | CDS | 453 | *murJ_4* |  |  | Lipid II flippase MurJ |
| SB021 contig 1 | LEJJMMNP_01523 | CDS | 264 |  |  |  | Hypothetical protein |
| SB021 contig 1 | LEJJMMNP_01524 | tRNA | 75 |  |  |  | tRNA-Gln(ttg) |
| SB021 contig 1 | LEJJMMNP_01525 | CDS | 297 | *spoVG* |  | COG2088 | Putative septation protein SpoVG |
| SB021 contig 1 | LEJJMMNP_01526 | CDS | 861 | *ispE* | 2.7.1.148 | COG1947 | 4-diphosphocytidyl-2-C-methyl-D-erythritol kinase |
| SB021 contig 1 | LEJJMMNP_01527 | CDS | 294 |  |  |  | Hypothetical protein |
| SB021 contig 1 | LEJJMMNP_01528 | CDS | 768 | *mazG* | 3.6.1.8 | COG1694 | Nucleoside triphosphate pyrophosphohydrolase |
| SB021 contig 1 | LEJJMMNP_01529 | CDS | 2997 | *mfd* | 3.6.4.- |  | Transcription-repair-coupling factor |
| SB021 contig 1 | LEJJMMNP_01530 | CDS | 324 | *yybR* |  | COG1733 | Putative HTH-type transcriptional regulator YybR |
| SB021 contig 1 | LEJJMMNP_01531 | CDS | 405 |  |  |  | Hypothetical protein |
| SB021 contig 1 | LEJJMMNP_01532 | CDS | 702 |  |  |  | IS1595 family transposase ISBth19 |
| SB021 contig 1 | LEJJMMNP_01533 | CDS | 576 | *folE* | 3.5.4.16 | COG0302 | GTP cyclohydrolase 1 |
| SB021 contig 1 | LEJJMMNP_01534 | CDS | 828 | *sulD* |  | COG0801 | Bifunctional folate synthesis protein |
| SB021 contig 1 | LEJJMMNP_01535 | CDS | 819 | *folP* | 2.5.1.15 |  | Dihydropteroate synthase |
| SB021 contig 1 | LEJJMMNP_01536 | CDS | 840 | *hbd* | 1.1.1.157 | COG1250 | 3-hydroxybutyryl-CoA dehydrogenase |
| SB021 contig 1 | LEJJMMNP_01537 | CDS | 777 | *crt* | 4.2.1.150 | COG1024 | Short-chain-enoyl-CoA hydratase |
| SB021 contig 1 | LEJJMMNP_01538 | CDS | 393 |  |  |  | Hypothetical protein |
| SB021 contig 1 | LEJJMMNP_01539 | CDS | 1113 |  |  |  | Hypothetical protein |
| SB021 contig 1 | LEJJMMNP_01540 | CDS | 1017 |  |  |  | Hypothetical protein |
| SB021 contig 1 | LEJJMMNP_01541 | CDS | 1593 | *rbsA* | 7.5.2.7 | COG1129 | Ribose import ATP-binding protein RbsA |
| SB021 contig 1 | LEJJMMNP_01542 | CDS | 1176 |  |  |  | Hypothetical protein |
| SB021 contig 1 | LEJJMMNP_01543 | CDS | 1170 |  |  |  | Hypothetical protein |
| SB021 contig 1 | LEJJMMNP_01544 | CDS | 1479 |  |  |  | IS1182 family transposase ISFnu2 |
| SB021 contig 1 | LEJJMMNP_01545 | CDS | 1203 | *fprA1* | 1.6.3.4 | COG0426 | Flavo-diiron protein FprA1 |
| SB021 contig 1 | LEJJMMNP_01546 | CDS | 1902 | *caiA* | 1.3.8.13 |  | Crotonobetainyl-CoA reductase |
| SB021 contig 1 | LEJJMMNP_01547 | CDS | 1656 | *phoR* | 2.7.13.3 | COG0642 | Alkaline phosphatase synthesis sensor protein PhoR |
| SB021 contig 1 | LEJJMMNP_01548 | CDS | 678 | *phoP_2* |  | COG0745 | Alkaline phosphatase synthesis transcriptional regulatory protein PhoP |
| SB021 contig 1 | LEJJMMNP_01549 | CDS | 714 |  |  |  | Hypothetical protein |
| SB021 contig 1 | LEJJMMNP_01550 | CDS | 762 | *pstB3* | 7.3.2.1 | COG1117 | Phosphate import ATP-binding protein PstB 3 |
| SB021 contig 1 | LEJJMMNP_01551 | CDS | 843 |  |  |  | Hypothetical protein |
| SB021 contig 1 | LEJJMMNP_01552 | CDS | 879 | *pstC* |  | COG0573 | Phosphate transport system permease protein PstC |
| SB021 contig 1 | LEJJMMNP_01553 | CDS | 378 |  |  |  | Hypothetical protein |
| SB021 contig 1 | LEJJMMNP_01554 | CDS | 1320 | *sasA_5* | 2.7.-.- |  | Adaptive-response sensory-kinase SasA |
| SB021 contig 1 | LEJJMMNP_01555 | CDS | 663 | *walR* |  | COG0745 | Transcriptional regulatory protein WalR |
| SB021 contig 1 | LEJJMMNP_01556 | CDS | 609 |  | 2.3.1.- | COG0110 | Putative acetyltransferase |
| SB021 contig 1 | LEJJMMNP_01557 | CDS | 477 | *ispF* | 4.6.1.12 | COG0245 | 2-C-methyl-D-erythritol 2,4-cyclodiphosphate synthase |
| SB021 contig 1 | LEJJMMNP_01558 | CDS | 987 | *rfaE* | 2.7.1.167 | COG2870 | D-beta-D-heptose 7-phosphate kinase |
| SB021 contig 1 | LEJJMMNP_01559 | CDS | 1626 | *sctC* |  |  | Type 3 secretion system secretin |
| SB021 contig 1 | LEJJMMNP_01560 | CDS | 810 |  |  |  | Hypothetical protein |
| SB021 contig 1 | LEJJMMNP_01561 | CDS | 903 |  |  |  | Hypothetical protein |
| SB021 contig 1 | LEJJMMNP_01562 | CDS | 633 |  |  |  | Hypothetical protein |
| SB021 contig 1 | LEJJMMNP_01563 | CDS | 471 |  |  |  | Hypothetical protein |
| SB021 contig 1 | LEJJMMNP_01564 | CDS | 447 |  |  |  | Hypothetical protein |
| SB021 contig 1 | LEJJMMNP_01565 | CDS | 474 |  |  |  | Hypothetical protein |
| SB021 contig 1 | LEJJMMNP_01566 | CDS | 504 |  |  |  | Hypothetical protein |
| SB021 contig 1 | LEJJMMNP_01567 | CDS | 1164 | *epsF_2* |  | COG1459 | Type II secretion system protein F |
| SB021 contig 1 | LEJJMMNP_01568 | CDS | 1275 | *xpsE* |  | COG2804 | Type II secretion system protein E |
| SB021 contig 1 | LEJJMMNP_01569 | CDS | 228 |  |  |  | Hypothetical protein |
| SB021 contig 1 | LEJJMMNP_01570 | CDS | 447 |  |  |  | Hypothetical protein |
| SB021 contig 1 | LEJJMMNP_01571 | CDS | 753 |  |  |  | Hypothetical protein |
| SB021 contig 1 | LEJJMMNP_01572 | CDS | 1212 | *sdaB* | 4.3.1.17 | COG1760 | L-serine dehydratase 2 |
| SB021 contig 1 | LEJJMMNP_01573 | CDS | 1014 |  |  |  | Hypothetical protein |
| SB021 contig 1 | LEJJMMNP_01574 | CDS | 537 | *puuR_2* |  |  | HTH-type transcriptional regulator PuuR |
| SB021 contig 1 | LEJJMMNP_01575 | CDS | 519 |  |  |  | Hypothetical protein |
| SB021 contig 1 | LEJJMMNP_01576 | CDS | 261 |  |  |  | Hypothetical protein |
| SB021 contig 1 | LEJJMMNP_01577 | CDS | 459 |  |  |  | Hypothetical protein |
| SB021 contig 1 | LEJJMMNP_01578 | CDS | 495 | *mog* | 2.7.7.75 | COG0521 | Molybdopterin adenylyltransferase |
| SB021 contig 1 | LEJJMMNP_01579 | CDS | 666 |  |  |  | Hypothetical protein |
| SB021 contig 1 | LEJJMMNP_01580 | CDS | 735 | *modA* |  | COG0725 | Molybdate-binding protein ModA |
| SB021 contig 1 | LEJJMMNP_01581 | CDS | 471 | *moaC* | 4.6.1.17 | COG0315 | Cyclic pyranopterin monophosphate synthase |
| SB021 contig 1 | LEJJMMNP_01582 | CDS | 1020 | *cinA* |  |  | Putative competence-damage inducible protein |
| SB021 contig 1 | LEJJMMNP_01583 | CDS | 438 |  |  |  | Hypothetical protein |
| SB021 contig 1 | LEJJMMNP_01584 | CDS | 828 |  |  |  | Hypothetical protein |
| SB021 contig 1 | LEJJMMNP_01585 | CDS | 966 | *moaA_2* | 4.1.99.22 | COG2896 | GTP 3',8-cyclase |
| SB021 contig 1 | LEJJMMNP_01586 | CDS | 819 |  |  |  | Hypothetical protein |
| SB021 contig 1 | LEJJMMNP_01587 | CDS | 1122 | *mobA* | 2.7.7.77 |  | Molybdenum cofactor guanylyltransferase |
| SB021 contig 1 | LEJJMMNP_01588 | CDS | 2571 |  |  |  | Hypothetical protein |
| SB021 contig 1 | LEJJMMNP_01589 | CDS | 1377 | *hyuA* | 3.5.2.- | COG0044 | D-phenylhydantoinase |
| SB021 contig 1 | LEJJMMNP_01590 | CDS | 1680 |  |  |  | Hypothetical protein |
| SB021 contig 1 | LEJJMMNP_01591 | CDS | 1338 | *uacT* |  | COG2233 | Uric acid transporter UacT |
| SB021 contig 1 | LEJJMMNP_01592 | CDS | 1326 | *ssnA* | 3.-.-.- | COG0402 | Putative aminohydrolase SsnA |
| SB021 contig 1 | LEJJMMNP_01593 | CDS | 519 | *hndA* | 1.12.1.3 | COG1905 | NADP-reducing hydrogenase subunit HndA |
| SB021 contig 1 | LEJJMMNP_01594 | CDS | 1785 | *hndC* | 1.12.1.3 | COG1145 | NADP-reducing hydrogenase subunit HndC |
| SB021 contig 1 | LEJJMMNP_01595 | CDS | 1758 | *hndD* | 1.12.1.3 | COG3383 | NADP-reducing hydrogenase subunit HndD |
| SB021 contig 1 | LEJJMMNP_01596 | CDS | 1716 | *norR_4* |  |  | Anaerobic nitric oxide reductase transcription regulator NorR |
| SB021 contig 1 | LEJJMMNP_01597 | CDS | 1221 | *dpaL* | 4.3.1.15 | COG1171 | Diaminopropionate ammonia-lyase |
| SB021 contig 1 | LEJJMMNP_01598 | CDS | 1185 | *argE* | 3.5.1.16 | COG0624 | Acetylornithine deacetylase |
| SB021 contig 1 | LEJJMMNP_01599 | CDS | 1593 | *dan* | 3.5.1.81 |  | D-aminoacylase |
| SB021 contig 1 | LEJJMMNP_01600 | CDS | 1479 |  |  |  | IS1182 family transposase ISFnu2 |
| SB021 contig 1 | LEJJMMNP_01601 | CDS | 1767 | *norR_5* |  |  | Anaerobic nitric oxide reductase transcription regulator NorR |
| SB021 contig 1 | LEJJMMNP_01602 | CDS | 831 | *pstS* |  | COG0226 | Phosphate-binding protein PstS |
| SB021 contig 1 | LEJJMMNP_01603 | CDS | 282 | *hup_2* |  |  | DNA-binding protein HU |
| SB021 contig 1 | LEJJMMNP_01604 | CDS | 1860 | *mnmG_2* |  |  | tRNA uridine 5-carboxymethylaminomethyl modification enzyme MnmG |
| SB021 contig 1 | LEJJMMNP_01605 | CDS | 1368 | *mnmE_2* | 3.6.-.- | COG0486 | tRNA modification GTPase MnmE |
| SB021 contig 1 | LEJJMMNP_01606 | CDS | 798 |  |  |  | Hypothetical protein |
| SB021 contig 1 | LEJJMMNP_01607 | CDS | 606 | *yidC2* |  | COG0706 | Membrane protein insertase YidC 2 |
| SB021 contig 1 | LEJJMMNP_01608 | CDS | 288 | *yidD* |  |  | Putative membrane protein insertion efficiency factor |
| SB021 contig 1 | LEJJMMNP_01609 | CDS | 342 | *rnpA* | 3.1.26.5 | COG0594 | Ribonuclease P protein component |
| SB021 contig 1 | LEJJMMNP_01610 | CDS | 144 | *rpmH* |  | COG0230 | 50S ribosomal protein L34 |
| SB021 contig 1 | LEJJMMNP_01611 | CDS | 1860 |  |  |  | Hypothetical protein |
| SB021 contig 1 | LEJJMMNP_01612 | rRNA | 1511 |  |  |  | 16S ribosomal RNA |
| SB021 contig 1 | LEJJMMNP_01613 | tRNA | 77 |  |  |  | tRNA-Ile(gat) |
| SB021 contig 1 | LEJJMMNP_01614 | tRNA | 77 |  |  |  | tRNA-Ala(tgc) |
| SB021 contig 1 | LEJJMMNP_01615 | rRNA | 2903 |  |  |  | 23S ribosomal RNA |
| SB021 contig 1 | LEJJMMNP_01616 | rRNA | 95 |  |  |  | 5S ribosomal RNA |
| SB021 contig 1 | LEJJMMNP_01617 | tRNA | 76 |  |  |  | tRNA-Asn(gtt) |
| SB021 contig 1 | LEJJMMNP_01618 | CDS | 351 | *rplS* |  | COG0335 | 50S ribosomal protein L19 |
| SB021 contig 1 | LEJJMMNP_01619 | CDS | 933 |  |  |  | Hypothetical protein |
| SB021 contig 1 | LEJJMMNP_01620 | CDS | 405 |  |  |  | Hypothetical protein |
| SB021 contig 1 | LEJJMMNP_01621 | CDS | 1434 | *purB* | 4.3.2.2 | COG0015 | Adenylosuccinate lyase |
| SB021 contig 1 | LEJJMMNP_01622 | CDS | 543 |  |  |  | Hypothetical protein |
| SB021 contig 1 | LEJJMMNP_01623 | CDS | 1359 | *glmM* | 5.4.2.10 | COG1109 | Phosphoglucosamine mutase |
| SB021 contig 1 | LEJJMMNP_01624 | CDS | 720 | *cmoA* | 2.1.3.- |  | Carboxy-S-adenosyl-L-methionine synthase |
| SB021 contig 1 | LEJJMMNP_01625 | CDS | 213 |  |  |  | Hypothetical protein |
| SB021 contig 1 | LEJJMMNP_01626 | CDS | 375 |  |  |  | Hypothetical protein |
| SB021 contig 1 | LEJJMMNP_01627 | CDS | 810 | *atpB* |  | COG0356 | ATP synthase subunit a |
| SB021 contig 1 | LEJJMMNP_01628 | CDS | 291 | *atpE* |  |  | ATP synthase subunit c, sodium ion specific |
| SB021 contig 1 | LEJJMMNP_01629 | CDS | 507 | *atpF* |  |  | ATP synthase subunit b, sodium ion specific |
| SB021 contig 1 | LEJJMMNP_01630 | CDS | 531 | *atpH* |  |  | ATP synthase subunit delta, sodium ion specific |
| SB021 contig 1 | LEJJMMNP_01631 | CDS | 1503 | *atpA* | 7.1.2.2 | COG0056 | ATP synthase subunit alpha |
| SB021 contig 1 | LEJJMMNP_01632 | CDS | 852 | *atpG* |  |  | ATP synthase gamma chain, sodium ion specific |
| SB021 contig 1 | LEJJMMNP_01633 | CDS | 1401 | *atpD* | 7.1.2.2 | COG0055 | ATP synthase subunit beta |
| SB021 contig 1 | LEJJMMNP_01634 | CDS | 408 | *atpC* |  |  | ATP synthase epsilon chain, sodium ion specific |
| SB021 contig 1 | LEJJMMNP_01635 | CDS | 447 |  |  |  | Hypothetical protein |
| SB021 contig 1 | LEJJMMNP_01636 | CDS | 1350 | *pgi* | 5.3.1.9 | COG0166 | Glucose-6-phosphate isomerase |
| SB021 contig 1 | LEJJMMNP_01637 | CDS | 1440 | *apeA* | 3.4.11.- | COG1362 | Putative M18 family aminopeptidase 1 |
| SB021 contig 2 | OKKMCADK_00001 | CDS | 1194 | *rffG* | 4.2.1.46 | COG1088 | dTDP-glucose 4,6-dehydratase 2 |
| SB021 contig 2 | OKKMCADK_00002 | CDS | 840 | *rfbD* | 1.1.1.133 | COG1091 | dTDP-4-dehydrorhamnose reductase |
| SB021 contig 2 | OKKMCADK_00003 | CDS | 576 | *rmlC* | 5.1.3.13 | COG1898 | dTDP-4-dehydrorhamnose 3,5-epimerase |
| SB021 contig 2 | OKKMCADK_00004 | CDS | 675 |  |  |  | Hypothetical protein |
| SB021 contig 2 | OKKMCADK_00005 | CDS | 867 | *rfbA* | 2.7.7.24 | COG1209 | Glucose-1-phosphate thymidylyltransferase 1 |
| SB021 contig 2 | OKKMCADK_00006 | CDS | 1179 |  |  |  | Hypothetical protein |
| SB021 contig 2 | OKKMCADK_00007 | CDS | 1455 | *rfbX* |  | COG2244 | Putative O-antigen transporter |
| SB021 contig 2 | OKKMCADK_00008 | CDS | 909 |  |  |  | Hypothetical protein |
| SB021 contig 2 | OKKMCADK_00009 | CDS | 1158 |  |  |  | Hypothetical protein |
| SB021 contig 2 | OKKMCADK_00010 | CDS | 936 |  |  |  | Hypothetical protein |
| SB021 contig 2 | OKKMCADK_00011 | CDS | 1017 | *epsJ* | 2.4.-.- | COG0463 | Putative glycosyltransferase EpsJ |
| SB021 contig 2 | OKKMCADK_00012 | CDS | 1155 | *epsD* | 2.4.-.- |  | Putative glycosyltransferase EpsD |
| SB021 contig 2 | OKKMCADK_00013 | CDS | 885 | *gnu_1* | 5.1.3.26 | COG0451 | N-acetyl-alpha-D-glucosaminyl-diphospho-ditrans,octacis-undecaprenol 4-epimerase |
| SB021 contig 2 | OKKMCADK_00014 | CDS | 681 | *wecA* | 2.7.8.40 |  | UDP-N-acetylgalactosamine-undecaprenyl-phosphate N-acetylgalactosaminephosphotransferase |
| SB021 contig 2 | OKKMCADK_00015 | CDS | 525 | *epsM* | 2.3.1.- | COG0110 | Putative acetyltransferase EpsM |
| SB021 contig 2 | OKKMCADK_00016 | CDS | 1947 | *fcl_1* | 1.1.1.271 |  | GDP-L-fucose synthase |
| SB021 contig 2 | OKKMCADK_00017 | CDS | 969 |  |  |  | IS3 family transposase ISFnu6 |
| SB021 contig 2 | OKKMCADK_00018 | CDS | 141 |  |  |  | Hypothetical protein |
| SB021 contig 2 | OKKMCADK_00019 | CDS | 666 |  |  |  | Hypothetical protein |
| SB021 contig 2 | OKKMCADK_00020 | CDS | 402 |  |  |  | Hypothetical protein |
| SB021 contig 2 | OKKMCADK_00021 | CDS | 423 |  |  |  | Hypothetical protein |
| SB021 contig 2 | OKKMCADK_00022 | CDS | 615 |  |  |  | Hypothetical protein |
| SB021 contig 2 | OKKMCADK_00023 | CDS | 453 |  |  |  | Hypothetical protein |
| SB021 contig 2 | OKKMCADK_00024 | CDS | 1191 |  |  |  | Hypothetical protein |
| SB021 contig 2 | OKKMCADK_00025 | CDS | 1074 | *fic* | 2.7.7.- | COG3177 | Protein adenylyltransferase SoFic |
| SB021 contig 2 | OKKMCADK_00026 | CDS | 1233 |  |  |  | Hypothetical protein |
| SB021 contig 2 | OKKMCADK_00027 | CDS | 177 |  |  |  | Hypothetical protein |
| SB021 contig 2 | OKKMCADK_00028 | CDS | 513 | *dinB* | 2.7.7.7 | COG0389 | DNA polymerase IV |
| SB021 contig 2 | OKKMCADK_00029 | CDS | 1554 |  |  |  | IS3 family transposase ISKpn38 |
| SB021 contig 2 | OKKMCADK_00030 | CDS | 549 | *dinB1* | 2.7.7.7 | COG0389 | DNA polymerase IV 1 |
| SB021 contig 2 | OKKMCADK_00031 | CDS | 240 |  |  |  | Hypothetical protein |
| SB021 contig 2 | OKKMCADK_00032 | CDS | 660 |  |  |  | Hypothetical protein |
| SB021 contig 2 | OKKMCADK_00033 | CDS | 1386 | *recF_1* |  |  | DNA replication and repair protein RecF |
| SB021 contig 2 | OKKMCADK_00034 | CDS | 732 |  |  |  | Hypothetical protein |
| SB021 contig 2 | OKKMCADK_00035 | CDS | 678 | *ppa* | 3.6.1.1 |  | Inorganic pyrophosphatase |
| SB021 contig 2 | OKKMCADK_00036 | CDS | 666 |  |  |  | Hypothetical protein |
| SB021 contig 2 | OKKMCADK_00037 | CDS | 1347 |  |  |  | Hypothetical protein |
| SB021 contig 2 | OKKMCADK_00038 | CDS | 354 |  |  |  | Hypothetical protein |
| SB021 contig 2 | OKKMCADK_00039 | CDS | 1311 | *recF_2* |  |  | DNA replication and repair protein RecF |
| SB021 contig 2 | OKKMCADK_00040 | CDS | 141 |  |  |  | Hypothetical protein |
| SB021 contig 2 | OKKMCADK_00041 | CDS | 270 |  |  |  | Hypothetical protein |
| SB021 contig 2 | OKKMCADK_00042 | CDS | 342 |  |  |  | Hypothetical protein |
| SB021 contig 2 | OKKMCADK_00043 | CDS | 1383 |  |  |  | Hypothetical protein |
| SB021 contig 2 | OKKMCADK_00044 | CDS | 2322 |  |  |  | Hypothetical protein |
| SB021 contig 2 | OKKMCADK_00045 | CDS | 1902 | *norR* |  |  | Anaerobic nitric oxide reductase transcription regulator NorR |
| SB021 contig 2 | OKKMCADK_00046 | CDS | 297 | *uxaA* | 4.2.1.7 | COG2721 | Altronate dehydratase |
| SB021 contig 2 | OKKMCADK_00047 | CDS | 1158 | *suyB* | 4.4.1.24 | COG2721 | (2R)-sulfolactate sulfo-lyase subunit beta |
| SB021 contig 2 | OKKMCADK_00048 | CDS | 1257 | *dtnK* | 2.7.1.219 | COG3395 | D-threonate kinase |
| SB021 contig 2 | OKKMCADK_00049 | CDS | 936 | *kdgT* |  |  | 2-keto-3-deoxygluconate permease |
| SB021 contig 2 | OKKMCADK_00050 | CDS | 1032 | *pdxA2_1* | 1.1.1.408 | COG1995 | D-threonate 4-phosphate dehydrogenase |
| SB021 contig 2 | OKKMCADK_00051 | CDS | 837 | *yvgN* | 1.1.1.- | COG0656 | Glyoxal reductase |
| SB021 contig 2 | OKKMCADK_00052 | CDS | 435 | *slyA* |  |  | Transcriptional regulator SlyA |
| SB021 contig 2 | OKKMCADK_00053 | CDS | 933 |  |  |  | Hypothetical protein |
| SB021 contig 2 | OKKMCADK_00054 | CDS | 1362 |  | 3.1.6.- |  | Ulvan-active sulfatase |
| SB021 contig 2 | OKKMCADK_00055 | CDS | 1536 |  |  |  | Hypothetical protein |
| SB021 contig 2 | OKKMCADK_00056 | CDS | 729 | *nanE* | 5.1.3.9 |  | Putative N-acetylmannosamine-6-phosphate 2-epimerase |
| SB021 contig 2 | OKKMCADK_00057 | CDS | 1575 | *ptsG* | 2.7.1.199 | COG1263 | PTS system glucose-specific EIICBA component |
| SB021 contig 2 | OKKMCADK_00058 | CDS | 720 | *mngR* |  | COG2188 | Mannosyl-D-glycerate transport/metabolism system repressor MngR |
| SB021 contig 2 | OKKMCADK_00059 | CDS | 309 | *ihfA_1* |  | COG0776 | Integration host factor subunit alpha |
| SB021 contig 2 | OKKMCADK_00060 | CDS | 1350 |  |  |  | Hypothetical protein |
| SB021 contig 2 | OKKMCADK_00061 | CDS | 735 | *artP* |  | COG0834 | Arginine-binding extracellular protein ArtP |
| SB021 contig 2 | OKKMCADK_00062 | CDS | 750 |  |  |  | Hypothetical protein |
| SB021 contig 2 | OKKMCADK_00063 | CDS | 1290 | *denK* | 2.7.1.220 | COG3395 | D-erythronate kinase |
| SB021 contig 2 | OKKMCADK_00064 | CDS | 1023 | *pdxA2_2* | 1.1.1.408 | COG1995 | D-threonate 4-phosphate dehydrogenase |
| SB021 contig 2 | OKKMCADK_00065 | CDS | 969 |  |  |  | Hypothetical protein |
| SB021 contig 2 | OKKMCADK_00066 | CDS | 1482 |  |  |  | Hypothetical protein |
| SB021 contig 2 | OKKMCADK_00067 | CDS | 486 |  |  |  | Hypothetical protein |
| SB021 contig 2 | OKKMCADK_00068 | CDS | 978 |  | 1.1.1.81 | COG0111 | Hydroxypyruvate reductase |
| SB021 contig 2 | OKKMCADK_00069 | CDS | 900 | *dapA* | 4.3.3.7 | COG0329 | 4-hydroxy-tetrahydrodipicolinate synthase |
| SB021 contig 2 | OKKMCADK_00070 | CDS | 1158 | *adh1* | 1.1.1.192 | COG1454 | Long-chain-alcohol dehydrogenase 1 |
| SB021 contig 2 | OKKMCADK_00071 | CDS | 678 | *gntR* |  |  | putative D-xylose utilization operon transcriptional repressor |
| SB021 contig 2 | OKKMCADK_00072 | CDS | 294 |  |  |  | IS3 family transposase IS1076 |
| SB021 contig 2 | OKKMCADK_00073 | CDS | 693 |  |  |  | IS3 family transposase IS1069 |
| SB021 contig 2 | OKKMCADK_00074 | CDS | 1443 |  |  |  | Hypothetical protein |
| SB021 contig 2 | OKKMCADK_00075 | CDS | 1227 |  | 2.6.1.1 | COG0436 | Aspartate aminotransferase |
| SB021 contig 2 | OKKMCADK_00076 | CDS | 231 |  |  |  | Hypothetical protein |
| SB021 contig 2 | OKKMCADK_00077 | CDS | 453 |  |  |  | Hypothetical protein |
| SB021 contig 2 | OKKMCADK_00078 | CDS | 720 |  |  |  | Hypothetical protein |
| SB021 contig 2 | OKKMCADK_00079 | CDS | 1203 |  |  |  | Hypothetical protein |
| SB021 contig 2 | OKKMCADK_00080 | CDS | 123 |  |  |  | Hypothetical protein |
| SB021 contig 2 | OKKMCADK_00081 | CDS | 1221 |  |  |  | Hypothetical protein |
| SB021 contig 2 | OKKMCADK_00082 | CDS | 801 |  |  |  | Hypothetical protein |
| SB021 contig 2 | OKKMCADK_00083 | CDS | 1167 | *patB* | 4.4.1.13 | COG1168 | Cystathionine beta-lyase PatB |
| SB021 contig 2 | OKKMCADK_00084 | CDS | 1446 | *mleN* |  | COG1757 | Malate-2H(+)/Na(+)-lactate antiporter |
| SB021 contig 2 | OKKMCADK_00085 | CDS | 1455 | *xylB* | 2.7.1.17 | COG1070 | Xylulose kinase |
| SB021 contig 2 | OKKMCADK_00086 | CDS | 1311 | *xylA* | 5.3.1.5 |  | Xylose isomerase |
| SB021 contig 2 | OKKMCADK_00087 | CDS | 1167 | *nagC* |  | COG1940 | N-acetylglucosamine repressor |
| SB021 contig 2 | OKKMCADK_00088 | CDS | 1056 |  |  |  | IS200/IS605 family transposase ISDge19 |
| SB021 contig 2 | OKKMCADK_00089 | CDS | 303 |  |  |  | Hypothetical protein |
| SB021 contig 2 | OKKMCADK_00090 | CDS | 279 |  |  |  | Hypothetical protein |
| SB021 contig 2 | OKKMCADK_00091 | CDS | 183 |  |  |  | Hypothetical protein |
| SB021 contig 2 | OKKMCADK_00092 | CDS | 420 |  |  |  | Hypothetical protein |
| SB021 contig 2 | OKKMCADK_00093 | CDS | 1515 |  |  |  | Hypothetical protein |
| SB021 contig 2 | OKKMCADK_00094 | CDS | 471 |  |  |  | Hypothetical protein |
| SB021 contig 2 | OKKMCADK_00095 | CDS | 345 |  |  |  | Hypothetical protein |
| SB021 contig 2 | OKKMCADK_00096 | CDS | 414 |  |  |  | Hypothetical protein |
| SB021 contig 2 | OKKMCADK_00097 | CDS | 1149 |  |  |  | Hypothetical protein |
| SB021 contig 2 | OKKMCADK_00098 | CDS | 894 |  |  |  | Hypothetical protein |
| SB021 contig 2 | OKKMCADK_00099 | CDS | 399 |  |  |  | Hypothetical protein |
| SB021 contig 2 | OKKMCADK_00100 | CDS | 1470 |  |  |  | IS200/IS605 family transposase ISCbt1 |
| SB021 contig 2 | OKKMCADK_00101 | CDS | 810 |  |  |  | Hypothetical protein |
| SB021 contig 2 | OKKMCADK_00102 | CDS | 2499 |  |  |  | Hypothetical protein |
| SB021 contig 2 | OKKMCADK_00103 | CDS | 657 |  |  |  | Hypothetical protein |
| SB021 contig 2 | OKKMCADK_00104 | CDS | 1479 |  |  |  | IS1182 family transposase ISFnu2 |
| SB021 contig 2 | OKKMCADK_00105 | CDS | 282 | *yafQ* | 3.1.-.- |  | mRNA interferase toxin YafQ |
| SB021 contig 2 | OKKMCADK_00106 | CDS | 267 |  |  |  | Hypothetical protein |
| SB021 contig 2 | OKKMCADK_00107 | CDS | 660 | *xerC* |  |  | Tyrosine recombinase XerC |
| SB021 contig 2 | OKKMCADK_00108 | CDS | 303 |  |  |  | Hypothetical protein |
| SB021 contig 2 | OKKMCADK_00109 | CDS | 675 |  |  |  | Hypothetical protein |
| SB021 contig 2 | OKKMCADK_00110 | CDS | 912 |  |  |  | Hypothetical protein |
| SB021 contig 2 | OKKMCADK_00111 | CDS | 1146 |  |  |  | Hypothetical protein |
| SB021 contig 2 | OKKMCADK_00112 | CDS | 522 |  |  |  | Hypothetical protein |
| SB021 contig 2 | OKKMCADK_00113 | CDS | 270 |  |  |  | Hypothetical protein |
| SB021 contig 2 | OKKMCADK_00114 | CDS | 261 |  |  |  | Hypothetical protein |
| SB021 contig 2 | OKKMCADK_00115 | CDS | 1026 |  |  |  | Hypothetical protein |
| SB021 contig 2 | OKKMCADK_00116 | CDS | 396 |  |  |  | Hypothetical protein |
| SB021 contig 2 | OKKMCADK_00117 | CDS | 1116 |  |  | COG0630 | Type IV secretion system protein VirB11 |
| SB021 contig 2 | OKKMCADK_00118 | CDS | 267 |  |  |  | Hypothetical protein |
| SB021 contig 2 | OKKMCADK_00119 | CDS | 204 |  |  |  | Hypothetical protein |
| SB021 contig 2 | OKKMCADK_00120 | CDS | 2445 | *virB4* |  | COG3451 | Type IV secretion system protein virB4 |
| SB021 contig 2 | OKKMCADK_00121 | CDS | 525 |  |  |  | Hypothetical protein |
| SB021 contig 2 | OKKMCADK_00122 | CDS | 846 |  |  |  | Hypothetical protein |
| SB021 contig 2 | OKKMCADK_00123 | CDS | 1041 |  |  |  | Hypothetical protein |
| SB021 contig 2 | OKKMCADK_00124 | CDS | 2562 |  |  |  | Hypothetical protein |
| SB021 contig 2 | OKKMCADK_00125 | CDS | 2328 | *topB* | 5.6.2.1 | COG0550 | DNA topoisomerase 3 |
| SB021 contig 2 | OKKMCADK_00126 | CDS | 546 |  |  |  | Hypothetical protein |
| SB021 contig 2 | OKKMCADK_00127 | CDS | 537 |  |  |  | Hypothetical protein |
| SB021 contig 2 | OKKMCADK_00128 | CDS | 420 |  |  |  | Hypothetical protein |
| SB021 contig 2 | OKKMCADK_00129 | CDS | 138 |  |  |  | Hypothetical protein |
| SB021 contig 2 | OKKMCADK_00130 | CDS | 1527 |  |  |  | Hypothetical protein |
| SB021 contig 2 | OKKMCADK_00131 | CDS | 312 |  |  |  | Hypothetical protein |
| SB021 contig 2 | OKKMCADK_00132 | CDS | 186 |  |  |  | Hypothetical protein |
| SB021 contig 2 | OKKMCADK_00133 | CDS | 366 |  |  |  | Hypothetical protein |
| SB021 contig 2 | OKKMCADK_00134 | CDS | 1161 |  |  |  | Hypothetical protein |
| SB021 contig 2 | OKKMCADK_00135 | CDS | 294 | *ihfA_2* |  |  | Integration host factor subunit alpha |
| SB021 contig 2 | OKKMCADK_00136 | CDS | 294 | *ihfA_3* |  |  | Integration host factor subunit alpha |
| SB021 contig 2 | OKKMCADK_00137 | CDS | 2145 | *fcl_2* | 1.1.1.271 |  | GDP-L-fucose synthase |
| SB021 contig 2 | OKKMCADK_00138 | CDS | 675 | *mshB* | 3.5.1.103 |  | 1D-myo-inositol 2-acetamido-2-deoxy-alpha-D-glucopyranoside deacetylase |
| SB021 contig 2 | OKKMCADK_00139 | CDS | 606 | *pglC* | 2.7.8.36 | COG2148 | Undecaprenyl phosphate N,N'-diacetylbacillosamine 1-phosphate transferase |
| SB021 contig 2 | OKKMCADK_00140 | CDS | 1242 | *lysA* | 4.1.1.20 | COG0019 | Diaminopimelate decarboxylase |
| SB021 contig 2 | OKKMCADK_00141 | CDS | 1047 |  |  |  | Hypothetical protein |
| SB021 contig 2 | OKKMCADK_00142 | CDS | 843 | *gnu_2* | 5.1.3.26 | COG0451 | N-acetyl-alpha-D-glucosaminyl-diphospho-ditrans,octacis-undecaprenol 4-epimerase |
| SB021 contig 2 | OKKMCADK_00143 | CDS | 1242 |  |  |  | Hypothetical protein |
| SB021 contig 2 | OKKMCADK_00144 | CDS | 1332 | *rkpK* | 1.1.1.22 | COG1004 | UDP-glucose 6-dehydrogenase |
| SB021 contig 2 | OKKMCADK_00145 | CDS | 1173 | *desV* | 2.6.1.106 |  | dTDP-3-amino-3,4,6-trideoxy-alpha-D-glucose transaminase |
| SB021 contig 2 | OKKMCADK_00146 | CDS | 612 | *wbpD* | 2.3.1.201 | COG0110 | UDP-2-acetamido-3-amino-2,3-dideoxy-D-glucuronate N-acetyltransferase |
| SB021 contig 2 | OKKMCADK_00147 | CDS | 462 | *purN* | 2.1.2.2 |  | Phosphoribosylglycinamide formyltransferase |
| SB021 contig 2 | OKKMCADK_00148 | CDS | 1098 | *iolG* | 1.1.1.18 | COG0673 | Myo-inositol 2-dehydrogenase |
| SB021 contig 2 | OKKMCADK_00149 | CDS | 1026 |  |  |  | Hypothetical protein |
| SB021 contig 2 | OKKMCADK_00150 | CDS | 1095 | *mnaA* | 5.1.3.14 | COG0381 | UDP-N-acetylglucosamine 2-epimerase |
| SB021 contig 2 | OKKMCADK_00151 | CDS | 1044 |  |  |  | Hypothetical protein |
| SB021 contig 2 | OKKMCADK_00152 | CDS | 408 |  |  |  | Hypothetical protein |
| SB021 contig 2 | OKKMCADK_00153 | CDS | 945 |  |  |  | Hypothetical protein |
| SB021 contig 2 | OKKMCADK_00154 | CDS | 1698 | *pgcA* | 5.4.2.2 |  | Phosphoglucomutase |
| SB021 contig 2 | OKKMCADK_00155 | CDS | 1071 | *algA* |  | COG0662 | Alginate biosynthesis protein AlgA |
| SB021 contig 2 | OKKMCADK_00156 | CDS | 1074 |  |  |  | Hypothetical protein |
| SB021 contig 2 | OKKMCADK_00157 | CDS | 1170 |  |  |  | Hypothetical protein |
| SB021 contig 2 | OKKMCADK_00158 | CDS | 1527 | *murJ* |  |  | Lipid II flippase MurJ |
| SB021 contig 2 | OKKMCADK_00159 | CDS | 1209 |  |  |  | IS256 family transposase ISCth4 |
| SB021 contig 2 | OKKMCADK_00160 | CDS | 981 | *rfaE* | 2.7.1.167 | COG2870 | D-beta-D-heptose 7-phosphate kinase |
| SB021 contig 3 | DPDHICOG_00001 | CDS | 747 | *yutF* | 3.1.3.- | COG0647 | Acid sugar phosphatase |
| SB021 contig 3 | DPDHICOG_00002 | CDS | 720 | *glgC_1* | 2.7.7.27 |  | Glucose-1-phosphate adenylyltransferase |
| SB021 contig 3 | DPDHICOG_00003 | CDS | 1038 |  |  |  | Hypothetical protein |
| SB021 contig 3 | DPDHICOG_00004 | CDS | 1461 | *murJ* |  |  | Lipid II flippase MurJ |
| SB021 contig 3 | DPDHICOG_00005 | CDS | 918 | *tarF* | 2.7.8.12 |  | Teichoic acid poly(glycerol phosphate) polymerase |
| SB021 contig 3 | DPDHICOG_00006 | CDS | 261 |  |  |  | Hypothetical protein |
| SB021 contig 3 | DPDHICOG_00007 | CDS | 852 | *rfbD* | 1.1.1.133 | COG1091 | dTDP-4-dehydrorhamnose reductase |
| SB021 contig 3 | DPDHICOG_00008 | CDS | 441 | *rfbC* | 5.1.3.13 | COG1898 | dTDP-4-dehydrorhamnose 3,5-epimerase |
| SB021 contig 3 | DPDHICOG_00009 | CDS | 870 | *rfbA* | 2.7.7.24 | COG1209 | Glucose-1-phosphate thymidylyltransferase 1 |
| SB021 contig 3 | DPDHICOG_00010 | CDS | 918 |  |  |  | Hypothetical protein |
| SB021 contig 3 | DPDHICOG_00011 | CDS | 678 | *hddC* | 2.7.7.71 |  | D-glycero-alpha-D-manno-heptose 1-phosphate guanylyltransferase |
| SB021 contig 3 | DPDHICOG_00012 | CDS | 666 | *gmhA* | 5.3.1.28 |  | Phosphoheptose isomerase |
| SB021 contig 3 | DPDHICOG_00013 | CDS | 1032 | *hddA* | 2.7.1.168 |  | D-glycero-alpha-D-manno-heptose 7-phosphate kinase |
| SB021 contig 3 | DPDHICOG_00014 | CDS | 1218 |  |  |  | Hypothetical protein |
| SB021 contig 3 | DPDHICOG_00015 | CDS | 1161 |  |  |  | Hypothetical protein |
| SB021 contig 3 | DPDHICOG_00016 | CDS | 288 | *kanE* | 2.4.1.301 |  | Alpha-D-kanosaminyltransferase |
| SB021 contig 3 | DPDHICOG_00017 | CDS | 462 | *wbnH* | 2.4.1.306 |  | O-antigen biosynthesis glycosyltransferase WbnH |
| SB021 contig 3 | DPDHICOG_00018 | CDS | 228 |  |  |  | Hypothetical protein |
| SB021 contig 3 | DPDHICOG_00019 | CDS | 1332 |  |  |  | Hypothetical protein |
| SB021 contig 3 | DPDHICOG_00020 | CDS | 327 |  |  |  | Hypothetical protein |
| SB021 contig 3 | DPDHICOG_00021 | CDS | 369 |  |  |  | Hypothetical protein |
| SB021 contig 3 | DPDHICOG_00022 | CDS | 231 |  |  |  | Hypothetical protein |
| SB021 contig 3 | DPDHICOG_00023 | CDS | 387 |  |  |  | Hypothetical protein |
| SB021 contig 3 | DPDHICOG_00024 | CDS | 696 | *dapB* | 1.17.1.8 | COG0289 | 4-hydroxy-tetrahydrodipicolinate reductase |
| SB021 contig 3 | DPDHICOG_00025 | CDS | 318 | *dapA_1* | 4.3.3.7 | COG0329 | 4-hydroxy-tetrahydrodipicolinate synthase |
| SB021 contig 3 | DPDHICOG_00026 | CDS | 468 |  |  |  | Hypothetical protein |
| SB021 contig 3 | DPDHICOG_00027 | CDS | 426 |  |  |  | Hypothetical protein |
| SB021 contig 3 | DPDHICOG_00028 | CDS | 345 | *queE_1* | 4.3.99.3 | COG0602 | 7-carboxy-7-deazaguanine synthase |
| SB021 contig 3 | DPDHICOG_00029 | CDS | 303 | *queE_2* | 4.3.99.3 |  | 7-carboxy-7-deazaguanine synthase |
| SB021 contig 3 | DPDHICOG_00030 | CDS | 585 | *folE* | 3.5.4.16 | COG0302 | GTP cyclohydrolase 1 |
| SB021 contig 3 | DPDHICOG_00031 | CDS | 711 | *queC* | 6.3.4.20 | COG0603 | 7-cyano-7-deazaguanine synthase |
| SB021 contig 3 | DPDHICOG_00032 | CDS | 483 | *queF* | 1.7.1.13 | COG0780 | NADPH-dependent 7-cyano-7-deazaguanine reductase |
| SB021 contig 3 | DPDHICOG_00033 | CDS | 960 | *fba* | 4.1.2.13 |  | Fructose-bisphosphate aldolase |
| SB021 contig 3 | DPDHICOG_00034 | CDS | 897 | *yihV* | 2.7.1.184 |  | Sulfofructose kinase |
| SB021 contig 3 | DPDHICOG_00035 | CDS | 1371 | *ulaA* |  | COG3037 | Ascorbate-specific PTS system EIIC component |
| SB021 contig 3 | DPDHICOG_00036 | CDS | 276 |  |  |  | Hypothetical protein |
| SB021 contig 3 | DPDHICOG_00037 | CDS | 438 | *ulaC* |  | COG1762 | Ascorbate-specific PTS system EIIA component |
| SB021 contig 3 | DPDHICOG_00038 | CDS | 1233 |  |  |  | Hypothetical protein |
| SB021 contig 3 | DPDHICOG_00039 | CDS | 207 |  |  |  | Hypothetical protein |
| SB021 contig 3 | DPDHICOG_00040 | CDS | 510 |  |  |  | Hypothetical protein |
| SB021 contig 3 | DPDHICOG_00041 | CDS | 1353 | *mleN_1* |  | COG1757 | Malate-2H(+)/Na(+)-lactate antiporter |
| SB021 contig 3 | DPDHICOG_00042 | CDS | 1008 | *asnA* | 6.3.1.1 | COG2502 | Aspartate--ammonia ligase |
| SB021 contig 3 | DPDHICOG_00043 | CDS | 342 |  |  |  | Hypothetical protein |
| SB021 contig 3 | DPDHICOG_00044 | CDS | 477 | *isiB* |  |  | Flavodoxin |
| SB021 contig 3 | DPDHICOG_00045 | CDS | 1557 | *sir* | 1.8.7.1 | COG0155 | Sulfite reductase [ferredoxin] |
| SB021 contig 3 | DPDHICOG_00046 | CDS | 1503 |  |  |  | Hypothetical protein |
| SB021 contig 3 | DPDHICOG_00047 | CDS | 453 |  |  |  | Hypothetical protein |
| SB021 contig 3 | DPDHICOG_00048 | CDS | 969 |  |  |  | Hypothetical protein |
| SB021 contig 3 | DPDHICOG_00049 | CDS | 1803 |  |  |  | Hypothetical protein |
| SB021 contig 3 | DPDHICOG_00050 | CDS | 1206 | *mlc* |  | COG1940 | Protein mlc |
| SB021 contig 3 | DPDHICOG_00051 | CDS | 339 |  |  |  | Hypothetical protein |
| SB021 contig 3 | DPDHICOG_00052 | CDS | 330 |  |  |  | Hypothetical protein |
| SB021 contig 3 | DPDHICOG_00053 | CDS | 1005 | *thiI* | 2.8.1.4 |  | tRNA sulfurtransferase |
| SB021 contig 3 | DPDHICOG_00054 | CDS | 492 | *sepF_1* |  |  | Cell division protein SepF |
| SB021 contig 3 | DPDHICOG_00055 | CDS | 681 |  |  | COG0325 | Pyridoxal phosphate homeostasis protein |
| SB021 contig 3 | DPDHICOG_00056 | CDS | 1083 | *hemW* |  | COG0635 | Heme chaperone HemW |
| SB021 contig 3 | DPDHICOG_00057 | CDS | 1734 | *pgcA* | 5.4.2.2 |  | Phosphoglucomutase |
| SB021 contig 3 | DPDHICOG_00058 | CDS | 1251 |  |  |  | IS200/IS605 family transposase ISAsp8 |
| SB021 contig 3 | DPDHICOG_00059 | CDS | 360 |  |  |  | Hypothetical protein |
| SB021 contig 3 | DPDHICOG_00060 | CDS | 579 | *shdB* | 2.5.1.129 |  | Putative UbiX-like flavin prenyltransferase |
| SB021 contig 3 | DPDHICOG_00061 | CDS | 1410 | *lpdC* | 4.1.1.59 | COG0043 | Gallate decarboxylase |
| SB021 contig 3 | DPDHICOG_00062 | CDS | 864 | *cynR_1* |  |  | HTH-type transcriptional regulator CynR |
| SB021 contig 3 | DPDHICOG_00063 | CDS | 783 | *zupT* |  | COG0428 | Zinc transporter ZupT |
| SB021 contig 3 | DPDHICOG_00064 | CDS | 537 | *rbr* |  | COG1592 | Rubrerythrin |
| SB021 contig 3 | DPDHICOG_00065 | CDS | 339 |  |  |  | Hypothetical protein |
| SB021 contig 3 | DPDHICOG_00066 | CDS | 615 | *kynB* | 3.5.1.9 | COG1878 | Kynurenine formamidase |
| SB021 contig 3 | DPDHICOG_00067 | CDS | 561 | *tdk* | 2.7.1.21 |  | Thymidine kinase |
| SB021 contig 3 | DPDHICOG_00068 | CDS | 204 | *cspLA_1* |  | COG1278 | Cold shock-like protein CspLA |
| SB021 contig 3 | DPDHICOG_00069 | CDS | 321 |  |  |  | Hypothetical protein |
| SB021 contig 3 | DPDHICOG_00070 | CDS | 369 |  |  |  | Hypothetical protein |
| SB021 contig 3 | DPDHICOG_00071 | CDS | 1110 |  |  |  | Hypothetical protein |
| SB021 contig 3 | DPDHICOG_00072 | CDS | 1443 | *ptsG* | 2.7.1.199 | COG1263 | PTS system glucose-specific EIICBA component |
| SB021 contig 3 | DPDHICOG_00073 | CDS | 993 | *degA_1* |  | COG1609 | HTH-type transcriptional regulator DegA |
| SB021 contig 3 | DPDHICOG_00074 | CDS | 1407 | *treP_1* |  | COG1263 | PTS system trehalose-specific EIIBC component |
| SB021 contig 3 | DPDHICOG_00075 | CDS | 1386 | *scrB_1* | 3.2.1.26 | COG1621 | Sucrose-6-phosphate hydrolase |
| SB021 contig 3 | DPDHICOG_00076 | CDS | 954 | *scrK_1* | 2.7.1.4 | COG0524 | Fructokinase |
| SB021 contig 3 | DPDHICOG_00077 | CDS | 1176 |  |  |  | Hypothetical protein |
| SB021 contig 3 | DPDHICOG_00078 | CDS | 2994 | *rep* | 3.6.4.12 |  | ATP-dependent DNA helicase Rep |
| SB021 contig 3 | DPDHICOG_00079 | CDS | 2055 |  |  |  | Hypothetical protein |
| SB021 contig 3 | DPDHICOG_00080 | CDS | 927 | *moaA* |  |  | GTP 3',8-cyclase |
| SB021 contig 3 | DPDHICOG_00081 | CDS | 684 | *yhhW* | 1.13.11.24 | COG1741 | Quercetin 2,3-dioxygenase |
| SB021 contig 3 | DPDHICOG_00082 | CDS | 474 | *ybaK* | 4.2.-.- | COG2606 | Cys-tRNA(Pro)/Cys-tRNA(Cys) deacylase YbaK |
| SB021 contig 3 | DPDHICOG_00083 | CDS | 708 | *comB* | 3.1.3.71 | COG2045 | Putative 2-phosphosulfolactate phosphatase |
| SB021 contig 3 | DPDHICOG_00084 | CDS | 354 | *yabJ* | 3.5.99.10 | COG0251 | 2-iminobutanoate/2-iminopropanoate deaminase |
| SB021 contig 3 | DPDHICOG_00085 | CDS | 306 | *sepF_2* |  |  | Cell division protein SepF |
| SB021 contig 3 | DPDHICOG_00086 | CDS | 945 | *nrnA* | 3.1.-.- | COG0618 | Bifunctional oligoribonuclease and PAP phosphatase NrnA |
| SB021 contig 3 | DPDHICOG_00087 | CDS | 1941 | *hprK* | 2.7.4.- |  | HPr kinase/phosphorylase |
| SB021 contig 3 | DPDHICOG_00088 | CDS | 192 |  |  |  | Hypothetical protein |
| SB021 contig 3 | DPDHICOG_00089 | CDS | 1230 | *folC* | 6.3.2.12 | COG0285 | Dihydrofolate synthase/folylpolyglutamate synthase |
| SB021 contig 3 | DPDHICOG_00090 | CDS | 693 | *mtnN* | 3.2.2.9 | COG0775 | 5'-methylthioadenosine/S-adenosylhomocysteine nucleosidase |
| SB021 contig 3 | DPDHICOG_00091 | CDS | 903 | *lpxL* | 2.3.1.241 | COG1560 | Lipid A biosynthesis lauroyltransferase |
| SB021 contig 3 | DPDHICOG_00092 | CDS | 747 | *yaaA* |  | COG3022 | Peroxide stress resistance protein YaaA |
| SB021 contig 3 | DPDHICOG_00093 | CDS | 453 | *bcp* | 1.11.1.15 | COG1225 | Putative peroxiredoxin bcp |
| SB021 contig 3 | DPDHICOG_00094 | CDS | 756 | *ydfG* | 1.1.1.381 | COG4221 | NADP-dependent 3-hydroxy acid dehydrogenase YdfG |
| SB021 contig 3 | DPDHICOG_00095 | CDS | 1203 |  |  |  | Hypothetical protein |
| SB021 contig 3 | DPDHICOG_00096 | CDS | 831 |  |  |  | Hypothetical protein |
| SB021 contig 3 | DPDHICOG_00097 | CDS | 279 |  |  |  | Hypothetical protein |
| SB021 contig 3 | DPDHICOG_00098 | CDS | 576 |  |  |  | Hypothetical protein |
| SB021 contig 3 | DPDHICOG_00099 | CDS | 234 |  |  |  | Hypothetical protein |
| SB021 contig 3 | DPDHICOG_00100 | CDS | 1377 | *tuf* |  |  | Elongation factor Tu |
| SB021 contig 3 | DPDHICOG_00101 | CDS | 1383 | *selA* | 2.9.1.1 | COG1921 | L-seryl-tRNA(Sec) selenium transferase |
| SB021 contig 3 | DPDHICOG_00102 | CDS | 912 | *selD* | 2.7.9.3 | COG0709 | Selenide, water dikinase |
| SB021 contig 3 | DPDHICOG_00103 | CDS | 273 | *acyP* | 3.6.1.7 | COG1254 | Acylphosphatase |
| SB021 contig 3 | DPDHICOG_00104 | CDS | 921 | *ppx2* | 3.6.1.11 | COG0248 | Exopolyphosphatase 2 |
| SB021 contig 3 | DPDHICOG_00105 | CDS | 486 | *linA* |  |  | Lincosamide resistance protein |
| SB021 contig 3 | DPDHICOG_00106 | CDS | 156 |  |  |  | Hypothetical protein |
| SB021 contig 3 | DPDHICOG_00107 | CDS | 339 |  |  |  | Hypothetical protein |
| SB021 contig 3 | DPDHICOG_00108 | CDS | 846 | *htpX* | 3.4.24.- | COG0501 | Protease HtpX |
| SB021 contig 3 | DPDHICOG_00109 | CDS | 801 |  |  |  | Hypothetical protein |
| SB021 contig 3 | DPDHICOG_00110 | CDS | 321 |  |  |  | Hypothetical protein |
| SB021 contig 3 | DPDHICOG_00111 | CDS | 855 |  |  |  | Hypothetical protein |
| SB021 contig 3 | DPDHICOG_00112 | CDS | 852 |  |  |  | Hypothetical protein |
| SB021 contig 3 | DPDHICOG_00113 | CDS | 855 |  |  |  | Hypothetical protein |
| SB021 contig 3 | DPDHICOG_00114 | CDS | 447 |  |  |  | Hypothetical protein |
| SB021 contig 3 | DPDHICOG_00115 | CDS | 357 |  |  |  | Hypothetical protein |
| SB021 contig 3 | DPDHICOG_00116 | CDS | 2703 |  |  |  | Hypothetical protein |
| SB021 contig 3 | DPDHICOG_00117 | CDS | 5937 |  |  |  | Hypothetical protein |
| SB021 contig 3 | DPDHICOG_00118 | CDS | 159 |  |  |  | Hypothetical protein |
| SB021 contig 3 | DPDHICOG_00119 | CDS | 747 | *ssuC* |  | COG0600 | Putative aliphatic sulfonates transport permease protein SsuC |
| SB021 contig 3 | DPDHICOG_00120 | CDS | 699 | *ssuB* | 3.6.3.- | COG1116 | Aliphatic sulfonates import ATP-binding protein SsuB |
| SB021 contig 3 | DPDHICOG_00121 | CDS | 948 | *ssuA* |  | COG0715 | Putative aliphatic sulfonates-binding protein |
| SB021 contig 3 | DPDHICOG_00122 | CDS | 645 |  |  |  | Hypothetical protein |
| SB021 contig 3 | DPDHICOG_00123 | CDS | 717 |  | 3.1.3.- | COG0560 | Putative phosphatase |
| SB021 contig 3 | DPDHICOG_00124 | CDS | 510 |  |  |  | Hypothetical protein |
| SB021 contig 3 | DPDHICOG_00125 | CDS | 648 |  |  |  | Hypothetical protein |
| SB021 contig 3 | DPDHICOG_00126 | CDS | 771 | *hdhA* | 1.1.1.159 |  | 7-alpha-hydroxysteroid dehydrogenase |
| SB021 contig 3 | DPDHICOG_00127 | CDS | 1062 | *alr1* | 5.1.1.1 |  | Alanine racemase 1 |
| SB021 contig 3 | DPDHICOG_00128 | CDS | 582 |  |  |  | Hypothetical protein |
| SB021 contig 3 | DPDHICOG_00129 | CDS | 408 |  |  |  | Hypothetical protein |
| SB021 contig 3 | DPDHICOG_00130 | CDS | 861 | *lgt* | 2.5.1.145 | COG0682 | Phosphatidylglycerol--prolipoprotein diacylglyceryl transferase |
| SB021 contig 3 | DPDHICOG_00131 | CDS | 1209 | *norV* |  | COG0426 | Anaerobic nitric oxide reductase flavorubredoxin |
| SB021 contig 3 | DPDHICOG_00132 | CDS | 255 |  |  |  | Hypothetical protein |
| SB021 contig 3 | DPDHICOG_00133 | CDS | 1449 | *panF* |  | COG4145 | Sodium/pantothenate symporter |
| SB021 contig 3 | DPDHICOG_00134 | CDS | 165 |  |  |  | Hypothetical protein |
| SB021 contig 3 | DPDHICOG_00135 | CDS | 720 | *lptB* | 3.6.3.- | COG1137 | Lipopolysaccharide export system ATP-binding protein LptB |
| SB021 contig 3 | DPDHICOG_00136 | CDS | 918 |  |  |  | Hypothetical protein |
| SB021 contig 3 | DPDHICOG_00137 | CDS | 1302 |  |  |  | Hypothetical protein |
| SB021 contig 3 | DPDHICOG_00138 | CDS | 723 |  |  |  | Hypothetical protein |
| SB021 contig 3 | DPDHICOG_00139 | CDS | 1722 |  |  | COG1132 | Putative ABC transporter ATP-binding protein |
| SB021 contig 3 | DPDHICOG_00140 | CDS | 888 | *btuF* |  | COG0614 | Vitamin B12-binding protein |
| SB021 contig 3 | DPDHICOG_00141 | CDS | 582 |  |  |  | Hypothetical protein |
| SB021 contig 3 | DPDHICOG_00142 | CDS | 1383 | *tldD* | 3.4.-.- | COG0312 | Metalloprotease TldD |
| SB021 contig 3 | DPDHICOG_00143 | CDS | 1479 |  |  |  | IS1182 family transposase ISFnu2 |
| SB021 contig 3 | DPDHICOG_00144 | CDS | 756 |  |  |  | Hypothetical protein |
| SB021 contig 3 | DPDHICOG_00145 | CDS | 534 |  |  |  | Hypothetical protein |
| SB021 contig 3 | DPDHICOG_00146 | CDS | 1362 |  |  |  | Hypothetical protein |
| SB021 contig 3 | DPDHICOG_00147 | CDS | 159 |  |  |  | Hypothetical protein |
| SB021 contig 3 | DPDHICOG_00148 | CDS | 570 |  |  |  | Hypothetical protein |
| SB021 contig 3 | DPDHICOG_00149 | CDS | 207 |  |  |  | Hypothetical protein |
| SB021 contig 3 | DPDHICOG_00150 | CDS | 132 |  |  |  | Hypothetical protein |
| SB021 contig 3 | DPDHICOG_00151 | CDS | 231 |  |  |  | Hypothetical protein |
| SB021 contig 3 | DPDHICOG_00152 | CDS | 357 | *ssb* |  | COG0629 | Single-stranded DNA-binding protein |
| SB021 contig 3 | DPDHICOG_00153 | CDS | 240 |  |  |  | Hypothetical protein |
| SB021 contig 3 | DPDHICOG_00154 | CDS | 900 |  |  |  | Hypothetical protein |
| SB021 contig 3 | DPDHICOG_00155 | CDS | 702 |  |  |  | Hypothetical protein |
| SB021 contig 3 | DPDHICOG_00156 | CDS | 195 |  |  |  | Hypothetical protein |
| SB021 contig 3 | DPDHICOG_00157 | CDS | 999 |  |  |  | Hypothetical protein |
| SB021 contig 3 | DPDHICOG_00158 | CDS | 138 |  |  |  | Hypothetical protein |
| SB021 contig 3 | DPDHICOG_00159 | CDS | 285 |  |  |  | Hypothetical protein |
| SB021 contig 3 | DPDHICOG_00160 | CDS | 810 |  |  |  | Hypothetical protein |
| SB021 contig 3 | DPDHICOG_00161 | CDS | 228 |  |  |  | Hypothetical protein |
| SB021 contig 3 | DPDHICOG_00162 | CDS | 333 |  |  |  | Hypothetical protein |
| SB021 contig 3 | DPDHICOG_00163 | CDS | 363 |  |  |  | Hypothetical protein |
| SB021 contig 3 | DPDHICOG_00164 | CDS | 624 |  |  |  | Hypothetical protein |
| SB021 contig 3 | DPDHICOG_00165 | CDS | 195 |  |  |  | Hypothetical protein |
| SB021 contig 3 | DPDHICOG_00166 | CDS | 684 | *lexA_1* | 3.4.21.88 |  | LexA repressor |
| SB021 contig 3 | DPDHICOG_00167 | CDS | 1056 |  |  |  | Hypothetical protein |
| SB021 contig 3 | DPDHICOG_00168 | CDS | 960 |  |  |  | Hypothetical protein |
| SB021 contig 3 | DPDHICOG_00169 | CDS | 180 |  |  |  | Hypothetical protein |
| SB021 contig 3 | DPDHICOG_00170 | CDS | 192 |  |  |  | Hypothetical protein |
| SB021 contig 3 | DPDHICOG_00171 | CDS | 174 |  |  |  | Hypothetical protein |
| SB021 contig 3 | DPDHICOG_00172 | tRNA | 71 |  |  |  | tRNA-Gln(ttg) |
| SB021 contig 3 | DPDHICOG_00173 | CDS | 1017 |  |  |  | Hypothetical protein |
| SB021 contig 3 | DPDHICOG_00174 | CDS | 495 |  |  |  | Hypothetical protein |
| SB021 contig 3 | DPDHICOG_00175 | CDS | 630 |  |  |  | Hypothetical protein |
| SB021 contig 3 | DPDHICOG_00176 | CDS | 324 |  |  |  | Hypothetical protein |
| SB021 contig 3 | DPDHICOG_00177 | CDS | 624 | *lexA_2* | 3.4.21.88 |  | LexA repressor |
| SB021 contig 3 | DPDHICOG_00178 | CDS | 579 |  |  |  | Hypothetical protein |
| SB021 contig 3 | DPDHICOG_00179 | CDS | 204 |  |  |  | Hypothetical protein |
| SB021 contig 3 | DPDHICOG_00180 | CDS | 609 |  |  |  | Hypothetical protein |
| SB021 contig 3 | DPDHICOG_00181 | CDS | 780 | *xerC_1* |  |  | Tyrosine recombinase XerC |
| SB021 contig 3 | DPDHICOG_00182 | CDS | 111 |  |  |  | Hypothetical protein |
| SB021 contig 3 | DPDHICOG_00183 | CDS | 411 |  |  |  | Hypothetical protein |
| SB021 contig 3 | DPDHICOG_00184 | CDS | 1860 |  |  |  | Hypothetical protein |
| SB021 contig 3 | DPDHICOG_00185 | CDS | 966 |  |  |  | Hypothetical protein |
| SB021 contig 3 | DPDHICOG_00186 | CDS | 243 |  |  |  | Hypothetical protein |
| SB021 contig 3 | DPDHICOG_00187 | CDS | 174 |  |  |  | Hypothetical protein |
| SB021 contig 3 | DPDHICOG_00188 | CDS | 657 |  |  |  | Hypothetical protein |
| SB021 contig 3 | DPDHICOG_00189 | CDS | 390 |  |  |  | Hypothetical protein |
| SB021 contig 3 | DPDHICOG_00190 | CDS | 225 |  |  |  | Hypothetical protein |
| SB021 contig 3 | DPDHICOG_00191 | CDS | 312 |  |  |  | Hypothetical protein |
| SB021 contig 3 | DPDHICOG_00192 | CDS | 102 |  |  |  | Hypothetical protein |
| SB021 contig 3 | DPDHICOG_00193 | CDS | 279 |  |  |  | Hypothetical protein |
| SB021 contig 3 | DPDHICOG_00194 | CDS | 273 |  |  |  | Hypothetical protein |
| SB021 contig 3 | DPDHICOG_00195 | CDS | 693 | *rpoS* |  |  | RNA polymerase sigma factor RpoS |
| SB021 contig 3 | DPDHICOG_00196 | CDS | 306 |  |  |  | Hypothetical protein |
| SB021 contig 3 | DPDHICOG_00197 | CDS | 252 |  |  |  | Hypothetical protein |
| SB021 contig 3 | DPDHICOG_00198 | CDS | 954 |  |  |  | Hypothetical protein |
| SB021 contig 3 | DPDHICOG_00199 | CDS | 984 |  |  |  | Hypothetical protein |
| SB021 contig 3 | DPDHICOG_00200 | CDS | 174 |  |  |  | Hypothetical protein |
| SB021 contig 3 | DPDHICOG_00201 | CDS | 882 |  |  |  | Hypothetical protein |
| SB021 contig 3 | DPDHICOG_00202 | CDS | 234 |  |  |  | Hypothetical protein |
| SB021 contig 3 | DPDHICOG_00203 | CDS | 456 |  |  |  | Hypothetical protein |
| SB021 contig 3 | DPDHICOG_00204 | CDS | 672 |  |  |  | Hypothetical protein |
| SB021 contig 3 | DPDHICOG_00205 | CDS | 570 |  |  |  | Hypothetical protein |
| SB021 contig 3 | DPDHICOG_00206 | CDS | 138 |  |  |  | Hypothetical protein |
| SB021 contig 3 | DPDHICOG_00207 | CDS | 1095 |  |  |  | Hypothetical protein |
| SB021 contig 3 | DPDHICOG_00208 | CDS | 747 |  |  |  | Hypothetical protein |
| SB021 contig 3 | DPDHICOG_00209 | CDS | 1791 |  |  |  | Hypothetical protein |
| SB021 contig 3 | DPDHICOG_00210 | CDS | 510 |  |  |  | Hypothetical protein |
| SB021 contig 3 | DPDHICOG_00211 | CDS | 501 |  |  |  | Hypothetical protein |
| SB021 contig 3 | DPDHICOG_00212 | CDS | 336 |  |  |  | Hypothetical protein |
| SB021 contig 3 | DPDHICOG_00213 | CDS | 516 |  |  |  | Hypothetical protein |
| SB021 contig 3 | DPDHICOG_00214 | CDS | 1050 |  |  |  | Hypothetical protein |
| SB021 contig 3 | DPDHICOG_00215 | CDS | 270 |  |  |  | Hypothetical protein |
| SB021 contig 3 | DPDHICOG_00216 | CDS | 393 |  |  |  | Hypothetical protein |
| SB021 contig 3 | DPDHICOG_00217 | CDS | 396 |  |  |  | Hypothetical protein |
| SB021 contig 3 | DPDHICOG_00218 | CDS | 1983 |  |  |  | Hypothetical protein |
| SB021 contig 3 | DPDHICOG_00219 | CDS | 543 |  |  |  | Hypothetical protein |
| SB021 contig 3 | DPDHICOG_00220 | CDS | 333 |  |  |  | Hypothetical protein |
| SB021 contig 3 | DPDHICOG_00221 | CDS | 408 |  |  |  | Hypothetical protein |
| SB021 contig 3 | DPDHICOG_00222 | CDS | 471 |  |  |  | Hypothetical protein |
| SB021 contig 3 | DPDHICOG_00223 | CDS | 588 |  |  |  | Hypothetical protein |
| SB021 contig 3 | DPDHICOG_00224 | CDS | 336 |  |  |  | Hypothetical protein |
| SB021 contig 3 | DPDHICOG_00225 | CDS | 1101 |  |  |  | Hypothetical protein |
| SB021 contig 3 | DPDHICOG_00226 | CDS | 621 |  |  |  | Hypothetical protein |
| SB021 contig 3 | DPDHICOG_00227 | CDS | 1581 |  |  |  | Hypothetical protein |
| SB021 contig 3 | DPDHICOG_00228 | CDS | 675 |  |  |  | Hypothetical protein |
| SB021 contig 3 | DPDHICOG_00229 | CDS | 312 |  |  |  | Hypothetical protein |
| SB021 contig 3 | DPDHICOG_00230 | CDS | 438 |  |  |  | Hypothetical protein |
| SB021 contig 3 | DPDHICOG_00231 | CDS | 468 |  |  |  | Hypothetical protein |
| SB021 contig 3 | DPDHICOG_00232 | CDS | 126 |  |  |  | Hypothetical protein |
| SB021 contig 3 | DPDHICOG_00233 | CDS | 204 |  |  |  | Hypothetical protein |
| SB021 contig 3 | DPDHICOG_00234 | CDS | 648 |  |  |  | Hypothetical protein |
| SB021 contig 3 | DPDHICOG_00235 | CDS | 174 |  |  |  | Hypothetical protein |
| SB021 contig 3 | DPDHICOG_00236 | CDS | 597 |  |  |  | Hypothetical protein |
| SB021 contig 3 | DPDHICOG_00237 | CDS | 414 |  |  |  | Hypothetical protein |
| SB021 contig 3 | DPDHICOG_00238 | CDS | 174 |  |  |  | Hypothetical protein |
| SB021 contig 3 | DPDHICOG_00239 | CDS | 942 |  |  |  | Hypothetical protein |
| SB021 contig 3 | DPDHICOG_00240 | CDS | 1323 | *dinF* |  | COG0534 | DNA damage-inducible protein F |
| SB021 contig 3 | DPDHICOG_00241 | CDS | 798 | *oppF* |  | COG4608 | Oligopeptide transport ATP-binding protein OppF |
| SB021 contig 3 | DPDHICOG_00242 | CDS | 837 | *cntD* | 7.2.2.- | COG0444 | Metal-staphylopine import system ATP-binding protein CntD |
| SB021 contig 3 | DPDHICOG_00243 | CDS | 825 | *cntC* |  | COG1173 | Metal-staphylopine import system permease protein CntC |
| SB021 contig 3 | DPDHICOG_00244 | CDS | 942 | *nikB* |  | COG0601 | Nickel transport system permease protein NikB |
| SB021 contig 3 | DPDHICOG_00245 | CDS | 1596 | *cntA* |  | COG0747 | Metal-staphylopine-binding protein CntA |
| SB021 contig 3 | DPDHICOG_00246 | CDS | 993 | *degA_2* |  | COG1609 | HTH-type transcriptional regulator DegA |
| SB021 contig 3 | DPDHICOG_00247 | CDS | 1407 | *treP_2* |  | COG1263 | PTS system trehalose-specific EIIBC component |
| SB021 contig 3 | DPDHICOG_00248 | CDS | 1386 | *scrB_2* | 3.2.1.26 | COG1621 | Sucrose-6-phosphate hydrolase |
| SB021 contig 3 | DPDHICOG_00249 | CDS | 954 | *scrK_2* | 2.7.1.4 | COG0524 | Fructokinase |
| SB021 contig 3 | DPDHICOG_00250 | CDS | 1545 |  |  |  | IS21 family transposase ISPpu7 |
| SB021 contig 3 | DPDHICOG_00251 | CDS | 768 |  |  |  | IS21 family transposase ISSso4 |
| SB021 contig 3 | DPDHICOG_00252 | CDS | 1218 |  |  |  | Hypothetical protein |
| SB021 contig 3 | DPDHICOG_00253 | CDS | 342 | *xerC_2* |  |  | Tyrosine recombinase XerC |
| SB021 contig 3 | DPDHICOG_00254 | tRNA | 77 |  |  |  | tRNA-Arg(tcg) |
| SB021 contig 3 | DPDHICOG_00255 | CDS | 2352 | *feoB* |  | COG0370 | Fe(2+) transporter FeoB |
| SB021 contig 3 | DPDHICOG_00256 | CDS | 147 |  |  |  | Hypothetical protein |
| SB021 contig 3 | DPDHICOG_00257 | CDS | 231 |  |  |  | Hypothetical protein |
| SB021 contig 3 | DPDHICOG_00258 | CDS | 312 | *glpE* | 2.8.1.1 |  | Thiosulfate sulfurtransferase GlpE |
| SB021 contig 3 | DPDHICOG_00259 | CDS | 2331 | *gshAB* |  | COG1181 | Glutathione biosynthesis bifunctional protein GshAB |
| SB021 contig 3 | DPDHICOG_00260 | CDS | 1320 | *mepA_1* |  |  | Multidrug export protein MepA |
| SB021 contig 3 | DPDHICOG_00261 | CDS | 1341 | *mepA_2* |  | COG0534 | Multidrug export protein MepA |
| SB021 contig 3 | DPDHICOG_00262 | CDS | 444 |  |  |  | Hypothetical protein |
| SB021 contig 3 | DPDHICOG_00263 | CDS | 1503 | *malQ* | 2.4.1.25 | COG1640 | 4-alpha-glucanotransferase |
| SB021 contig 3 | DPDHICOG_00264 | CDS | 204 |  |  |  | Hypothetical protein |
| SB021 contig 3 | DPDHICOG_00265 | CDS | 2247 | *nrdA* | 1.17.4.1 | COG0209 | Ribonucleoside-diphosphate reductase 1 subunit alpha |
| SB021 contig 3 | DPDHICOG_00266 | CDS | 1035 | *nrdB* | 1.17.4.1 | COG0208 | Ribonucleoside-diphosphate reductase subunit beta |
| SB021 contig 3 | DPDHICOG_00267 | CDS | 462 | *tabA* |  | COG2731 | Toxin-antitoxin biofilm protein TabA |
| SB021 contig 3 | DPDHICOG_00268 | CDS | 705 |  |  |  | Hypothetical protein |
| SB021 contig 3 | DPDHICOG_00269 | CDS | 876 | *cysL* |  | COG0583 | HTH-type transcriptional regulator CysL |
| SB021 contig 3 | DPDHICOG_00270 | CDS | 1008 |  |  |  | Hypothetical protein |
| SB021 contig 3 | DPDHICOG_00271 | CDS | 405 | *mscL* |  | COG1970 | Large-conductance mechanosensitive channel |
| SB021 contig 3 | DPDHICOG_00272 | CDS | 516 |  |  |  | Hypothetical protein |
| SB021 contig 3 | DPDHICOG_00273 | CDS | 408 |  |  |  | Hypothetical protein |
| SB021 contig 3 | DPDHICOG_00274 | CDS | 1398 |  |  |  | Hypothetical protein |
| SB021 contig 3 | DPDHICOG_00275 | CDS | 93 |  |  |  | Hypothetical protein |
| SB021 contig 3 | DPDHICOG_00276 | CDS | 2610 | *ppdK* | 2.7.9.1 | COG0574 | Pyruvate, phosphate dikinase |
| SB021 contig 3 | DPDHICOG_00277 | CDS | 819 | *yqfL* | 2.7.11.32 | COG1806 | Putative pyruvate, phosphate dikinase regulatory protein |
| SB021 contig 3 | DPDHICOG_00278 | CDS | 1194 | *nrdD_1* | 1.1.98.6 | COG1328 | Anaerobic ribonucleoside-triphosphate reductase |
| SB021 contig 3 | DPDHICOG_00279 | CDS | 519 | *nrdD_2* | 1.1.98.6 | COG1328 | Anaerobic ribonucleoside-triphosphate reductase |
| SB021 contig 3 | DPDHICOG_00280 | CDS | 348 | *nrdD_3* | 1.1.98.6 | COG1328 | Anaerobic ribonucleoside-triphosphate reductase |
| SB021 contig 3 | DPDHICOG_00281 | CDS | 336 | *nrdG* | 1.97.1.- | COG0602 | Anaerobic ribonucleoside-triphosphate reductase-activating protein |
| SB021 contig 3 | DPDHICOG_00282 | CDS | 1941 | *rsxB* | 7.-.-.- |  | Ion-translocating oxidoreductase complex subunit B |
| SB021 contig 3 | DPDHICOG_00283 | CDS | 1314 | *adeP* |  | COG2252 | Adenine permease AdeP |
| SB021 contig 3 | DPDHICOG_00284 | CDS | 1668 | *fhs* | 6.3.4.3 | COG2759 | Formate--tetrahydrofolate ligase |
| SB021 contig 3 | DPDHICOG_00285 | CDS | 204 | *cspLA_2* |  | COG1278 | Cold shock-like protein CspLA |
| SB021 contig 3 | DPDHICOG_00286 | CDS | 2499 | *menE* | 6.2.1.26 |  | 2-succinylbenzoate--CoA ligase |
| SB021 contig 3 | DPDHICOG_00287 | CDS | 147 |  |  |  | Hypothetical protein |
| SB021 contig 3 | DPDHICOG_00288 | CDS | 630 |  |  |  | Hypothetical protein |
| SB021 contig 3 | DPDHICOG_00289 | CDS | 729 |  |  |  | Hypothetical protein |
| SB021 contig 3 | DPDHICOG_00290 | CDS | 1032 | *rfaQ* | 2.-.-.- | COG0859 | Lipopolysaccharide core heptosyltransferase RfaQ |
| SB021 contig 3 | DPDHICOG_00291 | CDS | 711 |  |  |  | Hypothetical protein |
| SB021 contig 3 | DPDHICOG_00292 | CDS | 714 |  |  |  | Hypothetical protein |
| SB021 contig 3 | DPDHICOG_00293 | CDS | 423 | *gabR* |  | COG1167 | HTH-type transcriptional regulatory protein GabR |
| SB021 contig 3 | DPDHICOG_00294 | CDS | 255 | *lysN* | 2.6.1.39 | COG1167 | 2-aminoadipate transaminase |
| SB021 contig 3 | DPDHICOG_00295 | CDS | 762 |  |  |  | Hypothetical protein |
| SB021 contig 3 | DPDHICOG_00296 | CDS | 270 | *groS* |  | COG0234 | 10 kDa chaperonin |
| SB021 contig 3 | DPDHICOG_00297 | CDS | 1611 | *groL* |  |  | 60 kDa chaperonin |
| SB021 contig 3 | DPDHICOG_00298 | CDS | 600 |  |  |  | Hypothetical protein |
| SB021 contig 3 | DPDHICOG_00299 | CDS | 783 | *ybiV* | 3.1.3.23 | COG0561 | Sugar phosphatase YbiV |
| SB021 contig 3 | DPDHICOG_00300 | CDS | 909 | *nadA* | 2.5.1.72 | COG0379 | Quinolinate synthase A |
| SB021 contig 3 | DPDHICOG_00301 | CDS | 1311 | *nadB* | 1.4.3.16 | COG0029 | L-aspartate oxidase |
| SB021 contig 3 | DPDHICOG_00302 | CDS | 501 | *nadC_1* | 2.4.2.19 | COG0157 | Putative nicotinate-nucleotide pyrophosphorylase [carboxylating] |
| SB021 contig 3 | DPDHICOG_00303 | CDS | 303 | *nadC_2* | 2.4.2.19 | COG0157 | Nicotinate-nucleotide pyrophosphorylase [carboxylating] |
| SB021 contig 3 | DPDHICOG_00304 | CDS | 507 | *niaR* |  | COG1827 | Putative transcription repressor NiaR |
| SB021 contig 3 | DPDHICOG_00305 | CDS | 735 | *artJ* |  | COG0834 | ABC transporter arginine-binding protein 1 |
| SB021 contig 3 | DPDHICOG_00306 | CDS | 729 | *artM* |  | COG1126 | Arginine transport ATP-binding protein ArtM |
| SB021 contig 3 | DPDHICOG_00307 | CDS | 711 | *artQ* |  | COG0765 | Arginine transport system permease protein ArtQ |
| SB021 contig 3 | DPDHICOG_00308 | CDS | 780 |  | 3.1.3.- |  | Putative phosphatase |
| SB021 contig 3 | DPDHICOG_00309 | CDS | 717 |  |  |  | Hypothetical protein |
| SB021 contig 3 | DPDHICOG_00310 | CDS | 570 | *coaBC_1* |  | COG0452 | Coenzyme A biosynthesis bifunctional protein CoaBC |
| SB021 contig 3 | DPDHICOG_00311 | CDS | 552 | *coaBC_2* |  | COG0452 | Coenzyme A biosynthesis bifunctional protein CoaBC |
| SB021 contig 3 | DPDHICOG_00312 | CDS | 762 | *gmuE* | 2.7.1.4 | COG1940 | Putative fructokinase |
| SB021 contig 3 | DPDHICOG_00313 | CDS | 969 | *manA* | 5.3.1.8 | COG1482 | Mannose-6-phosphate isomerase ManA |
| SB021 contig 3 | DPDHICOG_00314 | CDS | 675 |  |  |  | Hypothetical protein |
| SB021 contig 3 | DPDHICOG_00315 | CDS | 561 |  |  |  | Hypothetical protein |
| SB021 contig 3 | DPDHICOG_00316 | CDS | 528 |  |  |  | Hypothetical protein |
| SB021 contig 3 | DPDHICOG_00317 | CDS | 240 |  |  |  | Hypothetical protein |
| SB021 contig 3 | DPDHICOG_00318 | CDS | 279 | *hup* |  | COG0776 | DNA-binding protein HU |
| SB021 contig 3 | DPDHICOG_00319 | CDS | 219 |  |  |  | Hypothetical protein |
| SB021 contig 3 | DPDHICOG_00320 | CDS | 186 |  |  |  | Hypothetical protein |
| SB021 contig 3 | DPDHICOG_00321 | CDS | 471 |  |  |  | Hypothetical protein |
| SB021 contig 3 | DPDHICOG_00322 | CDS | 414 |  |  |  | Hypothetical protein |
| SB021 contig 3 | DPDHICOG_00323 | CDS | 1446 |  |  |  | Hypothetical protein |
| SB021 contig 3 | DPDHICOG_00324 | CDS | 696 |  |  |  | Hypothetical protein |
| SB021 contig 3 | DPDHICOG_00325 | CDS | 519 |  |  |  | Hypothetical protein |
| SB021 contig 3 | DPDHICOG_00326 | CDS | 549 | *bcrC* | 3.6.1.27 | COG0671 | Undecaprenyl-disphosphatase BcrC |
| SB021 contig 3 | DPDHICOG_00327 | CDS | 651 | *murI* | 5.1.1.3 |  | Glutamate racemase |
| SB021 contig 3 | DPDHICOG_00328 | CDS | 1143 |  |  |  | Hypothetical protein |
| SB021 contig 3 | DPDHICOG_00329 | CDS | 1119 | *nagC_1* |  | COG1940 | N-acetylglucosamine repressor |
| SB021 contig 3 | DPDHICOG_00330 | CDS | 1464 | *opuE* |  | COG0591 | Osmoregulated proline transporter OpuE |
| SB021 contig 3 | DPDHICOG_00331 | CDS | 150 |  |  |  | Hypothetical protein |
| SB021 contig 3 | DPDHICOG_00332 | CDS | 1467 |  | 3.1.6.- | COG3119 | N-acetylglucosamine-6-O-sulfatase |
| SB021 contig 3 | DPDHICOG_00333 | CDS | 714 | *ylpA* |  |  | Lipoprotein YlpA |
| SB021 contig 3 | DPDHICOG_00334 | CDS | 225 |  |  |  | Hypothetical protein |
| SB021 contig 3 | DPDHICOG_00335 | CDS | 732 |  |  |  | Hypothetical protein |
| SB021 contig 3 | DPDHICOG_00336 | CDS | 552 |  |  |  | Hypothetical protein |
| SB021 contig 3 | DPDHICOG_00337 | CDS | 2571 |  |  |  | Hypothetical protein |
| SB021 contig 3 | DPDHICOG_00338 | CDS | 489 |  |  |  | Hypothetical protein |
| SB021 contig 3 | DPDHICOG_00339 | CDS | 480 |  |  |  | Hypothetical protein |
| SB021 contig 3 | DPDHICOG_00340 | CDS | 5136 |  |  |  | Hypothetical protein |
| SB021 contig 3 | DPDHICOG_00341 | CDS | 2838 |  |  |  | Hypothetical protein |
| SB021 contig 3 | DPDHICOG_00342 | CDS | 1317 |  |  |  | Hypothetical protein |
| SB021 contig 3 | DPDHICOG_00343 | CDS | 798 |  |  |  | Hypothetical protein |
| SB021 contig 3 | DPDHICOG_00344 | CDS | 2070 | *apu* |  | COG0366 | Amylopullulanase |
| SB021 contig 3 | DPDHICOG_00345 | CDS | 2433 | *glgP* | 2.4.1.1 | COG0058 | Glycogen phosphorylase |
| SB021 contig 3 | DPDHICOG_00346 | CDS | 1431 | *glgA* | 2.4.1.21 | COG0297 | Glycogen synthase |
| SB021 contig 3 | DPDHICOG_00347 | CDS | 1113 | *glgD* |  | COG0448 | Glycogen biosynthesis protein GlgD |
| SB021 contig 3 | DPDHICOG_00348 | CDS | 1140 | *glgC_2* | 2.7.7.27 | COG0448 | Glucose-1-phosphate adenylyltransferase |
| SB021 contig 3 | DPDHICOG_00349 | CDS | 1962 | *glgB* | 2.4.1.18 |  | 1,4-alpha-glucan branching enzyme GlgB |
| SB021 contig 3 | DPDHICOG_00350 | CDS | 456 |  |  |  | Hypothetical protein |
| SB021 contig 3 | DPDHICOG_00351 | CDS | 882 | *rluD* | 5.4.99.23 | COG0564 | Ribosomal large subunit pseudouridine synthase D |
| SB021 contig 3 | DPDHICOG_00352 | CDS | 921 | *glsA* | 3.5.1.2 | COG2066 | Glutaminase |
| SB021 contig 3 | DPDHICOG_00353 | CDS | 1164 | *nagA* | 3.5.1.25 | COG1820 | N-acetylglucosamine-6-phosphate deacetylase |
| SB021 contig 3 | DPDHICOG_00354 | CDS | 1338 | *mepA_3* |  | COG0534 | Multidrug export protein MepA |
| SB021 contig 3 | DPDHICOG_00355 | CDS | 303 | *ihfA_1* |  | COG0776 | Integration host factor subunit alpha |
| SB021 contig 3 | DPDHICOG_00356 | CDS | 285 |  |  |  | Hypothetical protein |
| SB021 contig 3 | DPDHICOG_00357 | CDS | 1383 | *mepA_4* |  | COG0534 | Multidrug export protein MepA |
| SB021 contig 3 | DPDHICOG_00358 | tRNA | 88 |  |  |  | tRNA-Leu(taa) |
| SB021 contig 3 | DPDHICOG_00359 | CDS | 1392 | *pepP* | 3.4.11.9 | COG0006 | Xaa-Pro aminopeptidase |
| SB021 contig 3 | DPDHICOG_00360 | CDS | 1140 |  | 1.8.98.- | COG0641 | Anaerobic sulfatase-maturating enzyme |
| SB021 contig 3 | DPDHICOG_00361 | CDS | 1107 | *ald* | 1.4.1.1 |  | Alanine dehydrogenase |
| SB021 contig 3 | DPDHICOG_00362 | CDS | 1320 | *mleN_2* |  | COG1757 | Malate-2H(+)/Na(+)-lactate antiporter |
| SB021 contig 3 | DPDHICOG_00363 | CDS | 366 |  |  |  | Hypothetical protein |
| SB021 contig 3 | DPDHICOG_00364 | CDS | 2148 |  |  |  | Hypothetical protein |
| SB021 contig 3 | DPDHICOG_00365 | CDS | 258 |  |  |  | Hypothetical protein |
| SB021 contig 3 | DPDHICOG_00366 | CDS | 747 |  |  |  | Hypothetical protein |
| SB021 contig 3 | DPDHICOG_00367 | CDS | 1179 |  |  |  | Hypothetical protein |
| SB021 contig 3 | DPDHICOG_00368 | CDS | 678 | *lldR* |  | COG2186 | Putative L-lactate dehydrogenase operon regulatory protein |
| SB021 contig 3 | DPDHICOG_00369 | CDS | 1542 | *glcA* |  | COG1620 | Glycolate permease GlcA |
| SB021 contig 3 | DPDHICOG_00370 | CDS | 1428 |  | 1.-.-.- | COG0277 | Putative FAD-linked oxidoreductase |
| SB021 contig 3 | DPDHICOG_00371 | CDS | 1137 |  | 1.3.8.1 | COG1960 | Acyl-CoA dehydrogenase, short-chain specific |
| SB021 contig 3 | DPDHICOG_00372 | CDS | 777 | *card* | 1.3.1.108 | COG2086 | Caffeyl-CoA reductase-Etf complex subunit CarD |
| SB021 contig 3 | DPDHICOG_00373 | CDS | 987 | *care* | 1.3.1.108 | COG2025 | Caffeyl-CoA reductase-Etf complex subunit CarE |
| SB021 contig 3 | DPDHICOG_00374 | CDS | 1440 | *bglA* | 3.2.1.86 | COG2723 | 6-phospho-beta-glucosidase BglA |
| SB021 contig 3 | DPDHICOG_00375 | CDS | 1386 | *bglF* |  | COG1263 | PTS system beta-glucoside-specific EIIBCA component |
| SB021 contig 3 | DPDHICOG_00376 | CDS | 879 | *murR_1* |  |  | HTH-type transcriptional regulator MurR |
| SB021 contig 3 | DPDHICOG_00377 | CDS | 1146 | *aspB* | 2.6.1.14 | COG0436 | Asparagine--oxo-acid transaminase |
| SB021 contig 3 | DPDHICOG_00378 | CDS | 1053 |  |  |  | Hypothetical protein |
| SB021 contig 3 | DPDHICOG_00379 | CDS | 723 | *hisJ* |  |  | Putative histidine-binding protein |
| SB021 contig 3 | DPDHICOG_00380 | CDS | 993 | *iolG* | 1.1.1.369 |  | Inositol 2-dehydrogenase/D-chiro-inositol 3-dehydrogenase |
| SB021 contig 3 | DPDHICOG_00381 | CDS | 660 | *rpiA* | 5.3.1.6 |  | Ribose-5-phosphate isomerase A |
| SB021 contig 3 | DPDHICOG_00382 | CDS | 846 | *murR_2* |  |  | HTH-type transcriptional regulator MurR |
| SB021 contig 3 | DPDHICOG_00383 | CDS | 1398 | *purF* | 2.4.2.14 | COG0034 | Amidophosphoribosyltransferase |
| SB021 contig 3 | DPDHICOG_00384 | CDS | 708 | *purC* | 6.3.2.6 | COG0152 | Phosphoribosylaminoimidazole-succinocarboxamide synthase |
| SB021 contig 3 | DPDHICOG_00385 | CDS | 132 |  |  |  | Hypothetical protein |
| SB021 contig 3 | DPDHICOG_00386 | CDS | 1611 | *pyrG* | 6.3.4.2 |  | CTP synthase |
| SB021 contig 3 | DPDHICOG_00387 | CDS | 681 | *glnM* |  | COG0765 | Putative glutamine ABC transporter permease protein GlnM |
| SB021 contig 3 | DPDHICOG_00388 | CDS | 732 | *glnQ* |  |  | Glutamine transport ATP-binding protein GlnQ |
| SB021 contig 3 | DPDHICOG_00389 | CDS | 774 | *artP_1* |  | COG0834 | Arginine-binding extracellular protein ArtP |
| SB021 contig 3 | DPDHICOG_00390 | CDS | 1428 | *tcyP* |  | COG1823 | L-cystine uptake protein TcyP |
| SB021 contig 3 | DPDHICOG_00391 | CDS | 1344 | *npr* | 1.11.1.1 | COG0446 | NADH peroxidase |
| SB021 contig 3 | DPDHICOG_00392 | CDS | 2196 | *btuB* |  |  | Vitamin B12 transporter BtuB |
| SB021 contig 3 | DPDHICOG_00393 | CDS | 384 |  |  |  | Hypothetical protein |
| SB021 contig 3 | DPDHICOG_00394 | CDS | 423 |  |  |  | Hypothetical protein |
| SB021 contig 3 | DPDHICOG_00395 | CDS | 903 | *cynR_2* |  |  | HTH-type transcriptional regulator CynR |
| SB021 contig 3 | DPDHICOG_00396 | CDS | 1038 |  |  |  | Hypothetical protein |
| SB021 contig 3 | DPDHICOG_00397 | CDS | 456 |  |  |  | Hypothetical protein |
| SB021 contig 3 | DPDHICOG_00398 | CDS | 1467 |  |  |  | Hypothetical protein |
| SB021 contig 3 | DPDHICOG_00399 | CDS | 1515 | *garD* | 4.2.1.42 | COG2721 | Galactarate dehydratase (L-threo-forming) |
| SB021 contig 3 | DPDHICOG_00400 | CDS | 969 |  |  |  | Hypothetical protein |
| SB021 contig 3 | DPDHICOG_00401 | CDS | 1326 | *gudD_1* | 4.2.1.40 | COG4948 | Glucarate dehydratase |
| SB021 contig 3 | DPDHICOG_00402 | CDS | 1356 | *gudD_2* | 4.2.1.40 |  | Glucarate dehydratase |
| SB021 contig 3 | DPDHICOG_00403 | CDS | 1305 | *citN* |  | COG2851 | Citrate transporter |
| SB021 contig 3 | DPDHICOG_00404 | CDS | 1110 | *cdaR* |  | COG3835 | Carbohydrate diacid regulator |
| SB021 contig 3 | DPDHICOG_00405 | CDS | 891 | *garR* | 1.1.1.60 | COG2084 | 2-hydroxy-3-oxopropionate reductase |
| SB021 contig 3 | DPDHICOG_00406 | CDS | 900 | *dapA_2* | 4.3.3.7 | COG0329 | 4-hydroxy-tetrahydrodipicolinate synthase |
| SB021 contig 3 | DPDHICOG_00407 | CDS | 1146 | *garK* | 2.7.1.165 | COG1929 | Glycerate 2-kinase |
| SB021 contig 3 | DPDHICOG_00408 | CDS | 966 | *hprA* | 1.1.1.29 | COG1052 | Glycerate dehydrogenase |
| SB021 contig 3 | DPDHICOG_00409 | CDS | 855 | *iolS* | 1.1.1.- | COG0667 | Aldo-keto reductase IolS |
| SB021 contig 3 | DPDHICOG_00410 | CDS | 633 |  |  |  | Hypothetical protein |
| SB021 contig 3 | DPDHICOG_00411 | CDS | 210 | *copZ* |  |  | Copper chaperone CopZ |
| SB021 contig 3 | DPDHICOG_00412 | CDS | 1209 |  |  |  | Hypothetical protein |
| SB021 contig 3 | DPDHICOG_00413 | CDS | 1254 | *gltP* |  | COG1301 | Proton/glutamate-aspartate symporter |
| SB021 contig 3 | DPDHICOG_00414 | CDS | 1314 |  |  |  | Hypothetical protein |
| SB021 contig 3 | DPDHICOG_00415 | CDS | 1317 |  |  |  | Hypothetical protein |
| SB021 contig 3 | DPDHICOG_00416 | CDS | 1536 | *malX* |  | COG1263 | PTS system maltose-specific EIICB component |
| SB021 contig 3 | DPDHICOG_00417 | CDS | 1182 | *malY* |  | COG1168 | Protein MalY |
| SB021 contig 3 | DPDHICOG_00418 | CDS | 1482 | *sasA* | 2.7.-.- |  | Adaptive-response sensory-kinase SasA |
| SB021 contig 3 | DPDHICOG_00419 | CDS | 666 | *rssB* |  |  | Regulator of RpoS |
| SB021 contig 3 | DPDHICOG_00420 | CDS | 435 | *kdpD_1* | 2.7.13.3 | COG2205 | Sensor protein KdpD |
| SB021 contig 3 | DPDHICOG_00421 | CDS | 639 | *kdpD_2* | 2.7.13.3 | COG2205 | Sensor protein KdpD |
| SB021 contig 3 | DPDHICOG_00422 | CDS | 1632 | *kdpA* |  | COG2060 | Potassium-transporting ATPase potassium-binding subunit |
| SB021 contig 3 | DPDHICOG_00423 | CDS | 2058 | *kdpB* | 7.2.2.6 | COG2216 | Potassium-transporting ATPase ATP-binding subunit |
| SB021 contig 3 | DPDHICOG_00424 | CDS | 573 | *kdpC* |  | COG2156 | Potassium-transporting ATPase KdpC subunit |
| SB021 contig 3 | DPDHICOG_00425 | CDS | 387 | *rlpA* | 4.2.2.- |  | Endolytic peptidoglycan transglycosylase RlpA |
| SB021 contig 3 | DPDHICOG_00426 | CDS | 1692 |  |  |  | Hypothetical protein |
| SB021 contig 3 | DPDHICOG_00427 | CDS | 1056 | *dinB* | 2.7.7.7 | COG0389 | DNA polymerase IV |
| SB021 contig 3 | DPDHICOG_00428 | CDS | 1467 | *nagE* |  | COG1263 | PTS system N-acetylglucosamine-specific EIICBA component |
| SB021 contig 3 | DPDHICOG_00429 | CDS | 264 |  |  |  | Hypothetical protein |
| SB021 contig 3 | DPDHICOG_00430 | CDS | 387 |  |  |  | Hypothetical protein |
| SB021 contig 3 | DPDHICOG_00431 | CDS | 1917 | *metG* | 6.1.1.10 |  | Methionine—tRNA ligase |
| SB021 contig 3 | DPDHICOG_00432 | CDS | 753 |  |  |  | Hypothetical protein |
| SB021 contig 3 | DPDHICOG_00433 | CDS | 879 | *gtaB* | 2.7.7.9 |  | UTP--glucose-1-phosphate uridylyltransferase |
| SB021 contig 3 | DPDHICOG_00434 | CDS | 783 | *yfcA* |  | COG0730 | Putative membrane transporter protein YfcA |
| SB021 contig 3 | DPDHICOG_00435 | CDS | 936 | *nifH* | 1.18.6.1 |  | Nitrogenase iron protein |
| SB021 contig 3 | DPDHICOG_00436 | CDS | 939 |  |  |  | Hypothetical protein |
| SB021 contig 3 | DPDHICOG_00437 | CDS | 831 |  |  |  | Hypothetical protein |
| SB021 contig 3 | DPDHICOG_00438 | CDS | 669 |  |  |  | Hypothetical protein |
| SB021 contig 3 | DPDHICOG_00439 | CDS | 636 |  |  |  | Hypothetical protein |
| SB021 contig 3 | DPDHICOG_00440 | CDS | 903 |  |  |  | Hypothetical protein |
| SB021 contig 3 | DPDHICOG_00441 | CDS | 744 |  |  |  | Hypothetical protein |
| SB021 contig 3 | DPDHICOG_00442 | CDS | 636 |  |  |  | Hypothetical protein |
| SB021 contig 3 | DPDHICOG_00443 | CDS | 1410 | *cof* | 3.6.1.- |  | HMP-PP phosphatase |
| SB021 contig 3 | DPDHICOG_00444 | CDS | 753 | *exoA* | 3.1.11.2 | COG0708 | Exodeoxyribonuclease |
| SB021 contig 3 | DPDHICOG_00445 | CDS | 930 | *ppx1* | 3.6.1.11 | COG0248 | Exopolyphosphatase 1 |
| SB021 contig 3 | DPDHICOG_00446 | CDS | 2055 | *ppk* | 2.7.4.1 |  | Polyphosphate kinase |
| SB021 contig 3 | DPDHICOG_00447 | CDS | 1902 |  |  |  | Hypothetical protein |
| SB021 contig 3 | DPDHICOG_00448 | CDS | 951 |  |  |  | Hypothetical protein |
| SB021 contig 3 | DPDHICOG_00449 | CDS | 1545 |  |  |  | IS21 family transposase ISPpu7 |
| SB021 contig 3 | DPDHICOG_00450 | CDS | 768 |  |  |  | IS21 family transposase ISSso4 |
| SB021 contig 3 | DPDHICOG_00451 | CDS | 150 |  |  |  | Hypothetical protein |
| SB021 contig 3 | DPDHICOG_00452 | CDS | 312 |  |  |  | Hypothetical protein |
| SB021 contig 3 | DPDHICOG_00453 | CDS | 213 |  |  |  | Hypothetical protein |
| SB021 contig 3 | DPDHICOG_00454 | CDS | 144 |  |  |  | Hypothetical protein |
| SB021 contig 3 | DPDHICOG_00455 | CDS | 213 |  |  |  | Hypothetical protein |
| SB021 contig 3 | DPDHICOG_00456 | CDS | 273 |  |  |  | Hypothetical protein |
| SB021 contig 3 | DPDHICOG_00457 | CDS | 129 |  |  |  | Hypothetical protein |
| SB021 contig 3 | DPDHICOG_00458 | CDS | 213 | *xis* |  |  | ICEBs1 excisionase |
| SB021 contig 3 | DPDHICOG_00459 | CDS | 189 |  |  |  | Hypothetical protein |
| SB021 contig 3 | DPDHICOG_00460 | CDS | 855 |  |  |  | Hypothetical protein |
| SB021 contig 3 | DPDHICOG_00461 | CDS | 1581 |  |  |  | Hypothetical protein |
| SB021 contig 3 | DPDHICOG_00462 | CDS | 687 | *lexA_3* | 3.4.21.88 |  | LexA repressor |
| SB021 contig 3 | DPDHICOG_00463 | CDS | 984 |  |  |  | Hypothetical protein |
| SB021 contig 3 | DPDHICOG_00464 | CDS | 1146 | *xerC_3* |  |  | Tyrosine recombinase XerC |
| SB021 contig 3 | DPDHICOG_00465 | CDS | 1554 | *ybiT* |  | COG0488 | Putative ABC transporter ATP-binding protein YbiT |
| SB021 contig 3 | DPDHICOG_00466 | CDS | 1407 | *alsT_1* |  | COG1115 | Amino-acid carrier protein AlsT |
| SB021 contig 3 | DPDHICOG_00467 | CDS | 426 |  |  |  | Hypothetical protein |
| SB021 contig 3 | DPDHICOG_00468 | CDS | 591 |  |  |  | Putative acetyltransferase |
| SB021 contig 3 | DPDHICOG_00469 | CDS | 873 | *yciV* | 3.1.13.- | COG0613 | 5'-3' exoribonuclease |
| SB021 contig 3 | DPDHICOG_00470 | CDS | 441 |  |  |  | Hypothetical protein |
| SB021 contig 3 | DPDHICOG_00471 | CDS | 1443 | *mepA_5* |  | COG0534 | Multidrug export protein MepA |
| SB021 contig 3 | DPDHICOG_00472 | CDS | 279 |  |  |  | Hypothetical protein |
| SB021 contig 3 | DPDHICOG_00473 | CDS | 795 | *yidA* | 3.1.3.23 | COG0561 | Sugar phosphatase YidA |
| SB021 contig 3 | DPDHICOG_00474 | CDS | 735 | *yfiC* | 2.1.1.223 |  | tRNA1(Val) (adenine(37)-N6)-methyltransferase |
| SB021 contig 3 | DPDHICOG_00475 | CDS | 315 |  |  |  | Hypothetical protein |
| SB021 contig 3 | DPDHICOG_00476 | CDS | 2424 | *xly* | 4.2.2.12 |  | Xanthan lyase |
| SB021 contig 3 | DPDHICOG_00477 | CDS | 2196 |  |  |  | Hypothetical protein |
| SB021 contig 3 | DPDHICOG_00478 | CDS | 2937 | *chonabc* | 4.2.2.21 |  | Chondroitin sulfate ABC exolyase |
| SB021 contig 3 | DPDHICOG_00479 | CDS | 1023 |  |  | COG1840 | Putative protein |
| SB021 contig 3 | DPDHICOG_00480 | CDS | 1134 | *potA* | 7.6.2.11 |  | Spermidine/putrescine import ATP-binding protein PotA |
| SB021 contig 3 | DPDHICOG_00481 | CDS | 519 |  |  |  | Hypothetical protein |
| SB021 contig 3 | DPDHICOG_00482 | CDS | 249 |  |  |  | Hypothetical protein |
| SB021 contig 3 | DPDHICOG_00483 | CDS | 819 | *phnV* |  | COG1177 | Putative 2-aminoethylphosphonate transport system permease protein PhnV |
| SB021 contig 3 | DPDHICOG_00484 | CDS | 1704 |  |  |  | Hypothetical protein |
| SB021 contig 3 | DPDHICOG_00485 | CDS | 795 |  |  | COG4753 | Putative response regulatory protein |
| SB021 contig 3 | DPDHICOG_00486 | CDS | 1362 | *yeeO* |  | COG0534 | Putative FMN/FAD exporter YeeO |
| SB021 contig 3 | DPDHICOG_00487 | CDS | 774 |  |  |  | Hypothetical protein |
| SB021 contig 3 | DPDHICOG_00488 | CDS | 435 |  |  |  | Hypothetical protein |
| SB021 contig 3 | DPDHICOG_00489 | CDS | 1518 | *galT* | 2.7.7.12 |  | Galactose-1-phosphate uridylyltransferase |
| SB021 contig 3 | DPDHICOG_00490 | CDS | 1182 | *galK* | 2.7.1.6 | COG0153 | Galactokinase |
| SB021 contig 3 | DPDHICOG_00491 | CDS | 1212 | *nagC_2* |  | COG1940 | N-acetylglucosamine repressor |
| SB021 contig 3 | DPDHICOG_00492 | CDS | 1053 | *mro* | 5.1.3.3 | COG2017 | Aldose 1-epimerase |
| SB021 contig 3 | DPDHICOG_00493 | CDS | 1017 | *mglB* |  | COG1879 | D-galactose-binding periplasmic protein |
| SB021 contig 3 | DPDHICOG_00494 | CDS | 1506 | *mglA* | 7.5.2.11 | COG1129 | Galactose/methyl galactoside import ATP-binding protein MglA |
| SB021 contig 3 | DPDHICOG_00495 | CDS | 1023 | *mglC* |  | COG4211 | Galactoside transport system permease protein MglC |
| SB021 contig 3 | DPDHICOG_00496 | CDS | 879 |  |  |  | Hypothetical protein |
| SB021 contig 3 | DPDHICOG_00497 | CDS | 1827 |  |  |  | Hypothetical protein |
| SB021 contig 3 | DPDHICOG_00498 | CDS | 723 |  |  |  | Hypothetical protein |
| SB021 contig 3 | DPDHICOG_00499 | CDS | 1416 | *argH* | 4.3.2.1 | COG0165 | Argininosuccinate lyase |
| SB021 contig 3 | DPDHICOG_00500 | CDS | 741 | *artP_2* |  | COG0834 | Arginine-binding extracellular protein ArtP |
| SB021 contig 3 | DPDHICOG_00501 | CDS | 1200 | *argG* | 6.3.4.5 | COG0137 | Argininosuccinate synthase |
| SB021 contig 3 | DPDHICOG_00502 | CDS | 375 |  |  |  | Hypothetical protein |
| SB021 contig 3 | DPDHICOG_00503 | CDS | 1188 |  |  |  | Hypothetical protein |
| SB021 contig 3 | DPDHICOG_00504 | CDS | 1626 | *hcp* | 1.7.99.1 | COG1151 | Hydroxylamine reductase |
| SB021 contig 3 | DPDHICOG_00505 | CDS | 660 |  |  |  | Hypothetical protein |
| SB021 contig 3 | DPDHICOG_00506 | CDS | 714 |  |  |  | Hypothetical protein |
| SB021 contig 3 | DPDHICOG_00507 | CDS | 588 | *xerC_4* |  |  | Tyrosine recombinase XerC |
| SB021 contig 3 | DPDHICOG_00508 | CDS | 993 | *gpr* | 1.1.1.- | COG0667 | L-glyceraldehyde 3-phosphate reductase |
| SB021 contig 3 | DPDHICOG_00509 | CDS | 642 |  |  |  | Hypothetical protein |
| SB021 contig 3 | DPDHICOG_00510 | CDS | 510 |  |  |  | Hypothetical protein |
| SB021 contig 3 | DPDHICOG_00511 | CDS | 1236 | *chuW* | 2.1.1.342 |  | Anaerobilin synthase |
| SB021 contig 3 | DPDHICOG_00512 | CDS | 705 |  |  |  | Hypothetical protein |
| SB021 contig 3 | DPDHICOG_00513 | CDS | 390 | *tolR* |  |  | Tol-Pal system protein TolR |
| SB021 contig 3 | DPDHICOG_00514 | CDS | 612 | *tolQ* |  |  | Tol-Pal system protein TolQ |
| SB021 contig 3 | DPDHICOG_00515 | CDS | 339 | *ybaN* |  | COG2832 | Inner membrane protein YbaN |
| SB021 contig 3 | DPDHICOG_00516 | CDS | 2220 | *fepA* |  | COG4771 | Ferrienterobactin receptor |
| SB021 contig 3 | DPDHICOG_00517 | CDS | 444 | *yvbJ* | 2.3.1.- |  | Putative N-acetyltransferase YvbK |
| SB021 contig 3 | DPDHICOG_00518 | CDS | 273 |  |  |  | Hypothetical protein |
| SB021 contig 3 | DPDHICOG_00519 | CDS | 1104 |  |  |  | Hypothetical protein |
| SB021 contig 3 | DPDHICOG_00520 | CDS | 1200 |  |  |  | Hypothetical protein |
| SB021 contig 3 | DPDHICOG_00521 | CDS | 1485 |  |  |  | Hypothetical protein |
| SB021 contig 3 | DPDHICOG_00522 | CDS | 960 |  |  |  | Hypothetical protein |
| SB021 contig 3 | DPDHICOG_00523 | CDS | 1305 | *creD* |  | COG4452 | Inner membrane protein CreD |
| SB021 contig 3 | DPDHICOG_00524 | CDS | 891 | *ywqG* |  | COG3878 | Putative protein YwqG |
| SB021 contig 3 | DPDHICOG_00525 | CDS | 390 |  |  |  | Hypothetical protein |
| SB021 contig 3 | DPDHICOG_00526 | CDS | 582 |  |  |  | Hypothetical protein |
| SB021 contig 3 | DPDHICOG_00527 | CDS | 330 |  |  |  | Hypothetical protein |
| SB021 contig 3 | DPDHICOG_00528 | CDS | 2076 |  |  |  | Hypothetical protein |
| SB021 contig 3 | DPDHICOG_00529 | CDS | 555 |  |  |  | Hypothetical protein |
| SB021 contig 3 | DPDHICOG_00530 | CDS | 765 |  |  |  | Hypothetical protein |
| SB021 contig 3 | DPDHICOG_00531 | CDS | 864 |  |  |  | Hypothetical protein |
| SB021 contig 3 | DPDHICOG_00532 | CDS | 738 |  |  |  | Hypothetical protein |
| SB021 contig 3 | DPDHICOG_00533 | CDS | 525 |  |  |  | Hypothetical protein |
| SB021 contig 3 | DPDHICOG_00534 | CDS | 531 |  |  |  | Hypothetical protein |
| SB021 contig 3 | DPDHICOG_00535 | CDS | 486 |  |  |  | Hypothetical protein |
| SB021 contig 3 | DPDHICOG_00536 | CDS | 135 |  |  |  | Hypothetical protein |
| SB021 contig 3 | DPDHICOG_00537 | CDS | 540 |  |  |  | Hypothetical protein |
| SB021 contig 3 | DPDHICOG_00538 | CDS | 432 |  |  |  | Hypothetical protein |
| SB021 contig 3 | DPDHICOG_00539 | CDS | 300 |  |  |  | Hypothetical protein |
| SB021 contig 3 | DPDHICOG_00540 | CDS | 1083 |  |  |  | Hypothetical protein |
| SB021 contig 3 | DPDHICOG_00541 | CDS | 1284 |  |  |  | Hypothetical protein |
| SB021 contig 3 | DPDHICOG_00542 | CDS | 309 |  |  |  | Hypothetical protein |
| SB021 contig 3 | DPDHICOG_00543 | CDS | 1125 |  |  |  | Hypothetical protein |
| SB021 contig 3 | DPDHICOG_00544 | CDS | 477 |  |  |  | Hypothetical protein |
| SB021 contig 3 | DPDHICOG_00545 | CDS | 498 |  |  |  | Hypothetical protein |
| SB021 contig 3 | DPDHICOG_00546 | CDS | 372 |  |  |  | Hypothetical protein |
| SB021 contig 3 | DPDHICOG_00547 | CDS | 537 | *speG* | 2.3.1.57 |  | Spermidine N(1)-acetyltransferase |
| SB021 contig 3 | DPDHICOG_00548 | CDS | 690 | *ydfK* |  | COG1811 | Putative membrane protein YdfK |
| SB021 contig 3 | DPDHICOG_00549 | CDS | 1323 | *rkpK* | 1.1.1.22 | COG1004 | UDP-glucose 6-dehydrogenase |
| SB021 contig 3 | DPDHICOG_00550 | CDS | 1062 | *wbgU* | 5.1.3.7 |  | UDP-N-acetylglucosamine 4-epimerase |
| SB021 contig 3 | DPDHICOG_00551 | CDS | 1608 | *arnT_1* | 2.4.2.43 |  | Undecaprenyl phosphate-alpha-4-amino-4-deoxy-L-arabinose arabinosyl transferase |
| SB021 contig 3 | DPDHICOG_00552 | CDS | 261 |  |  |  | Hypothetical protein |
| SB021 contig 3 | DPDHICOG_00553 | CDS | 723 | *arnC* | 2.4.2.53 | COG0463 | Undecaprenyl-phosphate 4-deoxy-4-formamido-L-arabinose transferase |
| SB021 contig 3 | DPDHICOG_00554 | CDS | 672 | *arlR* |  | COG0745 | Response regulator ArlR |
| SB021 contig 3 | DPDHICOG_00555 | CDS | 1014 |  |  |  | Hypothetical protein |
| SB021 contig 3 | DPDHICOG_00556 | CDS | 342 | *walK* | 2.7.13.3 |  | Sensor histidine kinase WalK |
| SB021 contig 3 | DPDHICOG_00557 | CDS | 1479 |  |  |  | IS1182 family transposase ISFnu2 |
| SB021 contig 3 | DPDHICOG_00558 | CDS | 1503 | *arnT_2* | 2.4.2.43 |  | Undecaprenyl phosphate-alpha-4-amino-4-deoxy-L-arabinose arabinosyl transferase |
| SB021 contig 3 | DPDHICOG_00559 | CDS | 927 |  |  |  | Hypothetical protein |
| SB021 contig 3 | DPDHICOG_00560 | CDS | 867 |  |  |  | Hypothetical protein |
| SB021 contig 3 | DPDHICOG_00561 | CDS | 1047 |  |  |  | Hypothetical protein |
| SB021 contig 3 | DPDHICOG_00562 | CDS | 1272 | *yihN* |  |  | Inner membrane protein YihN |
| SB021 contig 3 | DPDHICOG_00563 | CDS | 765 | *glcR_1* |  | COG1349 | HTH-type transcriptional repressor GlcR |
| SB021 contig 3 | DPDHICOG_00564 | CDS | 1350 | *alsT_2* |  | COG1115 | Amino-acid carrier protein AlsT |
| SB021 contig 3 | DPDHICOG_00565 | CDS | 939 | *lacC_1* | 2.7.1.144 | COG1105 | Tagatose-6-phosphate kinase |
| SB021 contig 3 | DPDHICOG_00566 | CDS | 852 | *gatY* | 4.1.2.40 | COG0191 | D-tagatose-1,6-bisphosphate aldolase subunit GatY |
| SB021 contig 3 | DPDHICOG_00567 | CDS | 1143 | *agaS* | 3.5.99.- | COG2222 | D-galactosamine-6-phosphate deaminase AgaS |
| SB021 contig 3 | DPDHICOG_00568 | CDS | 954 | *glcK* | 2.7.1.2 | COG1940 | Glucokinase |
| SB021 contig 3 | DPDHICOG_00569 | CDS | 726 | *nagR_1* |  | COG2188 | HTH-type transcriptional repressor NagR |
| SB021 contig 3 | DPDHICOG_00570 | CDS | 1455 |  | 3.1.4.- |  | Multifunctional alkaline phosphatase superfamily protein |
| SB021 contig 3 | DPDHICOG_00571 | CDS | 1704 | *yidK* |  | COG4146 | Putative symporter YidK |
| SB021 contig 3 | DPDHICOG_00572 | CDS | 741 | *nagR_2* |  | COG2188 | HTH-type transcriptional repressor NagR |
| SB021 contig 3 | DPDHICOG_00573 | CDS | 807 | *proC* | 1.5.1.2 | COG0345 | Pyrroline-5-carboxylate reductase |
| SB021 contig 3 | DPDHICOG_00574 | CDS | 825 |  |  |  | Hypothetical protein |
| SB021 contig 3 | DPDHICOG_00575 | CDS | 2286 | *pyrK* |  |  | Dihydroorotate dehydrogenase B (NAD(+)), electron transfer subunit |
| SB021 contig 3 | DPDHICOG_00576 | CDS | 1332 | *mdtK* |  |  | Multidrug resistance protein MdtK |
| SB021 contig 3 | DPDHICOG_00577 | CDS | 864 | *mscS* |  | COG0668 | Small-conductance mechanosensitive channel |
| SB021 contig 3 | DPDHICOG_00578 | CDS | 795 |  |  |  | Putative bifunctional phosphatase/peptidyl-prolyl cis-trans isomerase |
| SB021 contig 3 | DPDHICOG_00579 | CDS | 1851 | *fruA* |  | COG1299 | PTS system fructose-specific EIIABC component |
| SB021 contig 3 | DPDHICOG_00580 | CDS | 930 | *lacC_2* | 2.7.1.144 | COG1105 | Tagatose-6-phosphate kinase |
| SB021 contig 3 | DPDHICOG_00581 | CDS | 723 | *glcR_2* |  | COG1349 | HTH-type transcriptional repressor GlcR |
| SB021 contig 3 | DPDHICOG_00582 | CDS | 372 |  |  |  | Hypothetical protein |
| SB021 contig 3 | DPDHICOG_00583 | CDS | 612 |  |  |  | Hypothetical protein |
| SB021 contig 3 | DPDHICOG_00584 | CDS | 636 |  |  |  | Hypothetical protein |
| SB021 contig 3 | DPDHICOG_00585 | CDS | 1101 | *yxeI* |  | COG3049 | Putative protein YxeI |
| SB021 contig 3 | DPDHICOG_00586 | CDS | 780 | *fepC* |  | COG1120 | Ferric enterobactin transport ATP-binding protein FepC |
| SB021 contig 3 | DPDHICOG_00587 | CDS | 969 | *hmuU* |  | COG0609 | Hemin transport system permease protein HmuU |
| SB021 contig 3 | DPDHICOG_00588 | CDS | 1344 |  |  |  | Hypothetical protein |
| SB021 contig 3 | DPDHICOG_00589 | CDS | 1344 | *mepA_6* |  | COG0534 | Multidrug export protein MepA |
| SB021 contig 3 | DPDHICOG_00590 | CDS | 138 |  |  |  | Hypothetical protein |
| SB021 contig 3 | DPDHICOG_00591 | CDS | 444 | *hgdC* | 3.6.1.3 | COG1924 | (R)-2-hydroxyglutaryl-CoA dehydratase activating ATPase |
| SB021 contig 3 | DPDHICOG_00592 | CDS | 1152 | *fldC* | 4.2.1.- |  | (R)-phenyllactyl-CoA dehydratase beta subunit |
| SB021 contig 3 | DPDHICOG_00593 | CDS | 1479 |  |  |  | IS1182 family transposase ISFnu2 |
| SB021 contig 3 | DPDHICOG_00594 | CDS | 1014 |  |  |  | Hypothetical protein |
| SB021 contig 3 | DPDHICOG_00595 | CDS | 282 | *ihfA_2* |  |  | Integration host factor subunit alpha |
| SB021 contig 3 | DPDHICOG_00596 | CDS | 291 | *ihfA_3* |  |  | Integration host factor subunit alpha |
| SB021 contig 3 | DPDHICOG_00597 | CDS | 1161 |  |  |  | Hypothetical protein |
| SB021 contig 3 | DPDHICOG_00598 | CDS | 453 |  |  |  | Hypothetical protein |
| SB021 contig 4 | MFEAOJMA_00001 | CDS | 2475 |  |  |  | Hypothetical protein |
| SB021 contig 4 | MFEAOJMA_00002 | CDS | 558 |  |  |  | Hypothetical protein |
| SB021 contig 4 | MFEAOJMA_00003 | CDS | 984 |  |  |  | Hypothetical protein |
| SB021 contig 4 | MFEAOJMA_00004 | CDS | 1740 |  |  |  | Hypothetical protein |
| SB021 contig 4 | MFEAOJMA_00005 | CDS | 456 |  |  |  | Hypothetical protein |
| SB021 contig 4 | MFEAOJMA_00006 | CDS | 558 | *kptA* | 2.7.1.- | COG1859 | Putative RNA 2'-phosphotransferase |
| SB021 contig 4 | MFEAOJMA_00007 | CDS | 168 |  |  |  | Hypothetical protein |
| SB021 contig 4 | MFEAOJMA_00008 | CDS | 1395 |  |  |  | Hypothetical protein |
| SB021 contig 4 | MFEAOJMA_00009 | CDS | 759 |  |  |  | Hypothetical protein |
| SB021 contig 4 | MFEAOJMA_00010 | CDS | 426 |  |  |  | Hypothetical protein |
| SB021 contig 4 | MFEAOJMA_00011 | CDS | 873 |  |  |  | Hypothetical protein |
| SB021 contig 4 | MFEAOJMA_00012 | CDS | 252 |  |  |  | Hypothetical protein |
| SB021 contig 4 | MFEAOJMA_00013 | CDS | 549 |  |  |  | Hypothetical protein |
| SB021 contig 4 | MFEAOJMA_00014 | CDS | 141 |  |  |  | Hypothetical protein |
| SB021 contig 4 | MFEAOJMA_00015 | CDS | 273 | *yafQ* | 3.1.-.- | COG3041 | mRNA interferase toxin YafQ |
| SB021 contig 4 | MFEAOJMA_00016 | CDS | 276 |  |  |  | Hypothetical protein |
| SB021 contig 4 | MFEAOJMA_00017 | CDS | 870 |  |  |  | Hypothetical protein |
| SB021 contig 4 | MFEAOJMA_00018 | CDS | 873 |  |  |  | Hypothetical protein |
| SB021 contig 4 | MFEAOJMA_00019 | CDS | 873 |  |  |  | Hypothetical protein |
| SB021 contig 4 | MFEAOJMA_00020 | CDS | 990 |  |  |  | Hypothetical protein |
| SB021 contig 4 | MFEAOJMA_00021 | CDS | 246 |  |  |  | Hypothetical protein |
| SB021 contig 4 | MFEAOJMA_00022 | CDS | 690 |  |  |  | Hypothetical protein |
| SB021 contig 4 | MFEAOJMA_00023 | CDS | 597 |  |  |  | Hypothetical protein |
| SB021 contig 4 | MFEAOJMA_00024 | CDS | 1320 |  |  |  | Hypothetical protein |
| SB021 contig 4 | MFEAOJMA_00025 | CDS | 327 |  |  |  | Hypothetical protein |
| SB021 contig 4 | MFEAOJMA_00026 | CDS | 159 | *insK_1* |  | COG2801 | Putative transposase InsK for insertion sequence element IS150 |
| SB021 contig 4 | MFEAOJMA_00027 | CDS | 228 | *insK_2* |  | COG2801 | Putative transposase InsK for insertion sequence element IS150 |
| SB021 contig 4 | MFEAOJMA_00028 | CDS | 1641 |  |  |  | Hypothetical protein |
| SB021 contig 4 | MFEAOJMA_00029 | CDS | 798 |  |  |  | Hypothetical protein |
| SB021 contig 4 | MFEAOJMA_00030 | CDS | 1200 |  |  |  | Hypothetical protein |
| SB021 contig 4 | MFEAOJMA_00031 | CDS | 138 |  |  |  | Hypothetical protein |
| SB021 contig 4 | MFEAOJMA_00032 | CDS | 1440 |  |  |  | Hypothetical protein |

The *F. sphaericum sp. nov.* SB021 genome was annotated by the Rapid Annotation using Subsystem Technology (RAST) pipeline. Table indicates resulting genes for each contig, with their length and predicted product.

**Supplementary Table 3: Methyl-modified motifs across *F. sphaericum sp. nov.* SB021 genome**

| **Motif** | **Unique** | **Percent Detected** | **Coverage** |
| --- | --- | --- | --- |
| GG*CC | No | 85.7 | 315.1 |
| GGNT*ATT | No | 98.9 | 312.2 |
| CAAAA*AV | Yes | 75.3 | 314.1 |
| YMCAAAA*AT | Yes | 20.7 | 318.5 |

SB021 methyl-modified nucleotides were analyzed using Single-Molecule Real Time Sequencing (SMRTSeq)11 kinetics (Basemod analysis) and the predicted methyl-modified motif acquired via REBASE46 analysis.

**Supplementary Table 4: Average nucleotide identity (ANI) scores between *Fusobacterium* genomes**

|  | KCOM 1261 | 2_1_31 | ATCC 51191 | ATCC 25586 | ATCC 23726 | ATCC 10953 | OH4460  COT188 | ATCC 51357 | CMW8396 | ATCC 25563 | ATCC 25533 | Marseille  P2749T | ATCC 29250 | SB021 | ATCC  9817 | ATCC 27725 | ATCC 49185 |
| --- | --- | --- | --- | --- | --- | --- | --- | --- | --- | --- | --- | --- | --- | --- | --- | --- | --- |
| KCOM 1261 | 100 | 95.45 | 85.5 | 85.29 | 85.35 | 86.15 | 85.66 | 72.01 | 72.46 | 72.48 | 75.72 | 80.41 | 71.43 | 71.30 | 72.32 | 72.74 | 72.05 |
| 2_1_31 | 95.45 | 100 | 85.5 | 85.30 | 85.34 | 86.07 | 85.43 | 71.58 | 72.27 | 72.42 | 75.53 | 80.76 | 72.15 | 71.01 | 71.85 | 72.48 | 72.50 |
| ATCC 51191 | 85.5 | 85.50 | 100 | 91.17 | 91.16 | 90.56 | 90.02 | 72.55 | 72.21 | 72.60 | 75.91 | 81.07 | 71.73 | 71.34 | 72.64 | 72.68 | 72.33 |
| ATCC 25586 | 85.29 | 85.30 | 91.17 | 100 | 98.67 | 91.91 | 92.12 | 72.33 | 72.35 | 72.18 | 75.87 | 81.07 | 71.65 | 71.13 | 72.54 | 72.94 | 72.45 |
| ATCC 23726 | 85.35 | 85.34 | 91.16 | 98.67 | 100 | 91.80 | 92.24 | 72.36 | 72.31 | 72.72 | 75.79 | 80.80 | 71.67 | 71.18 | 72.50 | 72.99 | 72.55 |
| ATCC 10953 | 86.15 | 86.07 | 90.56 | 91.91 | 91.80 | 100 | 92.76 | 72.42 | 72.47 | 72.40 | 75.99 | 81.44 | 71.79 | 71.18 | 72.59 | 72.62 | 72.48 |
| OH4460  COT188 | 85.66 | 85.43 | 90.02 | 92.12 | 92.24 | 92.76 | 100 | 72.03 | 71.99 | 72.29 | 76.27 | 80.91 | 71.87 | 70.90 | 72.47 | 72.60 | 72.37 |
| ATCC 51357 | 72.01 | 71.58 | 72.55 | 72.33 | 72.36 | 72.42 | 72.03 | 100 | 82.69 | 82.46 | 71.40 | 72.68 | 70.54 | 69.53 | 70.20 | 71.15 | 70.36 |
| CMW  8396 | 72.46 | 72.27 | 72.21 | 72.35 | 72.31 | 72.47 | 71.99 | 82.69 | 100 | 98.65 | 71.07 | 72.49 | 70.79 | 69.76 | 71.32 | 71.48 | 71.06 |
| ATCC 25563 | 72.48 | 72.42 | 72.60 | 72.18 | 72.72 | 72.40 | 72.29 | 82.46 | 98.65 | 100 | 71.49 | 72.32 | 70.57 | 69.88 | 71.02 | 71.70 | 70.96 |
| ATCC 25533 | 75.72 | 75.53 | 75.91 | 75.87 | 75.79 | 75.99 | 76.27 | 71.40 | 71.07 | 71.49 | 100 | 75.95 | 70.83 | 70.33 | 71.68 | 72.43 | 71.77 |
| Marseille  P2749T | 80.41 | 80.76 | 81.07 | 81.07 | 80.8 | 81.44 | 80.91 | 72.68 | 72.49 | 72.32 | 75.95 | 100 | 72.06 | 71.21 | 73.02 | 73.14 | 72.57 |
| ATCC 29250 | 71.43 | 72.15 | 71.73 | 71.65 | 71.67 | 71.79 | 71.87 | 70.54 | 70.79 | 70.57 | 70.83 | 72.06 | 100 | 74.98 | 74.14 | 72.92 | 73.13 |
| SB021 | 71.30 | 71.01 | 71.34 | 71.13 | 71.18 | 71.18 | 70.90 | 69.53 | 69.76 | 69.88 | 70.33 | 71.21 | 74.98 | 100 | 73.00 | 72.59 | 72.90 |
| ATCC  9817 | 72.32 | 71.85 | 72.64 | 72.54 | 72.50 | 72.59 | 72.47 | 70.20 | 71.32 | 71.02 | 71.68 | 73.02 | 74.14 | 73.00 | 100 | 76.07 | 75.78 |
| ATCC 27725 | 72.74 | 72.48 | 72.68 | 72.94 | 72.99 | 72.62 | 72.60 | 71.15 | 71.48 | 71.70 | 72.43 | 73.14 | 72.92 | 72.59 | 76.07 | 100 | 88.09 |
| ATCC 49185 | 72.05 | 72.50 | 72.33 | 72.45 | 72.55 | 72.48 | 72.37 | 70.36 | 71.06 | 70.96 | 71.77 | 72.57 | 73.13 | 72.90 | 75.78 | 88.09 | 100 |

Table shows the pairwise average nucleotide identity (ANI) scores between each *Fusobacterium* genome (Table 1).

**Supplementary Table 5: GTDB-tk analysis of *F. sphaericum sp. nov.* SB021 genome**

| **User Genome** | SB021.fasta |
| --- | --- |
| **Classification** | d__Bacteria;p__Fusobacteriota;c__Fusobacteriia;o__Fusobacteriales;f__Fusobacteriaceae;g__Fusobacterium_B;s__Fusobacterium_B sp900541465 |
| **fastani_reference** | GCA_900541465.1 |
| **fastani_reference_radius** | 95 |
| **fastani_taxonomy** | d__Bacteria;p__Fusobacteriota;c__Fusobacteriia;o__Fusobacteriales;f__Fusobacteriaceae;g__Fusobacterium_B;s__Fusobacterium_B sp900541465 |
| **fastani_ani** | 97.33 |
| **fastani_af** | 0.92 |
| **closest_placement_reference** | GCA_900541465.1 |
| **closest_placement_radius** | 95 |
| **closest_placement_taxonomy** | d__Bacteria;p__Fusobacteriota;c__Fusobacteriia;o__Fusobacteriales;f__Fusobacteriaceae;g__Fusobacterium_B;s__Fusobacterium_B sp900541465 |
| **closest_placement_ani** | 97.33 |
| **closest_placement_af** | 0.92 |
| **pplacer_taxonomy** | d__Bacteria;p__Fusobacteriota;c__Fusobacteriia;o__Fusobacteriales;f__Fusobacteriaceae;g__Fusobacterium_B;s__ |
| **classification_method** | taxonomic classification defined by topology and ANI |
| **note** | topological placement and ANI have congruent species assignments |
| **other_related_references**  **(genome_id,**  **species_name,**  **radius,ANI,AF)** | GCA_905193185.1, s__Fusobacterium_B sp900554355, 95.0, 86.03, 0.81;  GCA_900542625.1, s__Fusobacterium_B sp900542625, 95.0, 82.97, 0.68;  GCA_900545035.1, s__Fusobacterium_B sp900545035, 95.0, 82.54, 0.73;  GCA_900554885.1, s__Fusobacterium_B sp900554885, 95.0, 80.33, 0.51;  GCF_000622245.1, s__Fusobacterium_B perfoetens, 95.0, 78.36, 0.41;  GCF_012843705.1, s__Fusobacterium_B sp012843705, 95.0, 78.23, 0.37;  GCF_010367435.1, s__Fusobacterium_B sp010367435, 95.0, 77.11, 0.24 |
| **msa_percent** | 93.27 |
| **translation_table** | 11 |
| **red_value** | N/A |
| **warnings** | N/A |

Table shows the assigned phylogenetic classification of SB021 as determined by GTDB-tk13.

**Supplementary Table 6: Analysis of *F. sphaericum sp. nov.* SB021 anvi’o-derived gene clusters**

| **Anvi’o** | | **KofamKOALA** | | | | | **VFDB** | |
| --- | --- | --- | --- | --- | --- | --- | --- | --- |
| GC | Representative Sequence | KO | Threshold | Score | E-value | Description | Hit | Category |
| GC_00000002 | MKKIPIGVDDFKKIITDNYFYIDKTKFIEEIFNDGAEVKLFTRPRRFGKTLNMSMLKNFFDVREAEENKKLFNNLYIKNSPVFAEQGKYPVVFVSMKEIKGTTWEEMQKSSRETLSNLYEKYKYLRENLDERNKRKFDKIWFEEIDGGYNDALNFLSKILEEQYNEKVIVLIDEYDAPLTMAYEYGFYDRAVVFFKSMYGACLKTNSSLKMGVLTGAIRVAQAGIFSDLNNIETHTILDEAYDEYFGLLENEVENALIEYKTEDKLEDVKSWYDGYKFGNIEVYNPWSILKYIKYKKLDAYWINTSGNALIKELLLLSDGTVFEDLDNLVNGQEKTIYINENVALGNDLDPNRLWELMLFSGYLTVKEKINSEAYLVKIPNKEIKSFFKGLFAEIIFKGKSNIASMKAALESKDINTIIRILEKIVLNAISFYDTNKKLENPYQTLLAGFFYALDDYYEMKPNPETGYGRADIILKPRNKKWSGYIFELKRAKTQNLEKEAEKALKQIEEKKYDTILINEGIKDIIKIGLVFDGKKAVAYY |  |  |  |  |  |  |  |
| GC_00000003 | MEFNIPKTHELFRQMIREFAEKEVKPLATELDEEERFPVETVKKMAEIGLMGIPIPKEYGGAGGDNVMYAMAVEELSRVCGTTGVVVSAHTSLGTWPILKFGTEAQKQKYIPKLASGEWIGAFGLTEPNAGTDAAGQQTTAVFDEATQEWVINGSKIFITNAGYANVYVIFAMTDRSKGLKGISSFIIEAGTPGFSIGKKEKKLGIRGSSTCELIFEDARIPKDNLLGEIGKGFKIAMMTLDGGRIGIASQALGLAQGALDETVAYVKERKQFGKAIAKFQNTQFQLADLEVKVEAARLLVYKAAWRESNHLPYTVDAARAKLFAAETAMEVTTKAVQLHGGYGYTREYPVERMMRDAKITEIYEGTSEVQRMVIAGNLLK | K00248 | 528.77 | 622.6 | 7.60E-188 | Butyryl-CoA dehydrogenase [EC:1.3.8.1] | Colibactin biosynthesis dehydrogenase ClbF | Exotoxin |
| GC_00000006 | MTGKIKELQNEILFLEAKAEYLNKLNSLIEIKKVQKSDKINIIFGLKKKYPLKILLEITNIKRSTYYFHLKKKDMDLKNEDIINKIKEIFYENKRRYGYRRITLELRKQGFIVNHKKVLRLMRKLNLQSILYKRNKKYSSYKGIIGKTPDNYIERNFEAERPNEKWFTDITEFKVKGKKLYLSVILDAYGRYVISYNISSHPNFNQIKDMLEKAFKNNSKITNLILHSDAGWQYQKNFYIKKLEEKKIIQSMSRMGNVLDNGLIESFFSIMKSEMFYGQESSYKNIEELKAAIDEYIDYYNNRRIKLKLKGLTPVEYRNQSL | K07497 | 15.07 | 134 | 1.00E-39 | Putative transposase |  |  |
| GC_00000009 | MAKKMQSMDGNQAAAYTSYAFTEVAGIYPITPSSPMAEYVDEWASKGMKNIFDVPVKVVEMQSEAGAVGTVHGSLQGGALTTTFTASQGLLLKIPNMYKIAGELLPGVMHVAARTLSVQAINIFGDHQDVYATRQTGWAMLASNSVQEVMDLGGVAHLSAIKASVPFLHFFDGFRTSHEIQKVEVMDYEVYKNLIDMEAVEKFRKRAINPEFPVTKGSCQNDDIYFASREAQNKYYDAIPDIVAHYLDEISKVTGRDYKPFNYYGHPEADRVIVAMGSVCETAEEVVDNLIKNGQKVGLVKVHLYRPFSEKYFFNVLPKTVKKIAVLDRTKESGALGQPLYLDVVALFNGREESPVIIGGRYGVGGHDTTPSQVFAVYDELLKDAPMRDFTVGIVDDVNHTSLKETNFTVVSDEDVKACLFYGLGADGTVGANKNSIKIIGDKTDLYAQAYFAYDSKKSGGVTRSHLRFGKKPIRSTYLIEAPTFVACSVPAYIGQYDMIGGLKKGGTFLLNCVWDKEEILNHIPNSVKKVLAEKEAKFYIVNATKLAQEIGLGNRTNTIMQSAFFKLADIIPFEQAQQYMKDYAKKSYAKKGDDIVQLNYNAIDKGADGIVEIPVDPAWKDLVPEENIAHKEYASECSCDATVHYAREIAYPVNHVKGYDLPVSTFNGYEDGTMENGLAAFEKRGIAVNVPEWKIENCIQCGQCAYVCPHAAIRPFLLDEAEVAAAPADMEFKAPIGKGLEGYKYRMQVSIMDCTGCGSCANVCPAKEKALVMIPLDEAKAKNEDKHAHYMFNKVTYKDNLMAKNTVKGSQFAQPLFEFNGACPGCGETPYLKAITQLFGERMMVANATGCSSIYSSSSPSTPYCKNANGHGPSFGTSLFEDNAEYGMGMHVAVETLRDRIQDVMEKGMDKVPAEVAELFKKWIANRTSGAVTQEVKEQLVPMLEAAGDCEVAKEILGLKQYLVKKSQWIFGGDGWAYDIGYGGLDHVLATNEDVNVVVLDTEVYSNTGGQASKSTPTGATAKFAAAGKSFKKKDLAAIAMSYGHIYVAQVSMGANQQQYLKAIQEAEAYNGPSLIIAYSPCINHGIKKGMSKAQTEMKLATECGYWPIFRYNPMLEKEGKNPLQLDCKEPNWDKYQEYLLGEVRYATLAKSNPTEAEALYAKNKADAKRRWKQYVRLAAMDFSNED | K03737 | 507.03 | 1793 | 0 | Pyruvate-ferredoxin/flavodoxin oxidoreductase [EC:1.2.7.1 1.2.7.-] |  |  |
| GC_00000010 | MSKVIHNNIFFNLNQPKIFHLVEYQISNDDPVRKLSKILEAMNFNKLMQVFSHKTKIHPVRMFAVILYAYSRGIYSTRDIEIACHENIKFRFLLQNSKIPDHSTISRFLTKIENLLPELFEQFFKIIFNLENISTDTIYIDGTKIEAYANKYTFVWRGSIEKYSLRLDEKIETLISDFNNDFNADYESFLEICAYLSNLNIKFVKGRGHRKSKEQKYFEKCMEYLEKYQRYSNHFINLRGRNSYSKTDIDATFMRMKDDYMRNGQLKPGYNLQIGVISEYICAYDIFPNPSDSKTLIPFLDKISTLNLNIKNIVADAGYESISNYEYLEKMRYNSYIKPIYFEKSKTRKFKNDLNRVENLVYDSKKNKLFRKDGLELDFLYSNKKGTIHYFFNSETKKKIKYNAKFRMLSDKSKENISSNYGKQLRLNRSIQVEGAFAVLKEDMKLRKLKVRGKPSVLREVGLFCMGYNFNRYISRSLRNCKGTTLHPLQAA | K07487 | 164.43 | 323.8 | 230E-97 | Transposase | Cytotoxin | Effector Delivery System |
| GC_00000012 | MLYLILGVLIFLTVFYLMITEKVPNAWATMLGGLVMALLGIINEENALEAVYERLEIIFLLVGMMIIVHIISETGVFQWFAIKVAQLAKGEPFRLIILLSVVTALCSAFLDNVTTILLMAPVSILLANQLRLDPFPFIISEVMSANIGGVATLIGDPTQLIIGSEGHLGFNEFLMNTSPMAIVSMVILILNVYFIYGKNMHVSRELKARIMELDSSRSLKDVKLLKEAALIFSLVLAGFILNNFINKGLAVIALSGAVILVVLAKREPKEVLNNVEWDTMFFFIGLFMMIRGIENLHVIDIIGGKLIEITAGNFDMALVAITWLSAAFTSVIGNVANAATVSKIVEVMIPSFDKIGNTQAFWWALSFGSCLGGNISMLASATNVVAVGAAGKAGCKIDFIKFLKFGALISIQTLIASTIYMWLRYMN |  |  |  |  |  |  |  |
| GC_00000013 | MNKGTVKWFNAEKGFGFITSEEGKDLFVHFSEIQKEGFKTLEEGEQVTFDVKEGQKGPQAANVVVVK | K03704 | 79.47 | 117 | 150E-34 | Cold shock protein |  |  |
| GC_00000014 | MIRVENLNKYYINGDMKLHALKDINFHIKQGEFTAIMGSSGSGKSTMMNILGCLDKNFTGKYILDKTDISRIEEKDLCRIRNLKIGFVFQSFNLLSKLSAFENVELPLIYAGVPKAEREILVKDVLKKVGLENRMSHKPSELSGGQKQRVAIARAIVNNPSVILADEPTGNLDSVSEEEIMKIFSALNSQGKTVVIVSHEPEIAKYCKRIILFKDGRIIKDGDVL | K02003 | 288.13 | 351 | 1.60E-105 | Putative ABC transport system ATP-binding protein | ATP-binding protein AatC | Others |
| GC_00000015 | MGSYEFKIQSRKEDIDFINKIIEAYEEVGVVRTTDAKAGLITIISTDDYKDTARDIILDLGKNYVKAEILEEGPWKGFL |  |  |  |  |  |  |  |
| GC_00000019 | MAKEKFDRSKPHVNIGTIGHVDHGKTTTTAAISKVLADKGLAERVDFDKIDVAPEERERGITINTAHIEYQTEKRHYAHVDCPGHADYVKNMITGAAQMDGAILVVSAADGPMPQTREHILLSRQVGVPYIVVYLNKADMVDDEELLELVEMEVREILTEYGFPGDDVPVVIGSSLGALNGEEKWVNQIMALMDAVDEYIPTPERTIDQPFLMPIEDVFTITGRGTVVTGRVERGIIKVGEEVEIVGIKPTTKTTVTGVEMFRKLLDQGQAGDNIGALLRGTKKEEVERGQVLAKPGTIHPHTNFNGEVYVLTKEEGGRHTPFFTGYRPQFYFRTTDITGAVNLPEGVEMVMPGDNITMSVELIHPIAMEVGLRFAIREGGRTVASGVVSEITK | K02358 | 345.4 | 817.9 | 7.80E-247 | Elongation factor Tu | Elongation factor Tu | Adherence |
| GC_00000021 | MTKKEFVNLYFEKGGFETKVDAEKKAMAFLAAVEEALVNGDGVTFTGWGKFEVAERAARVCKNPRTGEAVNVDAKKVIKFKAGKMLEESVNR |  |  |  |  |  |  |  |
| GC_00000022 | MKDTFTHLHLHTEYSLLDGVGKIDDYLKRAKELGMKSIAITDHGNMFGAIEMYKKAIKNGIKPIIGIEAYLAEFGMEKKEGRNFHLILLAKNEKGYKNLMKISSEAYINGFYYKPRTDKEFLKNHSEGIICLSACMQGEISRRIIDKEPFENIENAVNSYIEIFGKDDFYIEVQGNGIEGQRELNKKLSEIAEKFNLKLVATNDTHYVNKGDHTLQDIMICIQTGAKFSDEKRMRIETDELYLKSREEMIESLGEEYTEAVDNTALVSSKCNLSIDFGSFKFPYYKLPKCVKNTEEFLRKLVYVGIEERYPNGLTEKILERVEYELSVINKMGYAEYFVVVCDFIHYAKENGIPIGPGRGSAAGSIVAYALKITDLDPIKYNLIFERFLNPERISMPDIDIDICQERRQELIDYVTNKYGKDKVAQIVTFGTLKARAAIRDVGRVLDIPLSKIDRAAKLIPFNFSIEAALKNIPELNKLYTTDKDIENVINISARIENKVRHTSIHAAGIVITKDPLNETVPLYCDKDDVVSTQYQMKELEDLGLLKMDFLGLRNLTNIQRAVDYIEADTGEKIVLHDVPLNVKEVYEMLSKGDSLGVFQLESHGIRRILTKLKPDRFEDIIALLALYRPGPLGSGMVDSFINCKNGREEIKYPHDSLKDILQETYGVILYQEQVMKIANVMADYSLGEADLLRRAMGKKKAEIMDENREKFISRSVAKGYTEEKATEIFDLIDKFAGYGFNKSHSAAYALIAYWTAWLKKMYPKHYYAALMTSEISHIENVALYVEDAKNHGVKLKFPDINNAASKFKVSEDGVVFALSAIKNVGEKVAEGIKKEYDDNGEYKSFDDFIFRTKQFGMNKKAVEALVYSGALDSVPGNRKEKIDSIGKALEFAAKRLKEDEIQQMNLFGDAKSTVNGFSMAKSEDYSMEEKLEKEKEFLGFYFSAHPLDKYKGILQAYRSDKIQDIKDDGDDKPVKIFGILRDTKKIVTKKSGEIMAVFDLEDYYSRISGIIFPRDFSKNMGNFLEGKVVCVTGFLQTDYFNGTESKKVVAREIIPLEILAYERGYTVYILVKDNDRGKVPRLKKILHSYRGETPVSLAVKTAEEKKVVRTKTYVSPTNDFINEIINLMGEGSIVIK | K02337 | 954.2 | 1539.1 | 0 | DNA polymerase III subunit alpha [EC:2.7.7.7] |  |  |
| GC_00000023 | MKIFLATGNKHKIKEIEKIFKMDNVEILSINDGIEIPEVEEDGTTFEENSKKKALEIAKFTNMITIADDSGLCVDALDGAPGVYSARYAGEHGNDLDNNKKLVRELQGIENRKARFVCVITLAKPTGETYSFRGEVEGDIIDVPQGTEGFGYDPHFFMKEYGKTLAEIPEIKNKISHRARALEKLKENLDEILK | K01519 | 53.93 | 254.2 | 2.10E-76 | XTP/dITP diphosphohydrolase [EC:3.6.1.66] | Deoxyribonucleotide triphosphate pyrophosphatase | Immune Modulation |
| GC_00000025 | MKERLIALIEKDYLRTDIPQFKAGDTIAVYYKVKEGNKERVQLFQGTVIRVAGSGIAKTFTVRKVVDGVGVERIIPMNSPMVDKIEVLKVGKVRRSKLYYLRGLSGKQARIKELRK | K02884 | 26.9 | 159.5 | 1.10E-47 | Large subunit ribosomal protein L19 |  |  |
| GC_00000026 | MEAEKKGKKIIFIGDKYHPEVKGIVSFGNDVSIVNSFEEFIELSIDNEKEYCLLTQTTLNKELFFRIKDYIEENYKNVEIFSKICGATYERQKAVEKLAKEVEMVLIVGDNKSSNSKKLYEISKNINEKSYLIQDKTELNSSWFNNVEKIGITAGASTPEEIILEIEKEIRGTFDDKHGL | K03527 | 63.7 | 198.1 | 3.00E-59 | 4-hydroxy-3-methylbut-2-en-1-yl diphosphate reductase [EC:1.17.7.4] |  |  |
| GC_00000029 | MAKRIINTPKAPAALGPYSQAIEVNGTLYISGQIPFVPETMTLVSDDVKAQTRQSLENLKAILDEAGYTFNDVVKASCFIKDMNDFAAVNEVYAEYLGEAKPARACVEVARLPKDVKVEIELIAVK | K09022 | 78.27 | 218 | 2.40E-65 | 2-iminobutanoate/2-iminopropanoate deaminase [EC:3.5.99.10] |  |  |
| GC_00000031 | MIRVEHLYKNFGQLEVLKDISVNIKKGEVIAIIGPSGSGKSTFLRCLNLLEEPTGGAIYIKDKNLMDDKTDINLVRRNVGMVFQHFNLFPHKTVLENLTLAPMKVKNMKQSEIEKKAFMLLEKVGLKEKASAYPNQLSGGQKQRIAIARALAMDPEVILFDEPTSALDPEMIKEVLDVMKDLAEEGMTMIIVTHEMGFAKNVADRVFFMDRGNILEDTTPEELFNNPKHERTQEFLNKVLNR | K2028 | 379.93 | 447.5 | 4.20E-135 | Polar amino acid transport system ATP-binding protein [EC:7.4.2.1] | Iron(III) ABC transporter, ATP-binding protein | Nutritional/Metabolic Factor |
| GC_00000032 | MKTYLVTGAAGFIGANYLKYILKKYKDIFVVVLDKLTYAGNLKNIEDELKDKRVEFVKGDICNNELVENIFSRYDIDYVVNFAAESHVDRSIENPKLFLETNILGTQTLLDTAKKFWTIGKDEKGYPVYKENKKYLQVSTDEVYGSLKKDITEGKELTFNDKDLDILLQNRGEVKTFGNKFFSETTPLSPKSPYSTSKASADMLVMAYQETFHMPINITRCSNNYGAYQFPEKLIPLIINNVLHGKNLPVYGDGMNVRDWLYVEDHCKGIDTVLEKGRLGEIYNIGGFNEETNINIVKLIIDTISKIMTNEPEYRKILKTDLENVNYNLITYIQDRLGHDARYAIDPSKIVKELGWYPETPFVIGIEKTIRWYLDNQEWMENVTSGDYQKYYEKMYK | K01710 | 405.03 | 490.2 | 6.00E-148 | dTDP-glucose 4,6-dehydratase [EC:4.2.1.46] | dTDP-D-glucose 4,6-dehydratase | Immune Modulation |
| GC_00000033 | MSKFKKIETGIDGLYIIEPTVFGDNRGFFLESYNKKEFEEIGITDEFVQDNHSKSKKGVLRGLHFQTRHSQGKLVRVIKGSVFDVAVDLRHDSKTFGKWFGVELSAENKKMFFIPENFAHGFLTLEDETEFMYKCTDLYHPEYDSGIIWNDKDINIEWNFEKYNLKEEDLILSEKDTKHQSFKEYTNKLGE | K01790 | 201 | 294.7 | 7.10E-89 | dTDP-4-dehydrorhamnose 3,5-epimerase [EC:5.1.3.13] | dTDP-4-dehydrorhamnose 3,5-epimerase [EC:5.1.3.13] | Immune Modulation |
| GC_00000035 | MLIFKDYVKMGGMFMKKALMAMFMTAISAVSFAYHIGVVSGTVSQSEDSLRGAEAVMAKYGAADKGGKVIHITYPDNFMQEMETTISQIVSLADDPEMKAIIMTEAVPGTVEAFRRIKDKRPDIILMANTAHEDPEMIADVSDLALYPDAIARGYLIVKAAQDMGAKKFVHVSFPRHLSYEMLSRRRNIMAETAKDLGMEFIDVSAPDPVSDVGVAGAQQYILEQVPNWLAKYGKDTAFFATNDAHTEPLLKRVAEDGGYFVEADLPSPTMGYPGALGIDFNDNEKGNWPAILKKVEKVVTDKGAAGRMGTWAYSYNFSVAQAMGDHAVNVIEGKCDILDFDAVMNTLKANTPGAGWNGSYLVDVNGVERDNYILIYQDTYVFGKGYLHMTDVKVPEKYFDIR |  |  |  |  |  |  |  |
| GC_00000036 | MNTFEESKIYKSEKEIYFMLKIDENGGYIQPVSYTGEIIDSFQFYKIENEYIKKIVSLLLESKEDDFLIDWEKSSSSNIYLDDFYDLAEDLKHVENFVDENFQKIVWSFSENELTLKLEEVDYDTDMLKPSILLNKKYENFEVISEGLIYRKGVIYCFDLLDEDLQMLKKKLDLISKNELEAFLTLSRHYFKHITFDYMGYATEVLEKIEGIPQLTIEKISQDNSLYLKVDLIFSTITQNFINENELEEIVIVNNLEKKIYICTPSGDLDEAVNDVTNSLVRHQRKLGKAYSYFVDDDNLIIINEKVAKEFVTKELLQLAGKYKVVGTDKLSKYNMKPVKPKLIGKLSHSIDFLEGELQLEIGNEKFGLLDVLSKLKKDSYIVLADGTNAVINKKYIEKLEKVFKKVDGKKVKISFFDLPIIDELIEDKILKEQINQGRDFFRGINEIKDYNAPLPSLRADLREYQEYGYKWLCYLVDNKLGGCLADDMGLGKTLQAIALITRTHSLSKRKKRTLVVMPKSLVYNWESEIQKFSPTLTTGIYYGNNRDVSVFKDCEVILTTYGTVRNDIEKIKKYKFELIILDESQNIKNTNAQTTKAVMLLKGNNRIALSGTPVENNLGELYSLFRFLNPPMFGTAEEFNRYYAGPIHRENDESAIDELRKKIYPFILRRIKKDVLKDLPDKIEKTLYVEMNPEQKSFYEERRMYYYNMVHENIKSKGIGKSHIYILQALNELRQLTSCPESKNPHVISSKKEVVVENIMDAVQNNHKVLVFANYIHSIDAICKELKKNKIKYLSMTGATKDRHILVDKFQNDKEYKVFVMTLKTGGVGLNLTAADTIFIYDPWWNKTVENQAVDRAYRLGQDRTVFSYKIILKDSIEEKILKLQETKNKLVENLISEEGTAAKVLSEDDIEFLLGK |  |  |  |  |  |  |  |
| GC_00000038 | MTNTFKRVLLSTAALLILGSASSFANTVLKVGATPVPHAEILNAVKDKLAKEGIDLKVVDFTDYVTPNLALADGELDANYFQHKPYLDKFCEEKGLKLNVAGGVHVEPIGVFSAKLKSIDDIPNKATVAIPNDPSNGGRALILLHNAGIITLNDPTNLYATEFDVAKNPKKLKFKTLEAAQLPRVLKDVDFAVINGNYALEAGLNPAEDSLLLEGKESPYTNIVAVKAGDEKREEIVKLINALNSPETAEFINTTYKGAVVPAF | K02073 | 93.47 | 376.2 | 2.30E-113 | D-methionine transport system substrate-binding protein | Immunogenic lipoprotein A | Adherence |
| GC_00000039 | MVNLGNDWDELLKDEFEKEYYKELRKFLVSEYRTHIIHPDMNDIFSALKVSSYADTKILLLGQDPYHGEGQAHGMAFSVKPGVPAPPSLRNMYKELRDELGCTIPNNGYLMPWAEQGILLLNTALTVRDGQANSHQGKGWEIFTDKIIELLNEKDDPVIFILWGNNAKSKKRLITNPKHFILEGVHPSPLSASRGFFGCGHFKKVNEILKSLGKKEIDWQIPNI | K03648 | 67.37 | 357.3 | 1.40E-107 | Uracil-DNA glycosylase [EC:3.2.2.27] |  |  |
| GC_00000040 | MKKVVKLLGVLMLMLCFAFTANGAEKKEKLYVGTNAEFPPFEYLEEGKVVGFDIDFINAIGEVMGKEIVVKDMSFDGLLPALQTNKVDVVVAGMTATEERKKAVNFSQPYYSANQVIILAEGNNDIKDFADLKGKKVAVMLGFTGDVVVSEMEDVKVERFNAAYAGIMALQNGKVDAVVLDSETANNYVAKNSGLKLAEGKGEAEDYAIAVKKSDAKLLEEINAAIDQIKADGTFGKIIEKHFN | K02030 | 79.1 | 186 | 1.40E-55 | Polar amino acid transport system substrate-binding protein | Bifunctional adhesin/ABC transporter aspartate/glutamate-binding protein | Adherence |
| GC_00000044 | MKDFTIIASSTMGLESIVRDECIELGFKDVHAFNGKVEFKGNFKDVVKANIHLRCADRIFIKMGEFKALTFEELFQNVKKIKWSEIIEKNGEFPISWVSSVKSKLFSKSDIQRIAKKAIVENMKEIYDIEKFPENGPLFRIKIQGNKDIFTIMIDTSGDALHKRGYRNLINEAPLKETMAAALVKLSRWKGGDRPFVDPMCGTGTIAIEAAMIGRNIAPGVNRNFAAESWKVIPKDLWIDLRDEAFSQEDYDNKVMVYASDIDLDTIKIAEENAERAGVEDDILFECKNFLNLEPTAEKGCLVTNPPYGERLLNDEKVERLYTLLGDICRTRFPKWSYYIITSYPEFEKYFDKKATKNRKLYNGGIECHYYQYYGER | K07444 | 421.53 | 540.6 | 5.20E-163 | Putative N6-adenine-specific DNA methylase [EC:2.1.1.-] |  |  |
| GC_00000047 | MNKKYIVALDQGTTSSRAVIFDSEQKIVGVAQKEFKQIYPKEGWVEHDPMEIWASQSGVLAEVIAKEGISQHDIIGIGITNQRETTIVWDKNTGKPVYNAIVWQCRRTAEICDELKKIEGLNEYVKENTGLLIDAYFSGTKIKWILDNVEGAREKAEKGDLLFGTVDTWLIWKLTNGKVHATDYTNASRTMIYNIKKLEWDKKLLNILGIPKSMLPEVKDSSGTFGYANLGGTGGHRIPIAGVAGDQQSALFGQACFNKGDSKNTYGTGCFLLMNTGEEMVTSHNGLITTIAIGLNGKVQYALEGSIFIGGASVQWLRDEMKLVGESADTEYFARKAKDSGGVYVVPAFVGLGAPYWDMYARGAIVGITRGTNKNQIIRATLESIAYQTRDVLEAMQEDSGIKLNHLKVDGGAAANDFLMEFQSDILNSCVRRPVLLETTALGAAYLAGLAVGFWESKEEISKQWILGKEFCPSMSEEERTRRYKGWKKAVKRAMAWELDD | K00864 | 509.67 | 817.1 | 1.70E-246 | Glycerol kinase [EC:2.7.1.30] |  |  |
| GC_00000048 | MMKQLRAYKFRIYPNDEQKIFFSKTFGCVRLVYNLMLNDRIKAYEESKGNPDKKIKYPTPAKYKKQYEFLKEVDSLALANAQINLDKAYKNFFRDKSIGFPKFKSKKNPVQSYTTNNQNGTVNIFENWLKLPKLKELVKIKVHREIEGIIKSVTISRKGSGKYFISLLCETDIQELPKTNSSLGIDLGIKDMAVLSTGEKIENLKFRKQLEDKLKREQRKLSKRLLIAKKENKKLSEAKNYQKQRIKIAKIHEKIMNMRVDFLNKLSMDIIKNHDIICIEDLNTKGLLHNHKLSKSIADVSWANFVNKLEYKAKWYGKEIIKVDRLYPSSQICSVCGNHDGKKTLDVREWTCPICHTHHDRDINASKNILAEGLRIRQAV | K07496 | 46.63 | 399.7 | 3.90E-120 | Putative transposase |  |  |
| GC_00000050 | MYLEILCKIVGGLGIFLYGMENMSGGMQKLAGKRLKKILAVLTTNRVVAVLMGLFVTMLVQSSSVSTVMTIGFVNASLLTLKQGLGVILGANIGTTITGWILVLNIGKYGLPIAGAAAIAYVFLKGDKARTRALTIMGLGMIFLGLELMSNGLKPVRSMPEFIRLFHMFSADTYFGVVKTACIGALITGIVQSSSATLGITITLALQGLIDYNTAVALVLGENVGTTVTAILATLNANANAKRAAYAHTIINITGVLWATTIFPFYIKFLGNFADPNVNMTMAIATAHTMFNVINVIMFTPFVGVLAKFLCFLVKDDKGISTKVTQLDMLMVGTPSVVVGQTKKEVLQMGSNIKEMIFTIEEVFAKKQDITEEQVGKLQAIEDKLDIYQQEITDINFQILNKELDENMKEETRLNLEVCDEYETISDYLVRIAKTLRKLHDNEIVLSEEKIKKLSYMNDAVNTLFKNVNTAYETKDKEMFIKAIVLANHITDKFRETRTYHLENTAKVEGSAMFSTSYMDILNHYRRIRDHIFNIIEVYTR | K03324 | 180.27 | 550.8 | 5.10E-166 | Phosphate:Na+ symporter |  |  |
| GC_00000051 | MIYNLLRILLWIPLKIVMILRPKKRIFIEKRLNQNFDFLKSEKPYIWLHCSSVGEINLSDALIKKLLAERKEDILISIFTDTGYETAQNKYSKEERIKIIYFPLDCKNEIRKILKRIDLKLLILIETEIWPNLISCAKKKGKVILVNGRISDKSFGRYMKLKRILKNVLGKIDHFYMQTPLDGERIETIGADSSKVSVAGNLKFDIELQNYSEEEKNELKEIIKADGRKIFVAGSTRTGEDEVILDAFKHLKDYMLVLVPRHLDRIEKIEDLLIKRKIKFSKYSDCLEGKSEKSEVIIVDKMGVLRKFYSVCDTAFVGGTLVNIGGHSLLEPLFYRKTPIFGKYLQNVKDIADEILKRGIGFKVDSSEDIAVSIGEIDNGHVKIDEIEKFFAANQNTAQKITNEINKLV | K02527 | 130.9 | 379.8 | 2.90E-114 | 3-deoxy-D-manno-octulosonic-acid transferase [EC:2.4.99.12 2.4.99.13 2.4.99.14 2.4.99.15] | 3-deoxy-D-manno-octulosonic-acid transferase | Immune Modulation |
| GC_00000052 | MLDKSIKKVLVIGSGPIIIGQAAEFDYSGTQACETLKSEGIEVVLINSNPATIMTDKTVADRIYVEPITAEFVEKVIAKERPDSLLAGMGGQTALNLAVELTEKGILEKYGVRVIGTSVESIKKGEDREIFRETMEKIGEPCIESEIVESLEHGRDVAKEIGYPVVVRPAYTMGGTGGGIANNPKELEEILLKGLALSRVGQVLIEKSILGWKEIEYEVMRDKNGNCITICNMENVDPVGIHTGDSIVVAPTQTLTHKEAVMLKKSALKILDEVGVIGGCNVQFALHPKSMKYAIIEINPRVSRSSALASKATGYPIARVSTKLALGYTLDEIVNEVTGESYACTEPTIDYIVAKIPKWPFDKFRNANKVLGTKMMATGEVMSIGDNFEAAFLKGLKSLEIGRFNLEHPAVKKLTMEELKKAVVRPDDERIFVVAEMLRRGYIKEKLQKITGIDKFFMEKIEWLVKQEEILKKTKFKDLDVEYLRKLKKKGFSDRGIAELMGVSEEDIRSKRLEHGIIPAYKRVDTCAAEFKAQTSYLYSTYDEHDEVEVENRRKIIVIGSGPIRIGQGIEFDYCTVHSIKTLKKMGIESIIINNNPETVSTDYNTADRLYFEPLILEDVLNIIEKENPEGVILQFGGQTAIKLANALAERGIKILGTSAEKIDEAEDREKFEEMMEELDIKRPKGRAVWDINHGIEIANEIKYPVLVRPSYVLGGQGMEICHDEYNLVKYLESSFDRDPENPVLIDKYLNGIELEVDAICDGEDVLIPGIMEHLERAGVHSGDSITVYPQQNLYEGTEEKVLEITAKISKALGTKGMMNIQFIAYENELYVIEVNPRSSRTVPYISKISGLPVIEIATRIILGETLHEIGYGTGIYKKPNVVAVKVPVFSTEKLSNVEVSLGPEMRSTGEVLGIGNTVDEAIYKGLLAAKRVNKVSGRKVLLTIRDKDKEEFLPVAKELIELGCELFATAGTQKYLEEHGVKAEVVRRVGEEEPNILTMLMNRKVDLLINTPTKANDAQRDGFKMRRTAIEYGVEVLTSIDTLNAIIRMEKKHLKAGELEIFDISKL | K01955 | 1561.27 | 1633.5 | 0 | Carbamoyl-phosphate synthase large subunit [EC:6.3.5.5] | Carbamoyl phosphate synthase large subunit | Nutritional/Metabolic Factor |
| GC_00000054 | MKIVVCIKQVPDTTEIKLDPVKGTLIRDGVPSIMNPDDKGGLEEALKLKDKYGAHVTVITMGPPQAEAILREAFAMGADRAILVTDRKFGGADTLATSNTLAAALRNVEADLIIAGRQAIDGDTAQVGPQIAEHLGLPQVSYVKEMEYNKEDNSLTIKRVVEDGYYLVNVQLPALVTVLSEANSPRYMRVKGIVEAFDREVEVWTADNITVDPAVIGLAGSPTKVKKSFTKGAKQAGKVFELDTKEAVNLIIEKLKEKFVI | K03521 | 78.87 | 267.5 | 2.00E-80 | Electron transfer flavoprotein beta subunit |  |  |
| GC_00000055 | MSYIAGVDIGGTNTKIGIVDEEGEILVKKSIKTLSLEGAEKTMTRIWETIRELLSSLNIDEKDLKGVGMGIPGPVMNKKVVGFFANFPWERNLNVSQMFEIISGKTTRLDNDVNVIALGEAKFGAGKGAGSSVTIALGTGIGGGIYVDGNLISGFTGAGGEIGHMKLVQNGKLCGCGQRGCFEAYASATGIEREAVSRLLVNKTNLLYEKINGDISRLEAKDVFDAAKEGDAFSLEIVDYEAEYLAMGIGNILNIINPEKVILGGGVALAGDILLDRVKDKLPKYALAVTLNNFNIVLGTLGNDAGIKGAAALID | K25026 | 273.93 | 316.8 | 2.30E-95 | Glucokinase [EC:2.7.1.2] |  |  |
| GC_00000056 | MARKKSPKKNLVIVESPAKAKTIEKILGSNFNVTASFGHIRDLPKSTLGVDVENGFKPNYSTIRGKGEITKALKELAKKSDKIYLASDPDREGEAIAWHIAHTLKLDENENNRIEFNEITSSAIREAIKHPRKIDTDKVNAQQARRILDRLVGYGISPLLWKTISANTSAGRVQSVALKLICDLEDTIKNFIPEKFWDIKGKFNDKLELVLYKIDEQKIDKVKDEELVKRVKNLEKENFSVESAKVNKKVKNPPLPLKTSTLQQLASSYLGFSASKTMRVAQGLYEGINIDGTHKGLITYMRTDSTRISEEAKESAKDYILKNFGKEYLGKDAPKKSEKKQENVQDAHEGVRPTDINYEPKNLSKYLDKDQMKLYTLIWERFLISQLAPMKYDQFELICEKEKVQFRGVLNKITFDGYYKIFKDEDEIPLGDFPDIKAGDSMKLSKLLIKEDYTKPPARLTESSLIKKLEADGIGRPSTYATIIDTLKKREYVSLKGKSFVPTELGYEVKYVLNEHFPDIMNEKFTADLENKLDEIAEGKENWQKILEKFYKGLSKNIDKYKTEVEKDENKIIVSDVECPCGSGYMILKNGRFGRYLACPDENCKEKYSLKGVEIPMEDIQAGKIHVKDILKEKMEAKRGKTTDIKTESGAVMLLKFGRFGSYLESENYAEDNIREPLPSEIRKLLAQGAVEEKDGVVLLNEKIKAVKDEEARILSKAGVCEKCGKPFKVGRGRWGKFLACTGYPECRNIKKIDAEGNIVEPKTKEKKK | K03168 | 453.83 | 831 | 2.50E-250 | DNA topoisomerase I [EC:5.6.2.1] | Flagellar hook-length control protein FliK | Motility |
| GC_00000057 | MAVKKSETVDKNKALENAIKQITKDFGEGSIMKLGENAAMNIDVIPTGSINLDAALGLGGVPRGRIVEIYGAESSGKTTIALHIIAEAQKMGGVVAFIDAEHALDPTYAKALGVDIDEMLISQPDFGEQALEIADMLVRSGAVDVIVVDSVAALVPKAEIDGEMSDQQMGLQARLMSKALRKLTGTLNKSKTTLIFINQIRDKIGGFGFGPQTTTTGGKALKFYASVRMEIKRVGSVKQGDDIIGNEVLVKVTKNKIAPPFKEASFQIMYGKGISRVGEVLDAAIEANIVSKSGAWFSYGDIRLGQGKENVKARLEEETDLFNSIFDELKAKNAVGKAKNSEEIDEDVEENEDFEGSEE | K03553 | 99.53 | 585.3 | 1.60E-176 | Recombination protein RecA |  |  |
| GC_00000059 | MYCCTAIGNDIYWVGVNDRKTERFENYLPLPSGVAYNSYLINDEKVCLIDTVEVRQSGTFLNKIEAIIGNKKIDYLVVNHMEPDHSGAMEEILRAHPEIKVIGNAKTLQMIKAFYPSFPEASFQAVKEGDILDLGNHKLTFAMVPMVHWPESMVTYDTTEKILFSNDAFGSFGTLDGGMWDDEVNFDFYKGEMRRYYANIVGKYGVQVTNAIKKLGGLEIKYICPSHGILWRKDVNKVIKYYADWAQFIPEKKGVVVMYASMYGNTEVMANMIARELSQQGLKEIKVYDVSKTDSSFIMSDIWKYKGLIIGSCAHNNALYPKMQPILDKLQNYGLKNRYVGIFGNMMWSGGGVRGIQHFTDSLGGLEVIGKPVEAKGAPTHENMEDLKNLAREMAKRILEEI |  |  |  |  |  |  |  |
| GC_00000061 | MKKVTKAVIPAAGLGTRVLPATKAQPKEMLVIVDKPSLQYIVEELVQSGIEDIVIITGRNKNSIEDHFDYSFELEETLKEKGKEDLLEKVTHLSSMANIFYVRQNHPLGLGHAILKAKPFIGDEPFVIALGDDIVYNDKTATKQLIETYEKYGGSVLGCQEVPEEDVSKYGIVKPTKELDERTVEIEDFIEKPSKEEAPSRLACLGRYLLSGKIFEYLEKTEAGKGGEIQLTDGILKMMKDGEKVTAHNFIGKRYDIGNKIGLLKANIEFGLRNEETREDLINYLKNELEIK |  |  |  |  |  | UTP--glucose-1-phosphate uridylyltransferase HasC | Immune Modulation |
| GC_00000063 | MEKFFKLSHYHTTAKQEIIAGITTFLTMAYIVFVNPVMLSATGMEKGALITATCLSAAIGTGLTGLWVNAPFAMAPGMGLNAFFTYTVVLGQGATWQEALGVVFLSGIVFVILTVTGVRKALIEAIPVEMRLAVGAGIGLFIAFLGMQTMGLIVANPATLVGLGQFTKPVILGVIGFTVMAFLEVKRMKGGILLGIVVTTILGIIMGEISIPDAVVSMPPSIMPVAFKLDIIGALRPALFGSIFSFVFVDLFDSLGTLLACANEAGLIDENGKVEKIDKILEVDAASTMIGSVLGTSTVTTFVESASGIAAGGRTGLTSVTTSILFILTLFFSPIIGVVPGFATAPALIIVGVFMFKNLLDINLRDLETAIPCFLTIIMMPLSYSISIGIAFGFISYVAIKIFVGKAGTVKPILWAVAAFSFLEVSGLLRKLAMMIG | K06901 | 91.63 | 550.6 | 7.60E-166 | adenine/guanine/hypoxanthine permease |  |  |
| GC_00000064 | MFTKETYIERRKVLKENVKNGVILICGNDLSPMNCEDNAYPFMQDSTFLYYFGLNRENLFGVIDIDNDKEYIFGNEITMDDIIWMGPQITLQKQCESVGIKNLSSINELENFILSLKNEKRTLHYIPQYRHSIMIKMSEWLKTSPKEINNHVSRELCFAVANQRNIKTEEEITELEKAVNVTRAMHLAAMEKIKPGMMEYEVAAILENVAKSQNCGLSFPTICSINGQTLHNHYHGNKIKDGDLLLIDAGARLENGYCGDMTTTHPVSGKFTEKQKDIYNLLISMFDKAEELIKPGITYMAVHLAVCKVLAEGMISRGILKGNADEIVEKGVHALFMPHGLGHMMGLDVHDMENIGEPIVGYNGAEKSRQFGLSSLRLGRTLETGFVFTVEPGIYFIPELIEKWKSENKFIEYINYDELEKYIDFGGMRYEGDYLVTETGNRRLGDSMPKYPDEIEKVRAKAF |  |  |  |  |  |  |  |
| GC_00000065 | MERKRRKGINVTEGKRNIITALINEYDIKTAEDIQDVLKDLLGGTIQELLEAEMTQHLGYEPYERSETTNARNGRKVKTIHSKYGEAEIEVPQDREGTFEPQIVKKRQKDISAIEDKIISMYAKGMTTRQISEQIEDIYGFEVSEGLVSDITDKLLPKIEEWQQRPLSNIYPVVFVDAIHFSVRDNGIIKKKAAYIILGINEEGKKDVLSIEIGDNESSKYWLGVFNNLKNRGIKDILILCADGLSGMKEAIAVAFPKTEYQRCIVHQVRNTLKYVSYKDRKPFSEDLKTIYQAPSEQIALKNLEKITEKWEKIYSSSMKSWSKNWDAISPIFKFSMEVRKVIYTTNAIESLNSVYRRLNSQRSVFPSDTSLLKALYLATFEATKKWTSTLRNWGKIMVSFK | K07493 | 47.03 | 492.9 | 1.90E-148 | Putative transposase |  |  |
| GC_00000066 | MYINSPGGVVTAGMAIYDTMNYIKPDVQTICVGQAASMGAFLLSAGAKGKRFSLENSRIMIHQPLGGTQGQAADIEIHAKEIFKT | K01358 | 49.93 | 176.9 | 9.50E-53 | ATP-dependent Clp protease, protease subunit [EC:3.4.21.92] | ATP-dependent Clp protease proteolytic subunit | Stress survival |
| GC_00000067 | MEQKTKKSFFNKFLDFVEVGGNKLPHPVTLFVILSIIIIVVSEICVRAGVTVTYTGFNQKTKVVEEITLSARSLMNAEGLRYIFDTMTPNFTSFAPLGTVLVAMIGVGVAEGTGLIQTVLKKMVMSTPKKLLTAVIVFAGIMSNIASDAGYVVLIPLGAIIFLSVGRHPIAGLAAAFAGVSGGFSANLLIGTIDPLLGGITTESARIFAPDYFVPATANWYFMMFSTIIITVLGTFITEKIVEPRLGDYKGTHDVEAISELSDVEKKGLRWAGISLLIFVLIMGYLTVPADAVFRVNGSLNKFMGNGLLVAIMFFFMIPGIVYGAITKSIKNDKDVANLMGNSLGTLGGYLALSFAAAQFIVYFSYTNLGTIVAVKGAAFLEATGFTGFPLIIGFILVTAFINLFMGSASAKWAIMAPVFVPMFMKIGFTPEFTQAMYRIGDSSTNIISPLMSYFAFIVAYSQKFGKENGMGTLISTMLPFSMCFLVVWSIVLVIWYFLGLPIGPEIYATMPAL | K12942 | 227.77 | 730.5 | 2.10E-220 | Aminobenzoyl-glutamate transport protein |  |  |
| GC_00000068 | MNTDAKTMLEIKDLHVYYDNIHALKGVSLKVKEGEIVSLIGANGAGKTTTLQTISGLIQSRQGKIVFQGKDITKEKPHLICQAGIAQVPEGRRIFARLAVKDNLKLGAFTVKDTPENLEKDRAKFYKDFPRMSERKNQMAGTLSGGEQQMLAMGRAIMSRPKLLILDEPSMGLSPLFVKEIFSVIKKLKEDGVTILLVEQNAKMALSIADYAYVIETGKITLEGPAQELLNNPDIKKAYLGA | K01996 | 318.63 | 399.6 | 3.00E-120 | Branched-chain amino acid transport system ATP-binding protein | Iron(III) ABC transporter, ATP-binding protein | Nutritional/Metabolic Factor |
| GC_00000069 | MSGEADGIVVEAVMIYNTNQRESVYSFVNNIHTHEGGTHVSGFRTALTRVINDVGKSQGFLKDKDGKLQGSDIREGLSAIISIKIAQPQFEGQTKTKLGNSEVTGIVSNIVGTQLKMFLEDTPADTKIIIDKVLNSKRAREAAQKARELVLRKSALEVGSLPGKLADCSSKNPDECEIYIVEGDSAGGSAKQGRDRRFQAILPLRGKILNVEKAGLHKALENAEIRAMITAFGAGMGENFDISKLRYGKIILMTDADVDGAHIRTLLLTFFYRYMVELLHNGNIYIAQPPLYKITTGKTIQYAYDDKQLKFIVDNLEGENRKYALQRYKGLGEMNPEQLWETTMDPDTRTFYQVKVDDAREADMIFDKLMGDKVEPRRKFIEDGAEFVKNLDI |  |  |  |  |  |  |  |
| GC_00000071 | MKKLNVVLMGAALLLTACGGAKESSQEAKVMKLGGIGPLTGPLAIYGVTSTNGSKLAFEEINKNGGILGKQVEYIVLDEKGDSTEAVTAYNKLVDEGVVALIGDITSKPSLAVAEVAAQDNLPMITPTGTQFNITEAGPNVFRVCFTDPYQGVVLANFAKNNLNAETAAVVVNNSSDYSDGVAKAFVEQAEKLGLKVVAKEGYSDGDKDFRAQLTKILPTNPDVLVVPDYYEQVALITTQAREVGIKATFVGPDGWDGVAKTLDASAYGAVENSYFTNHYSLQDQSPKVQNFLKAYKEAYNEEPSAFSALSYDAAYMMKAAIEKAGTTDKQAVVDALKNLDYDGVTGHLTFDENNNPVKAVTVLKIVNGEYTFDSVVEPTK | K01999 | 164.03 | 340 | 4.30E-102 | Branched-chain amino acid transport system substrate-binding protein |  |  |
| GC_00000072 | MNKERLLEIIENFRKVKMAVIGDLMIDDYIIGNVERISPEAPVPVVSVKEERFVLGGAGNVINNLATLGVKTYCYGVIGDDIDGDRLKKSLKLLGVNTEGLIRSEDRPTIVKRRILGGNQQLLRIDWEDPTNINGILEETILSNLKANIENIDAIILSDYDKGVLTERVAKEAIKLARKYGKIVTVDPKPSNIMNYVKASSMTPNKKEALECAKLPKGTDIDTVGTTIREKLQLDNLLLTRSEEGVSLYDSEGVANIPTFAKEVYDVTGAGDTVISVYTLAKAAGASWTEAARIANTAAGVVVGKIGTSTVTKEEIIDFYNDIYKEWN |  |  |  |  |  | ADP-heptose synthase | Immune Modulation |
| GC_00000073 | MAELKGRALIASRCAKFFKDGDFVNLGIGVPLMCVNYLPEGVDLWLEAEIGTVGSGPSPSWDDADIDTIDAGGMPASVIPGGSVYDHTASFGFIRGGHIDIAVLGTLQVDQEGNIANWKIPGKLVPGMGGAMDLCAGVKRIIVATEHCEKNGSSKILKKCTLPLTGKNCVTDIVTERCYFKVTPEGLVLKELAPGYTVEDILAATEAEVIVPDEIGVMG |  |  |  |  |  |  |  |
| GC_00000074 | MDSLKELFKIGCGPSSSHTMGPERAAKKFGKENENAHSFRVELYGSLAATGKGHLTDWIIEETLKPRKTEIVWMPEYVHPYHTNGMKFIALDENGNILNEWLVFSVGGGTIKDLSDAGSKTEEVYKLNKLDDIIKWCKENNKELWQYVEECEGKEIWNHLKDILDTMHNAVSEGIEKDGILPGKLKLQRRAKGFYDKIKGQHNYHVITKKIFAYALAVAEQNSSAGTIVTAPTCGAAGVIPGLLRAMREEHSLDEETCLKALAIGGLIGNLIKQNATISGAEAGCQAEIGAACSMASAMAAYILGGTLEQIEYAAEMGMEHHLGMTCDPVGGYVQIPCIERNAIVAVRSLNTADYALSTDGHHTISFDQVVITMKETGKDMCPSYKETSIGGLAKYYEQFLKD | K01752 | 83.93 | 504 | 1.10E-151 | L-serine dehydratase [EC:4.3.1.17] |  |  |
| GC_00000076 | MKVGIIGAGTMGSGIAQAFAQTAGYEVCLCDINEQFAANGKAKIAKGFEKRIAKGKMEQAAADEILAKITTGTKEICGDCDLIVEAAIENMEIKKQTFKELQEICKPEAMFATNTSSLSITEIGAGLDRPVIGMHFFNPAPVMKLVEVIAGLNTPAEMVEKIKAISEEIGKVPVQVEEAAGFVVNRILIPMINEAVGIYADGVASVEGIDAAMKLGANHPMGPLALGDLIGLDVCLAIMEVLYAEMGDSKYRPHPLLRKMVRGKKLGMKTGKGFYDYTK | K00074 | 407.43 | 434.2 | 8.70E-131 | 3-hydroxybutyryl-CoA dehydrogenase [EC:1.1.1.157] | Colibactin biosynthesis dehydrogenase ClbD | Exotoxin |
| GC_00000077 | MTNLKEKGIGTQAIHAGQEKNPFGALATPIYQTSTFIFDSVEQGQARFMGEEEGYIYSRTKNPTVTVAEEKVAMLEKGEHAMATSSGMGAISSTLWTLLKSGDHILADKTLYGCTFALLSHGLTKFGIEVDFIDTADLEMVKEKLRPNTRVVYLETPANPNLKITDIEEVAKIAHSNNYAKVVVDNTFATPYNQNPLTLGADIVVHSATKYINGHGDVLAGFVVGTKEMVEEIRGNGLKDMTGAILGPTEAYYIIRGLKTFEVRMQRHCENAMKIAKYLEAHPKVEKVYYPGLESHEGYEIAKKQMKDFGGIMSFELKGGFEAGKTLLNNLELCSLAVSLGDTETLIQHPASMTHSPYTREERYAAGITDGLVRLSVGLENAEDIIADLEKGLAEIK | K01761 | 493.7 | 622.4 | 4.70E-188 | Methionine-gamma-lyase [EC:4.4.1.11] |  |  |
| GC_00000079 | MELKGTKTEKNLWTAFAGESQAFTKYNYYASKAKKDGYVQIGELFEATANNEKEHAKIWFKLLHDGMPSTVENLKDAAAGENYEWTDMYLNFAKEAREEGFEKIAVLMEGVAAIEKEHEERYRKLLENVETQTAFKKLDIVVWECGNCGHLHIGTEAPKVCPVCDHPQSYFRVRAVNY |  |  |  |  |  |  |  |
| GC_00000080 | MDEKIKNTYELWLNSTYIDEADRDELKSIASDEKEIEERFYTNISFGTAGMRGIRGVGTNRINKYMIRKATQGLANYIIATTGEEGMKKGVAIAYDCRVGSTEYAMNTALVLAGNGIKAYLYESLRSTPELSFAVRELKCQAGVMVTASHNPQEYNGYKVYWDIGGQIVEPQASGIVNEVNKVEKFEDIKMITEEEAKAQGLLVMIGKEIDDRFIEEVKKEAILTDIPGKKDFKIVYSPLHGTGRRPVQRILKEMGFESVYTVKEQEEPDGMFPTCPYANPEDHKVFALSTKLADEIGAKVCIANDPDADRTGLALKKENGEWFYPNGNQIGMIFMDYVLKMTKDLPKNGAVVSTIVSTPILDDIAKAYGVKVWRTLTGFKYIGEKIEQFKNKELDGTYLFGFEESIGYLKGTHVRDKDAVSASLLLAEIAAYYDSIGTSMPAELDKIYDRFGWYGEETISITKTGMDGAKQIAKIMEVLREKEIKEILGRKVLTLNDFKKQIEIDYENCNRKTIELPKADVLQFILEGDVKVTVRPSGTEPKIKYYLYVKEDTKEKADASLKEFGENFVKFVDSLI | K01835 | 371.57 | 440.1 | 2.360E-132 | Phosphoglucomutase [EC:5.4.2.2] | Phosphomannomutase | Immune Modulation |
| GC_00000082 | MAKLKDSLILKLILGVIVGLVVGLYSNETVIGLVNTIKFLLGQVISFTIPLIILGFIAPAITSMKSNASKMLGVMIGLAYFSSVGAALMSMVAGYSLIPMLNIVSAADSAKKVIPALIFKVEIPPAMSVMTALVLAIFVGLAVVWTNSKNFENVLVEFGNIMLAIVTRVVIPILPLFVATTFATLSYEGVILKQFPVFLKVIVIVLIGHYIWLALLYTIGGIVNKCNPMDVLKYYGPAYLTAVGTMSSAATLPIALSCARKSPVLKEEIVNFGVPLGATVHLCGSVLTEVFFVMTVSKILYGQLPSVGTMVLFIILLGVFAVGAPGVPGGTVMASLGIIISVLGFDEAGVGLMLTIFALQDSFGTACNVAGDGALTMILNGLFKDEVGTAK |  |  |  |  |  |  |  |
| GC_00000083 | MAVLHVTKDSFEKEVLKSDIPVLVDFWATWCGPCRALGPILDEVSEEVSSVKIVKVNVDEESDLAGDFRIMSIPTMILFKDGKPVEKSVGLLQKSEVLDLIKK | K03671 | 112.47 | 145.3 | 3.20E-43 | Thioredoxin 1 |  |  |
| GC_00000084 | MENYSYSKRKVGLPPGTIFYTGDKADKNVNMELYSFDGKETIKKNFKEDSDLSFLKEEGKLIWFNVNGIHNTELINKIGNILNINSLVLEDLTNVNQRVKIEVWEDYLFIVLKMAGFSTRNKNIEYEQVSFILGKNYLITFQEKQGDVFDTVRNRIEAPSSKVFQKGIGYLTYALIDVIADNYFVVLDRLEEKTDRLERKILNEFSDKLAEKILQLKGELAVLKRGIYPIREVSAKFQNEDTILYFGKSNKMYLADLHDHGITICDAIENMISRTSELFQLYYSILSNDMNNVMKVLAIISTIFMPLSFLAGLYGMNFSYMPELAWRYGYFIILGIMAVLVVGMLIVFKKKKWL | K03284 | 163.23 | 304.1 | 2.80E-91 | Magnesium transporter |  |  |
| GC_00000085 | MNLNDYKGILVFAEQRDGVIQNVGLELIGKAKELAAKLEVPVTAALIGDNVEGLAKTLIEYGADKVLVVNDARLAIYDTEAYAQVFKAIIDAKKPEIVLFGATTLGRDLAPRVSSRMNTGLTADCTKLEISEETKGLEMTRPAFGGNLMATIICPDHRPQMSTVRPGVMQKLAREEGRQGEVENFHVSLDTSKMRVKVLQVVKETANKVDISEAKILVSGGRGIGSAENFAALEAVAKQIGATVSASRAAVDAGYIEHDRQVGQTGKTVRPDIYFACGISGAIQHVAGMEESEYIVAINKDKDAPIFGIADLGIVGDANKIAPLLAEELKKAIEAK | K03522 | 227.93 | 418.6 | 6.00E-126 | Electron transfer flavoprotein alpha subunit |  |  |
| GC_00000086 | MKGEVRMTREEFYSDFKLIMVYINEAQKSRLEEFFEEIRFYYYAVQRKLETVWSDKIKHKNSTIWPGTDCIFMLSVPNCDVDKMLKYLKTFRMSLPEGIVMSVGIVPMDRVIPRMYEEDIPVDEELLEKLKNKYTK |  |  |  |  |  |  |  |
| GC_00000087 | MDFITYEQEGFVGVITINRPKALNALNSGVLDELNACLDAVDLETTRALVLTGAGEKSFVAGADIAEMSTLTKAEGEAFGKKGNDVFRKLETFPIPVIAAVNGFALGGGCEIAMSCDIRICSDNALFGQPEVGLGITPGFGGTQRLARLIGAGKAKEMIYACTNVKAEEAKALGLVNAVYTQEELLPAAKKLAGKIAKNAPIAVRACKKAINDGLDAPMDEAIVIEEKLFGSCFETEDQREGMGAFLEKRKVEGFKNR | K01715 | 329.8 | 361.9 | 5.70E-109 | Enoyl-CoA hydratase [EC:4.2.1.17] |  |  |
| GC_00000088 | MHCVRKVTEDLYWVGANDHRLNLFENIHPISRGVSYNSYLLMDKKTVLFDTVDWSVCRQFLENIEAVLGGRNLDYLVVNHMEPDHAASLEEVLLRYPKVKVISTEKAFMLMHQFGFDVEERKEVVKEGDEKSFGKHVVTFVSAPMVHWPEAMVTFDKTNGVLFAADAFGSFGALDGKLFNDEVNFDRDWIDEARRYYTNIVGKYGPHVQALLKKAAGIDIKIICPLHGPVWRSNLGYFIDKYDKWSRYEPEEKGVMIVYASMYGNTESAATALASRLVERGIKNVVMYDVSNTHVSQLISETFKYSHIVLASVTYNLGIYPLMHNYLMDMKALNVQNRTVAIVENGSWACKSGDLMAEFLDGMKQMSVLNERLTVVSSLSEEKVDEMEVLIDGIVDSMKD |  |  |  |  |  |  |  |
| GC_00000089 | MSLIGKKVNEFKTMAYHNGEFKEITSENLKGKWNVFVFYPADFTFVCPTELEDLAEHYEKFKAEGCEVYSVSTDTHFVHKAWHDTSERINKIKFPMLADPTGKLSRDFEVMIEEEGLALRGSFVINPEGTIVAYEVHDTGIGREASELLRKLQAAKFVAEHGEVCPAKWRPGSETIKPSIDLVGKL | K24119 | 240.8 | 339.3 | 1.80E-102 | NADH-dependent peroxiredoxin subunit C [EC:1.11.1.26] | Alkyl hydroperoxide reductase subunit AhpC | Stress survival |
| GC_00000091 | MNENYIHIEETKNLKDDTVLESLRTSYRKKIWRKFVKAINDFNLIEDGDKIAVGVSGGKDSLLLCKLFQELKRDKSKNFEVAFISMNPGFEAMDMEQFEKNLKILNIPCEIFNSDVWKIAFHEDPENPCFLCAKMRRGVLYKKVEELGFNKLALGHHFDDIVETALINMFYAGTIKTMIPKVSSTSGKLAVIRPMAYVKESDIISFVKRNEIKAMGCGCPVESGKVDSKRKEIKLLLKDLEEKNPNVKQSIFNSLKNINLEYVMGYTRGNKEK | K14058 | 173.47 | 310 | 3.40E-93 | tRNA 2-thiocytidine biosynthesis protein TtcA |  |  |
| GC_00000092 | MQEFDVIVVGAGHAGCEAALAPARMGLKTAIFTISLDKIGYLSCNPSIGGPAKSHLAREIDALGGEIGRNIDKTFIQIRVLNTKKGPAVRSLRAQADKPRYHIEMKKTLENTENLEVVQGMVTEIVTEGNKAVGIKTKEGVEYRAKTVVIATGTFMRGLIHVGETHFSGGRMGELSSEELPLSLEKLGIKLGRFKTGTPPRIDARTIDYSKTEEQPGDTEILKFSNRTSDEEIRTRKQIPCHILFTNKNVHEIIRSNRHRSPLFNGTIHGIGPRYCPSIEDKIFRYPDKERHHLFLEKEGYDTNEVYVSGFSSSLPSDVQYKMLNAIEGLEHAKIMRYAYAIEYDYVLTEEIGYSLESKKIENLFMAGQINGTSGYEEAAAQGIMAGINAARKVQGKEPIVLDRADSYIGTLIDDIVSKGTNEPYRMFTARSEYRLILREDNADLRLSKIGYEIGLLPKEEYDKVLYKEKVVKEIIEKLEKQHIGSSNPRVNEVLERCGEEPIKNGITLFELLRRPNIVYDDIKYVAELIDGFELGNYISDIEYQVEVQVKYSGYIERSLRMIERHKSLEEKRIPADIDYDSLENIPKEAKDKLKLVRPMNIGQASRISGVSPADIQVLLIYLKMRGNN | K03495 | 388.8 | 904.9 | 5.70E-273 | tRNA uridine 5-carboxymethylaminomethyl modification enzyme |  |  |
| GC_00000093 | MENLTYFTSEFVSPGHPDKVSDQISDAVLDACLANDPDSRVACEVFCTTGQVIVGGEITTKTYVDIQDIVRKKIDEIGYKPGMGFDSDCGVLNAIHAQSPDIAMGVDVGGAGDQGIMFGGAVKETPELMPLALVLAREILVELTRLTRNSTLSWARPDAKSQVTLAYDRDGKVQFVDTVVVSVQHNPFVTQEQIHHDVKEYVIRPVLTRYKLNFDDVKKVHINPTGRFEIGGPHGDTGLTGRKIIVDTYGGYFRHGGGAFSGKDPSKVDRSAAYAARWVAKNIVAADLADKCEVQLSYAIGVAEPTSVKVETFGTGKVDVIELENAVKSIFDLTPRGIERDLKLRSCEFKYQDLAAFGHIGRTDIKLPWEELNKAEELKRYFNK | K00789 | 18.53 | 566.6 | 7.50E-171 | S-adenosylmethionine synthetase [EC:2.5.1.6] |  |  |
| GC_00000094 | MKKTKIVCTIGPKTESKEVLKQLLEAGMNVMRLNFSHGDYEEHGRRISNLREVMAETGMRAAILLDTKGPEIRTIKLEGGNDVSLVAGQEFTFTTDRTVIGNNKIVAVTYEGFASDLKAGDTVLVDDGLIAMEVKEVAGNEVRCIVKNNGDLGENKGINLPNVSVNLPALAEKDINDLKFGCEQGIDFVAASFIRKAEDVLAVRKVLCENGGENVKIISKIENQEGLNNFDEILEVTDGVMVARGDLGVEIPVEEVPFAQKMMIEKCNEAGKPVITATQMLDSMIKNPRPTRAEANDVANAIIDGTDAVMLSGETAKGKYPVEAVKVMARIAEKTDPLIYTNVEFDNEESTITEAVAKGTVDVAEALEAKLIVVGTGTGRAAKSLRKYFPTARILALTNSQTTANQLLLSRGVLSVVAEKPETLDCFFKQAEAEAVKTGLVKSGDIIVVTCGEQVYVQGTTNTMKVIKVK | K00873 | 54.57 | 667.5 | 2.00E-201 | Pyruvate kinase [EC:2.7.1.40] |  |  |
| GC_00000095 | MNTEIFNYTFGMPETLAIAVALLLLGRWIKEKVTILKKFFIPAPVVGGLIFSIVTLIGHEYNLFHFSFDSQLKSLLMLAFFTTIGFAASIKMLLKGGLSVMIFLVVSIILIIIQNVVGVTLCGVLGINKLLGLAAGSIALTGGHGTAAAFGPELVKAGAEAGVSVSVAAATFGLVAGCMIGGPIAKRLMHKYNLKSTSEGENTEIVEGKLTKDEKNIDENLLFNALMYIIISMGIGSFIILGFKKLGMVFPAYLGPMIVASIIRNFMDKKGKSLPLHCINIIGSISLQLFLGIALMTMQLWELANLAVPLISILLVQTVIMALYTYFVTFRIMGKDYDAAVIATGHCGFGMGASPNAIANMETFTKANGYSPKAFFVVPIVGAMFIDFVNAPIITFFIEVLKTK | K03312 | 113.47 | 439.1 | 2.30E-132 | Glutamate:Na+ symporter, ESS family |  |  |
| GC_00000096 | MNENLKERRSWFVELLKLSPVVILGVLMVGVGLDVLVAAPIAVIFAAIVACITEKFSFQKIVDSAVDSVKEIQLVFFILMLAYAMAEAFMATGVGASIIIMSLNFGLTAKTIAVTGIIVTSILSVAIGSSWGTFAACAPVFLWLNHIVGGNIVLTTAAIAGGACFGDNIGLISDTTVVSSGIQKVEVIDRIKHQGVWSLSCLVLSIITFYGVSVFMGLPNTVGSASEAINNIPQSVWSALAAERESAVTLLRQVQSGIPAYMGVPLFLVLLAAIKGLPTLLCLFLGIVSSLVFGIFAGTVTITPEAAEAAALAGKSAIDLKGFIGLVYTGFEGAGSWVIVMMMWIAAFGGIMGSMNAFEPLSRLVKSLAKNVRQLMFYNGVFSILGNIALADEMAQIVTIGPIIRNLVDENVEGDEKAVYKLKLRNALFSDALGVFGSQLIPWHVYIGFYIGIAKVVYPLYDFSPMDIIKYNFLAMISVFSVLFLTLTGLDKFIPLFGLDTEPKVKLKK | K03315 | 155.97 | 172.1 | 1.50E-51 | Na+:H+ antiporter, NhaC family |  |  |
| GC_00000097 | MEELNQIVQNVSGFVWNSLLIFLLVGTGIFYTIKLKFIQVRKFGEGYTKVVKGVNLNGEAAGADGMSSFQSLATAVAAQVGTGNLAGAATAIAAGGPGAIFWMWISAFFGMATVYSEAVLGQVYKKKIGGEVIGGPAFYISEGLGENWFSKGLAAFFGLSCILALSFMGNAVQSNSIAAAFSNEFPIPPVYIGIGVAVVSGFIFWGGTQRIASFTEKIVPLMAGLYVGASIIIILMNFSHIIPAVKMIFVGAFNPTAVMGGAFGITIKQAVRFGVARGLFSNEAGMGSTPHAHAVAKVDHPCEQGAVAIITVFIDTFVVLTCTALVIIMNVDLTDKALMDSIPGIALTQAAFKMSFGNFGGIFIAVCLFFFALSTIIGWYYFGVANVKYLFGNKAMNIYKVIVICFILLGSLAKVDLVWNLSDLFNGLMALPNLIALLVLYKLVVKATDEYRKLHPVKTKEKEQSAVKELKA | K03310 | 266.3 | 568.6 | 1.70E-171 | Alanine or glycine:cation symporter, AGCS family |  |  |
| GC_00000098 | MIFNPVILSVTAMMVLCLLKLNVIIAILISALIAGVAAGMGLGETMSILINGMGGNSETALSYILLGTLAVAINNSGLAKIIALKISGIVKDKKYLLIFILAGIACSSQNLIPVHIAFIPILIPPLLTLMNKLKIDRRAVACALTFGLKAPYIVLPVGFGLIFQTIVKDQLNANNVPITLEGVSSVNWILGLGMFLGLLTAVFFSYRKPREYKMKEDENKSSESYAFEKKHLFALIGAVIAFGVQLKTGSLPLGALFALIFMAVTNVIPWKHIDETLDGGLHLMGFIAFVMLVAAGYGNVIRETGAVPELVEGVIGIIGTNKILGAFVMLLVGLFVTLGIGTSFGTIPILAVIYCPLAVSLGFTPAGTAVLVAAAGALGDAGSPASDSTLGPTAGLNADGQHQHIWDTCVPTFLHYNIPLIIFGLIGSLIF | K07084 | 344.03 | 696 | 1.00E-209 | Putative amino acid transporter |  |  |
| GC_00000099 | MGEKKLNDEKPLWKMDKKDITEEQYNEFYKHTFYDFEDPLLHFHFKVQGSLEYTALIYIPKRTPMDFYTRDYKRGLQLYSKNVFIMDKAEKLIPEYFSFMKGVVDTDNLSLNISREILQQNDELEKIAKNIEKKITAELQKLMKDDKEKYITLWEAFGRSIKFGVQDMYGIYKDKLKDLLIFKSSFEDKYVTLKEYVERMKEDQKEILYVSGENIEVLKSLPKMKVLKEKGLEVLYLTDRIDEFTIKALMEYEGKQFKSINASDFELDDEKTKEEKENLAKENKPLLERIKNLLGEKIAEVKLNNSLGSGAAGLSAKGEISLEMEKTLSQIPGNENIKAEKILELNPNHPVFEKIKSSDDETLKDLIYILYNESLMIEGFAMENPVEFAEKVNKVLSK | K04079 | 94.37 | 397.1 | 2.90E-119 | Molecular chaperone HtpG |  |  |
| GC_00000100 | MENVYDVLKDRGYLKQFTDEEAIKKLLGEEKITFYIGFDPTADSLHVGHFIAMMFMAHMQRFGHRPIALVGGGTAMVGDPSGRTDMRAMMTKETIAHNVACIKKQMEKFIDFSDGKAILENNADWLLGLNYIDFLRDIGAEFSVNKMLAAECFKSRMEKGLSFLEFNYMLMQGYDFYVLNQKHNCKMELGGDDQWSNIIAGINLVRKKKQETVYGMTCTLLTNSEGKKMGKTAKGALWLDPEKTTPHEFYQYWRNVADEDVEKCLALLTFIPMDEVRRLGALKDAEINKAKVVLAYEITKMIHGQEEADKAKAAAEGAFGGSGDMSNLPTTEIEKANLGVSLIDYLTENGILKTKSEGRRLIQQNGMSLNGDKVTDIGFALTEETFKDNETIVKMGKKKFHRIVLK | K01866 | 77.7 | 397.8 | 1.50E-119 | Tyrosyl-tRNA synthetase [EC:6.1.1.1] |  |  |
| GC_00000101 | MKKIFLTALAAAVMFAGCGKEKEEAKPVEKIKYVVTEPAQIRKMSQIFKTDAVLTPEGKIDHKTEKGGTIEKILKRNGDTVKKGELVMELSDSATESAYLSAKANFASSEASYKIARNNYNKFKKLYDKELISHLEYVEYENAYVNAKGAYESSKAAFENAKNDYNKLFRKADIDGIVGNLFGKEGNEVSADEVVFTVVDDDNMESYVGFPAEWLGQIKVGGPVTVEVPAAGKTLEGKILEINPIADTETKKFKIKFGIENKDRTVKDGMYAYATVPVGEVNVLSTKDSAVFIRELLSYVYKVEDGRARRIEITTGATNQPYTAITSDTIKEGDIIVVDGIFGLEEGDKVEEAGTEKSAEAQAK |  |  |  |  |  | Membrane fusion protein MtrC | Antimicrobial activity/Competitive advantage |
| GC_00000102 | MIKEKLNQVYKSDALGGFLKYSLRYKGWLIWTIFISSLSSALGAAPAWLTKYLVDDVLISKNGKVMLLIGIAIFATTVLKLITAYYSSTTSAYLTEKIRRDIKIDIFEHIENLPMSYFTQNKLGDIMARLSGDSSSLGRIGFLLFEMLREFITVLIFLIRMFQVDFILALVSITVLPSMLAVVKKYTKKMRKSGKIRQDTIGQATAFMQESLAGIQVIKGFNKTDTVINKYKEVTMGELERIYRTQKIKAKISPLNELLATVMILLVAAYGGYQIVYTGAITAGDLISFITAIGLMQSPLKKLISRNSELYEIIPSGDRVMEVLKEEKEVDSYSENPKSFNENIKSIKFENVDFIYPNSEVKVLKNINLKVNKGEVVAFVGSSGSGKTTLVNMIPRFYDPVSGSIKINDIDIRDYSLKDFRGHIGMVPQETFLFSGTIAENISFGKEGITREQIEEAAKMANAYNFIIDLDKGFETEVGERGVLLSGGQKQRIAIARALIQNPSIMILDEATSALDTESERLVQDALEKLMVGRTTFVIAHRLSTIVGADKIVVMEKGEIKEIGNHSELLMKNGIYTRLYNIQFNRDKELDREIAE |  |  |  |  |  | Lipid transporter ATP-binding/permease | Immune Modulation |
| GC_00000103 | MIFLFFYKNISIFTVFLVFSVWQMILYRIFKKLSYGKDNRYTPKFFNERNIVKFLIDSLSVVAGIVMAFMLKFSFGWQNYLKIEYIITYLGIFLVGYVYKNMSEKSWSYTNILDVLNILILNLGTAVIFTIFMYISSVYSFPDTTLVMVLILSVSMQLLGRYAFRLKRYYGRPHKNVKDIKRTLIYGAGEAGAILARESMTNPYFPYELVGFIDDDEKKIGSEMYNIKVLGNKDSLEMIIKNEKIEEVLLALPSIHSLDIRRIVERIQKLDGVKIKTVPTISEILENESLASQVRDVRIEDLLGRDEIVINDGSIRRLIEGKTIFVTGGAGSIGSELARQIAKFNPKQLITLDVNENDTYFLALELKRKYPNLDLVSEICNIREKDKLEFLFNKYRPNVVFHAAAHKHVPLMEHNPEEAIKNNIFGTKKVAECADKYGVERMVLISTDKAVNPTNLMGASKRACELVIEHMNKVSKNTKFMAVRFGNVLGSNGSVIPIFKKLISEGKNLTVTHKDITRYFMTIPEAAQLVIEAGAIGKGGEIFILDMGKPVKIYDLAMSMIKLSNANVGIDIVGLRPGEKLFEELLYDVNSAIKTDNKKIFITKIEDGTVDITKYFDKLEECIKNPNIEEIKKVMKQLVVSYKEVKYN |  |  |  |  |  | Type 8 capsular polysaccharide synthesis protein Cap8D | Immune Modulation |
| GC_00000104 | MIFVKDYNKTAIIYDDREYSYKEVILSAKSFSERINIKKEDRVIIFMENRPELLFSFLGIWNKQGTCVCLDGGFSGEELTYYIKDCEPKYIFTSKVNYEAAVKGLELAGMKNSVEILIVDDLPIDYTGDDLTIEIEDKYFVSLILYTSGTTGNPKGVMLMFDNILINMEGLDEYKMFRTTDRVLALLPMHHIFPLLGAGVVPLGKGSTIVFLKEVSSQAMVEALQKYKITFIIGVPKLWEMLHKKIMEKINGGKITKFIFKLAEKINNKNFSRKIFKKVHDGFGGNIRFFVSGGSKLDPQVSRDFLTLGIDVCEGYGLTETSPMISFTPTDEVVPGSAGKILTGVTVKIADDGEILAKGRNVMKGYYGRPDATAEVIDKDGWFHTGDLGEIKNGLLYVTGRKKEMIVLSNGKNINPIEIEQWIMANTDLIKEMAIMDYEDKLTAVIYPDFYKLHEEGITNITETFKKGVIDKYNKQAASYKKVLDVKIVQEELPKTKIGKIRRFMLKDVINKKEEKVQNIVEPSTEEYKNISAFLKSIKNKPIIPNAHLELDLGLDSLDTVELLSHIEGTFGVKIDEQTFVEHCTVEKLAEYVEKHSNEMMNDTQMDWKEILSKDTEGELPKSNGIGKIIKFILKPAFLFFVKVKKEGLENIEKNEPVIFAGNHQSFLDGFIVNQAVPNSVLDKTYYFADIKHFKKGYMKFMGENSNIIFVDINKNLVNSLQMLSKALRSGKNIVIFPEGTRTRDGKINNFKKFFAILSKELNIPIVPFVLDGAYEAYPPSSKYPKGGDVKVKFLEKIYPADMSYEEITEKVYNTIKKELK | K00655 | 119.57 | 140.7 | 8.80E-42 | 1-acyl-sn-glycerol-3-phosphate acyltransferase [EC:2.3.1.51] | Cereulide synthetase B, CesB | Exotoxin |
| GC_00000105 | MTLAGLSIRRPVATTMLMISMMFIGLIAMFSMKSEMLPNMNIPVVTIRTSWNGAVPEDVETQITKKIEEVLPNVEGIDKIESTSQYGMSTIVVKFDYGIDADDKVTDIQREVSRLVNDLPDDADTPVVKKVEAGVGNLTMVVSVSGASRMELNTALENYLKPRLESLTGIGEVNIFGTPDKQVQIQVNGDKLAAFGMSPMELYQMIGSSSQSIPLGTIDTGKKQIVARFMGETNYIDQIENIILRSNGNILRVKDIADVVLTTEDPTDVGYFNGKENIVLVIEKSSDGSTIDLNNAARKVLEDVKSVMPPGTEFNIMLDTSEDIVKSISSIGSSALQALVLATIVLLLFLKNIRATVLISIALPVSVIFTFAFLALNGTSLNLISLMGLSLGVGMLTDNSVVVIDNIYRHMTELKSPVMAAADDGTTEVTMSVIASSLTTMVVFIPVLFIPGIAREIFRDLSFSIIFSNLAALIVSLTLMPMVASRFLSNKQNITKEGKIFTAVKEKYLVVINWAVKHRVKTIIIPIVLFVIVMFGLGRFLKVQFMPKQDQGRYSVVAELGKGVDIERAEEIGRKIEEIVKANKNTQTYFSLIQNDTVAVNVEIGKKDTRDESVFEIIDEIRPSVEKIPGIRPNLSEDFQMSSPQRDVEFDIVGPNLDELKVIGKQVMAHMVKYPGAVDVTSTVDAGNEEARVVLNREKIRSYGINPVTVGQTVSYFVLGGDRGDTLTVKTGTEEIDVLIRLPKDKRKEVSDLSNLNIKIGEGQFVKLSDLATIEMAEGSPEINKTDRIYSVTVSANDGGVGLANIQAEMMKAFNEQNPPSSISYKWGGDSENLADASTQLGAALAISIFLIYALLASQFENFVFPIIIIGSIPLALIGVVIGLLVTNQPIDVMVMIGIIMLAGVVVNNAIVLIDFIKMTRERGSDRETAVIESCRTRLRPILMTTMTTVFGMLPLAMGMGEGSEIYRGMAITTMFGLSFSTLLTLVVIPIFYTLVEDMNNAIIRFVKKIFGKLLSKFPKKKEKTK |  |  |  |  |  | Acriflavine resistance protein B | Antimicrobial activity/Competitive advantage |
| GC_00000107 | MKYNRENTEKLLQDIFQKKILVKGVVSNMKGNYPYSKVNIKPLLIKNNFFFQFEQFQNNKAYHSNLNMEESIEKLKELLDNFKQFMIFTTEGDYQILKGKNDFTVKETKKTREITTLNHNKTKNYILEEGTPIPFLIKLGVMGEKGEVFKDSYNKFRQINKYLEFIDDTIKELKNKNLIGDTIKVVDFGCGKSYLTFALHYYLKNVKNMKFTIIGLDLKKDVMDYCNKVARELNCENLEFLTGNIKDFDKMKDADIIFSLHACNNATDYALLKWLELNAKAILAVPCCQHEFNQKMSSNKNSEFFNIENVIGKHGILLEKFTSVATDAFRAQALELCGFKTQVMEFIDMEHTPKNILIRGIKDKVKPETLEKKYIEYETFKKFLGIEPILDELLKPYFLIK |  |  |  |  |  |  |  |
| GC_00000108 | MFKQNTFSESDLYRGSLPFFLAEEKKGVIFLASSNKNIEDYYYTLKDFYNGEIIKIDDFEDENDEYKKNYALIDCLKNKNKYIILISLQGIMKKYVKEGERLFFEKGKEIGRKELEEKLIGSGYRKNYLVEERMEYSLRGDILDIFPISSEFPYRIEFFDEEVERITEFDLYTQKSIEEKENIYMYIDKNKNERFSFLEILKDFGKSAEKFYIENTEILRYKLEEQILKDRDNEEEYRKIFEDVASSFEPLETKRLDFDKIKKYEDLEVIKKESRKQKILILSEEKKRYDEIFADCKNIEIKKYPHYEGFYAGDTLVLTDREIKGIRVKKEVRIRGGVRYENISQIREGDYIIHENFGVGKYLGIEIIDGNDYLKIQYAGEDKLYVPTENLNRIEKYICDPGNVPEIYNLGRRGFKKKREKLENEMREFANELISIQARRKSALGFAFSKDTVWQEEFEEGFPYTETKDQLEAIRMVKRDMESDMVMDRIICGDVGFGKTEVAMRAAFKAVMDGKQVLIITPTTVLADQHYERFEERFKNYSINIALLSRIKTGKEQEEIIKKLAIGGVDIVIGTHRLLSDDISMGNLGLVIVDEEQKFGVKAKEKLKKMKENVDLLTLTATPIPRTLNLALLGIRDISVIKTAPPNRLPIENMLIDNNKDTIRDVIMKEIGREGQVFYIYNSVYGMEYKVQELAKILPAYIKIGYAHGRMAAKQIKEILHQFENGDIDVLVTTTIVENGIDIENANTIIIEGIEKLGLSQIYQLRGRVGRGKKKAYCYLINDSDRKYNKKTNLRKESIEKLEGLGGGFNLSLEDMNIRGAGEILGEKQHGALETFGYNLYMKLLQEEIERLKGEYKPKADIKINLCEPAYIPKEYIEEFEKITIYKRAVDMTEIEDIKNLFEEIEDRFGKAPQEVKNLFRFLEIKSLALKFGVSEIQKEEKGSYSLKFNKDKIIPEKIISMITTGKCRLKGKDGIEYFKDIMEFFKEYEEEEIEGIL | K03723 | 540.27 | 1038.4 | 0 | Transcription-repair coupling factor (superfamily II helicase) [EC:5.6.2.4] | Dot/Icm type IV secretion system effector LegC2/YlfB | T4SS Effector Delivery System |
| GC_00000109 | MKYDLVVIGGGPGGLAAAIEARNNGVEKILVIERDKELGGILQQCIHNGFGLHEFKEELTGPEYAERFIKKLYELNIEYKLDTMVLEVSPEKIIHAINTKDGYMMIEAKAVVLAMGCRERTRGAIAIPGERPSGIFTAGTAQRFINMEGYMVGKKVLILGSGDIGLIMARRMTLEGAEVKAVVELMPFSGGLARNIAQCLNDYDIPLYLSHTVVDIKGKERLEKVVIAKVDENRRPIPGTEIEYDCDTLLLSVGLIPENDISRKTGIEMDRRTNGPVVNEMMETSIPGIFACGNVVHVHDLVDFVSAESRRAGAAAAKYVKDEIKNGEYKTIKNGFGITYTVPQKFRVENVENNLEVFMRVNNIYKNMRLQVKDGDKVIVNLKKPHLAPGEMEKVIIPNKILQSITGNEIVIELAGGDE |  |  |  |  |  |  |  |
| GC_00000112 | MDILDLYGIEYEVIPGVSSFLAAAASVKKEFTLPDISQTVICTRLEGRTPVPERESLESLASHRASMAIFLSVQMIDEVVKKLLKYYSPETSIAVVQRATWEDEKIVMGTLENIAEKVKEAKINKTAQILVGDFLAEKPKYSKSKLYDKYFTHEYREGIKNKEEKI | K05936 | 217.6 | 234.4 | 1.90E-70 | Precorrin-4/cobalt-precorrin-4 C11-methyltransferase [EC:2.1.1.133 2.1.1.271] |  |  |
| GC_00000113 | MEKTDISLEIEKNEKENLNEKKEIEEKSIIIKEREEKIQPEKQEGFFASLKEKLFRTREGLFGKIKNIFSGRTVIDEELYEELEDLLIQSDIGMDMTIKIVNALEKEVKARGIKDPAEIYSVLKEVMSRFLIDGDNKLDIREGELNVILVVGVNGVGKTTTIGKIAKKLKNSGKKVIIGAADTFRAAAVEQLEEWGKRADVEVIKKEEGSDPGAVVYDTIQVGIDKKADVVIIDTAGRLHNKNNLMKELEKINNIIIKKLGHSRYESILVIDGTTGQNGLTQAKVFNEATKLTGFIVTKLDGTAKGGIVFSISEEIKKPIKYIGVGEKIDDLREFNTKDYIDAIFD | K03110 | 294.27 | 462.7 | 1.50E-139 | Fused signal recognition particle receptor | Flagellar biosynthesis regulator FlhF | Motility |
| GC_00000114 | MSKKDVIELEGTILEALPNAMFKVSLENGHTILGHISGKMRMNYIKILPGDKVTVQISPYDLSRGRIVYRKK | K02518 | 42.37 | 145.7 | 1.60E-43 | Translation initiation factor IF-1 |  |  |
| GC_00000115 | MPKMKTHRGTAKRVKVTGTGKYVVKHSGTSHILTKKTRKRKNRLKKDFVVLDIHKKDMVALLPYGVGR | K02916 | 18.63 | 43.7 | 3.50E-12 | large subunit ribosomal protein L35 |  |  |
| GC_00000116 | MPTLNQLVKKGRQTLEEKGKSPALQGNPQRRGVCVRVYTTTPKKPNSALRKVARVKLTNGLEVTCYIPGEGHNLQEHSIALIRGGRTKDLPGVRYKIIRGALDTAGVNNRKQSRSKYGVKKA | K02950 | 77.4 | 240.7 | 2.50E-72 | small subunit ribosomal protein S12 |  |  |
| GC_00000117 | MGQKVDPRGLRLGITRSWDSNWYADKKEYAKYFHEDVKIREFVKKTYFHAGISKVNIERTSPSHVVVIVYAAKAGIVIGRKGAEIEELRTKLEALTGKKVLVKVQEVKNFNKDAVLVAENIAGGIERRVAYKRAVNQAVMRAMKSGAKGIKVMVSGRLNGAEIARSEWVVEGKVPLHTLRADIDYATATAHTTYGALGIKVWIFNGEVLPTKKEGGEA | K02982 | 109.4 | 308 | 7.80E-93 | small subunit ribosomal protein S3 |  |  |
| GC_00000118 | MDILVAKTIVLAASAVGAGCAMIAGLGPGIGEGYAAGKAVEAVARQPEAKSNIISTMILGQAVSESTGIYSLVIAMILLYANPFMSILEKAAELVK | K02110 | 68.87 | 114.4 | 4.40E-34 | F-type H+-transporting ATPase subunit c |  |  |
| GC_00000119 | MAKEVIKIIKLQLPAGKANPAPPVGPALGQHGVNIMEFCKAFNAKTQDKSGWIIPVEISVYNDRSFTFIMKTPPASDLLKKAAGVQSGAGNSKKQVAGTITKEKLKEIAETKMPDLNAGSLEAAMSIIAGSARSMGIKIED | K02867 | 62.8 | 246.2 | 8.90E-74 | large subunit ribosomal protein L11 |  |  |
| GC_00000120 | MARSLRKGPFCDHHLMKKVEEAVAANNLKAVIKTWSRRSTIFPNFIGLTFGVYNGKKHIPVYVTEQMVGHKLGEFAPTRTYHGHTKTGKK | K02965 | 104.13 | 163.6 | 7.40E-49 | small subunit ribosomal protein S19 |  |  |
| GC_00000121 | MANKLRIYLKAYDHMLLDQSAKKIAEVAEKSGAEIAGPMPLPTKIKKYTVLRSVHVNKDSREQFEMRIHRRMVEIKNSTQKTISSLTAVNLPAGVGIEIKQA | K02946 | 57.9 | 113.5 | 1.60E-33 | large subunit ribosomal protein L21 |  |  |
| GC_00000122 | MYAVIKTGGKQYKVAEGDVLRVEKLNAEVNQTVELTEVLLVANGEDVKVGTPVVEGAKVVVEVLSQGKGAKVVNFKYKPKTGNHRKKGHRQLFTEIKVTAINA | K02888 | 28.77 | 136.9 | 1.20E-40 | large subunit ribosomal protein L21 |  |  |
| GC_00000123 | MAKKKVAKVKKKLKNIPSGVAHIHSTFNNTIIAITDTEGKVVSWRSGGTSGFKGTKKGTPFAAQIAAEQAANIAMENGMKKVEVKVKGPGSGREACVRSLQAAGLEVTKITDVTPVPHNGCRPPKRRRV | K02948 | 102.07 | 180.1 | 8.20E-54 | small subunit ribosomal protein S11 |  |  |
| GC_00000124 | MLFTLNIQLFAHKKGQGSVKNGRDSNPKYLGVKKYDGEAVKAGNIIVRQRGNKFHAGVNMGEGKDHTLFALIDGYVKFERLGKDKKQVSVYAEK | K02899 | 36.03 | 147.8 | 4.80E-44 | large subunit ribosomal protein L27 |  |  |
| GC_00000125 | MAKPKIKFVPASLHVKTGDTVYVISGKDKGKTGKVLKVFPKKGKIVVEDVNVVTKNMKPSQTNPQGGVVTKPAPIFSSKVMLFDAKAGKPTRVGYKVVDGKKVRYSKVSGEVL | K02895 | 46.13 | 133.5 | 1.50E-39 | large subunit ribosomal protein L24 |  |  |
| GC_00000126 | MIIKQAEFVKSAVYEKDYPEELSNIEFAFVGRSNVGKSSLINSITRRGKLARISKTPGRTQLINYFIINNEFFLVDLPGYGFAKVPKAMKAEWGQTMERYLASKRKKLVFVLLDIRRVPTAEDMDMLHWLDHFDIPFKIIFTKMDKVSNNEKFKCLKAIRTKLEFHNEDVFFHSSLKDTGRDEILDYIEEILNEEKIQQEGN | K03978 | 92.67 | 253 | 4.90E-76 | GTP-binding protein |  |  |
| GC_00000127 | MKTKRELIAEFGKNEKDTGSTEVQIAILTEEINHLTEHLKVHKKDFHSRLGLLKKVGKRKRLLNYLAGRDIEEYRNLIARLGIRK | K02956 | 29.73 | 123.3 | 2.00E-36 | small subunit ribosomal protein S15 |  |  |
| GC_00000128 | MNQCRGTGRRKTSVARVRLIPGEKGIVINGKEMSQYFGGREILSKIVEQPLVLTETLDKYQVIVNVYGGGNAGQAGAIRHGISRALLEADETLRGALKEAGFLTRDSRMVERKKYGKKKSRRSPQFSKR | K02996 | 104.4 | 197 | 6.30E-59 | small subunit ribosomal protein S9 |  |  |
| GC_00000129 | MSRRRAAVKRDVLPDSRYSDKVVTKTINAFMVDGKKSLAESIFYSAMDLIKEKTGKEGYDVFKQALENIKPQVEVRSRRIGGATYQVPTEVRLERQQTLALRWLVTYTRERKEYGMVEKLAAELIAASNNEGATVKKKEDTYRMAEANRAFAHYKY | K02992 | 105.77 | 206.9 | 5.00E-62 | small subunit ribosomal protein S7 |  |  |
| GC_00000130 | MVQQQTILNVADNSGAKKLMIIRVLGGTKKRFGKIGDIVVCSVKEAIPGGNVKKGDVVKAVIVRTRKELRREDGSYIKFDDNAGVIINNNNEPKATRIFGPVARELRAKNFMKIVSLAPEVI | K02874 | 99.6 | 208.5 | 1.40E-62 | large subunit ribosomal protein L14 |  |  |
| GC_00000131 | MLDKIKEIVVEQLGVDADQVTPEANFVDDLGADSLDTVELIMAFEEEFDIEIPDTDAEKIKTVQDVMDYIEENK | K02078 | 38.97 | 107.9 | 8.40E-32 | acyl carrier protein | acyl carrier protein | Immune Modulation |
| GC_00000132 | MAVPKKKTSKAKKNMRRSHHGLAASTLITCEKCGATKRPHRVCLECGDYNGKQVLKTADAE | K02911 | 21.07 | 81.9 | 7.50E-24 | large subunit ribosomal protein L32 |  |  |
| GC_00000133 | MEDVLVPEGYKNKLSLLETEVSIKKVKDFFERALSYELNLTRVSAPLFVREDSGLNDNLSGYEKPVSFTTKLEDGNYHIVHSLAKWKRMALYKYQFPMHTGLYTDMNAIRREEDLSNIHSLYVDQWDWELIIKKEERTMKTLEHIVRNIFRAFKKTEEYILREYPQLSKKLPEDITFITSQELEDRYPDLTPKEREHAIAKEYKAVFISQIGKTLKSGKKHDGRAPDYDDWELNGDILFWYPPLNRSVELSSMGIRVSEESLEKQLKLANAEERKEFPFHKALLEGKLPYTIGGGIGQSRICLFFLDKVHIGEVQASVWPKEMVEAYEKHGVHFL | K01914 | 234.7 | 526 | 4.10E-159 | aspartate--ammonia ligase [EC:6.3.1.1] |  |  |
| GC_00000134 | MEGAENLKRVPSSMEAEKSVLGGIFLKPDCFGDIIEIISPNDFYKVGHKYIFEAMIECYNTGENIDPIVVMNKLRKMNKFDEIGGESMFYDVIGGVVTAAHIDVHAKIIKEKSILRRLGDVGTKIVEMSYDGYEDVDTILDKAEGLIFKISENKESKDVISIKDAMTEEFMRLEEVFNNKGAATGISSGFVNFDEKTSGFNPSDLIILAARPSMGKTAFALNLALNAAMKSDKSVLIFSLEMSSSQLLQRLLAVQSGIGLQKIRNGFLEEEEWGRLGIASGQLAESHINIADVPNVNVLEIRAMARRLKAMNKLDMIVIDYLQLIKGTGRGDNRQQEISDISRSLKGIARELDIPIIALSQLSRAPEQRADRRPMLSDLRESGAIEQDADMVVFLYRDDYYNEESEEKGITEVIIGKQRNGPVGTVKLRFFHEITKFADYTTRVE | K02314 | 105.5 | 586.4 | 9.50E-177 | replicative DNA helicase [EC:5.6.2.3] |  |  |
| GC_00000135 | MAVVTMKQLLESGVHFGHQAKRWNPKMAKYIFTERNGIHVIDLHKSLKKIEEAYEVVREIAAQGGTVLFVGTKKQAQEAMKDQAERSGMYYVNNRWLGGMLTNFETIKGRIARLKELDRMDKDGTLDTAYTKKEAANLRKELEKLSKNLAGIQDMPRIPDALFVVDVKKEALAVAEADHLGIPVIAMIDTNVDPDLITYPIPANDDAIRSVKLMSTLVANAIIEGKQGAEEKVVDETTEVTEEVAENGAAE | K02967 | 171.6 | 394.4 | 7.90E-119 | small subunit ribosomal protein S2 |  |  |
| GC_00000136 | MKKGIHPDYKVVTVECTCGEKFETRSTYAKGDLKVAVCSKCHPFYTGKAKFLDTAGRVDKFNKKYNIK | K02909 | 19.67 | 125.2 | 2.80E-37 | large subunit ribosomal protein L31 |  |  |
| GC_00000137 | MKDINLLSKTANELRKNVVKMICNAKSGHPGGSLSAADIVTALYFSEMNVDPKNPRMEDRDRFVLCKGHAAPVLYAALAEKGYFEKDLLMTLRKYGSPLQGHPDMKKLPGVDASTGSLGQGLSIANGMALAGKLDKKDYRVYALLGDGEQQEGQVWEAAMTAAHYGLDNLCAFLDSNGLQIDGEVEKVMNVAPLDEKWRAFGWHVIEINGHDFKEIFDALDEARTVKGKPTMIIAKTVKGKGVSFMENLASWHGSAPNAEQTEKALEELSKND | K06615 | 183.87 | 376.5 | 3.50E-113 | transketolase [EC:2.2.1.1] |  |  |
| GC_00000138 | MDRIVTDKELANEIDSQKSLRPKRFSEYIGQTNLKEKMGIFIEAAKRRGGCIDHTLLYGPPGLGKTTLAGVIANEMNVNLKITSGPVLDKAGDLAAILTSLEENDILFIDEIHRLNTSVEEILYPAMEDGELDIIIGKGPAARSIRIELPHFTLIGATTRAGLLSSPLRDRFGVSHRMEYYTEEELVQILIRGAKVLGVEIEKEGAVEIARRSRGTPRIANRFLKRVRDYSEIKGNGIITKRIAEEALRLLGVDDEGLDELDRNIIFSIMKNYGGGPVGIETLSLLLGEDKRTIEEVYEPYLVKTGYIKRTPRGRMVTNKAKEHFEIKE | K03551 | 183.87 | 570.3 | 4.80E-172 | holliday junction DNA helicase RuvB [EC:5.6.2.4] | ESX-1 type VII secretion system AAA+ ATPase EccA1 | Effector Delivery System |
| GC_00000139 | MKFISWNVNGIRACLTKGFMDFFNNEDADIFCLQETKVQAGQVELDLKGYHQYWNYAEKKGYSGTAVFTKQEPLSVRYGLGIEEHDKEGRVITLEFPDFYFITVYTPNSKTELERLDYRMVWEDEFRKYMKDLEKEKPVVVCGDLNVAHKEIDLKNPKTNRKNAGFTDEERNKFTELMDAGFIDTFRYFYPEQEGIYSWWSYRFKAREKNAGWRIDYFLTSASMKDRLVSAKIHTEILGSDHCPVELVIK | K01142 | 224.3 | 388.2 | 7.50E-117 | exodeoxyribonuclease III [EC:3.1.11.2] | catabolite repression control protein | Adherence |
| GC_00000140 | MFKKVNRQAVRDRKHLSIRTKISGTAERPRLSIYRSNTNMFAQLIDDVNGVTLVSASTVDKELKAQIANGGNVEAAKLVGKLLAERAVAKEIKDVVFDRSGYIYTGRVAALAESARENGLNF | K02881 | 42.77 | 150.7 | 6.70E-45 | large subunit ribosomal protein L18 |  |  |
| GC_00000141 | MLDKIVIKGAREHNLKNIDVEIPKNKFVVITGVSGSGKSSLAFDTIYSEGQRRYVESLSAYARQFIGQMKKPEVDSIEGLSPAISIEQKTTNKNPRSTVGTVTEIYDYMRLLFAHIGTAHCPVCGTVVDKKSVEEIVENIIEKFEEKAKMTILAPVVREKKGTHKNLFLNLVKKGFVRARVNGEILYLEDEIELDKNKKHNIEVVIDRLVLKKENSEFVSRLTQSIEAGMEVSGGKIIINIDGEDFNYSENYACPNHEDISIPELTPRLFSFNAPYGACPECKGIGQKLEVDENKLIIDKTLSIEDGGLYVPGASSRKGWTWEMFLSMCRDFDIDYTIPVEDIPKEQMDIILHGTDKKFRFDFESKEFSFHGMREFRGIVKNMEKRYYETFSDSAKEEIENRFMIEKECKVCHGKRLKDEVLAVTINGKNIIDLCLMSVKDSLEFFNNLILTEKQEKIAKEILKEIRERLSFMINVGLDYLSLFRPTKTLSGGESQRIRLATQIGSGLTGVLYVLDEPSIGLHQRDNDKLLATLNRLRDLGNTLIVVEHDEDTMNQADYIFDLGPGAGEFGGNLVAKGTPAQIKRNKNSLTGKYLRGEIKIEVPKERRTSDKFIELTGAKGNNLKNLSVKIPVGVLTVVTGVSGSGKSTLINQTLFPVLFNELNKGKLYPLEYKSISGLEHIEKVIDIDQTPIGRTPRSNPATYTKIFDEIRDIFAQTKDAKLKGFSKGRFSFNVKGGRCEACQGAGIVKIEMNFLPDVYVECDVCRGKRYNKETLEVYYKGKTISDVLNMSIDEAYEFFNVVPSLEKKIKVLKDVGLGYIKLGQPATTLSGGEAQRIKLAAELSKSSKGKTVYILDEPTTGLHFEDVKKLMEVLQRLVDRGNSVIIIEHNLDVIKTADYIIDIGPEGGDRGGTVVACGTPEEIAEVKKSYTGKYLKKYLR | K03701 | 354.1 | 1461.3 | 0 | excinuclease ABC subunit A | ATP-binding protein AatC | Others |
| GC_00000142 | MENTNYLLVMDNISKEFPGVKALDGVNLKVRPYSVHALMGENGAGKSTLMKCLFGIYKKDGGKITFDGKEINFTSAKDALENGVSMVHQELNQVTQRNVLDNIWLGRFPMKGMFVDEKKMYDDTVKIFKDLDIEVDPRMKVADLSVSQRQMIEIAKAVSYNSKIIVMDEPTSSLTENEVQHLFRIINKLRDKGCGIVYISHKMEEIKEICDDITIMRDGKWVATDSVKDLTTDQIINMMVGRDLTNRFPPKDNEVKEVILKVENLSALHQPSLKEASFELHKGEILGVAGLVGSKRTDIVETIFGMRERATGKIILNGKEVKNRTPGEAIRNGFALVTEERRATGIFSMLDIKVNSIVSNIDHYKNKLLGLLDNKKMVEDTRWVIDSMRVKTPSQSTSIGSLSGGNQQKVILGRWLLTEPDVLMLDEPTRGIDVGAKYEIYQLMIELAKKDKGIIMISSEMPELLGVTDRILVMSNGRVAGIVNTKETTQEEILALSAKYL | K10542 | 728.27 | 864.8 | 1.90E-261 | methyl-galactoside transport system ATP-binding protein [EC:7.5.2.11] | ATP-binding component of hemin transport system | Nutritional/Metabolic Factor |
| GC_00000143 | MARIAGVDVPRNKRVEISLTYIYGIGKKTAQDILTQAGVNFDTRVKDLTEEELNKIRGIIDGLKVEGDLRKEVRLAIKRLLDIRCYRGLRHKMNLPVRGQKSKTNARTRKGPKKMVKK | K02952 | 114.77 | 185.8 | 1.30E-55 | Small subunit ribosomal protein S13 |  |  |
| GC_00000144 | MDIKEILERNPIIPAVKNENYLQEAMECGSEIVFVIMSNLINIQDIVEKLKSAEKIVFVHVDMIEGLSSSNYGVDYLMAHTKADGIITTKHNIVAFAKKNNIKVIQRFFILDSFSFKNTITHIRENKPDAVEILPGVMPKIIKRTCNLVNIPVITGGLIDEKEDIINALNSGAEGISTTDITLWDF | K02443 | 91.07 | 241.1 | 8.60E-73 | glycerol uptake operon antiterminator |  |  |
| GC_00000145 | MANQYNANGKKEGLWVKYYENGAVKEEKNYVNGVREGEYKCYYPNGNIETKKHYKNGNLHGVYETFLSNGARDTVYHLINGKS |  |  |  |  |  |  |  |
| GC_00000146 | MSYILGIGLAVIGLCIVFAMLYKKSVIDKKIQELRDTEDEKLKAKIKAKEIIKNAESEALILKKDIELKAKETVYQMKEEAEKEIKIAKNEVLQKELRLTRKEESVDNKLEKIEAKSIELEKTRGELEKKNEEVQALIEKQEVELERISELSKSEAKEMLISKLRDSLTHETAVAIREFEAKLKETKEDISKRILSTVIGKAASEYVVDSTVSVVNLPNDEMKGRIIGREGRNIRTIESLTGVDIIIDDTPEAVVLSSYDGVKREVARRAIEKLISDGRIHPGKIEELVNKSRKEIEKDILEAGEQALIEVGIQGMHPEIVKTLGRLKYRTSYGQNVLVHSIEVARLAANLAAELGADAELAKRGGLLHDIGKVLDHEIESSHALIGGEFLKKFGEHEDVINAVMAHHNEVEYSTVESILVQAADAVSASRPGARMETLSSYLKRLASLEEIAKSFKGVESSYAIQAGREIRIIINPDVVSDDEAAVMAREVAKKIEETMQYPGQIKVTILRETRATEYAK | K18682 | 117 | 692.3 | 5.20E-209 | ribonucrease Y [EC:3.1.-.-] | type VI secretion system ATPase TssH | Effector Delivery System |
| GC_00000147 | MKPQIKEIIVVEGRDDITAVKAAVDAEIVAVNGFSVGKNIEKVKAAYERKGIVVLTDPDFAGLKIRDYIQKRFPKAKHAYINRAEGTKNGDIGVENASPEAIIKALEKARCELGEIREVFKPEILFEYKLTGHSDSKNLREKLGKKLGIGYSNGKQLLSKLNRYDISMEEFIKAMEEIRADENEK | K05985 | 85.7 | 227.4 | 2.30E-68 | ribonuclease M5 [EC:3.1.26.8] |  |  |
| GC_00000148 | MLMPKRTKHRKMFRGRMKGSAQRGNTVAFGDYGLQALEPAWITNRQIESCRIGINRTFKREGKTFIRIFPDKPITSRPAGVRMGKGKGAVEGWVCVVKPGRIMFEVSQVSEELAMAALRKASMKLPIRCKIVKRENGGDK | K02878 | 80.7 | 213.7 | 5.10E-64 | large subunit ribosomal protein L16 |  |  |
| GC_00000149 | MEEKMLYIDSKENNSFKKIKKYRTKKYRDKDKRFLAEGTKFLDFDTVPEYIIIDEEYSKNREIMKKVEKFEKSEKLVLSQGLFSQLSSQENSQGIILVYKYEEKDIENAGDNVIVLDRVGDPGNLGTIIRTVDAAGFKDIILTKGSVDCYNEKVIRSTMGSIFNINLYYAEEEKLLNFLKEKNYKIISTVLSDDCIPYTEMKPAEKNAVIFGNEGSGIGENIIKNSHEKVIIPIYGTAESLNVAMACGIIIYKLRELYK | K03437 | 205.8 | 232.6 | 7.20E-70 | RNA methyltransferase, TrmH family |  |  |
| GC_00000150 | MICLNLKELGVQNLIVKASSKKHRKLLEKIGASQVIYPEEYVGKRTALVAMEPNMIEHLRFSQDFLIVEVKAPTIFWDKTLMESEIRKRYNSNVVGIKKHNGKFVPNPNPNEKIEENDILIVVTDSKTANALNGLVKKEFKEN |  |  |  |  |  |  |  |
| GC_00000151 | MDLHYLKIFYEVAREKSFTKAANKLYINQSAVSIQVKKFEELLNAKLFDRSSKKIRLTYTGEALYRMAEDIFNKVQRAEKEINRIIKLGKAKIVIGATSIIGDPLLPRLMEEFSAKHGEIEYEIQMGNKAWLLKSLKEGEIDIALLDEEHIVDSNLDVMTVEKVPYVLITGKKNITLENVADYPLITRSNVPNNAKAINLLEEKYKISFDNRITVLGSLEVIKGMVRNEIANVILPYYAVHKEIKSGEFKVVEQLNEIKDGYQVVVTKDKSTLTPIIKFLNFMSEYKING |  |  |  |  |  | LysR family transcriptional regulator | Nutritional/Metabolic Factor |
| GC_00000152 | MSRIGKKPIVVPAGVTVTVDGHKVTVKGPKGTLTKEFNKNLTIALEDGHVVVTRPDDSIEMRALHGTTRALIHNMVVGVSEGFKKVLNLVGVGYRAAVKGKGLELALGYSHPVIIDEIPNITFAVEKNTTIIVEGIEKDVVGQVAAVIRSKRAPEPYKGKGVKYADEVIRRKEGKKS | K02933 | 112.9 | 271.5 | 1.10E-81 | large subunit ribosomal protein L6 |  |  |
| GC_00000153 | MAKHRGKKYLEVAKLVDTTKLYEVKEALELIKKTRTAKFLETIEVALRLGVDPRHASQQIRGTVVLPHGTGKTVKILAITQGENINKALEAGADYAGAEEYIEKIQQGWLDFDIVIATPDMMPKLGRLGKILGTKGLMPNPKSGTVTPNIAAAVSEFKKGKLAFRVDKLGSIHVPIGKADFDDEKVYENFKAFMAEIIRLKPADAKGQYLRTVAVSLTMGPGVKIDPLLVAKEIGA | K02863 | 92.27 | 319.6 | 4.20E-96 | large subunit ribosomal protein L1 |  |  |
| GC_00000155 | MKDNRVCSLLGIKYPIIQGAMAWIADGNLAGHVSKEGGLGIIAGGGMPIDILRNEIKKAKAITENPFGVNLMLMMPNIEEQIDVCIEEGVKVVTTGAGNPGVYMEKLKAAGIKVIPVVASVALAKRMEKIGADAVVAEGMEAGGHIGEITTMALVPQIAEAVSIPVIAAGGIGGGKQFLAAFALGAEGVQVGTKFLVADECNVHDNYKEAIIKAKDRSTVATGNYTGHPVRVINNKLAKEMLEMEKHGASVEELEKLGTGKLRLAAVEGDVKEGSVMSGQVASMVTKRESVKDIIEGLMRDLETEKENLFKYFGK | K02371 | 361.6 | 433.7 | 4.60E-131 | enoyl-[acyl-carrier protein] reductase II [EC:1.3.1.9] |  |  |
| GC_00000156 | MFLTDPIADMLTRIRNANAVMHEKVDVPYSNLKLTIAKILKDEGYIANYKVITDGNVKNIRVYLKYAGKERVIKGLKRISKPGRRVYAPVDELPRVLSGLGIAIVSTSKGIVTDRVARQENVGGEILAFVW | K02994 | 90.4 | 205.9 | 4.40E-62 | small subunit ribosomal protein S8 |  |  |
| GC_00000157 | MNYKDYITSIEGFPKEGITFRDITTFIGHGEAFRASIKEFSEFAKEKGAEVIVGPESRGFIFGAPVACELGIGFVPVRKPGKLPREVVSCSYELEYGTNTIEIHKDAIQKGQKVVIVDDLLATGGTTEAAVKLIESLGGEVVGIAFLIELADEFDGRGKLKNYPVLSLIKY | K00759 | 102.9 | 254.1 | 2.00E-76 | adenine phosphoribosyltransferase [EC:2.4.2.7] |  |  |
| GC_00000158 | MKNIKIAPSILSADFSKLGEEIIAIDKAGADWVHIDVMDGIFVPNITFGPAVMKSVRDKTNLVFDVHLMITQPERYIEDFVKAGADMITIHVESTLHPHRVIQQIKSYGVKAGIVLNPGTPAESVKYLINEVDMVLVMSVNPGFGGQSFIESAVEKIKELRAMNPTVDIEVDGGITHETIGRCIEAGANIFVAGSYVFKGNYEERIANLKKEG | K01783 | 254.97 | 353.3 | 2.00E-106 | ribulose-phosphate 3-epimerase [EC:5.1.3.1] | ribulose-phosphate 3-epimerase | Immune Modulation |
| GC_00000159 | MAIKKMRPTSDGVRHMSRLVVPELSKVRPEKSLTVPLKSAYGRDNYGHRTNVNRQKGHKRLYRIIDFKRNKLDVPARVVTIEYDPNRTANIALLHYADGAKAYILAPKGLKVGDVVMNGSNAEIKVGNALKLKEMPVGTQIHNIELQRGKGGQLVRSAGTAARLVAKEGTYCHVELPSGELRLIHGECTATIGEVGNAEHSLVSIGKAGRNRLMGKRPHVRGSAMNPCDHPHGGGEGKAPVGRKSPLTPWGKPALGVKTRGRKTSDKFIVRRRNDK | K02886 | 143.4 | 304 | 1.50E-91 | large subunit ribosomal protein L2 |  |  |
| GC_00000160 | MLQKFKRNFSIIAHIDHGKSTIADRLLEATHTVAERDMKAQLLDSMELEREKGITIKAQAVTLYYDAKDGNRYELNLIDTPGHVDFIYEVSRSLSACEGALLVVDAAQGVEAQTLANVYLALENDLDLIPVINKIDLPAAEPERVKEEIENVIGLPADDAVLTSAKINLGIEELLEAIVARIPAPNYDEEAPLRALIFDSKFDDYRGVITYVKVEDGSIKKGDKIKIWSTGREAEILECGIFSPNMKATGELTSGSVGYIITGLKTIKDSRVGDTITHVGRPCDEPLQGFRPAQSMVFAGIYPISTDDYGALRESLEKLQLNDASLNFVPETSLALGFGFRCGFLGLLHMEIIVERLRREYNIDLLSTAPSVEYRITMEGKPTYVIDNPCDFPEAGKAKFIIEEPYIKGNIIVPKDYVGGVMELCQEKRGTYIGMNYIDDNRTMLTYELPLAEIVLDFYDKLKSRTRGYASFEYELIDYRPGELIKVDILVSGTVVDAFSFISHKDNAVSKGRAICEKLKEVIPRQQFEIPIQAALGAKIIARETIKAYRKNVIAKCYGGDITRKKKLLEKQKAGKKRMKQIGNVEIPQEAFVSVLKLNDN | K03596 | 934.7 | 1062.1 | 0 | GTP-binding protein LepA | elongation factor Tu | Adherence |
| GC_00000161 | MRVLEGNFSGKGLKVGIVAGRFNEFITSKLIGGAVDALRRHEVNDDDIDLAWVPGAFEIPLVVKKMAESGKYDAVIALGAVIKGATPHFDYVCAEVSKGVAQIGLQTGIPVMFGVLTTNNIEEAIERAGTKAGNKGFDVANGAIEMCNLLKGM | K00794 | 47.97 | 240.8 | 2.10E-72 | 6,7-dimethyl-8-ribityllumazine synthase [EC:2.5.1.78] |  |  |
| GC_00000162 | MNCPFCNNSDTRVIDSRLFINGNSIKRRRECTACKKRFTTYEKVEERAVYVVKKDQSREKFDKEKLMRGLSIATIKRNISRDTLEEFVLEIERGLQNSLDSEISTKDLGEIVMKKLKELDEVAYVRFASVYMEFNDIKSFIEIVENIEKDKK | K07738 | 42.97 | 203.8 | 3.20E-61 | transcriptional repressor NrdR |  |  |
| GC_00000163 | MKREDYINWDEYFMGVALLSAKRSKDPNTQVGACIVTEDKRIVGLGYNGLPRGCSDDEFPWEREGEFLNTKYPFVCHAELNAILNSTKSLKDCIIYVALFPCHECSKAIIQSGIKEIVFLSDKYSGTDSDLASKRMLDAAGVTYRKLKPNIDKLILSFDEKDY | K01493 | 78.47 | 276.8 | 4.10E-83 | dCMP deaminase [EC:3.5.4.12] |  |  |
| GC_00000164 | MAKKIVTDLNVNDKKVLMRVDFNVPMKDGKITNDNRIVAALPTIKYVLENGGKVIAFSHLGKVKTEEDLKTKSIRPAAERLAELLGQPVKFVPVTRGAELEAAVNELKSGEIMMFENTRFEDLDGKKESKNDPELGKYWASLGDLFVNDAFGTAHRAHASNIGIAANIGEGKAAAGFLMEKEIKFIGDAVDSPVRPLVAILGGAKVSDKIGVIENLLVKADKVLVGGAMMFTFLRALGKSTGTSLVEEDKIELAKALLEKANGKLVLPIDTVVAKEFNNDAAHTTVSVDDIPADQMGLDIGAATIELFSKEIAAAKTVVWNGPMGVFEMPNFAKGTIGVCEAIANLQGATTIIGGGDSAAAAMQLGYADKFTHISTGGGASLEYLEGKALPGVTSLSDK | K00927 | 254.9 | 609.4 | 1.20E-183 | phosphoglycerate kinase [EC:2.7.2.3] |  |  |
| GC_00000165 | MVEKKYIAPNAITAANMLLGYLSITSSIRGNIEYAIWFIFLAMVCDGLDGKTARKLDAFSEFGKEFDSFSDAISFGIAPSILVYSILSMYPKFVSIVIPVAFIYALCGVMRLVKFNVITTASEEKDDFSGMPIPNGAASVISYLLICQALEKHFDYQFFNVEVFIAITIIAAILMVSTITFLTPDKVFRIPKKFMMPFVILVLVTLKYSMFIISFYYIIYNLFQYHLYRKELKHSSENIEEK | K17103 | 127.1 | 207.2 | 5.10E-62 | CDP-diacylglycerol---serine O-phosphatidyltransferase [EC:2.7.8.8] |  |  |
| GC_00000166 | MQKLPVTYELVKKTGNARAGVITTPHGKIETPVFMPVGTQGTVKGMTKEELIELGSEIILGNTYHLHLRPGDDLVARFGGLHKFSAWEKPILTDSGGFQVFSLGHLRKITEEGVAFSSHLDGSKRFLSPEKSIEIQNNLGSDIVMLFDECPPGMSSKEYLIPSIERTARWAKRCIDAHKRPDEQGLFAIVQGGIYEELREKSLESLMEMDEAFSGYAMGGLAVGEPREDMYRILENVTPKFPENKPRYLMGVGEPLDMLEAVESGIDMMDCVQPTRIGRHGTVFTKYGRLVIKNASYAEDDRPLDEGCDCYVCRNYSRGYIRHLLKAGEILGQRLTTYHNLYFLVHLMKNARKAILEDRFPEFKEEFIKNYKMGNSSEWIKPKKIK | K00773 | 534.57 | 673.7 | 4.30E-203 | queuine tRNA-ribosyltransferase [EC:2.4.2.29] |  |  |
| GC_00000167 | MTFLGFRIENDLKEEIESNAENELSFVETVGEFIEKIKEKNHDCVLIEEKNLPSETLINLIKKVNEFQQKTVVIVLGQSSNLKVVAGSVKAGAYDYLLKPLATSEILRVCGKAVRDYKLLAERVERNKNIGDKLIGQTKEIVQVYKKIGKVAAGRMPVLVTGEKGTGKKSVARAIHQFGDSSKKPFISINCLSFPHSLLERRLFGYEKGAFEGAIFSQAGELEKANGGTLHLGNVEALSLDVQSKLLYFLQEGEFFRLGGADPIKTDMRIIATTSADLTKEVKEGRFIEELFDTLKVLEIVIPPLRERKDDIPFIIDNYLRECNRELNKSVKGVSKPAMKKILRFDWPGNVNELKNAIKSAVAMCRGTSIVIEDLPSDVAGVRASRRKEAFQTWMLNEWIEDELKNYKKSIHKGAYFSYIVTTVEKELIKQVLERTSGKKIETAEILGITRNTLRTKMTNYGLE |  |  |  |  |  | two-component response regulator PilR | Adherence |
| GC_00000168 | MRNDRKVREGIVVSDKMEKTIVVSIATMDLHPIYKKRVKKTTKFKAHDENNVAQVGDRVRIMETRPLSRDKRWRLVEVLERAK | K02961 | 83.73 | 132.8 | 2.00E-39 | small subunit ribosomal protein S17 |  |  |
| GC_00000169 | MKITDIRLRAVKSENEVKLKAYADVTFDGSFVVHGLKIIDGQKGMFVAMPSRKMPDGEYKDIAHPITPELRKEITDTVIAKYNEVMESEGAIPEAVEE | K06412 | 31.63 | 139.8 | 7.20E-42 | stage V sporulation protein G |  |  |
| GC_00000170 | MISTSNLSMRFPDKKLFEDVNIKFTPGNCYGLIGANGAGKSTFVKILSGVITPTEGEVILDKNKRMAVLSQNHFAYEDVKVLDVVLMGHKKLWDIMVEKNAIYAKEDFTEEDGMRAADLEGEFAELNGWEAESEASTLLTGLGLKAKYHEMLMKELNEPQKIKVLLAQCLFGNPDVLLLDEPTNGLDIKAVAWLENFLMGLEDTTVIVVSHDRHFLNKVCTHIADIDYGKIKMFVGNYDFWYESSQLMLSLIANKNKKLEQKRQELQEFIARFSANAAKSKQATSRKKQLEKLQLEDMQVSNRKYPFIEFKPDREAGNNMLKVENLTKSINGVKIIDNLSFTINTKDKVVFLSESDIVKTTLLSLLAGDLEPDSGTITWGVTVTHAYMPKDNSAFFDGVDLDLVDWLRPYSPDQHDSYVRGFLGRMLFSGEESLKKAKVLSGGEKVRCMLSRMMMSGANALLFDNPTDHLDLESITSLNKALIKFPGTILFAGHDHEFIQTVANRIIEILPDGKLIDKLMTYDDYIQMKIDEEKDND |  |  |  |  |  | ferric siderophore ABC transporter, ATP-binding protein BauE | Nutritional/Metabolic Factor |
| GC_00000171 | MDILELKREFQTYKEKISGIKQTVKLEEREKTVEELEKKTTEDGFWNDKKESQSVIKKINENKDLINEYKQIENMYHDEEVLIEFVDMGEEDFVAELEEKHKKLAHEIDTLDVKLLLDGKYDGNNAIITIHSGAGGTEACDWVDMLYRMYSRWFSEKGYKVSQLDFMPGDSVGIKSITLLVEGVYAYGYLKAEKGVHRLVRISPFDANKKRHTSFASVDVLPEVDETVEVEINPGDLRIDTYRAGGAGGQHVNMTDSAVRITHIPTGIVTTCQQERSQLLNREKAMKVLQAKLLDLEIKKKEEEMKKLQGEQTDIGWGNQIRSYVFQPYTMVKDHRTGCESGNIRAVMDGDIDDFVNAYLRWIKR | K02836 | 364.17 | 533.9 | 3.90E-161 | peptide chain release factor 2 |  |  |
| GC_00000172 | MILANNKKAYFDYFVEDTIEAGIELVGSEVKSAKAGKVSIKEAFVRIINGEVFIMGMSIVPWQYGSVYNPEEKRVRKLLLNKKEIKKFHEKVTQKGYTIVPLNVHLSKGYVKVDIALGRGKKTYDKRESIAKKDQKRDMDRMLKVR | K03664 | 50.87 | 219 | 7.90E-66 | SsrA-binding protein |  |  |
| GC_00000173 | MNFIFIFILIFLIIILLAFHVRIVSQSRAYVIERLGAYLTTWQVGLNILMPFIDRVVKIVSLKEQVIDFPPQPVITKDNVTMQIDSVVYFQITDPKLYTYGVEHPMSAIENLTATTLRNIIGEMELDTTLTSRDTINTKMRAVLDEATDPWGIKINRIELKNIIPPREIQDAMEKQMKAEREKREAIRRAEGQKEAAILVAEGEKKSQILRAEAEKEAAILRAEAKKESLEKEAEGQAAAILSIQKAKAAAITALKEAGATKEVLALKGMETFEKVADGKSTKIIIPSELQNLASLTSVFGEMLKKDEIKE |  |  |  |  |  |  |  |
| GC_00000174 | MIRRKSREIKIGNLYMGGNNPVVIQSMTNTFTKNADATIKQILELEKEGCQLVRVTVNNEEAAETIKEIKKGIHIPLVADIHFDYKLAIKAIENGIDKLRINPGNIGDDEKVRLVVEKAKEYNVPIRIGVNSGSIEKKILEKYGKPTADGMVESAMYHVGLLEKFDFHNIVISIKASNVQMMIEAYRKIAALVDYPLHLGVTEAGTAFQGTVKSAIGIGSLLADGIGDTIRVSLTENPVKEIKVAKEILKVLGMRQGVEIISCPTCGRTEIDLINLAKKVEKEFGNMERNIKIAVMGCIVNGPGEAKEADYGVAGGKGVGLLFKKGEIIKKVKEEDIISELRAMIEGE | K03526 | 110.17 | 560.7 | 4.30E-169 | (E)-4-hydroxy-3-methylbut-2-enyl-diphosphate synthase [EC:1.17.7.1 1.17.7.3] |  |  |
| GC_00000175 | MRVKTGIVRRRKHKKVLRAAKGFRGASGDVIKQAKQATMRAAAYSTRDRKVKKRKMRQLWIIRINAGARINGLTYSTLMNGLKRAGIVLDRKVLADMALNNAAEFAKLAETAKAAL | K02887 | 32.43 | 159.9 | 1.10E-47 | large subunit ribosomal protein L20 |  |  |
| GC_00000176 | MQGKGIVEKINKCDKIYSKIKFREAENKMIGIGIVGLPNVGKSTLFNAITKAGAAEAANYPFCTIEPNVGMVTVPDKRIDELAKIINPQRVVQATVEFVDIAGLVAGASKGEGLGNKFLSNIRGTAAICQVVRCFDDDNVVHVSGGVDPIRDINVINGELILADMETIDKALEKNKKLLVTKNKDVMKLVPVLEKCQAHLNEEKLLKTLDLTDEEKELIKVYQLLTIKPMIFVTNVSEDDLAVGNEYVEKVREYAKGLDSEVVIVSAKVEEELQEMEEADKEEFLESLGVEEAGLNRLIRAGFKLLGLQTYFTAGVKEVRAWTINIGDTAPKAAGVIHTDFERGFIRAKVASYDDFIKYSGWKGTQEAGVMRVEGKDYVVQDGDLMEFLFNV | K06942 | 337.9 | 384.4 | 1.30E-115 | ribosome-binding ATPase | hyaluronidase | Exoenzyme |
| GC_00000177 | MTKKEKVKYILQKMEEKFGKPKCALNFETPFELLVAVILSAQCTDKRVNIVTEKMFREDKINTPEQFASMELSEIEELIRSTGFYKNKAKNIQKAAKQLLEKYNGELPKDMDKLLELGGVGRKTANVVRGEIWGLADGITVDTHVKRITNLLGLTEETDPVKIEKDLMKIVPKKSWIDFSHYIILQGRDKCIARRPQCQECEIKEVCRYFSKLQK | K10773 | 135.9 | 275.3 | 8.70E-83 | endonuclease III [EC:3.2.2.- 4.2.99.18] |  |  |
| GC_00000178 | MKDIKKVDFKGSVILNPVPVVLITSRNKEGKDNVFTVAWVGTVCTKPPMLSISIRPERLSYEYIKETMEFTVNLPSQDLVKKVDFCGVRSGRNIDKIKEMGFTMREGEKVSSPYINDCPVSIECQVKQIIPLGTHDVFIAEVVSSHVNENLMDEKGKIHFEWADLITYCHGEYFEMTKNPIGSFGYSVMKEKTKQRKAEEKSKEIKKKTKISENKNSDKTKEKRKKKFGGKNGQHKTGKRFV |  |  |  |  |  |  |  |
| GC_00000179 | MSFLTEFKKFAVRGNVMDMAVGVIIGGAFGKIVSSLVNDIVMPLIGIVTGRIDVSSLALTIPASKADGTPVIVKYGIFLQTTLDFIIMVFCIFVMVKMINKLKKEAPKAPPAPSKEEILLTEIRDLLKDNMKKN | K03282 | 40.47 | 178.2 | 3.10E-53 | large conductance mechanosensitive channel |  |  |
| GC_00000180 | MVKTDIQIAQEAKLLHISKIAEKLGLTEDDYDQYGKYKAKLDFNLLNKFANKEDGKLVLVTAITPTPAGEGKSTVSVGLTQALNKLGYSSLAALREPSLGPVFGIKGGATGGGYSQVVPMEDINLHFTGDLHAIGVAHNLISAVIDNHIKFGNQLNIDITKITWKRVLDMNDRSLRHTVIGLGGSASGIPRENSFQITVASEIMASLCLASSLKDLKERISKIIFGYDVTGKPLTVADLKITGAVAALLKDAIKPNLVQTLENTPVLIHGGPFANIAHGCNSLIATKLALKLGDYVITEAGFAADLGAEKFLDIKCRQGNLSPKCVVIVATVRALKHHGGAKELGVENLEALSKGLENLDKHIENMKKFNLPVVVAMNRFLTDIEAEFDLIRNRCKEQGVPVALCDVWARGGEGGIDLAKLVVEAVENNKEEYKPLYSLDLTPAQKIETIVKEIYGGDGVVFSATAKKTLAVIEKNGYNNLPICVSKTQKSLSDNANLLGRPTGFTVTINEVKIAAGAGFIIAMAGDIIDMPGLPKVPAAEMIDIDEFGKITGLF | K01938 | 885.83 | 919.3 | 1.20E-277 | formate--tetrahydrofolate ligase [EC:6.3.4.3] |  |  |
| GC_00000181 | MDRYKKLSYTFTLILVLVLYGVLGSLIGSGMISRYQTGILIIICINIILAVSLNITVGCLGQITIGHAGFMSVGAYAAALFSKAGIIDGLPGYVVALIIGGIVAGIVGIIIGIPALRLNGDYLAIITLAFGEIIRVLIEYFKFTGGAQGLRGIPRTNSNFTVIYFVMVISVMMMFSLMTSRHGRAVLSIRDDEIASGASGVNTTYYKTFAFTVSAIFAGVAGAVYAHHLGILGAKQFDFNYSINILTMVVLGEWEALQDLFFLQ | K01998 | 242.63 | 252.1 | 1.30E-75 | Branched-chain amino acid transport system permease protein |  |  |
| GC_00000182 | MTIGERIKKKRNEKGFSLRELAGKVDLSASFLSQIEQGKASPSIENLKKIANYLEVKVSYLIEEEDEALGSFHIKKEDRKYVESIDSKTSIALLTSSKIEKNMEPIMYEIKPGGESGRGFFNHNGEEFIYIVEGTLDIYIEDQLTTLNEGDSFYFKSSLNHRFKNNGKKLTKAIWIVTPPTF | K23779 | 88.83 | 101.8 | 6.00E-30 | XRE family transcriptional regulator, regulator of sulfur utilization | mannose-1-phosphate guanylyltransferase | Immune Modulation |
| GC_00000183 | MMKKGLRKVNTRLMVAGIPNVGKSRLINRIVGKNSAGVGNMPGYTRGKQWIRIKEGLELLDTPGILWPKFEDERVGFNLAISGAIKDDILPIDDVACKLIDKMMKYGLKENLKSRYKLLDEDFEGVTGNVIENIAKRMQMIQKGGNLNVHQATLTLLRDYRAAKLGKFGLDRELLSQEAKNEK |  |  |  |  |  | ferrous iron transporter B | Nutritional/Metabolic Factor |
| GC_00000184 | MAVVEIKHPLIEHKLTYLRDKNTDTKTFRENLNEIAKLMIYETTKDLELEEIEVETPIMKTKAGVLKDKAVAIVPILRAGLGMVDGILSLIPTAKVGHIGVYRNEETLQPVYYYCKLPVDIQERKVILVDPMLATGGSAIYAIDYLKDRGVKDITFMCLISAPEGIARVQEAHPEVSIYTAKIDERLNEHGYIVPGLGDCGDRIFGTK | K00761 | 209.53 | 344 | 7.70E-104 | uracil phosphoribosyltransferase [EC:2.4.2.9] |  |  |
| GC_00000185 | MKIALGCDHGGYELKEKVKKHLTEKGYEVTDFGCHSTESVNYPVYGKAVGHAVADKEADLGIVICGTGIGIGIAANKVKGVRAALCMNTTMARLTKEHNNANVLAMGARMLGDVLALEIVDEFLTAEFQGGRHAERIAMLED | K01808 | 180.27 | 242.6 | 7.40E-73 | ribose 5-phosphate isomerase B [EC:5.3.1.6] |  |  |
| GC_00000186 | MKIKNIAIIAHVDHGKTTLVDCLLRQAGVFGAHELEKVSDRVMDSNDIERERGITIFSKNASVKYKDYKINIVDTPGHADFGGEVQRIMKMVDSVILLVDAFEGPMPQTKYVLKKALEQGHRPIVVVNKVDRPNARPEEVLYMVYDLFLELNANEHQLEFPVLYASGKGGFAKKELDDPSENMVPLFETILSEVDDPEGDENKPLQFLITNIEYDNYVGQLAVGKIHNGKIRKNQEVMLIKRDGKMVKGKVSLLYGYEGLNRVEIPEATTGEIISIAGLSGLDIGETIADINNPEALPLISIDEPTLAMTFMVNNSPFAGKDGKYITSRNIWDRLQKELQKNVSMRVEATDSPDAFTVKGRGELQLSILLENMRREGYEVQVSKPRVIFKEIDGKTYEPVEIAVVDVEETFAGVVIEKLGGRKGELISMTPGQDGYTRLEFKIPSRGIIGYRNEFLTDTKGSGILNHSFYDFEPFKGPISGRKKGVLIATETGVSVAYALNALQDRGEMFIDPGINVYEGMVVGEHSKENDLVVNVCKTKKLTNTRAAGSDDAVKLAPPRKLTLEQALDYISEDEFVEVTPNFIRLRKKYLTDNERRRHFNKN | K06207 | 313.73 | 988.7 | 1.40E-298 | GTP-binding protein | elongation factor Tu | Adherence |
| GC_00000187 | MSKYVSRYHKLFDEVIKENLMKELDLKNIMECPTLEKIIVNMGVGEATQNAKLIDAAMNDLGIITGQKPLVRKAKKSEAGFKLRENMPIGAKVTLRKERMYDFLDRLVNVVLPRVRDFEGVPSNSFDGRGNYSLGLRDQLVFPEIEFDKVDKLQGMSITIVSSARTDEEGRALLKAFGMPFKK | K02931 | 99.57 | 303.7 | 1.30E-91 | large subunit ribosomal protein L5 |  |  |
| GC_00000188 | MKIGFDHNKYLEEQSKYILERVNNFDKLYLEFGGKLLYDLHAKRVLPGFDENAKIKLLHKLKENVEVVICVYAGDIERNKIRGDFGITYDMDVLRLIDDLRSYELEVNSVVITRFDDQPATTVFINKLERRGIKVYKHRATKGYPTDVDTIVSEEGYGRNPYIETTKPIVVVTAPGPGSGKLATCLSQLYHEDKRGKVAGYSKFETFPVWNVPLKHPLNIAYEAATVDLKDVNMIDPFHLEAYGETAVNYNRDIEAFPVLKRIIEKITGKESIYKSPTDMGVNRVGFGIVDDEVVREASKQEIIRRVLNAACDYKKGYIDKDTYQRAKLIMEELQLKETDRRVLRAARERLEKLKAEDPNEFYSAVAIELDDGTMITGKGSQTMCAAASAILNAIKYHAGIADEVHLIAPEIAEPIMNLKSINFKSKNVTLNCEEVLIALSICAVTDERAKRAIDNLYKLKGSQAHSTSILGKTDDQLVRKLGMDMTCDSVFPTENLYYND |  |  |  |  |  |  |  |
| GC_00000189 | MIRIGNGYDVHKLVEGRKLVLGGVEIPHAKGVLGHSDGDVLIHAVMDAVLGALSLGDIGKHFPDTDMKYEGIDSKILLKKVYELMSEKGYKIGNLDSIVVAQKPKLKDYIFEMRKRMAEILHTDIENVSVKATTEERLGFTGNEEGIKSYCVVLLEKT | K01770 | 247.83 | 262.3 | 5.80E-79 | 2-C-methyl-D-erythritol 2,4-cyclodiphosphate synthase [EC:4.6.1.12] |  |  |
| GC_00000190 | MKKEMVAMVLAGGQGSRLKLLTKNNAKPAVPFGGKYRIIDFPLSNCTNSGIDTVGVLIQYKPQILNNYIGIGRAWDLDRNFGGISLLPPYMQENGGWWYKGTANSIYQNMDFIDEYDPEYVLILSGDHIYKMDYNEMLKYHKSHNADATIAVIEVSLEEASRFGIMNVREDKQIYEFEEKPAHPKSTLASMGIYIFNWKTLKKALIEDEANPNSSNDFGKDIIPKLLGENCKLMAYPFKGYWKDVGTIESLWEANMDLINKDVDFDIYDKNWRIYSQAPNKPGQVIGDNAKIKNSLITEGCVIKGEVENSVLFSGVYIEEGAKVTNSVIMCDTRICKNSKVDRAIFGRKVLVKENLEVAGKSEILLIEEDRVVDENIVK | K00975 | 195.63 | 501.7 | 2.70E-151 | glucose-1-phosphate adenylyltransferase [EC:2.7.7.27] | glucose-1-phosphate thymidylyltransferase RfbA | Immune Modulation |
| GC_00000191 | MKFSNYLDSNLIFTDVKGSSMEEIIKEMVEKIASKEKSVNLRKDEITSAVIKREQEISTAIGKGVAIPHARIENFNDFIVAVGVVETPFRAKVEASASKDTVELVFLIISDVLKNKNILKIMSAVSKLVIKYPHIADKMKTIKDSAEILKAVQEADIEIGHKITAEDVLSPDIIPLSPNSTLEEVAKRFIIEHTSGLPVVDENGKFLGEITEKELIEFGMPKYLSLMQDLNFLTVGEPFEEYLVNEKTTIIENLYRSKDELIILDRKAPIMEICFIMVSKNITRLYVVENGEYLGLIKRSDIIKKVLHI |  |  |  |  |  |  |  |
| GC_00000192 | MNIMLFGAPGAGKGTQAKFIIDRYGIPQISTGDILRAAVKEGTPMGLEAKQFMEAGKLVPDSTIIGIIKERLSMDDCKKGFILDGFPRTLAQAEALEVLMKEMGIKLDKVISLNVPDELIVGRVTGRRVCKDCGASFHVEFNPSKVEGVCDLCGGELIQRKDDTAETVTKRLSEYHAQTAPLFDFYMERGILADLDGTKDIDEITKEIFNILG | K00939 | 189.4 | 252.1 | 1.30E-75 | adenylate kinase [EC:2.7.4.3] |  |  |
| GC_00000193 | MNFEESFKSSIKALKGNKVRSFLTMLGIVIGIASVITMSAIGKGGQESITGNLKKTGYGKFTVYVDREDQAFRWKYLLDDDIIEKLKNTDKFKAVSPMIKKRLFAKLGGRDEIMWLTVTTPEYEEIDKVNITEGRSLLSFEYKTGERSVLIDHITAKDLFGSSENALGKSLEMSESRKSVKIPYTIVGVFQNQFEQYVKAMGGRRIPRFVRMPLRTYDKVYDLKANGYTDIVIESKNPDFMSEDMGEAKRLLEKFTGVKDLYEVGTLSDGASSFDSILTTLNIFVIFVAGISLFVGGIGVMNIMLVSVIERTKEIGIRKAIGAADKNILMQFLLEAVILTGCGGIIGIISGILLGIVIGNIVGIPPVFSLWSIIISLAVSMSIGIIFGVIPAKKAAKLNPIDALRSE | K02004 | 47.37 | 120.5 | 7.30E-36 | putative ABC transport system permease protein |  |  |
| GC_00000194 | MNIVLFEPEIPYNTGNIGRSCVLTNTKLHLIKPLGFSIDEKEVRRAGLDYWHLVDLTVWESFEDFVKGNPNGNFYFATTKCKNRYSDVKYEENDFIIFGPESRGLPKEILEKYADRCITIPMIPMGRSLNLSNSAAIILYEALRQTGFHFADEE | K03216 | 93.93 | 235.5 | 4.40E-71 | tRNA (cytidine/uridine-2'-O-)-methyltransferase [EC:2.1.1.207] |  |  |
| GC_00000195 | MAKSKIGIVLNGIFKENPVLVLLLGLCPTLGTSSSAINGMSMGLATTAVLVFSNILISVFKKVIPDKVRIPAFIMIIASLVTIVQMLMEAYTPDIYKVLGLYIPLIVVNCIVLGRAESFASKNSVIDSMFDGIGSGLGFTLALTVLGMIREVLGNGTIFNIVITPANWQPALIFILPPGGFLTIACVIAFQNYLKQKKEV | K03613 | 244.93 | 321.8 | 2.80E-97 | H+/Na+-translocating ferredoxin:NAD+ oxidoreductase subunit E |  |  |
| GC_00000196 | MKSNKVVLSAKNISITFGALKAVTDFNLEIKENELVGLIGPNGAGKTTVFNIITGVYSPTSGEYYFNGEPVTKTPTYKLVKKGLARTFQNIRLFKYMSVLDNVLVANNFNMKYGILSGIFRFPNYWREEKEAKAKAMELLKIFDLDQYADTAAGNLPYGKQRKLEIARAMATNPKLLLLDEPAAGMNPTETEELMNTIRLIRDKFNIAILLIEHDMKLVLGICERLVVLDHGTTIAAGDPIEVINTPAVVTAYLGQEEE | K01995 | 318.13 | 423.1 | 3.10E-127 | branched-chain amino acid transport system ATP-binding protein | ABC (ATP-binding cassette) transporter CylA | Exotoxin |
| GC_00000197 | MKKKNKVIYVGKAKNLKKRVSSYFNREHEDEKTINLVKNIEDIETIVCNSEIDAFVLENNLIKKYSPKYNIALKDEKTYPYIKISKEKFPKISIIRTTRALDTKSGDYFGPYPQGAWFLLKTLIKIFKIRDCNKDMEKEIQRPCLKYHMGMCPAPCKFKNIEAEYRENVENAVKVLKGQGNFIIKELEKKMLSASENMEFEKAIEYREQKNEIEKALNNQVTEYGRDIDEDVFTFIHDGNLIFICVLNMREGKLLGKISLTVSTEEKIYSEIFENIVSEFYSKHPIPQNIIFQPEYIESREIISEWLKIKSGKNISLHFPKIASRREELLNMALLNLNKDIVIYYDKKSVIESGMAKLYKVLELKNFPRVIECFDISNIQGKDAVASMSVSVEGKAAPKNYRKFKIRCKDTPDDFQMMREVITRRYSKLEPKDFPDVILIDGGIGQINAAGEVLKNLGKDNISDLLSLAEREELIYKYGNNEPFAFSHSEEGLKILIRVRDEAHRFGVTYHRKLRSKRVISSELDKIEGIGPVRREKLLKHFGSVKNIKEASLDDLKLILPEKTAVLLLEKLNKGE | K03703 | 235.03 | 599.3 | 1.40E-180 | excinuclease ABC subunit C |  |  |
| GC_00000198 | MSSYGKKVNTVFENFYKLFYESEDLALKRGIKCLTHTELHTIEAIGKDCITMNELSERLAITMGTATVAITKLGEKGFVTRARSNSDRRKVHVSLSKKELRHLTITTIITK |  |  |  |  |  | PapX protein regulates flagellum synthesis to repress motility | Adherence |
| GC_00000199 | MVRGSSGKESVKSLVQIIKLVKNEGYSLGTPLDGPKGPVYEVKPGMIYAAQKSRKQLVLVGGAYSKKWIFSKTWDKFQLPKPFSKVVCIVGEPIDVPKDIDPKDYSGFVKEKLNELNDLAEKEILKIK |  |  |  |  |  |  |  |
| GC_00000200 | MNPNIFTENSILAINDSRDLAIKYKQSSIKPEILALALLLNKEGLIPRVIEKMNLNVSSIISGLEREIEKFPKIEGNGLGDVSIDSAANRVLIDAEEEMKKMGDSYVSVEHIFISLLKECKTLQKLGIDLNKFREAIKTIRGNQKVDSQNPESKYEVLDKYAKDLVELAREGKIDPIIGRDAEIRRTIQIISRRTKNNPILIGEPGVGKTAIAEGLAQRILNGDVPENLKDRKIFSLDMGALIAGAKYRGEFEERLKAVLKEVQASEGKIILFIDEIHTIVGAGKTDGAMDAGNLLKPMLARGEVRVIGATTIDEYRKYIEKDPALERRFQTVLVDEPTVEDTISVLRGLKEKYEVYHGIRISDGAIVSAAVLSDRYISDRFLPDKAIDLIDEAAAMIRTEIDSMPSELDELTRKSMQLEIEREALKKEDDEASKERLKALEKELAEINANKSVLKSQWEVEKQEIEKVKQLKAEIDKTKLEIEKAERNYDLNKLAELKYGTLATLEKQLKEQQEASDKKFDHALLKLEVSENEIADIVSKWTGIPVSKLVESEKEKILNLEKSLNERVMGQEEAVKLVADTILRARAGLKDKRRPIGSFIFLGPTGVGKTYLAKSLAYNLFDNEDNMIRIDMSEYMDKFSVTRLIGAPPGYVGYEEGGQLTEAVRRKPYSVILFDEIEKAHPDVFNTFLQILDDGRLTDGQGRVVDFKNTLIIMTSNVGSSLILEDPDLSEETKKSVNNMLKQGFKPEFLNRIDDIIIFKSLSLESVKDIVKQLLKETQEKLKDKYIKLEFTNDVIDYLAVNSYDPHYGARPLKRFIQKEIETELAKKVLSNEIKEKDSVTAELVDGKIKFTVTQ | K03695 | 1085.8 | 1261.5 | 0 | ATP-dependent Clp protease ATP-binding subunit ClpB | endopeptidase Clp ATP-binding chain C | Stress survival |
| GC_00000201 | MDLAKLFSIIITSIFIQNYVFGRVLGICPYMGVSKKVESSIGMGMAIIFVISIASAVTWLIYQYMLVPFGLEYLQTIMFILIIASLVQFVEMAIQKMSPNLYNALGVYLPLITTNCVVLGVAILNIQEGYNFIETIVNGIGAAVGFTLALILLAGVRERIEYSNIPKPFQGVPIAFISATCLALAFMGFAGMQI | K03617 | 244.63 | 306.5 | 3.00E-92 | H+/Na+-translocating ferredoxin:NAD+ oxidoreductase subunit A |  |  |
| GC_00000202 | MPILSAAMDTVTEADLAIALARQGGLGFIHKNMTIEEQAKEVDKVKRNESGMIKDPITLTKDCTLADADELMGQYKISGLPVIEEDGKLIGIITNRDLKYRKDYDTPVIDVMTKENLITAPVGTGLEEAKEILISNRIEKLPIVDENGYLKGLITIKDIDKIVEYPNACKDEHGRLRVGAAVGVGNDTLERVAALVKAGADIITVDSAHGHSAGVIRRVKEIRDAFPDIQLVAGNIVTAEAALALIEAGVDAVKVGIGPGSICTTRVVAGVGVPQLTAVNDVYQVCKERGIGVIADGGIKLSGDIVKALASGADCVMLGGVLAGTKEAPGEEVIYEGRRYKVYVGMGSLAAMKRGSKDRYFQNDAKKLVPEGIEGRIAYRGDLKDVIFQLCGGIRAGMGYCGTPTIKDLQLNGKFVKITGAGLKESHPHDIQITKEAPNYSR | K00088 | 372.73 | 697.1 | 6.20E-210 | IMP dehydrogenase [EC:1.1.1.205] | D-arabinose 5-phosphate isomerase | Immune Modulation |
| GC_00000203 | MINISNALEKNLLLEVKNKLILLNPIDIASILETLSKENTLKAFRILSKDLASEVFSYLSSEKQQEIIESSSDEELKRIIDDMFLDDTVDLIEEMPAGVVAKILKNTSVENRKLINQFLKYPEDSAGSIMTVEYVSLKNDMNVKESLERIRAKGIKNETINDCFVIDKERKLVGTLPIRELIINQEETLIKNIMTDNFEKVQADTDREFVADLFRRYDLSTMAVVDTENRLVGIITIDDIVDVIDQENTEDFQKMAAMEPSDKEYLKESVFALAKHRILWLLILMISATATGTIIRRYEDTLQSVVVLAAFIPMLMDTGGNAGSQSSTLIIRGLALGEIKTKDVWKILWKEFRVSIIVGITLAIVNFLRILYFDKIGFTMTLVVCLSLFATVAIAKVVGGILPIAAKKLKLDPAIMASPLITTIVDACALVIYFTMATALLHI | K06213 | 103.37 | 552.7 | 1.20E-166 | magnesium transporter |  |  |
| GC_00000204 | MLLNSILLILFLVNIILNNIKVIGAVFFGELLLNMFLNPNLKNNIKKLKVLIYIYFGTFIIQILSVQEGEVLFKIFSIYITKTAILNFAVNFMRIINLIMLSWLVSKKSSIFNHFGEYKRVMENVVELVPEVFVIFRKRMRLKNFFRHIFKKIKI |  |  |  |  |  |  |  |
| GC_00000205 | MLENLGNRFQDIFKKVRGHGKLSESNIKDALREVKMSLLEADVNYKVVKDFINKIQEKAIGTEVIRGINPGQQFIKIVNDELVELLGGTNSKLTKGVKNPTVLMLAGLQGAGKTTFAAKLANKLKKDGERPYLVAADVYRPAAIKQLQVLGEQINVPVYADEENKDPVDIAKKAWGQAKANDYTYMIIDTAGRLHVDEALMEELSQIKKTVRPQEILLVVDAMIGQDAVNLAKNFNDKLNIDGVVLTKFDGDTRGGAALSIKSVVGKPIKFVGVGEKIEDLELFHPERLASRILGMGDVVSLVEKAQENINEDDAKSLEEKIRTQKFNLEDFLKMLHMVKKMGPLASILKMLPGVGNDLGDLSLAEKEMKKVRAIIQSMTREERAKPEILKASRKMRIAKGSGTDVSDVNRLLKQFEMMKNMMKMFSSGKMPNLGAMKNMGTGMPNMRNGRGKGRFPF | K03106 | 333.4 | 696.2 | 7.80E-210 | signal recognition particle subunit SRP54 [EC:3.6.5.4] | flagellar biosynthesis regulator FlhF | Motility |
| GC_00000206 | MLHTVEEAIEDIRNGKCIVVVDNEDRENEGDVICAAEFATTENVNFMATYAKGLICMPMSREIVKKLDLPQMVTENTDNHCTAFTISIDHISTTTGISAYERGITAVKCTEEDAKPSDFRRPGHMFPLLAKDNGVLERDGHTEATVDLVRLAGLKPVGLCCEIMNVDGTMSRFEDLQKFAKEHDLKMISIEELIKYRKAHDELMKIECRAKMPTAYGTFEIVGFDNKLDGKEHIALVKGDVNGKEDVLVRIHSECFTGDILGSYRCDCGLQLKTAMQRIEKFGEGIVLYLRQEGRGIGLVNKIKAYKLQDEGYDTVDANLKLGFESDARDYAVAAQMLKALGVKSVRLMTNNPEKIKGLESYGIKVAERREIEIPANEINEGYLRTKQERMGHELHIGKCSCGCEKNK | K14652 | 354.33 | 641.3 | 1.90E-193 | 3,4-dihydroxy 2-butanone 4-phosphate synthase / GTP cyclohydrolase II [EC:4.1.99.12 3.5.4.25] |  |  |
| GC_00000207 | MKKILTIFFIFVSLAVYGSPEGKAYIGSFNALRLGETKKDYKELSNILVLLDIIGLQEVSNREGVETLVDEISKNTNEKWDYHISPYPVGTKKYKEYYAYIWKKDKVSFIKSRGFYKDKGDKFIREPYGADFKIGEFDFTFVIIHAVYGKNKSVRIAEAMNLPKVYDYFQKLDEKENDIIIGGDFNLSVRSEGFSNLLNHEDKIINCISPNMKTTIGTKGYANQYDNIFISEIYTKEFTGRSGGIDTANGNYKKTREVISDHIPVFIEADTSKDDD |  |  |  |  |  |  |  |
| GC_00000208 | MGFFDFLKGKKAEEWFEVYSPLNGKVIPLSEVPDEAFAQKMIGDGCAIDPAPGAIYAPVDGEIDIFETNHAVSLEAPNGIEMIVHFGIDTVKLNSEGLKRVADVGSAKKGDKLIEYDLDYIRENAKSTKTPIIITSMDMVDTIEVVASGDVKVGDLLMRVKLKK |  |  |  |  |  |  |  |
| GC_00000209 | MLNIVKITDYMSKDLIALDLKSRNKEAVLEELSVLMSKSENIQDKNIIKKALMEREELGSTGIGKGIAIPHAKTDAAKKLTVAFGISREKIDFDSLDKEGVNIFFVFASPIEDSQIYLKVLARISRLIRNEGFRNKLLNCHTPEEVIKYIDEEEAV | K02806 | 156.53 | 161.9 | 3.00E-48 | nitrogen PTS system EIIA component [EC:2.7.1.-] |  |  |
| GC_00000210 | MRKTIIAGNWKMNKTNADAVAMLTELKAAVAGVENVGIVIGAPFTALSDAVKAVAGSNIKIAAENMYPKASGAYTGEVSPEMLKAIGVEYVILGHSERREYFHETDAFINEKVKCALAYGLTPILCVGEKLEDREAGRTDFVNETQVKGGLAGLTKEEAVKVVIAYEPVWAIGTGKTATPEIAEETHKAIRKVLAEMFGAEAAEEITIQYGGSMKADNAKDLLAQADIDGGLVGGASLEAESFAKIVKAGM | K01803 | 29.1 | 359.5 | 2.80E-108 | triosephosphate isomerase (TIM) [EC:5.3.1.1] |  |  |
| GC_00000211 | MTRIIDVIAREILDSRGNPTVEVDVVLECGAKGRAAVPSGASTGAYEAVELRDNDKSRYLGKGVLTAVKNVNTEIKEAILGMDALDQVRIDKTMIALDGTPNKGRLGANAILGVSLAVAKAAAEALGMPLYKYLGGVNTTELPLPMMNILNGGSHADSAVDVQEFMIQPVGASNFMEAMRMGCEVFHHLGKLLKANGDSTNVGNEGGYAPAKINGTEGALDLMVEAIKKAGYEPGKDITFAMDAASSEFCKEVAPGKFEYHFEREGGVTRTSEEMVEWYAGLVEKYPIKSIEDGLGEDDWAGWQLLTARLGDKVQLVGDDLFVTNTERLKKGIELKAANSILIKLNQIGSLTETLDAIEMAKRAGMTAVVSHRSGETEDATIADIAVATNAGQIKTGSTSRTDRMAKYNQLLRIEEELGDMAQYKGMDVFYNLSK | K01689 | 65.2 | 639.5 | 5.80E-193 | enolase [EC:4.2.1.11] |  |  |
| GC_00000212 | MKPIVAIVGRPNVGKSTLFNKLVGDRVAIVDDQPGVTRDRLYRDTEWSGKEFVLVDTGGLEPRNNDFMMSKIKEQAEVAMNEADVILFVVDGKAGLNPLDEEIAYYLRKKHKPIILCVNKIDNYQTQQEDLYDFWALGFDNLIGISAEHKTNLGDMLDLVVDLIDTVEMPEEEEGLKIAIIGKPNAGKSSLVNKLSGKERAIVSNIAGTTRDAIDTPIEYEGQKYVLIDTAGIRRKSKVEEALEYYSVLKALKAIKRADVCFLLFDGKEGLSEQDKRIAGIAHEEKKPIVIVVNKWDLIEKEKNTMKEMKEYLLGELPFLSYAPIEFISALTGQRTTRLFGIAEKIYEEYIRRISTGLLNTVINEAVIMNPPPTRKGRVTKVNYATQIATAPPRFVLFCNYPELMHFSYARYIENKLREAFGFEGSPIEIIFEKKNQEKQ | K03977 | 253 | 655.2 | 9.80E-198 | GTPase | Fe(2+) transporter permease subunit FeoB | Nutritional/Metabolic Factor |
| GC_00000213 | MSTKLIDYDYNLPEELIGQHPAEPRDHSRLMAVDKKNETTEDKHFYDIIDYLKEGDVLVRNSTKVIPARLFGRKETGAVLEVLLLKRINLDTWECLVGHAKKLKLGQKVYIGNNNELIGELIEIKDDGNRIIKFSYEGVFEEVLDHLGKMPLPPYIVEALQDQSRYQTVYAIKGESVAAPTAGLHFTKELLKKIEDKGVTIVDIFLEVGLGTFRPVQTEDVLDHKMHEERFEIPKEACEIINRAKKEGRRIVAVGTTTVRALESAAKDDGTLEAESSSTDIFIYPGYRFKIIDALITNFHLPKSTLLMLVSAFSNREFMLKVYKEAVEKKYHFFSFGDAMFIY | K07568 | 194.97 | 495.5 | 1.50E-149 | S-adenosylmethionine:tRNA ribosyltransferase-isomerase [EC:2.4.99.17] |  |  |
| GC_00000214 | MKLNKGYFVTGTDTGIGKTYVSAFLYKAVKKYEGAYYKPVQSGCIEKNGRLTAPDIDFVCSFNNEEYDISKGTYFLKEEVSPHLAAEIENIEINFEKIKKYWENLKIKYKTIIVEGAGGVYVPLVRNKYFIFNLIKDLNIPVILVCSTKVGAVNHALLTINFLKNTGIKIHGIVFNRVSENIKNFEKDNIEIILKTGEIKKYLIIKENQKNISEDEILKFLEEDNE | K01935 | 116.97 | 186.1 | 1.40E-55 | dethiobiotin synthetase [EC:6.3.3.3] | dethiobiotin synthetase | Nutritional/Metabolic Factor |
| GC_00000215 | MFSSNSEIRYTLIVAAAGVGKRMGLSYPKQFLEHNGKPLFINVLEIGEKSKLISDIIIVTGKELKEEVKNMCEKFKITKVKEIVEGGKERQDSIYNALKFCDKNSIIAVQDGVRPFFKEKYLEDAFNELKNNPYIDGVVVGVPVKDTIKVVNEEGIIISTPVRRALVAAQTPQVFRGKILIEAYEKAKEENFLGTDDSSLVEKYGGKVKISLGDYGNIKITTIEDISFLKREGD |  |  |  |  |  | Bcs1' | Immune Modulation |
| GC_00000216 | MKFVTDFNKKAVFYKNKEYSYKDIIRTAKYFSSLIEMKKNEDKAVIFMENRPEFICSFFGIWNSHGVPVNIDAGYTAEELEYILTDAEPKYIFTSEKNLKTAEEAVKLSGKEIKIINVDTLFIPENFEVDEYVIYSPETEDTGVLLYTSGTTGKPKGVVLTFDNLMSNVDAITEIKLATPQDRVLALLPYHHVLPLSINLLMAIHIGTLIVINDELSAQAIQEALKKYKITIVVGVPRLWEMIHKGIMTKIKANSIALKMFNICKKVNSQTLSRIVFKKVHEGLGGNIRFLVSGGAKIEPSILEDFKTLGIKVLEGYGLTETSPIIAFNRPDDIHIGTVGTTIPGVSAKLADDGEIIVKGRNVMKGYYKKPAATQEVIDDRGWFHTGDLGKIEDGYISIVGRKKEMIVLSNGKNINPADIENEIFKGTDLIHDIAVVEHNNHLLALVYPDFDKVKERKITNITETLKWEIIDSYNVKAPAYRKILEIKIVKEELPKTKLGKLRRFMLKDVIKNLDKPKTEEKKNEIKIDKSDKEYGTKEFHALSDYMKKEHDLEITPDSHIEIDLGLDSLDVVEMNAFIEKTFDFSVNEDEAGGIKVIRDICEYIRVHSNTYHNESVNWGDILNERVDYKLPKSWAVGLFRILTAPIFKFYLKLTKKGQEKISSEPRIYVLNHESFADAFALGHMFTYKQAKNVYFFAIKKHFEKPVRRFFADNGNIVLVDINKNLKESLQIAAEVLKENKSLVIFPEGARTRDGEIHDFKKFFAILSKELNIPVTVMGIKGFYESMPFGSSFPRSGSVEIEVLGDIIPEKISVEEIVEKSRNLIVEWKKKK | K01897 | 441.3 | 447.9 | 8.90E-135 | long-chain acyl-CoA synthetase [EC:6.2.1.3] | Cereulide synthetase B, CesB | Exotoxin |
| GC_00000217 | MNKLRLLFEIGMEENPARFLVKALDDLKKNLENKLKNERIKYEDIKTFGTPRRMVILVEGLAEKQEDLNELNMGPARKVAYDANGELSRAGLGFAKSQGVEGTDLEIVDTPKGEYIAVRKFSEGVATKTLLPEILKSLVLELEFPKSMKWADRKFKFARPIQWFLAMADNDVVEFEIEGIKSGLSSKGHRFFGKPFTVESIDDYFTKIRENNVIIDIKERKQMIRDLIDKNCTKENEQVLIHENLLNEVTNLVEYPFPIVGTFNSDFLEVPQEVLIISMEVHQRYFPILDKNGKLLPKFVVVRNGIEYSDKVKAGNEKVLSARLADARFFYQEDLKNPLEANVPKLATVVFQKDLGTIADKMARVTKLADFLTDKLGYTAEKEDIFRTIKLCKADLVSNMINEKEFTELQGLMGADYALKSGEKETVSKGIQEHYFPRFKGDKLPETKEGIVAGICDRMDTLAGCLGVGVTVTGSKDPFALRRAALGIVNVIINSKLNVSLKELVEKSIEILDEAGVLQKDKALLEKEVLEFLKQRVINVFTDMGYRRDVILAVVAKAWDNVIETKAMIEVLEKEVQEDSFKNLVGIIKRVGNIVKDHTEREVNKELFKEAAETSLYDYVEELDRTTAALLAAKDYKGYLDAVLNGEEIVNNYFNSVMINDKDAAVKNNRLSQMKRLDDIYERMADLDLIEG | K01879 | 560.07 | 782.7 | 7.40E-236 | glycyl-tRNA synthetase beta chain [EC:6.1.1.14] |  |  |
| GC_00000218 | MRLLEILEFSKEYLKKYSFSKPRLESEKVISHILGIKRIELYSNFEMELESGEKDKIKIFLREMARKRKTFDEVIKEKENSFEEKQEEKSSEKKSFKDENINLLSKSIQYLKKYNVENARLDAEYIFAYVLGVKRISLMLNFDEEISEENKNLIRQYIVRRGKYREPLQYIVKEWEFYGYPIKVDGRVLIPRQDTEILVEQCIYLMKEKENPKILDIGTGSGAISIALAKELPESEVLGLDVSDDALKMAVINRELNNVSNLKFLKSDVFQHVREKNYDLIVSNPPYIPVEEYNELMPEVKEYEPRMALTDGGDGYYFYKKISEESVNYLKNGGYLAFEVGYNQGETVSQLMEKNGFQIIGRVADYGGIERVIIGRKEEK | K02493 | 237.37 | 309.4 | 5.60E-93 | release factor glutamine methyltransferase [EC:2.1.1.297] | class I SAM-dependent methyltransferase | Immune Modulation |
| GC_00000219 | MAKGKSVYVCSECGYKTSKWVGKCMNCGSWGTIEEQEEISSSVSSSKVNRNSLSLVDTEKKVSPFSNITIEENFRFKTKQSEFDRLLGGGLVQGEVVLLTGNPGIGKSTLLLQMAKEYSDYGDVLYVSGEESPAQIKSRGERLGIKSRTLYLMSETDIDKISEYVSVKKPKVVIVDSIQTLYSENSDSIAGTPTQIREATLKIVELAKRMGISFFIVGHITKDGKVAGPKLLEHMVDAVFSFEGEEGLFYRILRSTKNRFGSTNELAVYSMEEDGMREVQNSSEFFLSEREEKNIGSMIVPVLEGTKVFLLEIQSLLTESGGIGIPKRIVQGFDRNRIQILMAIGEKRMGMNLGMKDVFINIPGGISIKDPSADLGALISLLSVYKNVAISQKIAAIGELGLRGEIRKVFFIDKRLRELEKLGFKGVYVPEANRKEIEKKEYKLKLIYLKNLEEFLERM | K04485 | 154.2 | 569.3 | 9.90E-172 | DNA repair protein RadA/Sms |  |  |
| GC_00000220 | MTVYEYDYPLYRPPSEAYSLIIQATLGCSQNKCTFCSMYKSKKFTIKPLEQIKKEIDFFRIYVKKAERIFLADGDALIMPMKILKEIFIYINEKFPEAERISLYGSPKSILLKTPEELLELKNLGLGLIYLGVESGSDKILSSVKKGVSREEIIAAGKKVKKVGIPLSVTAVAGLGGKENSIEHAVETASLISEINPDYFGVLTLMLEEGTELLEEYKKGNFIPLSSYEILEETKLMIKNINVKEKCIFRSNHASNYVSLKGTLPYEKENILKTIDSALENNEIKSEFLRRL |  |  |  |  |  |  |  |
| GC_00000221 | MENINIYLEIEKLLAFAENVKLIEKEDIVYSRNKLLAVFGLDDCEEVTETFEIEKPYDILNRMCDWAAEKGIIENTFDERDLFDTKVMGELTPRPSEVIRKFKEDYEVSPETATDNYYAFSQNTNYIRVDRIAKNLHWLADTEYGNIEITVNLSKPEKDPRDIAKAKLAPQSSYPKCLLCKENEGYQGRMNHPARQNHRIIPVTLTNEPWFLQYSPYVYYNEHCILFSGEHRPMKISRGSFERVLEFVDIFPHYFVGSNADLPIVGGSILSHDHFQGGHHDFPMAVAEAEETFTIKGFEDVTVEKVKWPMSVIRLRGESKEKLVDLSDKILTAWRGYSDEKCEILAFTGDTPHNTITPIARKKNGAYEIDLVLRNNRTSEEYPLGIFHPHQELHHIKKENIGLIEVMGLAVLPGRLKEEMKLLEKLMVEKNAASLIRENEKVEKHADWCEEILKKYESITAENVENIIKVEIGIAFSKVLENAGVYKQDEEGKAGYRRFIEFLNK |  |  |  |  |  |  |  |
| GC_00000222 | MEGIVEVLISREKVEERIKELAKEIEKDYAGKELVCVGLLKGSVMFMADLIKAVDLDLRIDFMKVSSYGSGTNSTGVVKILKDVDVDLAGKDVLIVEDIIDTGLTILNVKDFLSKKNPNSVRVCTLLDKPSRRVVEVKGEYVGFEIPDEFVVGYGLDLDEKYRNLPFVGRFVRK | K00760 | 85.63 | 229.3 | 1.40E-68 | hypoxanthine phosphoribosyltransferase [EC:2.4.2.8] |  |  |
| GC_00000224 | MKRLEESLKEITVLNKNSINKCQKIWDSKMKPAGSLGVMEDIVLKIAGIFEDSFENIKTKGCHIVAAADNGVTDEGVSSCPVEYTRIVAEAMLNKTAAIGIMCSNLGIDLKVVDTGIKNEIPRKYSNLYEMKVMNGTENFYKNPAMTKEQAVETIENGILFMEELEEDYDIFSTGEMGIGNTTTSSAILYSLAKSSIEDVVGRGGGLSDESFMKKKKVIFESCVKYNTFQLDTLEILADVGGLDIAFLTGLYIGAAKCRKLILVDGFISGVAALAACKLNPLIKDYILVTHLSEEPGMKIIIRELGAKPFLDMNLRLGEGTGAVLAYPVIKSAVEIYKNMKTPEEVYKLFNN | K00768 | 100.83 | 354.2 | 1.80E-106 | nicotinate-nucleotide--dimethylbenzimidazole phosphoribosyltransferase [EC:2.4.2.21] |  |  |
| GC_00000225 | MESADVILMKRDLRDVAVAMKLSHAVIKNIKENLFWAFLYNSLGIPVAAGVLYLITGHLLNPMIAGGAMAMSSVSVVTNALRLRNFGK |  |  |  |  |  |  |  |
| GC_00000226 | MNPDLIVVVAYGKIIPQSIIDIPKYGIINVHSSLLPKYRGAAPINAALINGDDKTGVTIMYIAEELDAGDIILTKETEITEEETFLTLHDKLKDIGAEALTEAVRMIFEGTNPRIVQNHSEATFVKPFKKEDLKIDWNKGEKEIYNFVRGINPFPCAFTTHNGKMLKVYEVKKNGKVYENGENGEIVDKIKGKGPVVKVGDGSLILTLAKPESKKVLSGADLLNGNVIKIGEKLL |  |  |  |  |  | GDP-mannose 4,6-dehydratase /GDP-4-amino-4,6-dideoxy-D-mannose formyltransferase | Immune Modulation |
| GC_00000227 | MQNTVGQNKVDVIKERAVSINPEIKIHAFKEKYDESTKDIFFKDKSYDYIVDAIDLVSSKLNLIETAAEKNIPIISSMGTGNKINPTMLEVSDINKTSVCPLAKVMRRELKARGVKKLKVVYSKELPMKPVNEEGGREKSKNVGSISFVPSAAGLIIGGEVIKDICGSKNSGGN | K22132 | 158.77 | 166.8 | 1.30E-49 | tRNA threonylcarbamoyladenosine dehydratase |  |  |
| GC_00000228 | MIRLNTHSHNILEFNKLKDVLNEYMVIEKNKERVYELGIYKDINSLRKDFEIVRDFIDFSKYDGGIETAGMRDILEILKKCDLMGMYFEPEELYDINQNLRLFRLFKNRLEDLDKYKELKGKFLSVPVVKFIEDVINKTIDNEKKIQDDASLDLRDIRAQKKLLSANIKRKFDDMFSNETYSKAIQEKIITIRDGRSVIPVKLDFKGLIKGIEHDRSSSGQTVFIEPLSIVSLNNKMRELEAREREEIRKILLRITDQIRMNAEDIRKIGEGVLELDFLNGKSRYAIENSCAIPEINAREQLSIVEGRHPFIEKDKVVPLTFEIGRKYNTLLITGPNTGGKTVALKVAGLLTLMALAGIPIPAKETSSIGFFSGVYADIGDEQSIEQSLSSFSAHLKNVQEILSSVTKNSLVLLDELGSGTDPVEGSAFAMAVIDYLKDRKVKSMITTHYSEVKAYGYNEEEIETASMEFNADTLSPTYRLLIGIPGESNALTIAKRLGVCDEVIEKAKSYISDENKKVEKMISNIKDKADELEAMQVEVERLKQKAKKDQEEYEERLRQLEAEKNIILKEAYEKADAMIKDMQNKAAALVKKLQSEENKKDDMKNVQKSLNMLRTSLDQEKKANVEQKPKTVRKVDIKEGEKVLVLSLKQYAVVLKINLSKETAFIQAGILKLEVPLDDLKKITEKKEKTYSSASTSSRTSVKPKIDLRGKMVEEAIYELESYFDRAMLNGYKEVQVIHGNGTGALRKGIVEYLKTCRYVKEFRFGGQGEGGVGCSVVTLK | K07456 | 303.07 | 750.3 | 3.80E-226 | DNA mismatch repair protein MutS2 | Dot/Icm type IV secretion system effector LepB | T4SS Effector Delivery System |
| GC_00000229 | MENRLPFLPTRDLVIFPGVVTPVYVGREKSMKTLEKLENSENTKMLFGMQKETLKEEPKLPEDIYTTGVIVNVLQSIKMPNKTIKILVEAEKRVLLENPKEEEDGSYSSGYVEVECKNSDSKETLAVYRKVIEYYERYTKFLGKTLPEVLVTLKSTKDINGGFDLIANNLFIDSQDKQKILEVLDVEQRGYMILDLLSKEIEINEIEKKVEDKVRNKMNDAQKAYYLKEKINAMKEEIQDYTPEDEDKELAERIEKAKLPAEVKKKVDEELKKMSKMPGFSAEASVSRNYIETLLELPWKKTTKDDLDIERASSVLDRDHYGLKEVKERILDYLAVKKLNPKMKGTIICLVGPPGVGKTSLAKSVADSMGRKFVRVSLGGVRDEAEIRGHRRTYIGSMPGRIMKAMKLAGVKNPLILLDELDKMSSDFKGDPASAMLEVLDPEQNIHFEDHYIDVPYDLSQVFFLATANDLRNIPEPLIDRMEIISLSSYTEYEKLHIAKQYLVKQLQEENGLKDIKITISDNVILKIINEYTREAGVRSLKREINNLFRKLARKVVKEKLEKITVNAGNLEKYLGKAKFRPEKMKERTYKVGIVNGLAWTSVGGITLEVQGVLIPGKGALNLTGTLGNVMKESAEVSFTYVKSNFEKYHINEKEFLEKKNIHLHFPEGATPKDGPSAGIAITTAILSVLTGREIRQDIAMTGEITITGEVLAIGGVKEKVIGAHRAGIREVILPEDNRPDIADIPHEVAKDMKINCVKNYDEVEKLVFKK | K01338 | 851 | 948.5 | 5.10E-286 | ATP-dependent Lon protease [EC:3.4.21.53] | endopeptidase Clp ATP-binding chain C | Stress survival |
| GC_00000230 | MYNHYKRIKLADETDKSVDVQKSNVLIIGPTGSGKTLLAQTLARILNIPFAIADATTLTEAGYVGDDVENVLVRLLQSADYDVEAAEKGIVYIDEIDKIARKSENMSITRDVSGEGVQQALLKIIEGTKSQVPPQGGRKHPNQELIEIDTKNILFIVGGAFEGLEKVISKRTQQKSLGFGADTSRVKRGEEGEVLRKVTPEDLIKQGIIPELVGRLPLVTTLDSLDEDALLRILVEPKNAIVKQYQKFFEMEGIELVITEGALREVAKRAMARRIGARGLRAILENTMLELMYEVPSQKNVEKVIVDIDGLDDYKKVQIIKKEG | K03544 | 145.17 | 508.9 | 1.90E-153 | ATP-dependent Clp protease ATP-binding subunit ClpX | endopeptidase Clp ATP-binding chain C | Stress survival |
| GC_00000231 | MIKKATREAYGEALVELGKINENVVVLDADLSGSTKTSVFKKAFPERHFNVGIAEADLIGTAAGFAACGKTAFASTFAMFAAGRAFEQIRNTVAYPKLNVKIAPTHAGISVGEDGGSHQSVEDIAIMRAIPGMVVLSPADATETKKMVFAAAEYDGPVYIRMGRLGVPVLFDDNYDFQIGIANTLRDGKDVTIAATGLMVAEALKAAEELEKEGISVRVINVGTIKPLDGETILKAAQETKFIVTAEEHSVIGGLGSAVSEFLSEVHPTKVKKVGIYDKFGQSGTGEELLQKYELTAEKLISVIKENL | K00615 | 183.87 | 222.8 | 1.10E-66 | transketolase [EC:2.2.1.1] |  |  |
| GC_00000232 | MKSDERGVVWEAAELTKKKFSVPVSKFVNGVLRSYAREKDEEIEKLKAEGKFDILYSYPLWFFEKVKKDYGDKAEEVLKSLKKIPYMAVRVNKLTYSEEKFEKLLKKNDIKIIKKVDTVYYLDSGAVLYFDEFKEGKIIAQDGASYLSVKVLNPKPDEHVVDTCAAPGSKTVLIAEFMENRGEIMAFDIYPHKIKLIEENCSKMGIDIVKAVKMDARKLKEQGKKFDKILVDAPCSGYGVIRKKPEILYSKGIENVEELSSLQYEILESASSILKDDGELVYSTCTITYEENTGNVKKFLENNPQFETVKFEVPENVNGDFDEFGGFTINYKEEILDNFYIAKFRKKGE | K03500 | 298.03 | 310.9 | 3.40E-93 | 16S rRNA (cytosine967-C5)-methyltransferase [EC:2.1.1.176] |  |  |
| GC_00000233 | MRGDYKMDFKLHSKFKPTGDQPEAIAKISDGIKRGVKDQVLLGVTGSGKTFTIANIIAETGRPALILAPNKTLAAQLYSEYKNFFPENAVEYFVSYYDYYQPEAYIKSTDTYIEKDSAINDEIDRLRYAATSAIINRRDTIIVASVSAIYGLGSPETYKKNSIPIDLKTGYDRGELIEKLVALRYERNDIAFERGKFRIKGDVIDIYPSSHEVGYRLEFFGDDLEAISEINTITGQKIKKNVERIIIPPASHYLTEEDEVDRILGEIKNDMEIEVENFKKEGKLLEAQRLEQRTTYDMEMIKEIGYCKGIENYSRYLSGKKPGERPDTLIDYFPKDFLIFIDESHIGVSQIGGMYNGDRARKTNLVENGFRLKAALDNRPLRFEEFKSIANQIIYVSATPGDYEMEVSGGEVAEQLIRPTGLIDPEIEVRKTENQVDDLLHEIRERAKKKERVLVTTLTKKMAEELTEYYINLGVKVKYMHSDIDTLERIDIIRDLRHGVIDVLVGINLLREGLDIPEVSLVAILEADKEGFLRSRRSLIQTIGRAARNVNGKVILYGDVITKSMRQAIDETERRRKIQREYNAFHNIDPKSVVREVSEEVLNLDYGLPQEEVKQFSSEKKYHSKEEIEKDISKLLKEIKKLSEELDFENAIKKRDEMLKLKNILLEL | K03702 | 309.63 | 1084.5 | 0 | excinuclease ABC subunit B |  |  |
| GC_00000234 | MARQVSLEMTRNIGIMAHIDAGKTTTTERILFYTGVNHNLGEVHDGAATMDWMEQEQERGITITSAATTCFWKNHRINIIDTPGHVDFTVEVERSLRVLDGAVAVFSAVDGVQPQSETVWRQADKYGVPRMAFFNKMDRIGADFKMCVNDIKEKLGANPVPIQLPIGAEDAFEGIIDLIEMKETVYLDDKGQNFEIRDIRPELADEAEAAREFMIESVVECDDELMEKYLGGEEVSNEEVRKALRAGTIGNMIIPVLCGTAFRNRGIQPLLDAITAYMPAPTQKGVIKGTDVKDPEKVIELPIGDDAPFAALAFKVMTDPFVGRLTFFRVYTGIVEKGSYVLNSTKGQKERMGRLLQMHANKREEKEVVYCGDIAAVVGLKNTTTGDTLCDEKNPIILEKMEFPDPVISVAVEPKTKADQEKMGIALAKLAEEDPTFRVKSDEETGQTIISGMGELHLDIIVDRMRREFKVESTVGKPQVAYRETITASTEQEVKYAKQSGGKGQYGHVKIKVEPITGKDFEFVNEITGGAIPREYIPAVEKGCREALENGVVAGYPLVGVKVTLYDGSFHEVDSSEMAFKIAGSMAMKQGAEKCKPVILEPIFKVEVTTPEEYMGDIIGDINSRRGMIGGMTDRNGAKIIDAKVPLSEMFGYATDLRSKSQGRANYSMEFEEYVPVPRAIQDAIKAERGR | K02355 | 535.57 | 1148.3 | 0 | elongation factor G | elongation factor Tu | Adherence |
| GC_00000235 | MLRNKIIIGSRGSILAMAQTELVVKMLKEKFPEMDFEIKVIVTTGDKDMRTNWGDKNVSLKSFFTKEIEKELLEKDIDLAVHSMKDMPAVSPDGLICGAIPVREDNRDVFVSKSGKKLSELPENPIIGTSSLRRAVMVKDMRPDAEIKPIRGNIHTRLKKLEEENFDGIVLAAAGLIRVGLQDKITQFFTKDEVMPAPAQGALCVQCREDDKEILEILEKIADKNTTEVVAVEREFSKIFDGGCHTPMGCAAEIIGDEIEIKGIYYSGEKLYRGTHRGKREKGIILADELAEKIRGNINE | K01749 | 112.97 | 371.3 | 1.00E-111 | hydroxymethylbilane synthase [EC:2.5.1.61] |  |  |
| GC_00000236 | MLKELQIENLAIIEKLDLEFGDGLITLTGETGAGKSIILSGINLLIGEKTNIEMLRDGEKLLVAQGVFSVNEEQEKELSELGIEAEDGEVIVRRTMDSKGKGKAFVNNMRVPVSGLKEIMGTLVDIVGQHSHQMLLNKENHIKLLDKFTEDETKEIRKNLETALDDFVRLDRKIERIDEEKKELREKKEFYEFQLDEIDKVNLAKGEDENLESEYKKLFNAGKIKEKLSMAEACLKNGEVNALSIIYASKKNIESIADYGEEFEENLERLERVYYDLEDCVSSMERLNEDIEVDEGRLEEVISRLDAIGKLKNKYGSTIEEILDYRNEIEKKLKNLDESNFQVKKLVKERENAREKYWNYAFELRDIRKKCIKKIEEQLGEELTYLNMKDAKFEINLEEKDVMTKNGSDGIEFFISTNAGQKPKPLQKIASGGEVSRIMLALKVIFSHVDNIPILIFDEIDTGVGEKL | K03631 | 148.37 | 501.9 | 3.40E-151 | DNA repair protein RecN (Recombination protein N) | DNA repair protein RecN | Stress survival |
| GC_00000237 | MNKLSLDLSNVSSFVSEEKLMGMEAEVKAAVKTLEEGTGAGNDFLGWINLPTDYDKEEFDRIKKAAEKIKSDSDVLVVVGIGGSYLGARAAIELLSHTFYNKLSKEDRKAPEVYFAGNSISGTYLAHLIQVIGDRDFSVNVISKSGTTTEPAIAFRIFKEMLEKKYGVEGARKRIYATTDAAKGALKKLSTEEGYETFTIPDNVGGRFSVLTPVGLLPIAAAGISIDDLMAGAREAQNDYKAEFKNNDCYKYAAVRNLLLRDGKAIELLINYEPKVHFVAEWWKQLFGESEGKDGKGLFPASVDLSTDLHSMGQYIQDGQRILFETLIDVVSPDADVVIPFDEADLDGLNYIAGKGMNFVNQKAMEGTQLAHVDGGVPNIRIAVPKMDAFNLGYLFYFFEKACGVSGYLLDVNPFNQPGVEAYKKNMFALLGKAGYEKEAEVLNKRLKK | K01810 | 174.13 | 259.3 | 1.00E-77 | glucose-6-phosphate isomerase [EC:5.3.1.9] | glucose-6-phosphate isomerase | Immune Modulation |
| GC_00000238 | MRINLDFNIKDDTIHEFKKVMLPDVEIETLNVKTLEENSDIISLELSSGENRFVFTLKNYSDKMVDQKIVMIKAGLLKLFKKVYPWGALMGVRPTKLVRRFLIMGYSYEEIDEILEKLYFAFPEKRKLLIDVVKKKAFTLMIKL |  |  |  |  |  |  |  |
| GC_00000239 | MLDDTPMMKQYNEIKADYEDSILFYRMGDFYEMFYEDAKTASRELGLTLTKRNKKTDVPLAGIPYHSAASYIAKLVGKGYKVAICEQVEDPKTAKGIVKRDVIRVITPGTIIDTEYLDEKVNNYLLGVLADDKKIALAYVDITTGEFRASEITGGNLDYKLLGEINKISPKEIMTDDKGYEFLSKELESHRMKDGISLHRGTVVRNSEKYLAEYFGVQSLESFGIGDKELVIKTAGMVLKYVVELQKGKELPVTKIVYSSSKEVMELNLTTQKNLDIISNWRGESTNGTLLWVLDYCKSSMGSRLLKKIIKNPLLNIEKIKKRQEHIEFFINEVLLREEIREKLKDIYDIERIIGKLILETINGRDLTALKQSIRNSLELYKMLQGHELFKIDAEKLIEVYNMIEKIIVEDPPFSIREGGIIKDGYNEELDELRNISNHGKDYILKIESDEREKTGIKGLKVKYNKVFGYFIEITKANLENVPDYYIRKQTLTNAERFIVPELKTYEEKVLNAKTRIETLEYYLFKELTEEIKKYRETLQDLGYKIAYLDVVSNLAHVAIKNGYVKPEITDDSSLEILGGRHPIIEKLIPAGEFVKNKVILDDRKNFIILTGPNMSGKSTYMKQTALIIIMAHMGSYVPADYAKIGLVDRIFTRIGASDDLMTGQSTFMLEMSEVANILNSATEKSFIILDEIGRGTSTFDGISIATAISEYIHDNIRAKTIFATHYHELTQLGDKLDRAENYRIEVEENDKDIKFLREIVKGGADKSYGIEVARLAGLPKEILVNSKKILKVLEERKNIIEKKFGGEQMMLFAPSTQEEVKKEEIPKEEEKNISKEEELTLRLLRELDINTLTPMDAMIKLNELKKLLN | K03555 | 657.87 | 1098.9 | 0 | DNA mismatch repair protein MutS | Dot/Icm type IV secretion system effector SidH | T4SS Effector Delivery System |
| GC_00000240 | MAVKVAINGFGRIGRLALRLMIENPEFDVVAINDLTDAQTLAHLFKYDSAQGRFNGTIEVVDGGFVVNGHEIKVCAQASPKDLPWGELNVDVVLECTGFFTKKEKAEEHIAAGAKKVVISAPATGDLKTIVYNVNDNILDGTETVISGASCTTNCLAPMAKALQDNFGIVEGLMTTIHAYTNDQNTLDAPHKKGDLRRARAAAANIVPNTTGAAKAIGLVIPELKGKLDGAAQRVPVITGSITELVTVLEKPVTVEEVNAAMKAASTESFGYTEEPLVSSDIIGINYGSLFDATQTKVMTVGDKQLVKTVAWYDNEMSYTSQLIRTLKKFVELAK | K00134 | 423.23 | 484.7 | 6.20E-146 | glyceraldehyde 3-phosphate dehydrogenase (phosphorylating) [EC:1.2.1.12] |  |  |
| GC_00000241 | MEENITELASVINNPKTQIFFDSLIDSIAKILPKIAIRSLWVIFILIIMKPTVNMIVKTLRALLHKSKADPLLESFLASLVKTVVYIGFFFLIISGIGVQATSLVAILGTAGLAVGLALQGSLANLAGGVLILFFKPFLKGEFITASAGSGTVTAIHILYTTLTTPDNKRIIIPNGQLANGAVTNISREPERRVDLVISTSYDTPVEKAKAVLKRIAEENEKVLHEKGYTIRLNAHSASSLDYIFRVWAKTPDYWEVYFGLMETVIIEFNKEGIEIPYQKIDIYNK | K03442 | 235.27 | 262.9 | 5.20E-79 | small conductance mechanosensitive channel |  |  |
| GC_00000243 | MKINILTLFPELFSAFKSQSIIGRAVKNNLIEINIINIRDFCFDKHQQADDTPFGGEGGMVMKPEPLFRALENCGGKVIYTSPQGVKFDQQLALKLKEEEEITIIAGHYEGIDERVVENKVDMEISIGDFVLTGGELPSMVMVDAIARLIPGVITQASYENDSFFNGLLDYPQYTRPAEYMGLKVPDVLISGNHKKIRDWRMKESLKRTYLRRPDLLKDREFSKEEKKFMKEILEELMEGEK | K00554 | 109 | 374.3 | 8.70E-113 | tRNA (guanine37-N1)-methyltransferase [EC:2.1.1.228] |  |  |
| GC_00000244 | MITKVKSSGYFGIESFSVDVEVDISKGLPAFNIVGLGDTAINESRERIKAGIRNSGYKLEPKRITVNLTPANIKKVGTHFDLPTAVGIMEGYGLIKSRKEILENYIFMGEVSLAGEVKRTQGIVNGAILAKENNCRGIVIPYDNLREGSLIKGIEIVAVKNLKEAVEFLENGNYKKYDVPEEKEIFNEEIDFYDVKGQEKAKRALEICAAGGHNLLMAGTPGSGKTMLAKRITTILPPMTDEEKIEVTKLYSISGKLSETNPVINKRPFRSPHHTSSSTAVIGGGKTPSLGEISLADKGVLFLDEIVEFKKDVLEALREPLEERKVSVTRAVYKAEFPADFIFIGACNPCKCGYAFEDGDLCTCSAGEISRYMKKLSGPILDRIDLKIEIKRLTEDELTGDMPRETSEEIRKRVIRAREIQKKRFGCEKLNGHMTRKDLEKFCRLDEETKAIMKTAVKNLNLSARSFDRVLKAARTISDLAGSRDIKKIHIMEALSYRISEFNI | K07391 | 87.6 | 635.3 | 1.00E-191 | magnesium chelatase family protein | two-component response regulator AlgB | Biofilm |
| GC_00000245 | MMFDTIAAISTPRGEGGIGIVRISGKEALDILCEIFKPKSSRNVSELKSYTITYGHIYDGDKLIDEVLVSFMKGPRTYTREDIVEINCHGGYIITERVLELVLKKGAKLAEPGEFTRRAFLNGRLDLTQAESVMDIIHGKTEKSISLSLNQLRGDLKEQIGVLKKLLLDVAAHVNVVLDYPEEGIDDPLPKNLRDNLDTVIATTTKLIASYDKGKMIKEGVKTAIVGKPNVGKSSLLNSVLREERAIVTRIAGTTRDTIEEIVNIKGIPLVMIDTAGIRETEDEVENIGVQKSKSLIKNADLVLFVLDASRELDKEDFEIYERIESDKVIGILNKIDIERKIDIKNLTKVKKWIEISALEKIGIEDLEDEIYEYVVSGQVEDSSEKLVITNVRHKSALEKTKKAVENILETIDMGYPMDLIAVDLNEALDSLSEVTGEISNEDLLDHIFSNFCVGK | K03650 | 172.5 | 533.5 | 1.10E-160 | tRNA modification GTPase [EC:3.6.-.-] | Fe(2+) transporter permease subunit FeoB | Nutritional/Metabolic Factor |
| GC_00000246 | MRAVVQRVSSAEVKIDGNSVGKIDKGFLVLLGVTHSDTEKDVEWLANKIKGLRVFEDAEGKMNLGLDDIKGDILIVSQFTLYGNCLKGKRPSFIEAARPETAVPLYEKFIETFKGFGLNKVEHGVFGADMKVSLTNDGPVTMIIDTPEKK | K07560 | 31.7 | 218.6 | 2.00E-65 | D-aminoacyl-tRNA deacylase [EC:3.1.1.96] |  |  |
| GC_00000247 | MYLTVKQQLKHLSKDEFLSLRELSHTAKNLYNQAVYNVRQFYFQEKKYLNYQNNYAVLKTSENYKLLNSNMAQQILKEVDGSFKSFFGLLKLAKKGKYNFKDIKLPNYLQKNGFTTLVIGFVRINKNTLIIPYSNSFSKNHKKISIKIPPILLDKKIKEIRIIPKFKARFFEVQYTYEVQEQQRNLDKNNALAIDFGINNLAACVTSKGKSFIIDGKKLKSINQWFNKENARLQSIKDKQKYGKKPTLRQKYLYASRNNKVNDYMSKTARKIIGYCLKNNIGTLVCGYNETFQRNSNIGKANNQTFVNIPFGKLREKLEYLCKLYGLIFVEQEESYTSKSSFFDMDILPKFEADNSQNHSFLGKRIKRGLYQTSKGYIFNADVNGALNILRKSNVVDLEVLYTRGAVDTPERIRIA | K07496 | 46.63 | 151.1 | 6.20E-45 | putative transposase |  |  |
| GC_00000248 | MDEKIYDLIVIGGGPAGLTAGIYAGRAVLDVLVIEKDRAGGQINLTNEIVNYPGIIETSGEKFGEELKKQALGFGVNFLNDEVIGMDLSKDIKTIKTAKEEFKTLSVVIATGASPRKLGFKGEKEFTGRGVAYCATCDGEFFTDLDVFVIGAGFAAAEEAIFLTKYAKKVTVIAREPEFTCVKSIAEKVLKNPKIEVKFNTEILEADGETQLKRAVFKNNVTNEIFEYNAPEGKTFGIFVFVGYEPQSRLFEGHVALDNYGYVPTDSDLMTNIPGVYAVGDVRPKKLKQVVTAVADGADAAMNIEKYVLGIREKLGLKKEKNREVKKSQEVSEKNGEFLDSSLKEQLGAVAEKFENEIEIVLIKDSSNEKSFEMETAFKDIASVSEKIKFSSYEKGENKELEQKINAERFPTSAVLDKDGNFSGIKYSAVPGGHELNSFILAMYNVAGPGQKLSDETLEKIRNIKRSINVKIGISLSCSNCPETVQSVQRIAAENKNIQVEIIDVLTFRDFKDKHEIMSVPAMVVNDRGIFFGRKNIDEVLHFL | K00384 | 334.77 | 385.4 | 5.60E-116 | thioredoxin reductase (NADPH) [EC:1.8.1.9] |  |  |
| GC_00000249 | MKKLSVLALAAVMSASAFAAKVGVVNTQELFYKYSKTKVIDQNLKKQGASLENTINQKQVELKKLQLELQSKGEKITDAEKKSFEDKVKALDKFVRDSQMKMDKERNARLQEVEKTMTNAINKVAKADKYDYVLEAGAVKFGGTDITAKVLQEMEKTK | K06142 | 35.63 | 102 | 4.60E-30 | outer membrane protein |  |  |
| GC_00000250 | MEKNIYSNPLVERYSSKEMLEIFSPKNKFSTWRKLWVALAESEKELGLNITDEQIKDMKEHIYDIDFEMAKKREKEVRHDVMAHVYTFGMQAKSAMPIIHLGATSAYVGDNTDLIQIKEGLALLRKEIVNVMDGLSKFAMEYKGMPTLGFTHFQAAQLTTVGKRATLWLQSLLLDFEELEFRDKTLRFRGVKGTTGTQASFEELFDGNFEKVKTLDEMVTEKAGFKKRFMVAGQTYDRKIDSEIMNLLSNIAQSAHKFTNDLRLLQHLKEVEEPFEKNQIGSSAMAYKRNPMRSERISSLAKFVIALQQSTAMTASTQWFERTLDDSANKRLSLPQAFLAVDAILIIWKNILDGLVVYPKMIEKHIMAELPFMATEYIIMEGVKNGGDRQELHEKIRVHSMEAGKMVKVHGLENDLIERIVNDPSFNIDKEKLKEILSPKNFIGFAEKQTEEFIEIEIKPILDKYSELIGMNADLKV | K01756 | 296.63 | 588 | 3.60E-177 | adenylosuccinate lyase [EC:4.3.2.2] |  |  |
| GC_00000251 | MAKIIGIDLGTTNSCVAVMEGGSASIITNAEGARTTPSVVNIKDNGEIIVGEIAKRQAITNPTSTVLSIKTHMGSDYKVHINGKDYTPQEISAMILKKLKKDAEAYLGEEVKEAVITVPAYFTDAQRQATKDAGAIAGLEVKRIINEPTAAALAYGLDKNKDEKVLVFDLGGGTFDVSILEIADGVIEVLATAGNNHLGGDDFDAKIIDWLVSEFKKEQGIDLSNDKMAYQRLKDAAEKAKKELSTMMEAQISLPFITMDATGPKHLEMKLTRAKFNDLTRDLVEATQGPTRTALSDAGLSPADIDEVLLVGGSTRMLSVQEWVESYFGKKPNKGINPDEVVAAGAAIQGGVLMGDVKDVLLLDVTPLSLGIETLGGVCTRIIEKNTTIPVKKSQVFSTAVDNQPAVTINVLQGERAKAADNHKLGEFNLEGIPAAPRGIPQIEVTFDIDANGIVHVSAKDLGTGKENTVTISGSTNLSSEEIERMKKDAEANEAEDKKFKELVETRNKADMLIASTENTLKDHSGKVTEEERKAIEAALEELKQVKDKEDKAAIEAAIEKLSKVAQKLAEEVYKEAQAKQQAGAANGQAGAENAKKDDDIEDAEIVD | K04043 | 810.03 | 924.7 | 8.40E-279 | molecular chaperone DnaK |  |  |
| GC_00000252 | MKVLVINCGSSSLKYQLLNPETKELFAIGLCERIGIEGSKMEYEVPAKDFEITVTEPMPTHKEALALVINAITDKEHGVIASVDEIDAIGHRVVHGGEDFVKSVLITEEVIKGVEANNELAPLHNPANLIGIRTCQELMPGKPNVGVFDTAFHQTMPAEAFMYALPYEDYTELKVRKYGFHGTSHKFVSQECLRELGNPEHSKIIVCHLGNGASISAVKDGKCIDTSMGLTPLQGVMMGTRCGDIDPAAVLFIKGKRGLTDKEMDNRLNKQSGILGIFGKSSDCRDMENGVAEGDERAILAEKMFIYKIKAYVGNYAAQLGGVDAICFTGGIGENAAHIREAVVEGLEFMGIKINKEVNAVRKKGIVDLTAADSKTKIFKIPTNEELAIARDTYEIVSKK | K00925 | 127.53 | 585.5 | 2.00E-176 | acetate kinase [EC:2.7.2.1] |  |  |
| GC_00000253 | MENKMFCYQCQETAGCIGCTMSGVCGKTPDTAYLQDFLIYITKGISEIAVRLREEGQKISDETDFMVVENLFTTITNVNFSENDLKDKIEKSLKIKNELLNLLADKENLSNAALIYIEREDYLIKIEDVCIPAEKDEDKRSLKEMITYGLKGLSAYLKHAEVLKFTDKNINFFIEKTLAELLRKDITAEELTALALEVGKYGVLGMELLDRANTKTYGNPEITKVNIGVGKNPGILISGHDLKDLETLLEQTQGTGVDVYTHSEMLPAHYYPKFKKYKNFAGNYGNAWWKQQEEFESFNGPIIMTTNCIVPPKPSYKNRVFTTGLVAFDGCRHISGEKKDFSEVIETAKKSLPPKEIEQGEIIGGFAHNQVFQLLDKVVEEVKKGNIKKFVVMGGCDGRSKKREYYTEFAKNLPKDTVILTAGCAKYRYNKLNLGDINGIPRVLDAGQCNDSYSLALIALKLKEVFGLDDINKLPIIYNIAWYEQKAVIVLLSLLYLGIKNIHLGPTLPAFLSPNVAKVLAENFRIGKITEVSNDLKLFGM | K05601 | 139.7 | 867.1 | 8.50E-262 | hydroxylamine reductase [EC:1.7.99.1] |  |  |
| GC_00000254 | MSLSKKTQIFYGMGVSYAIADQIFAQWILYFYLPPESSGLKPVMAPILISIALAISRIVDMITDPAVGYISDKVNTRWGRRIPFIAAGSIPLGLATVAFFYPPMGNNTITFIYLAVVGSLFFTFYTIVGAPYNALIPEIGNTMEERLDLSTWQSVFRLLYTAAAMIIPGVLIKIIGEGDTVFGIRGMVIILSIAASLGAYITVFGVSEKNYSKGETSKISFKETVKIITSDKSFIYYLFGLLFFFVGFNTLRATMNYYVEDIMGYGKSQITIASAILFGVSAMFFYPTNKLSKKIGYRKVMLGCLALLTIFTVMLFLLGKIIPVSFGFILFGLIGIPVAGGAFIFPPAMLSEISSRITERTGNKIEGICFGIQGFFLKMAFMLSILILPFILVLGGTAGAVTKYGIYGTALFSAVSFIISFFFYYKYKEK |  |  |  |  |  |  |  |
| GC_00000255 | MAKLIKFDEEARKKLEKGVNTLADAVKITLGPKGRNVILEKSYGSPLITNDGVSIAREIELEDPFENLGAKLIKEVATKANDVAGDGTTTATILAQNIVKEGLKVVSAGANPIFIKKGIDKAAKEVVRNLKIKAKKVQSNEEIEQVASISAGDEEIGKLIAEAMAKVGETGVITVEEAKSLETTLEVVEGMQFDKGYISPHMVTDPVRMEAELENPYVLITDKKISSMKDILPLLEKIVQESKPLLIIADDLEGEALTTLVINNLRGTLNVVAVKAPAFGDRRKAILEDIAVLTGGKVVSEEMGMKLEEAGLEVLGTAKKIKITKDSTTIVDGGGEAQAISERIERIKAQISESTSSYDAEKLQERAAKLSGGVAVIKVGAATETEMKDKKLRIEDALNATKAAVEEGIVPGGGVAFVEILKSMEDFKLQGEEGMGVEIVKKALMSPLKQIAVNAGLDGGVVAEKVKTLPDGFGLDAAKEEYVDMMANGIIDPAKVTRSAIQNAASIASLILTTEVVVAEKKEPAPMPSGNPNMMM | K04077 | 145.97 | 838.9 | 3.30E-253 | chaperonin GroEL [EC:5.6.1.7] | chaperonin GroEL | Adherence |
| GC_00000256 | MNIEKDLQKLKKNLNERKKSLTLDEIYSLLGWSPKFKKENREIVNSWVEAGEIVKNNKGRYNVPENVGIVKGTFSVVKNKFAFVDTEEEGFFIPRRDFNGALNGDTVLARIRESSFKDGKREGEVVKIIKRENDTVVGILSKRKDFGFVVPTHSFGQDIYIPKKYLKMAEDGDLVLVKIDFWGDKDRKPEGKIVEKLGNPLDSDVMIEALIKRTGLNPEFPDEVLSEARNIKTEISKEEYEGRKDLRNLSIITIDGADAKDLDDAVYVEKLENGNFRLIVSIADVSHYVKEGTALDKEALKRGNSVYLVDRVLPMLPKEISNGICSLNPNEDKLTFTCDMEITPEGKVVSADTYKSVIKTAFRMTYDNVNKIFEGDEEVTKQYESIKDMCFNMLELSKIIREVKYKRGSIDFDLPEIQVVLDENKKVKYLRRRDRGEAEKVIEDFMIEANEAVAEKLFWLEIPSIYRTHEKPDSERIEALNDVLGKFGYRIHSAEGIHPKKFQTIIEDSKDKGLSLIVHKMVLMALKQARYTPENVGHFGLASSYYTHFTSPIRRYSDLMIHRILGSVLHGYPKEKYIKKLEEILGGICLAISKTERDAMKAEEESVKIKVVEYMLDKVGEVFKGTITGFANRKVFIETDELVECMWDVTNSPHYYEFDEVNYVMADKDSGEIYNLGDKLDVIVVRADMAELEVEVAPYTEEFITGLRK | K12573 | 479.63 | 812.1 | 7.60E-245 | ribonuclease R [EC:3.1.13.1] |  |  |
| GC_00000258 | MEFLLQIINGLQIGSIYALISLGYTMVYGIAQLINFAHGDIIMVGAYISLFSIPAFTRMGLPVWLTMIPAVIVCVLLGMFTERVAYRPLRNSPRISNLITAIGVSLFLENLFMKLFTPNTRPFPKVFTQPPITFAGLHLNFGTVVTIIVTIILSVGLQYFMKKTKYGKAMLATSEDYGAARLVGINVDHTIQLTFAIGSGLAAVAAVLYVASYPQVQPLMGSMPGIKAFIAAVLGGIGILPGAVLGGFILGIVESLTRAYLSSQLADAFVFAILIIVLLVKPTGILGKNVREKV | K01997 | 224.43 | 346.1 | 4.00E-104 | branched-chain amino acid transport system permease protein |  |  |
| GC_00000259 | MKRVVVTGLGLITALGTGLEKSWAGIISGKTGIKTIESFDTEDTPVKVAGEVRDFQPEEFGIEKKEIKKLARNTQFAIGAAKMALSDSGLTIDENNAERVGVIISSGIGGMEIFEAQHEVLLNKGPKRLSPFTIPGMIANMASGNVGIYLGAKGPNKSIVTACAAGTHSIGDAFETIKLGKADVMFAGGTEACITKFAVNSFANMKALSTNPDPNTASRPFTIDRDGFVMGEGAGVLVLEELEHALARNAKIYAEVVGYGETCDAHHITAPAVDGAVRAFKMALEESGASVSEVDYINAHGTSTGLNDKNETAAIKEVFGEDARRLNISSTKGATGHVLGGAGGIEGVILAKSIADSIIPPTANYENPDPECDLNYTPNKAEEKEIRVGMSSSLGFGGHNAVIVMKKYK | K09458 | 471.3 | 555.2 | 2.30E-167 | 3-oxoacyl-[acyl-carrier-protein] synthase II [EC:2.3.1.179] | capsular polysaccharide biosynthesis fatty acid synthase | Immune Modulation |
| GC_00000260 | MCKTTVRNLYRNEKEFLGKEIEVSGWVRKLRDQKNFGFIELNDGSFFKGMQVVYDTSLANFEEISHLSISSSITVKGIFKESEGKGQATEIKATDVKIIQKASLDYPLQNKRQSFEYLREIAHLRPRTNTFAAVFRVRSVLAYAIHKFFQEQGFVYTHTPIITGSDAEGAGEMFRVTTLDFSNLPKKEDGTVDNSKDFFGKSTNLTVSGQLNGETYCSAFRNIYTFGPTFRAENSNTSRHAAEFWMIEPEIAFADLNANMDLGEAMIKYIIKYVMDECPEEMAFFNQFIEKGLIDKLNNVLHNEFARVTYTEAIDILLASGQKFSYPVKWGIDLQSEHERFLAEKHFGKPVFLTDYPKDIKAFYMKLNEDGKTVRAMDLLAPGIGEIIGGSQREDNLEALEKRMDEVGLKKEDYSFYLDLRRFGSFPHSGYGLGFERMMMYITGMTNIRDVIPFPRTPGNAAF | K01893 | 369.7 | 697.5 | 1.70E-210 | asparaginyl-tRNA synthetase [EC:6.1.1.22] |  |  |
| GC_00000261 | MEIKVNVLNAMRLTKLLIAASRWLSRHADILNDLNVYPVPDGDTGTNMSMTLQSVENQLIKLSYEPDMEELCDTVSEAILLGARGNSGTILSQIIQGFLDGIRDKEEATVEDTIAAFELAKEKAYKAVSNPVEGTMLTVIRKVSEGAKAYTGDKSDFVPFLIHLKNVAAEAVEETPKLLAKLKEAGVVDAGGKGIFYILEGFEKSISDPQMLEDLERIVQSQAKRQERLDANAMTMEEIKFKYCTEFIIENGKFDLDSYKNEIGVLGDSMVCAQTSKKTKTHIHTNNPGLVLEIAGKLGNLSNIKIDNMELQQHKNMMFSDDAYKAEDSKNKILVRNENSRPVAYFAIADNKEIGELFLNSGATAVLLGGQTQNPSVADIEEGLKKIEAQTIVILPNNKNIISSAKIAAERSNKDVTVLETTSMLGGHYIVKNKDLNMEKIAKNLNRNSSVEITRAVRNTKVDNLEISEGDYIAMVNGRIREKASSLKELVEIIKENYITEYTLNVFVSIGKDADKEATAVLREIGGEKSYTDIEGNQNNYPYYIYIENRDPKLAEIAIVTDSTSDLTPELIKDLDVEIVPLKIKLDGNNYYREGVDISKREFWRKLLTEGQLPKTSQPSPAEFKAMYEKLFAKGYKKIISIHISGKLSGTQQAARVGKGMTAREGDIAIIDSKTTTFALGHIVTEAAKLAKERKSFKEIIDWIEETKESMKVFFVVKDLEFLQKGGRIGRASAMVGGLLKLKPVLKVENGEVSVEAKAIGERGAMLHMEKLIKNSKNSIILYTAWGGTQSELANADALRNAAEKMKRVDYRGRVEIGATLGSHTGPVYGMGVMDKIR | K07030 | 307.77 | 755.2 | 1.30E-227 | fatty acid kinase [EC:2.7.2.18] | adhesin HMW2A, two-partner secretion (TPS) pathway TpsA exoprotein | Adherence |
| GC_00000262 | MKKFKLFGFKFIFKMEKENKDDVQIYSESYNLKEKVIYILLIMALLSFSAKFHLFFNENSYKKGSIVKEDIYAPRTVKYNDVAKREAIIEELILTSKKEYIHVPEVEVSHYNGMKNFYDQIIALKKGAKIDFNYKIIEESLEKEVNKNLVKEISGLSTKKITAQRDKLLKLLNEIYESRVVKEDGIINIKDEQVEKIEKLSPLEQKVLHTFIVPNYVFDKEKTKDEIEKKTSKIGEQIVEIQAGSIVAKKGEVLTDRKMELLSALGIYSYSETIARLVMNIMYLFIISFISYQTLYRTFKNEILKKNTYRAVLILIAGAFFILRFLSNEYMFIVPFEVMFFMFGILVNPSFAFAMTIISLGFLMPILDYNLIYFFIYLFAVLLGTILLKDIKTRSQLINLGVQLSIVKFILFFILSYFTGVMNVSVVVKAGQIIIAGILSGMVSIALVPYFERTFNILTRFKLLELGDLSHPLLRDLSVKTPGTFYHSMMVATLSEAAADAIGADSIFTRVASYYHDIGKMKRPKFYVENQEGGVNPHNSISPFLSALIIAAHTKEGAELGKEYQIPKEIRDIMFEHQGTTLLAYFYNKAKQIDENVQEEDFRYPGPKPKTKESAIIMLADSIEAAVRSLDEKTPVTIEAMIRKIIAGKMGDGQLSDADLTFKEIEIIIKVFTKTLMGIHHVRIKYPGQK | K07037 | 227.4 | 631.2 | 3.10E-190 | cyclic-di-AMP phosphodiesterase PgpH [EC:3.1.4.-] |  |  |
| GC_00000263 | MKREALKLLKQIYGYDSFRKGQNHIISSVLNRRDTLGVMSTGGGKSICYQIPALIFPGITLVISPLISLMKDQVDALKILGVNSLYINSTLTREEYISALRKIKSRKVKIVYLAPERIVNEKFINFMKDVDISLIAVDEAHCISQWGHDFRKSYLEIPKFIKAMGKNIQILALTATATPRVRQDIEKLLNMNRCYHYVDGFDRENIFFKVERGVVPEAYIVRYLKEHKNKSGIIYASTRKEVDNLYAYLSMKGFKVGKYHAGLTEKERTDFQEKFLKDDIEIMVATNAFGMGIDKSNVRFVIHRNIPKDLESYYQEAGRAGRDGVPSEAVLLFFEEDVATQEFFIINNEETSDKIRQIKRKKLDKMVEYAYLDTCYREFILKYFGDKRIKNYCGNCGNCRNLKDVENLTTETKMVISCIGRAKENIGISTLVNILLGKSDTKIERKGYRELSTFGIMEEKDREWLEEFVNFLISEEYLDLSAGSFPTVQLNKNSFSVLKDKKRILRRPNETVSFDYYEDPLFENLNKLRKEISQEENVAPYIIFSDLTLIELAEKKPKNRWEMLKIRGIGNQKFKNYGERFLKVINSFSDDDMEILKVENFIDENYLSEEKLLNLKKSLDTNIDIIKLKEILIKNLFS | K03654 | 625.97 | 689.2 | 1.20E-207 | ATP-dependent DNA helicase RecQ [EC:5.6.2.4] |  |  |
| GC_00000264 | MSKKDYYDLLGVEKTASENDIKKAYRKLAMKYHPDKFSNASEKEKKEAEEKFKEVNEAYQVLSDPDKRAKYDRFGHAAFENGGGGAGGFGGFGGFEGFGNAEDIFSSFFGGGGGFGGFGGGRQRGPEPGADLRVDVTLTLEEVAKGVEKEINYRRQAKCKTCNGTGAEPGSNLKTCDKCNGTGKIKVTQRTVFGNFQSVEECDKCKGKGKIPEKKCKSCNGTGLERETVKKTVKIPIGIEDGQRLRLSGMGDASTEGGPNGDLYIFIHVKEHDFFVRHGDDIICEIPITFAKAALGGEIDIPTLKGKKSIKIPAGTQNGKVFRLKGEGINNPRGYVPGDQLVKIIIEVPTNLTDDQQELLRKFDESLKDKNYKMNKSFIERFKDFFRG | K03686 | 398.1 | 474 | 1.20E-142 | molecular chaperone DnaJ |  |  |
| GC_00000266 | MKNILIGVCGGIAAYKTANIISKLKKKGHNVKVIMTENATKIITPLTLETLSRNKVVVDMWDINRGYEVEHISLADWADVVLIAPATYNIVGKVANGIADDMLSTVISACTKPKYFALAMNVNMYENPILRDNIEKLKKYENYYFIEADEGFLACNVNAKGRLKNEDDIVEILEESFTETEKTLLGKKF |  |  |  |  |  |  |  |
| GC_00000267 | MAKDLLKEGSPEDIVASLLKSAYEDVLDTSNYNEIEEATVDHTGKTRLFIALGRVDKITPKKLVEFIVKESSIDKNKIKGVEVYENFSFMSVPFAEAEVILDIFKKVRKGRKPLVEKAKEKSKSESSETETKEKSSGRKRAGRKTETKEKTAEKPKRTRAKKETKKEDK |  |  |  |  |  |  |  |
| GC_00000268 | MKKNSILILDFGSQYNQLIARRIREMGVFAEIVPYFEPLEKIVEREPKGIILSGGPASVYLEDAFMIDKKIYDLGIPVLAVCYGMQLTTHLLGGKVECADKQEFGKAQLILDDAANPLFEGIPNNSQVWMSHQDHVTQLAPGFVQIAHTDSCIATACNPEKNIYCVQFHPEVTHTEYGKELYRNFVFNIAKCEKNWSMSNYIEDTIEKIRKTVGDKKVLLGLSGGVDSSVAATLIHKAIGDQLTCIFVDTGLLRKDEALNVMKIYGENFHMNIKCVNAEARFLFKLAGVSDPEAKRKIIGKEFIEVFNEEAGKLTDVDFLAQGTIYPDVIESQSVKGPSMTIKSHHNVGGLPEDMKFTLLEPLRELFKDEVRAVGRELGIPDHMIDRHPFPGPGLGIRILGEVTKEKADILREADNIFIEELRNADLYNKVSQAFVVLLPVKSVGVMGDVRTYEYTAVLRSANTIDFMTATWSRLPYEFLEKVSNRIINEVKGINRLTYDISSKPPATIEWE | K01951 | 100.4 | 759.9 | 2.20E-229 | GMP synthase (glutamine-hydrolysing) [EC:6.3.5.2] | carbamoyl phosphate synthase small subunit | Nutritional/Metabolic Factor |
| GC_00000269 | MNNGGGIVVEIHGGNIYKLQREGKKNILDYSSNINPLGVPKSLKKAVSENFSVLTRYPDINYTELRESIGKYNNISPENIIAGNGATEVLFLYIKALKPKKVLIAAPTFAEYERAVKNAECEIDFFELKEEDNFTLNKDKFIQKAVNYNLAVICNPNNPTGKFISKEIIFEINENLKKSGTKLFIDECFIEFISGWEEKTSALFKSENIFILRALTKFFALPGLRLGYGICFDKKITEKINEIREPWSVNGFADLAGKVILNDVEYIKNSEKWILEERKYFTDSLKEIEKSGRIKVYDTEINFILIKLFEMTSEEFKSRMEEKNILVRNASNFKFLDNSFVRLAIKDREKNQKVVEAVKEVLL | K04720 | 313.23 | 428.9 | 2.40E-129 | threonine-phosphate decarboxylase [EC:4.1.1.81] | aminotransferase | Immune Modulation |
| GC_00000270 | MNKRIFVSKKQGFQVEGNSILNEIRENLYETGLTGAELYNVYDVFNCDEEDVKLLKTKVLSEIVTDNVYDEIDLTGKTYVAIENLPGQYDQRADSAEQCLALLNSKDSVTIKSGRIIVFYGEVKDLEKIKKYLINPVETREKDLSILENNEDVTVEPVPVLDGFRKLSREELENFVSVNGLAMTADDLEHIQKYFVEEDRDPTETEIKVLDTYWSDHCRHTTFETFLKSVVIEKGEMTEAIQRAYEKYLELRTTVHGNKKPMTLMDMGTLGGKYMRKIGKLDDMEITDEINACSIEVDIDVDGETERWLLMFKNETHNHPTEIEPFGGASTCIGGAIRDPLSGRAYVYQAMRITGAGDICEKLEDTLPNKLPQVRISKGAAHGYASYGNQIGLAATFVREIYHNGYKAKRMEVGAVVGAVKKEYVLREEPKPGDLVIVLGGRTGRDGVGGATGSSKEHNDSSLTKCSSEVQKGNAPIERKIQRAFRNPEVTKLIKKSNDFGAGGVSVAIGELARGIEVNLDMVSVKYLGLSGTELAISESQERMAVVIAPENKEKFEQLMKEENLETRLVAKVTEKENLVIKFRGETIVNIARSFLDTNGVRQEQDVKVAAPVKQEMFKSEKTGTVEEKLTATLKDMNVASQRGMIEMFDSTVGRSTVLMPFGGKYQLTESEASVQKFPTCGFTNTASVMTYGFNPYISEKSPYLGAIYAVIESLARLTSVGADYKKARLTFQEYFERLGKEPTRWGKPFMALLGALEAQVEFETPAIGGKDSMSGTFKNIDVPPTLISFAVTTEKTQNIVSSEFKTAGNYIYLVKPEYNSDFTPKYDTVKAVFDNVRKEMLNGNIVSASVVKFGGAAEALMKMAFGNKLGVNIETTEELFDLMPGALVVESREKLSFGFLLGTVEDTKEIRINGAVINIDKAISDWEERYAKIYPYDTGAKAEKVFMPTEKKGNMKQKASVLYDKPKVLVLAFPGTNSEYDTAKAFERAGGEAEIFVINNLTVKDMEVSIEELSRKIKESQIVMLPGGFSAGDEPDGSGKFITNILQNPKVKSAIKEFLAKEGLMLGICNGFQALIKSGLLPYGDMDKLNENSPTLFRNDINRHVSRIATTRIASNSSPWLSSFEIGDMHSMTLSHGEGKFVVSEEFAKELFENGQVATQYCNLEGEPTMDGKYNLNGSCYAVEGITSKDGHIFGKMGHSERYEDGIFKNIYGEKMQDIFQNGVNFFKK | K01952 | 635.93 | 870.1 | 4.60E-262 | phosphoribosylformylglycinamidine synthase [EC:6.3.5.3] |  |  |
| GC_00000271 | MKIIRDILKGNEKLKNSYVALGTFDGVHRGHRVLISEAVKKAKENNGVSVVFTFSNHPMEVIFPERVPKMINTLPEKLHLLEEMGVDYVVLQTFDREFAKCDREKFIDDILIDALGVKEIFVGFNYTFGERGAGNTEYLKNICCEKGFAVNEVPPVEYKGYVLSSTLIRNLILKGELELANCFLGRPFLISEIVEHGKKYGRLLGFPTANLKVVNKVYPPFGIFGGSTVIEGRKEKYSSVVNIGKNPTLKPGELSVEVHILDFSEDLYGKRIFVDIEHFMRPEKKFDSFEDLKHGIQKDVENWRKFSDGK | K11753 | 131.9 | 354.5 | 7.20E-107 | riboflavin kinase / FMN adenylyltransferase [EC:2.7.1.26 2.7.7.2] |  |  |
| GC_00000272 | MLILGIESSCDETSIAVVRDGKEILSNNISSQIEIHKEYGGVVPEIASRQHIKNIAAILDESLAQAGVTLDDIDYIGVTYAPGLIGALLVGVSFAKGLSYGHNIPLVPVHHIKGHIYANFAEHDVKLPCIALVVSGGHTNIIYIDENHKFTNLGGTLDDAVGETMDKVARVIGIGYPGGPVIDRMYYEGNPDFLKIPEPKVGEYEFSFSGVKTNVINYVNKMRMKGEEFKKEDLAASLQKTIVDILCKKVLKACDDKNVKQIIIAGGVAANSLLRKELKEKGAEKGIEVSYPSMKLCTDNGAMIAIAAYHKLMNGYKPKDILSLNGIATLNIADETE | K25706 | 404.03 | 482.3 | 1.80E-145 | tRNA N6-adenosine threonylcarbamoyltransferase [EC:2.3.1.234] |  |  |
| GC_00000274 | MSTRYLEKLIDEFHKLPGIGRKSAARLAFHVLDMNENEVEKFSEAMLNVKKFVKKCSVCGNFSENELCDICSNETRDRSIICIVEDSKDIIPLEKTGKYNGVYHVLNGKIAPLNGVTPDKLNIKSLLERIAKDDIKEIIFALNPDLEGETTVLYLTKLIKPFGITLTKIASGIPMGGNIEFTDSATIAKALEGRQEL | K06187 | 75.07 | 304.6 | 1.10E-91 | recombination protein RecR |  |  |
| GC_00000275 | MDGNGRWAKNQNKPRVFGHKAGANTLRKIMEYCNKIGVTYLTVYAFSTENWKRSQEEVDALMFLFKSYIKSERENLLKNKIRFMVSGREEGVNLSLMEAIKELEEATSKDYEMTLNIAFNYGGRAEITDAVNKILKDGRTSITEEEFSKYLYNDIPDPDFVIRTSGEFRISNFLLWQIAYSEIYITDKYWPDFDEIEMEKAILSYSKRERRFGGRLDVK |  |  |  |  |  | undecaprenyl diphosphate synthase | Immune Modulation |
| GC_00000276 | MFNETRVELEIGGRTLSLSTGKFARQSNGAVMIQYGDTVMLCTVNRSKEGRPGIDFFPLTVDYIEKFYAAGKFPGGFNKRESRPGIDATLISRLTDRPIRPMFPDGFNYEVQIVNTIFSYDGKNTPDYLGIIGASAAIMISDIPFLGPVAGVVVGRKDGQFILNPTPEELETSELNLKVAGTKDAVNMVEAGAAEMDEETMLQAIMFAHENIKKLCEFQEEFTKLIGKEKIEFVKEEPNPLVKDFLETNGEERLKQAVLTTGKQARQDAVDALHDELRERFIAENFAELTEEELPEDVMTEFDVYYEEMMKRLVREVIIFHKHRVDGRKIDEIRPLYAEVGTLPMPHGSAMFTRGETQALVTTTLGSKANEQLIDTLDEEYYKKFYLHYNFPAYSVGEIGRNGAPGRRELGHGSLAERALSYVIPSEEEFPYTIRVVSDITESNGSSSQASICGGSLALMDAGVPIKEHVAGIAMGLVKEGDEFTVLTDIMGLEDHLGDMDFKVAGTKSGITALQMDIKITGITEEIMRIALNQAHKARLEILEVMNAAIPEPRKELAPNAPRIVQMQINTDKIAALIGPAGKNIKKIIEETGATVDITDDGKVSVFCNDLEQLQKTVKMIEAHTKDVEVGEIYSGRVVKIAKFGAFMEILPGKEGLLHVSEISKERVANVEDVLKEGDVFDVKVISTEGGKISLSRKRIIEG | K00962 | 344.73 | 1025 | 0 | polyribonucleotide nucleotidyltransferase [EC:2.7.7.8] |  |  |
| GC_00000277 | MRNIKISYSYDGSDFYGFQRQPDKRTVQGEIERVLGIILKKR |  |  |  |  |  |  |  |
| GC_00000278 | MALRRAGLENCKVILGSATPSVESYYYGKKNIFELLSLETRYNNAVLPDVEIVDMKEEDDSFFSRELLKNIRETLLRKEQVILLLNRKGYSTMVQCKDCGHIEECEHCSIKMSYYHSKRTLKCNYCGTEKKFNGKCSNCGSTNLDFGGKGVEQVEHKLREYFDVPIVRMDGESAKEKNFYKETYYKFLNKEYDIMIGTQLIAKGLHFPNVTLVGVINADMILSFPDFRAGEKTYQLITQAAGRAGRGDKKGKVIIQTYQPENYVMEKIMKNDYEGFYNSEIEMRKILEYPPFSKIINIGISSSNEEKLERTARKLFEAVKRDYVEVYGPNKSLVYKVKDRYRENIFIKGNKKNIDYYKKELEKILSEFNDEGCRIVVDVDPVNLI | K04066 | 120.77 | 449.4 | 3.60E-135 | primosomal protein N' (replication factor Y) (superfamily II helicase) [EC:5.6.2.4] |  |  |
| GC_00000279 | MFGKLDEVVKRHEELTHLLGTVEVASDTKKMIEYNKALNEITPLVEKYSEYKSLVDDLEFIKENIKSEKDAEMKEMMQEEMKEIEEKLPGLEDELRILLLPKDPNDDRNVIIEIRGGAGGDEAALFAGDLYRMYVRYAERHRWKVEVIELQEIGIGGIKEVVFSIGGQGAYSKLKYESGVHRVQRVPATEAAGRIHTSTATVAVLPEVDEVEQINSINPSELKIDTYRSGGAGGQHVNMTDSAVRITHLPTGIVVQCQDERSQLKNREKAMKHLLSKLYEIECEKQRSQVESERKLQVGSGARSEKIRTYNFPQGRITDHRIKYTVYQLEAFLDGDLEEMIDALTTFDQAEKLKSIAE | K02835 | 358.27 | 535.1 | 2.70E-161 | peptide chain release factor 1 |  |  |
| GC_00000280 | MKAKDIREMSTEDLVVKCKELKEELFNLKFQLSLGQLTNTAKIREVRREIARMNTILNER | K02904 | 58.8 | 93.5 | 2.00E-27 | large subunit ribosomal protein L29 |  |  |
| GC_00000281 | MAKAIGTYQPNTRKYKKDHGFRARMKTKGGRQVLKRRRARGRKKLSA | K02914 | 24.63 | 70.9 | 1.50E-20 | large subunit ribosomal protein L34 |  |  |
| GC_00000282 | MKIAQELRQGSTIKIGNDPFVVLRAEYNKSGRNAAVVKLKMKNLIAGNIVDTVVKADEKMDDIRLEKVKAIYSYHDGTSYVFSNPETWDQVELSEEDLGDALNYLEEEMEVEVVYYETTPVAVEIPTFVERQIEYTEPGLRGDTSGKVLKPARLNTGFEIQVPIFVEQGEWIKIDTRTNEYVERIKK | K02356 | 92.23 | 224.6 | 1.30E-67 | elongation factor P |  |  |
| GC_00000283 | MAKVRIRLAKSIIGRKPNHIATVKSLGLKKMNSVVEHEATPELMGKIAQVSYLLDVEEVQ | K02907 | 40.97 | 64.1 | 1.80E-18 | large subunit ribosomal protein L30 |  |  |
| GC_00000284 | MAEFRRRRAKLRVKAEEIDYKNVDLLKRFVSDKGRINPSRVTGANAKLQRKIAKAIKRARNIALIPYTRTER | K02963 | 75.1 | 82.7 | 3.90E-24 | small subunit ribosomal protein S18 |  |  |
| GC_00000285 | MTAYEIIRKPLITEKTELLRRNNNKYTFEVNRKANKIEIKKAVEEIFNVKVASVATVNIKPVTKRHGMKLYKTQAKKKAIVELASGTISYFKEV | K02892 | 53.07 | 78.1 | 1.10E-22 | large subunit ribosomal protein L23 |  |  |
| GC_00000286 | MEVKASTRFVRMSPRKARLVADLVRGKSALEALDILEYTNKKAARIIKKTLASAIANATHNAKLDDEKLVISTIMINDGPALKRISPRAMGRADIIRKPTAHVIVGVSEK | K02890 | 76.43 | 161 | 4.10E-48 | large subunit ribosomal protein L22 |  |  |
| GC_00000287 | MQRCEISGKGITFGNQISHSHRLTGRVWKPNLQPVTVVINGQTLKLKVCTKVLKSIKGASETELMQILKANANTLSPRITKALSK | K02902 | 20.47 | 65 | 1.30E-18 | large subunit ribosomal protein L28 |  |  |
| GC_00000288 | MTKKELASQLQAKGIFETKAEAERKIDAILDTMEEALLSGDSINFIGWGKLEVVNRAPRLGRNPKTGEEVEIEARKSVKFKAGKGLLEKLN |  |  |  |  |  |  |  |
| GC_00000289 | MVTKDSNILEAARKYPVIGMVFRKYGLGCIGCMVASGESLGEGLAAHGLNPDAIIAEINMLIEEQEKAAK |  |  |  |  |  |  |  |
| GC_00000290 | MKLDFKCLKCGCEKYYVRTAILPEKEAGLKIEMGTYYLKICAECGYTEMYSAKILNKDKKKENSFEPKTEPNP | K07069 | 29.73 | 40.7 | 2.50E-11 | uncharacterized protein |  |  |
| GC_00000291 | MACSKTVEITNETGLHTRPGNEFVSLAKTFSSAIEVENEEGKKVKGTSLLKLLSLGIKKGAKVTVHADGADEAEACEKLAELLANLKD | K02784 | 78.17 | 91.6 | 7.10E-27 | phosphocarrier protein HPr |  |  |
| GC_00000292 | MDFDEIYDEYFDRVYYKILSSVKNPEDAEDIAQEVFISVYKNLHKFRADSKIYTWIYRIAINKTYDFFRKKKIDLELNEEILNIADNEDIGGSMIVEENLKKLSKEEREIVVLKDVYGYKLREISKMKDRNISTIKSIYYKALKNLEED | K03088 | 98.1 | 102.5 | 3.20E-30 | RNA polymerase sigma-70 factor, ECF subfamily | alginate biosynthesis protein AlgZ/FimS | Biofilm |
| GC_00000293 | MELNELRPSVPRKARKRVGRGESSGLGKTAGKGSNGQKSRAGGGTKPGFEGGQMPIIRRTPKRGFSNYPFKKEYAIINLDVLNRFEEGTVVTPELLLETGLVSKLLDGLKVLGNGNLEKKISVEAHKVSKSAQKAIEEKGGSVEIIEVKTFADVAKNNK | K02876 | 45.63 | 188.1 | 3.20E-56 | large subunit ribosomal protein L15 |  |  |
| GC_00000294 | MAKKSMIARDIKRAELCDKYAAKRAELKKRVLEGDQEAMFELSKLPANSSPVRKRNRCQLDGRPRGFMREFGMSRVKFRQLAGAGVIPGVKKSSW | K02954 | 28.9 | 120.3 | 1.10E-35 | small subunit ribosomal protein S14 |  |  |
| GC_00000295 | MNHNKSYRKLGRRADHRMAMLKNLTISLVREERIETTVTRAKELRKFAERMITLGKNGTLADRRRAFAFLRDEEAVAKVFGDLAARYAERNGGYTRIIKTSVRKGDSAELAIIALV | K02879 | 33.17 | 191.4 | 3.50E-57 | large subunit ribosomal protein L17 |  |  |
| GC_00000296 | MEGLLTALLFIFAIALIVLVLIQPDRSRGLSGSMGMGSANTVFGLSKDGGPLAKATKIVAALFIITALLLYLYLS | K03075 | 28.07 | 64.5 | 1.60E-18 | preprotein translocase subunit SecG |  |  |
| GC_00000297 | MRLDKFLKVSRIIKRRPVAKTVVDGGKAKINGKTAKAGTVVKTGDILELEYYDKYFKFEIVEVPEGNVPKEKSNDLIKVLDSRGIEVDLSSDKEILE |  |  |  |  |  |  |  |
| GC_00000298 | MANSKSAKKRVLINERNRVRNQAVKTRVKTMIKKVLAAVEAKEVEAAKAALTVAFKELDKAVTKGVLKKNTASRKKSRLAVKVNSL | K02968 | 24.4 | 108 | 6.80E-32 | small subunit ribosomal protein S20 |  |  |
| GC_00000299 | MGRYRKKRNIMSLDLTPLIDVVFLLLIFFMVSTTFNKYGKIDIDVPVSKVSEKTEDTKVEIVIDKNENYFILKDGKTFPINIDDLGEYLKDVKEVSITGDKNLKYQTVMDLITKVKQQGIENLGINFYE | K03559 | 31.37 | 95.6 | 4.70E-28 | biopolymer transport protein ExbD |  |  |
| GC_00000300 | MAVEMQVVAKFDEIFGSEGKKEIFFTPGRVNLIGEHIDYNGGYVFPCALSFGTYAVCRRRDDDIFRMYSMNFEKDGIVEFSLKKLIKTDVWADYCKGVIDTFIKHGYKIEHGADIVFFGNIPNGAGLSSSASLEVLMGTIIREISGLNDKVSMIDIVKFGQEAENKFIGVNCGIMDQFAVGMGKEDKAILLNCDTLEYDYVPVKLDGISIVIMNTNKRRGLGESKYNERRASCEAALKDIQEAGKKINHLCDMTMEDFEEVKKYIKSEEAVPRVRHAVSENVRVLESVKCLKENDIEKFGKLMNGSHISLRDDYEVTGKELDAIVAAAWEQEGTVGARMTGAGFGGCAVSLVKNEFIDEFIKNVGEKYTKETGLEAAFYVANVGDGSRKLGEC | K00849 | 357.33 | 453.2 | 1.20E-136 | galactokinase [EC:2.7.1.6] | D-glycero-D-manno-heptose 7-phosphate kinase | Immune Modulation |
| GC_00000301 | MKLQRLKRRNSGSIILEMTPLIDVVFLLLIFFLVATSFEDIDSGIKIDLPQSTIREIKAVKELQINITNGKVIYLKYQEGNERKSVKVSKEGLKKTLADKLSRAEDKTVIISGDKSLDYGFIVDIMTISKEAGAAELDIDTMLGK | K03559 | 31.37 | 91.8 | 7.00E-27 | biopolymer transport protein ExbD |  |  |
| GC_00000302 | MTFQEMIFTLQQYWGSKGCVLGNPYDVETGAGTFNPNTFLMSLGPEPWKTAYVEPSRRPKDGRYGENPNRVYQHHQFQVIMKPSPDNIQELYLESLRLLGIIPEEHDIRFVEDDWESPTLGAWGLGWEVWLDGMEITQFTYFQQVGGLELDIVPVEITYGLERIALYIQNKSNVYDLDWTENVKYGDMRYQYEYENSKYSFELADLDLYFKWFDEYEKEAKSILEQGLVLPAYDFVLKCSHTFNVLDSRGAISTTERMAYILRVRDLAKRCAEIYVENRKNLGYPLLKK | K01878 | 503.5 | 560.9 | 3.80E-169 | glycyl-tRNA synthetase alpha chain [EC:6.1.1.14] |  |  |
| GC_00000303 | MKPLFVNYPKCSTCQKAKKWLEENNIEFDSRHIVENNPNKEELKKWLSMSGEPVKKFFNTSGILYREMNLKEKVASESEDNLLALLATNGMLVKRPLLITDDTVLIGFKAEKWAEYFGK | K00537 | 88 | 109.9 | 1.80E-32 | arsenate reductase (glutaredoxin) [EC:1.20.4.1] |  |  |
| GC_00000304 | MVDFKKVDEYIDYIEEGKIPEGMTFNEFAVEFYHESKVIPISKYLRNRGRTSKMPKIMNTKKAGEILSETEKAGDEVRAYLKRRGYSEIPELNYTLVMLVRKVELLDNWKKIIAYLQGDKTIEEINNSTKSKLLPGEVARLENYIMEELNLDEEKMNWFLSKFKKLENNKELFRAVKKLNRQ |  |  |  |  |  |  |  |
| GC_00000305 | MAKIQVILTQDVAGQGRKGDMITVSDGYAKNFILNKNKGIIATPEALQKIENDKKKEAKRNEDEKNKAIILKNQLEKEKLVLTVKVGDNGKLFGAITNKEIAAEMEKSFGLKIDKKKIECSIKSLGEHKVTIKLHPEVKAEITVITKE | K02939 | 61.53 | 127.9 | 8.70E-38 | large subunit ribosomal protein L9 |  |  |
| GC_00000307 | MAFNKEQFIADLEAMSVLELRELVSALEEHFGVTAAAPVAVAAGPAAAEVEEKTEFDVILANAGDKKIGVIKEIRGITGLGLKEAKELADNGGVIKEGASKEEAAEIKAKLEAAGATVEVK | K02935 | 22.6 | 142.4 | 2.30E-42 | large subunit ribosomal protein L7/L12 |  |  |
| GC_00000308 | MGIKSFEKIRIKLASPEKIYEWSHGEVTKPETINYRTLNPEMDGLFCERIFGPSKDWECACGKYKRMRYKGLVCEKCGVEVTKSKVRRERMGHIALAAPVSHIWYSKGTPNKMALILGISPKELESILYFARYIVIESSEETLPVGKILNEKEYKLFKQMYGNAFDAKMGAEAILKLLEDLNLPVLREELEKELEDVNSSQKRKKVAKRLKIVRDFMESNNQPSWMILKNVPVIPADLRPMVQLDGGRFATSDLNDLYRRVINRNNRLKKLLEIKAPEIVVKNEKRMLQEAVDALIDNGRRGKPVVAQNNRELKSLSDMLKGKQGRFRQNLLGKRVDYSARSVIVVGPSLKMNQCGIPKKMALELYKPFIMRELVKRELATNIKTAKKLVEEADDKVWDVIEDVIKDHPVLLNRAPTLHRLSIQAFEPVLIEGKAIRLHPLVCSAFNADFDGDQMAVHLVLSPEAIMEAKLLMLAPNNIISPANGQPIAIPGQDMVMGCFYMTKDRPGCKGEGKLFSNKEQVLTAYQNGVVDTHSIIKVRIKEEFVTTTPGRILFAELLPEEIRDYHMTYGKGPLKKLIGELYERYGFVKAAEIIDKLKNFGYHYSTFAGVSVGIEDLEIPASKKEILENADKEVARIEQEYKAGHIINEERYRRTIAVWSQATAAVTDAMMNGLDEFNPVYMMANSGARGSIQQMRQLAAMRGNMADTRGRIIEVPIKANFREGLTVLEFFMSSHGARKGLADTALRTADSGYLTRRLVDISHEVMVNADDCGTHEGIEVAELVSDGNVIETLAERINGRVLAEDLIVDGEVIAPRNTMIGKALIKKIEELGIKKVKIRTPLTCALEKGVCKKCYGMDLSNHKEVLLGEAVGVIAAQSIGEPGTQLTMRTFHTGGVAMATASATTKKAEVSGKLKFKDVKILVNEETNDEIVVSQSAKISIGNYDHEIPSGAILRVKEGDTVQAGDILADIDPYHVPIICDQDGTVEFKEIYIKANYDEKYDVTEYLAVKPVESGDANPRLIVYDKDHNPKASYPIPFGAYLMVKEGDKVTKGQILAKLIKEGEGTKDITGGLPRIQELFEARNPKGKALLTEIDGKVEITAKKRKGMRVIIIRNEKDPDLFREYLVSVGDHLVVTDGMLIKSGDKITEGAISPFDILNIKGLVAAEQFILESVQQVYRDQGVTVNDKHIEIIVKQMFKKVRITNSGSSLLLEDEVVEKRLVDLENEELKAKGKKLVEYEPVIQGITKAAVNTESFISAASFQETTKVLSNAAIEGKEDYLEGLKENVIIGKKIPAGTGYVDYRNVVPTEVKEEQ | K03046 | 980.37 | 2104.2 | 0 | DNA-directed RNA polymerase subunit beta' [EC:2.7.7.6] |  |  |
| GC_00000309 | MDTYHYLFLLVILILLSGLFSASETALTSFRSIHLEEISEKNPKKGELLKYWLKKPNEMLTALLLGNNIVNILATSLATAFITSWLNDRGMGNSQNMSVFISTIVMTVVILIFGEITPKVIAKNNSTEISKSVIVPIYCLTKLTTPVIWILTLISKFIGRIIGVDIKDEAIMITEQDILSYVNVGEAEGVIEAEEKDMIESMVTFGETCAREVMTPRTSVFAIEGNKSINDVWKDIVEQGYSRIPVYKDGMDEIIGILYIKDLLNAVKEGKCDMPVKEFMRKAYFVPETKSIIKILEDFKTQKVHMAIVIDEYGGTVGVVTIEDLIEEIFGEIRDEYDKEEEEAIKEKGPNTYEVDAMLDIETINKELDIELPESEDYESLGGLIMNELGEIAKAGDIVKISGVELKVLEIQKMRISKVQIKKESEEKVCTEE | K03699 | 304.37 | 474 | 7.60E-143 | magnesium and cobalt exporter, CNNM family | hemolysin C | Exoenzyme |
| GC_00000310 | MGKLILIRHGQTDMNKDQLYYGRLDVPINETGKEQAENTRKNLVELEIDYDKIYSSPMKRAYETAEIVNYKNLEIEKDNELREMDFGIFEGLSYKEIIKKYPEEMEKLKKDWKTYSYVTGENPFMLQKRALKFLEKIDKNKNNMVVTHWGIICTLLSFLFSSELEGYWKYQVKNGGIVIIEFADGYPVLCGFNVGR |  |  |  |  |  |  |  |
| GC_00000311 | MGKANEKALEFINVLVNNDMVGELENHDDQGVKVSAHTYDVLKVSIDEIRRDYSDFDEAKRFIDFFAIIVGVIIHDLSKGSIRKTSETMSHSQMMLKKPDYIMKEADSLLTEVENQTGLKIKESIRKNITHIVVSHHGRWGKIAPNTREAHIVHRADMYSAKYHRINPIGANEILEALCMGENLVEVSKRFMCTTGVIKDRLKRAKMELRLKNTKHLINYYKKTRKVPIGDNFFTKRVKETAKLIAAVDKKGFKKLILTNQLLDYLVDSEIFEPAGEENERKN |  |  |  |  |  |  |  |
| GC_00000312 | MHITLYRKYRPKDFSEIAGESDIVKTLKNSLDNDRISHAYLFSGPRGVGKTTSARLIAKGVNCLKNGISSSPCNECENCREIDNGSFIDLIEIDAASNRGIDEIRELKDKINYQPSKGRKKIYIIDEVHMLTKEAFNALLKTLEEPPEHVIFILATTEPDKILPTIISRCQRYDFKSLIYTEVKDKLSEICRGENVEIDEGSLGLIYESSGGSMRDAISILERVIITYLGEKIDEDKCSKVIGITSKTLLKEFLGIVKGNRISEGAVFLDKLWLDSLDIEKFFKDFGKYIKNLVLSGEMEAEEGLKIIGNIFDSLNKFKYEEDKRLLGYVVLNSLIKKADKGTKEVVVEKQIVYKEVPKVSQEKNGTENKISEKITTEYQREINIDEIKEKWSEIVRAAKNEKMTFSAFLMDAAPYRIEDNILYIKFNSNLFAKEQMETEYYNSIFQDVVERITGLKIRTKYIFKENNKSEKSKSDNDLASQLMTYFSEEN | K02343 | 238.13 | 447.8 | 6.50E-135 | DNA polymerase III subunit gamma/tau [EC:2.7.7.7] | Type VII secretion system protein EccA3 | Effector Delivery System |
| GC_00000313 | MKILISLLVLGIIIMIHELGHFLCAKFFKIPVSEFAIGMGPEVYTYEGKKTKYTFRSIPVGGFVNIEGMEVDDDVEGGFNKRNPFVRFAVLFAGVFMNFLLTYFILFGITMSQGEVILNPLPVVGKVAEASKSSFKEGDLILEIENHKIEKWTEIRGIISELPNKDELKKVVIERNKQREVLEVKLNKGPDDRYYLGILPDYRIEKFGVSEALKISGKGFINIFGEILNGFKMIAAGKVSRSDISGPIGIVNIVGEASEQGVFSIIWIMAILSVNVGIFNLLPFPALDGGRIIFVILEMFGIKVNKKIEENVHKVGILILFILIIFITTNDFFNLTGK | K11749 | 74.77 | 306.4 | 5.40E-92 | regulator of sigma E protease [EC:3.4.24.-] | metalloprotease protease | Biofilm |
| GC_00000314 | MSIRPIGNRVLIKTPKAEEKTAGGIILTPSSLKSNPNIGEVIAVGNGEDVKILKTGDRIIHSEYTGTKVKDGNEEYTIIDFDSVLGIVE | K04078 | 27.9 | 88.2 | 7.10E-26 | chaperonin GroES |  |  |
| GC_00000315 | MATRKRKRAESEEKREYFIVDRRILPTSIQNVIKVNDLIQQEKISKYEAIKRVGLSRSTYYKYKDYIKPFFESGKEKVFSIHLALVDEPGILARILDVIASHDMNILTIIQNIAIDGIGRATISVQTTENILRKIEGMLEIISEIEGVKELRIIGSN | K06209 | 121.5 | 154.6 | 1.70E-46 | chorismate mutase [EC:5.4.99.5] |  |  |
| GC_00000316 | MRKQLTILLSFLLLIFAYGADTNYKIEKIVVENIQEIPEASIVSIMKEKVGDKYSAKDMIADYQKIKELDYVGSVSIYPQYYNEGIKLVVDIREKRDTKELLTKNGIIPMSERDKIDKTVIVKGVEVYGNVSMKKKDILKYIPIKTGGYFSKKKVIDGYRSLGESGYFSQVVPDVQKTGNGVTVVYYVTENPTITGVNIIGNTVYPTDELLALLETKPNETLNFNSLRKDREAIIGKYSKDGYVLARVIDIGLNNSYELDIYLTEGVVRDIKLQKMVTKQKGNRRQATDTLLKTKDYVIEREIEFKEGEIFNINKYTQTENNLKRLGYIKNVKYEARDILGDSDGKDIVLLIDEDRTARLQGAISYGSELGLMGMLSLEETNWKGKGQTTSFTYEKSDEDYSSLSLSFSDPWIKDTDRISWGWSLYKNEYENDDSEAFANIDTYGFRINVGKGITRNVRIGLGTKVEYVTTEPDDSLSDAYIKEAGYYDDKYYLWSLYPSITYDTRNSYFNPTRGEYARWQVEGGYASGEKADYFANTTIELRKYHQGFFKKNTFAYRAVFGIQSDTTKESQRYWVGGSSTLRGYDGGSFRGTQKFTASIENRTEFNDVLGGVLFFDFGRAWDYYKNDAVDQGYRKSRENADEKFPDGIAMAAGVGLRINTPMGPLRFDFGWPINDDEESGMQFYFNMGQSF | K07277 | 289.5 | 314.6 | 3.00E-94 | outer membrane protein insertion porin family |  |  |
| GC_00000317 | MDFGIISAISTVVNFLNNIFWSYLLIILLIGAGVYFTLKNKFVQFRMMKEMVVLLKEGTGEAKHGISSFQAFCISTASRVGTGNLAGVALAVAAGGPGAVFWMWLIALLGSASAFVESTIAQVYKEKNGTTFKGGPAYYIEKALHCKWLGLVFAVLISITYGLVFNSVQANTISFAFENAFGIGRTSVAIALFVLTGLVIFGGIHRIASISEKIVPFMAVIYVLIVLGVLAININKLPGIIVLIVKSAFGPEQFMGGALGAAVMQGIKRGLFSNEAGMGSAPNAAATAHVTHPVKQGLVQTLAVFTDTLIVCSATAFLILVSGTYQTSSADGIALTQEALTSQIGSFGGPFVAICIFLFAFSSLVGNYYYGEANIQFMCPENKFFLNIYRFACLFMISFGSIAELTFVWSLADLFMALMAVINIYAILRLGKVAKICLDDYIAQKKAGKDPVFRGKDVGLTENVECWD | K03310 | 266.3 | 569.2 | 1.20E-171 | alanine or glycine:cation symporter, AGCS family |  |  |
| GC_00000318 | MEKIGIFYGSNTGKTAAVAEEIEFNLKKDNYEVINVADGIETMKDFKNLILLTPTYGVGEVQEDWANVMPQFEKIDFTGKRVAVAGLGNQFAFGESFAGGMRVLYDVVVKNGGEVIGFTSNEGYHYEESEAVIDNQFVGLAIDENNQDDETPERVMDWVAELKKKFY | K03839 | 119.83 | 174 | 4.80E-52 | flavodoxin I |  |  |
| GC_00000319 | MLLQERGNYKMNNNLKKTLINNIVPILILIIIAFCFPLSGLSVTYTIQEMILRLSRNLFLVLSLLIPIIAGMGLNFGIVLGAMAGQIALVFITDWQVVGMQGVLLAAIISVPISILLGIMSGMILNRAKGREMITSMILGFFINGVYQLVVLYGMGKVIPITNNKLVLSRGYGIRNAIDLKDVRKVLDSVFSLKIGEIEIPVFVFLIVAALAWFTVWFRKTKLGQDMKAIGQDMEVSKTAGIDVERTRIIAIVISTVLAGFGQIIYLQNIGTMNTYNSHEQIGMFSIAALLVGGASASRATILNAISGIFLFHMLFIVSPMAGKELMGSAQIGEYFRVFVSYGVIALTLVLHQWRREKEREELRKRLMEERAAQTQDGDE |  |  |  |  |  |  |  |
| GC_00000320 | MEKLENLLSYILKGLVEKQDEVRITYELIDDTIIFKVNVAEGEMGRVIGKNGLTANAIRGVMQAAGVKDKLNVNVEFLD | K06960 | 47.53 | 75.8 | 4.10E-22 | uncharacterized protein |  |  |
| GC_00000321 | MGHIADKDFIRGEVPMTKQEVRAVSVAKLKLSENSVLIDVGAGTGSVGIEAATYIPHGKVYAVERKSEGIKLIEENLKKFSIKNLEVIQGTAPDDLCIKNFDRMFVGGSGGRLDEIIKYFSDYSAEKSIVVINAITLETLSEVKNIFEKYKIKNMEIISMSVARGKKAGNYTMMFGENPIYIISGEKGEENE | K02191 | 213.83 | 240 | 2.00E-72 | cobalt-precorrin-6B (C15)-methyltransferase [EC:2.1.1.196] |  |  |
| GC_00000323 | MKGQRVNPEKLNPMEMNSMSSMMGMMSLLQKIGKGKRKYSVKLEKNHKKFLARFMTDIKKQFTETYADTQMKGVLTFFDYVKSACENKTATELKLSYEEFDFLKRLLTDSLKGIESMQLKWYNLIKKATLKMMKTQYKEVLKQMK |  |  |  |  |  |  |  |
| GC_00000324 | MKERIDVLLVQNGFFEDIEKAKRAVMAGIVLVNDLKIEKPGTQIKIEDDKELNIRIKGKSFKYVSRGGLKLEKAVKVFGLDFAGKKVLDVGSSTGGFTDCALSFGADHVYAVDVGTNQLDWKLRNDERVTSIENRHIRDLTLEEINNSEIDFIVMDVSFISITNVFDSLKKFFKADTKLMALIKPQFEVEKEMIAKGGIVRDKKNQIFAVERVIKEAEKHGLYLEKLDFSPIKGGKGNSEYISLFGLEKKDNADINIENIVELCENLGGAL | K06442 | 68 | 328.3 | 7.60E-99 | 23S rRNA (cytidine1920-2'-O)/16S rRNA (cytidine1409-2'-O)-methyltransferase [EC:2.1.1.226 2.1.1.227] |  |  |
| GC_00000325 | MKKLKSGYTTGACAAVCVKAALLSLLENYEAEEIEIDSLNGKKITVPIKKIKKRKNSATAVVEKYSGDDPDVTNGIDICVKVKLLEKDKNFPEIKRGYYFNNILIYGGYGVGISTKKGLQCPVGKSAVNPGPLKMIENTAKEILDERNLKAEILIYIPQGREKAKKTFNEKFGVTGGLSVLGSTGILNPMSEEALKESLYTELKVLKENKGGDFVIFSFGNHGKKYCEEHGFSPERIIVMSNYVGFMLESAAELGFKKIILVGHIGKAVKIAGGIYNTHSRTADCRMEIMGANAFLIGEKSENIMKILKANTVEEACEYVEKQELFSLIAEKAAAKCREYVRDENIYCEVMLFSFSGKELGHSRNFYELLNEVSSVEEN | K02188 | 127.2 | 419.9 | 1.80E-126 | cobalt-precorrin-5B (C1)-methyltransferase [EC:2.1.1.195] |  |  |
| GC_00000326 | MRKYFGTDGVRGEANRELTADLAMRLGYALGYYLRKENPDKKKLKVIMGSDTRISGYMLRSALSAGLNAMGVNIDFVGVIPTPGVAFITQAKGAEAGIMISASHNPAKDNGIKIFANDGCKLPDEVELELEKYMDNFAEMTKDPLPGDQVGKFKYAEDDYFLYRDHLKSIVKGDFSGMKIILDAANGSAYRAAKDVFLSLGAEIVVINDAPNGKNINVRCGSTHPEILSKVVMGYEADLGLAYDGDADRLIAVDRNGKIVDGDKIIATLAVDMKKKNTLVNNRVVTTVMSNMGLESFLNDNGIVLVRANVGDRYVLEKMKSQGLNIGGEQSGHIILLDYGTTGDGIQSSLKLVEVVRDSGKTLDELVGVIPEWPQVLINVRVDNVKKNLWNKNENIVSFIDAKEKLMGNSGRVLVRTSGTEPIVRVMVEGKDAEAVKVIAEEIAEVVKKELA | K03431 | 461.33 | 627.8 | 2.80E-189 | phosphoglucosamine mutase [EC:5.4.2.10] | phosphomannomutase CpsG | Immune Modulation |
| GC_00000327 | MKNFIMAIDGPAGSGKSTIAKILAEKNNLTYLDTGAMYRMVALYFFENNVDLDNDMEVKINLSKIKMDIEKDKFILNGKDVSKDIRTPRVSGLVSFVARIKRVREKLVELQREISRGKNVVLDGRDIGTVVFPDAPLKIFLIASAEERAKRRMKEYEEKGIKEDFEAVLANIKERDMIDSTRSEGPLKKADDAVEIDTSFMTIEEVTDEITRLVKEKTGE | K00945 | 148.17 | 299.7 | 4.50E-90 | CMP/dCMP kinase [EC:2.7.4.25] | adenylyl-sulfate kinase | Immune Modulation |
| GC_00000328 | MTKYEIMYIINPTILEEGRDAVIAKVDAILTESGAALTKTEKWGERKLAYPIDKKKTGFYVLTTLEMDGTRLVEVERKLNITEEVMRYIIVKQD | K02990 | 21.33 | 91.6 | 5.80E-27 | small subunit ribosomal protein S6 |  |  |
| GC_00000329 | MKKFIIEPEYNNFKVSDYLKEVKGYSSRGLRNAEIYLNGKKVRLDKKIKKLNRLVVVEQEKGTNIQPIPMDLKIVFEDKNLLIINKDANVVVHPTLKKTDITLANGVVDYLFKATGKIQVPRFYNRLDMNTSGLIVVAKNAFAQAFLQEKAEIRKFYLAICEGIIEKDEFFIERPIGRVGDNIKREELSVENGGQEAKTKVKVIERFPEKNLTLVELELFTGRTHQIRVHLSLEGHPILGDHLYGKENPDIKRQLLHSYKFIYTDIDTHEQKIIEIGLPDDMKNIIGVK |  |  |  |  |  |  |  |
| GC_00000330 | MYVIPLGGLDEVGKNMNLVQYRDEIIIIDCGVGFPGEGLLGIDLVIPDFSYVENNKNKIKGLFVTHGHEDHIGSIPYLYQKIDKEVSIFSGKLTLALIEGKFENLKMRDLPKMREVKNRSKVKVGKYFNVEFIRVTHSIADSYAVLVTTPAGTALFTGDFKIDLTPVDGLGTDLARLGQIGEKGVDLLLADSTNSEVEGYTPSERTVGAAFQLEFAKAKGRIIVAAFASHIHRLQQIVTIAESYGRKIAIDGRSLVKVFEVAQKMGYLKIKKDTMIALSDVENEKDNKVVILCTGTQGEPMASLSRIAQNIHKHTKIKEGDTVIISATPIPGNEKAVSKNINNLLKYDAEVIFKKVAGIHVSGHASKKEQELMLNLIKPKYFMPVHGEYKMLKAHIETAIATGVPKANTLLGLNGSKIEITKSGVKIKGKVSAGATFIDGLGVGDIGQTVLRDRQQLSQDGVIIVVFTLNKETGKIIAGPEIVTRGFTYSKDSDELIQGTVEHINEKLAKLEDTKIKDWQPIKNLTKEAVAKFVYNKTKRNPVILPIVMEV | K12574 | 130.87 | 748.4 | 9.20E-226 | ribonuclease J [EC:3.1.-.-] | adhesin FdeC | Adherence |
| GC_00000331 | MLYIVATPIGNLEDITLRAVRILKEADYIFAEDTRVTKKLLNHLEIANTLYRYDEHTKQHQVENIVNLLKEGKNVALVTDAGTPCISDPGYEVVDAALNEGIKVVPIPGVSAMTAAASVAGVSMRRFIFEGFLPKKKGRQTLLKSFADEKRTIMFFESPHRVVKTLKDIEEFMGVKNIVLVREITKIYEEIIRGTTTELIEKFEKQTLKGEFVIIVRGNEQEEKKEKVNKYAKDDYEEEDDYEDEE | K07056 | 97.8 | 342 | 5.80E-103 | 16S rRNA (cytidine1402-2'-O)-methyltransferase [EC:2.1.1.198] |  |  |
| GC_00000332 | MKEFIKNEKVLGLLRKAIQEKTITYEEINRELKDELPVDKIEYLIEGMIDQGIDIVKEEDLKKNAEEEFDDKKYEEFSKKSFMDEDEDDFGDFDDLDLDDEDMDIDSIEEISRDELLDDELLNISGDMGVDEPIKMYLREIGQIPLLNHNEELEYAKRAYEGDEFAAKQLVEANLRLVVSIAKKHTNRGLKLLDLIQEGNIGLMKAVEKFEYTKGYKFSTYATWWIRQAITRAIADQGRTIRIPVHMIETINKIKKEARIYLQETGRDATAEVLAKRLGMEVEKVKAIQEMNQDPISLETPVGSEEDSELGDFVEDNKMLNPYELTNRVLLREQLDEVLDTLNNREKQVLRYRYGLDDGAPKTLEEVGKIFKVTRERIRQIEVKALRKLRHPSRRKKLEDFKQK | K03086 | 459.57 | 490.9 | 6.80E-148 | RNA polymerase primary sigma factor | RNA polymerase sigma factor SigA | Regulation |
| GC_00000333 | MTEEIKEISEVEIEINEITEEPREEQFYTVLGVMFDITKKRYYFEVVDKDEVYKKGDKVIVDTIRGQEIGIVYNLPITLSEKYLVLPLKPVIKKANEEEIKRYEDLRREAGEAQKICKEKIEKHNLPMKLVNTEYTFDKTKLIFYFTAEGRIDFRELVKDLANIFKLRIELRQIGVRDEARILGDIGVCGKELCCRTFINKFNSVSIKMARDQGLVINPSKISGVCGRLLCCINYEYAQYEEALKNFPAINQNVGTEKGEGKVASISPLNGFLYVDVPKLGITKFNIEEIKFNRKEAKKLKSETTEEERAHKDLEKE |  |  |  |  |  |  |  |
| GC_00000334 | MYNERGYVDITASNKLLRTSFTYMILGLLVTFLVPAYIMFSENGFVLAGYIAKFYTPIIILEFATVLLFSFRIYKVSLMSAKLMFFFYSFLNGLVFSLIGMMFIGNLMIIAYSLLTTIVMFTVIAVYGYTTNEDLSNYGGYLKTGLISLIIMSLINMFLHAPMLYWTVTILGVVIFSALIAYDVNRIKNMAYEVAEGDDEIIGKLGIVGALNLYLDFINLFLYIIRIFSKRK | K06890 | 153.17 | 194 | 4.80E-58 | uncharacterized protein | type VII secretion system protein EsaA | Effector Delivery System |
| GC_00000335 | MKKILSFDEINKIAEKLADYVSAGDIVALIGDLGTGKTTFTKKFAETLGVKENLKSPTFNYVLEYFSGRIPLYHFDVYRLSEPEEIYEVGYEDYLNGDGVMLIEWADIIKSELPKEYIEIILEYDGDETRKVSIEYIGNKEKEKEMLEYVNFSR | K06925 | 56.43 | 159.4 | 1.70E-47 | tRNA threonylcarbamoyladenosine biosynthesis protein TsaE |  |  |
| GC_00000336 | MFEYLKGEVAYKKPEYLALDVNGVGYKIFISLKTYDSVEEGEIKRFFIYNHIKEEEFKLVGFLEAKERNLFEMLLSVKGIGVSLALAIMSTFDVEMIRGLVLQEDFVTLKKVPKLGEKKAQQIILDLKDKLKKLDIISMEKTDTVVYTSVEDELIMALEGLGYNKKDIDKLIDKEEIKNYKNIQEAIKGTLKKIQNSKK | K03550 | 47 | 184.4 | 2.90E-55 | holliday junction DNA helicase RuvA [EC:5.6.2.4] |  |  |
| GC_00000337 | MNNLGNIKIADDVVKAIAAKATEDVEGVYKLAGGVADEVSKMLGKKRVTNGVKVEVGEKECSIDTFIIIDYGYPISEVAKKVQESVLKAVSELTALKVVEVNVFVQDIKIHDENTVEPEIL |  |  |  |  |  |  |  |
| GC_00000338 | MNLDNMTLENLNKLCSDIREKIIDVVLKNGGHLASNLGVVELTVALKKVFNDNEKNRILFDVGHQAYVYKILTGREENFSTLRTYKGLGPFLDPKESSEDYFISGHAGSALSAGCGIAYAEPEVRVIVVVGDASIANGHSLEALNNMGNLKNMIVVLNDNDMSIGKSVGSLSNFFSRMISSRMYMNVKKDVKNIIDRGKFGRKVKNTLGRAEHSIKNFFLPMSISENLGFKYFGVVDGHNMEELLNIFEKAKETEGPVFIHVRTKKGKGYKPAEENKEKFHGVSPYNPFKVEGQKIYSDIFGEKLVSMAETDKDIYAISAGMVKGTGLKEFFEKYPERSIDVGIAEGHGVTFAAGLAISGKKPYYAVYSTFLSRGVGQLIHDVSLQNLPVRFMIDRAGIVGEDGKTHHGIYDVPFMLSIPNFTIIAPTTKKELEEVLEFSQNYNSPIGIRYSKENGFDIEGDKPFVLGKWREVRKGEKNLYIGTGSMLKEILDVEEILKEKGISGTIVSAASIKPMDTEYINKNFEKYDNIIVLEESYFENSFGSEIIDYINSSKINRKIIKIGIETGAVPHGKRGILLEEYGLRGLKLVERIEGKINGES | K01662 | 342.8 | 709.1 | 7.60E-214 | 1-deoxy-D-xylulose-5-phosphate synthase [EC:2.2.1.7] |  |  |
| GC_00000339 | MATLDQNLTPLFTVLKDVYVKRNIVPFHVPGHKQGHGVDEEFLEFMGTNPFKIDVTIFKMVDGLHHPKSCIKEAQELAADAYGVKKSFFAVNGTSGAIQAMILSVVKSGEKILVPRNVHKSVSAGIILSGAVPVYMNPEIDDELGIAHGVRPSTVEKMLEQHSDIKAVLIINPTYYGVATDIQKIANIVHSYDIPLIVDEAHGPHLHFHEDLPLSAVDAGADICCQSTHKILGAMTQMSLLHVNSNRVDANRVQQILSLLHTTSPSYPLMASLDCARRQMAIHGRDLLTRTIELANYLRSEINKIPGIHSFGAEIVGREGVFAFDPTKITISAKGLGITGFELESILTDDYNIQMELSDYYNVLGLITIGDTPDSALKLINALKDISKRFYDTKEIKKVKSLRIPAIPEPVLVPREAFYSDKNRVLFEESEGKICAEMIMAYPPGIPIIVPGERISKEVIEYIDELKETKTHLQGMEDPNLEYINVIEDEDAMYIYTEKMKNKIFGVPLNLGADKSGIEFGVDVLVENYPDTFDEIEVIDIEKQREDFNHPNMKYKNTILHTCEKVAKSINEAIEDGYRPITIGGDHSIALGTISGVAKTKEIGVIWIDAHADMNTNETTITGNIHGMPLALLQGEGDEDMVNCFFEGAKIKPENVVLLGARDIDVRERDVIEKLGVKVYHYDDVLRKGIDSVLAEIHDYLKVDNIHISFDIDSMNPIAAPGVSTPVKNGFDEDDVYKTFKFLFKNYFITSVDIVEFNPVRDKNEKTAALVRDFTEFMLNPIY | K01585 | 2.8.07 | 979.4 | 2.10E-295 | arginine decarboxylase [EC:4.1.1.19] | UDP-2-acetamido-2-deoxy-3-oxo-D-glucuronate aminotransferase | Immune Modulation |
| GC_00000340 | MSKFYGIGVGVGDPEMLTIKAVNALKELDIIIVPDAGRDFESTAYSIAKNYLKNDSKIINMEFSMNPDVKKREEERKKNGKIVEEYLNEGKNVGFLTIGDPMTFSTYVYLLENISSSHEVVTIAGISSFADMSSRFNLPLVMGNETLKVVPLHKNCDIKREIECADNIVFMKVALKFSELKKAVKETGNMNNILMVSESGKEKQKIYFNLDEVDEENVPYFSTMILKKGGVEKWKRFIS | K03394 | 138.33 | 228.9 | 1.20E-68 | precorrin-2/cobalt-factor-2 C20-methyltransferase [EC:2.1.1.130 2.1.1.151] |  |  |
| GC_00000341 | MNRLEGKVALVTGGSMGIGRAIVDRFAAEGAKMVISCDINPCEFEQENVRGEILNVTDREGIKALVKKIVEEFGTIDILVNNAGITQDAPFVRMSEAQWDAVININLKGVFNVTQAVAPVMTKHKKGSIITLSSVVGLYGNIGQTNYAATKAGVIAMSNTWKKELARKGAQIRVNCIAPGFIQSPMTDKLSEKAVEGILSGVPLQRMGTKEDVANTALFLASDESSYITGAVIPVSGGLSF | K00059 | 270.4 | 326.5 | 3.60E-98 | 3-oxoacyl-[acyl-carrier protein] reductase [EC:1.1.1.100] | short chain dehydrogenase/reductase family oxidoreductase | Motility |
| GC_00000342 | MARNRQPILKKCRALGIEPMVLGVNKTSKRGFRPNANRKPTEYAVQLREKQKARFIYNVMEKQFRKLYEEANRKDGVTGLNLVEYLERRLENVVYRMGFAKTRRQARQIVSHGHVLVNGRRVNIASYRVKVGDVISILENSKNLDLIKEAVEAANVPAWMELDKAAFSGKILQNPTKDDLDFDLDESLIVELYSR | K02986 | 70.13 | 203.8 | 5.70E-61 | small subunit ribosomal protein S4 |  |  |
| GC_00000343 | MKNSAAEGLKLDLKTTEEILVNFLREEVYKTGFKKVILGLSGGIDSALVAFLAAKAFGPENVYTVMMPYKTSSRESVEHAELVVKALGINSKKVEITPMADAYFAMNEDMSSLRKGNYMARTRMCVLFDNSAKEGAMVLGTSNKTEILLGYSTQFGDSAAAINPIGDLYKAQVWALSEYMGVPGEVVNKKPSADLWEGQTDEQELGFSYQTADEVLYYLIEERLTPEEVIKMGYDEKVVNAIIRKIKLSQYKRKLPVIANVSKRGMGNNFKFSRDWGM | K01916 | 284.67 | 309.4 | 7.00E-93 | NAD+ synthase [EC:6.3.1.5] |  |  |
| GC_00000344 | MEKIKSRNEIDKKYKWNTEDIYKDWNEWQKDVKKMKELMKEIPQYENKIENSREDFVKLIKLEESLSRILEKVYLYPYMLKDLDSTDQLVSQKLQEIEFLYAQFSVATSWISPKILEIPKETLEKWIDENDEIKDHKFNLMELYRLKAHVLDKDKEKLLSHFSQYMGAVNDIYDELSTSDIKWNEITLSTGEKVLVTNGMYSKILDGNKNQDDRRKAFEALYSSYDINKNTYSAIYKGILQRDAAGTKAKNYNSTVEKALEPNNIPVKVYENLINSAKENSGPLKKYVEFRKKQLGLDSYHYYDNQIKLADYTREFSYEEAKEIVLKSVEPLGEDYTKNLEKAVGEGWLDVYETPNKRSGAYSLNIYDVHPYMLLNYNGTMDAVFTLAHELGHTLHSMYSTKYQPYATNDYTIFVAEVASTFNERLLLDYMLKNTTDHKEKIALIEEAIGNIMGTYYIQALFAAYEYEAHKLTEEGNPITPDVLSEIMAKLFAEYFGDSLVMDELQKIIWARIPHFFNSPYYVYQYATSFAASSRLYEKVTNEKYSPEEREEARKKYIELLKSGGNNYPIEQLKLAGVNLEEKENFEAVAHEMTRLIGLLEKELKELK | K08602 | 139.97 | 609.9 | 9.30E-184 | oligoendopeptidase F [EC:3.4.24.-] | s-layer protein | Adherence |
| GC_00000345 | MALSKEEVLHVAKLARLEFSPEEIEKYQQELNDILNYIDMLNEVDAAEIKPLAQVNDDTNNLREDEVRKSLTVEEALLNAPEAEDGAVIVPKVVGGE | K02435 | 44.3 | 91.1 | 7.40E-27 | aspartyl-tRNA(Asn)/glutamyl-tRNA(Gln) amidotransferase subunit C [EC:6.3.5.6 6.3.5.7] | Hemolysin D | Exotoxin |
| GC_00000346 | MLGIIFFIFGTIGIVIPLLPTTPFYLLSAFLFGKSSERCYKFLLNNKIFGKYIRDYHERKGITLKNKINSIAVLSIGIGWSMYKMENIHGIIFLSIVFICVCFHIIKLPTLK | K09790 | 33.5 | 125.3 | 3.20E-37 | uncharacterized protein | YbaN family protein | Nutritional/Metabolic Factor |
| GC_00000347 | MSNIINRYIEEEMKQSYLDYSMSVIVSRALPDVRDGLKPVHRRILFAMNEMGMTYDKAYKKSARIVGEVLGKYHPHGDTAVYNTMVRMAQEFNYRYELVDGHGNFGSIDGDSAAAMRYTEARMKKITGELLEDIDKDTIDFRKNFDDSLDEPVVLPAKLPNLLLNGSTGIAVGMATNIPPHNLGELVDGTLALIDNRDITTDELMEYIPGPDFPTGGIIDGRKGIREAYATGRGRLRVRGKIETEEAKSGRVFLVVKEIPYQLNKSALIERIAALVREKKLTGISDLRDESDREGIRIVIELKRGEEPELVLNKLYKYTDLQSTFGVIMLALVNNVPKVLNLKQVLEEYMKHRFDVITRRTRYDLDKAEKRDHILQGFRIALENIDRIIELIRASKDGNEAKEALIERYAFSEVQAKAIMDMKLQRLTGLEREKIEQEFQELEIKIRELKEILADENKIYEIMKKELTELKEKYNDPRRTSIEDERLVIDREDLIKQEKVIITITNKGYVKRIGLDQYKSQKRGGKGVSTQHTVEDDFVQDMEIMSTHDSMMIFTNKGRVFNIKAYEIPESSKQARGKLIENIIKLQENEKVRFTIKIKEFTNKKEIIFLTQDGTVKKTNLMEFQNIHSGGLKAVKLREDDDLIFVGVVTSEDDELFMATKFGYSVRTKVSEFRSMSRNATGVKGITLRHGDSVVSGLLIKNEDEEMILTITENGFGKRTRVNEYPLQGRGGKGVINFKCSDKTGNIVEVKPVREDEELMAITSSGVVIRTPIESVSIYGRATQGVKIMRTSDEEKVVAIAKVKSEKDEEKIISDDQREELESKKEVGNQSSEKEDFENKYMSTFENEDEEQMQYESEQGSDDSEEN | K02469 | 1030.33 | 1268.6 | 0 | DNA gyrase subunit A [EC:5.6.2.2] | macrophage infectivity potentiator Mip | Post-translational modification |
| GC_00000348 | MIKGLSKSFDGVQVIKNIDLEIKAGEFFSILGPSGCGKTTLLRILAGFIEPDSGVVYLGDKDITNLPPNLRSTNTIFQKYALFPHLTVYENIAFPLRMKKMDSDYIDTEVKKMLKMIELEEHASKKPNQLSGGQQQRVSIARALVNKPEVLLLDEPLSALDAKLRQNLLIELDNIHEEVGITFIFITHDQQEALSISDRIAIMNKGKVLQVGTPAEVYESPADMFVADFIGENNFIEGEVISIDSETCATLKHERLGEIRFEMDRKVKIGDHVKVSVRPEKIRLTRNLPKNLGKIHNVIKVYVDELIYTGFQSKYFVWLNGEKDLLFKVFKQHAIFFEDDESSVRWDEEAYISWNSEDSYLVEVDENEKR | K11072 | 488.1 | 490.7 | 5.00E-148 | spermidine/putrescine transport system ATP-binding protein [EC:7.6.2.11] | iron(III) ABC transporter, ATP-binding protein | Nutritional/Metabolic Factor |
| GC_00000349 | MENKENKNENFEKNIENKNGILLKIDNFEGPLELLLYLIEKKKLKISEVKISQLIDEYLAVIERSKKDNLEIKVEFLLTATELLEIKAMSVLHIDDEIETEKELKQKLEDYKLFKEVTEKISTMGSEFNISYSRGEGRKIRKVESKEYDLNSLKKEDLFNVYKKHLDVVDEEFIQIKYEKNYSVEEEIEKIKVILFEKPLTLDEIFSRAENKIHLVYIFLAVLDIYKEGKIDIINKDKDIFIKRTDAY | K05896 | 39.6 | 143.2 | 1.40E-42 | segregation and condensation protein A |  |  |
| GC_00000350 | MGGYIKKPEDIEKRSFEIITEELGEKVKKFTEEELPIVKRVIHTTADFEYADLIEFLGNPIRSAKKVLSKGCKIYCDTNMIVNGLSKNILKKFNCVPYTLVSDAEVSKEAKERGVTRSIVGMEHAGKDKETKIFLIGNAPTALYKLKEMIEKNEIEKPALVVGVPVGFVGAAESKEIFKNIKVPYITINGRKGGSTAAVSILHGILYQIYKREGF | K06042 | 92.87 | 263.8 | 3.20E-79 | precorrin-8X/cobalt-precorrin-8 methylmutase [EC:5.4.99.61 5.4.99.60] |  |  |
| GC_00000351 | MAVLNIYNLAGAQTGTVEVNDAIFGIEPNKVVLHEVLVAELAAARQGSASTKTRSMVRGGGRKPFKQKGTGRARQGTIRAPHMVGGGVVFGPSPRSYEKKVNKKTRVLALKSALSAKVAAGEIVVLDGVMETPKTKTIVALTKALEANTKQMFVVNDLTAQGDYNLFLSARNLENAVVFQPNELGIYWLLKQNKVIITKEALATIEEVLG | K02926 | 89.3 | 265.1 | 1.50E-79 | large subunit ribosomal protein L4 |  |  |
| GC_00000352 | MFKKYISLDIGDVRIGVARSDIMGLVATPLEVIDRKKVKSVKRIAEILNENNTKSLVVGIPKSLDGTEKRQAEKVREYLEKLKKNIEGLEIFEVDERLTTVSADRMLTEGGKKGALEKRKVVDKIAAAIILQTFLDSKK | K07447 | 47.1 | 157.3 | 4.30E-47 | putative pre-16S rRNA nuclease [EC:3.1.-.-] |  |  |
| GC_00000353 | MKETAEESIVKIEKIVLPVLKEMELELVDIEYLQEGGYWYVRIYIEKLDGDVSLDDCAKVSMAVEDDIDKLIDKKFFLEISSPGVERPLKREKDYIRFTGSKIKVSLKHKINERKILKEYYPNLKMTQYF | K09748 | 28.73 | 124.9 | 5.80E-37 | ribosome maturation factor RimP | Coxiella Dot/Icm type IVB secretion system translocated effector | T4SS Effector Delivery System |
| GC_00000354 | MKQEITYDTLLEKIPNKYILTIAAGKRVREIVNGSPVLVKTSKKDTLVRKVFKEIVEGKLTYSMEETPVSK | K03060 | 24 | 50.3 | 4.00E-14 | DNA-directed RNA polymerase subunit omega [EC:2.7.7.6] |  |  |
| GC_00000355 | MTTTAMYLAEFVGTALLLLLGNGINMTLSLRKSFGKGGGWSVTCIGWGMAVTMSAYLTGWVNGAHLNPALSIAMVLSGRMDAALLPGYIIAQILGGIFGATLAYLAYKDLMDDEPEAGTKLGVFSTGPAIDHKPWNVVTEAIGTAVLVIGILAIGYGANGVSGGMGPFLVGMLICVIGMALGGATGFAINPARDLGPRIAHAILPIKGKGSSNWQYAWVPIVGPIIGAVLGVVIFDLFAAACVGI | K02440 | 270.8 | 322 | 5.00E-97 | glycerol uptake facilitator protein |  |  |
| GC_00000356 | MSYTYQVKNEIIKKGGYTTKEKIAELRGILDAKNAVFDDHIELKLESIELANRVYRILKEITELKLNVKYSTSKNFGEHNIYIIGIVNQKGFKDFLKLLEEFRGKHIREDEEKVYGFIRGLFLSTGYIKSPEKEYALDFFIDNEEIAEELYEILGDLGKKVSKTKKRNKNLVYLRNSEDIMDILVINGSIKEFYKYEETTMMKDLKNKTIREMNWEVANETKTLNTGIKQIKQINYIGAKIGINNLTPVLEEVAFLRLQNPESSLQELAEMIGISKSGIRNRFRRIEEIYNQLLEENGEQPKE | K09762 | 107.1 | 233.2 | 5.60E-70 | cell division protein WhiA | ADP-ribosyltransferase exoenzyme family protein | Exotoxin |
| GC_00000357 | MLDTLEIMKRIPHRYPFLLVDRILELDMENQSVKGIKNVTVNEEYFNGHFPGHPIVPGVIIVEGIAQCLGVLVFEQQGDPEHPKVPYFAAIESVKFKAPVRPGDQLVYEAQIEKQRRNIVKANGVAKVDGKVVAEVKFTFSIMDK | K02372 | 72.53 | 198.3 | 2.60E-59 | 3-hydroxyacyl-[acyl-carrier-protein] dehydratase [EC:4.2.1.59] | (3R)-hydroxymyristoyl ACP dehydratase | Immune Modulation |
| GC_00000358 | MLIISDKVRINEKIKGKEFRIISSTGEQLGIMSISEALEAARKEELDLVEISPAAVPPVCKIMDYGKYKYEQTRKAKDAKKKQKVVVVKEVKLRTRIDDHDLETKVNAIKKFLEKDNKVKVTLVQFGREKSYQDMGIELLDQVAGQLEDVAEVEKRYKESQKYLMLSAKK | K02520 | 38.33 | 203.8 | 5.50E-61 | translation initiation factor IF-3 |  |  |
| GC_00000359 | MEKIRINKFLAELGVGSRRAIDKMIEEKRIKVNGVLAESGIKVDKSDKISVNGKLLEFEKKQKVYFMLNKPKRVLSTAKDERGRKTVVDLIDTKERIFPIGRLDYDTEGLLLLTNDGEIFNKVIHPRTEVYKTYLVEARGNINMTTLNKLKRGIMLDDKMTLPAKAKILLADERHTVLHFAIKEGRNRQVRRMFELVGHPVINLKRIMLGELNLEGLEVGEYRPLTKKEINYLYSL | K06178 | 263.33 | 320.8 | 2.80E-96 | 23S rRNA pseudouridine2605 synthase [EC:5.4.99.22] | negative regulator EscL, stator protein | Effector Delivery System |
| GC_00000360 | MKLADSHCHVNDSKFDEDREEVFKRAETELEFIVNIGNDLKTSEESIKYSEKYPFVYAVVGVHPTDISTYNDEVEKRIEDLSKHEKAVAIGEIGLDYYWMEDEKEVQKEGFRRQMKLAEKVGLPVVIHSRDAMEDTINILNEFPDVKGIFHCYPGSFESAMLVPKGYVFGIGGVLTFKNARKTVEFIEKIDLSRIVIETDSPYLTPSPFRGKRNEPVYVKYVAEKIAEIKNISVEEVIRITTENAKKIYNIR | K03424 | 89.57 | 296 | 8.30E-89 | TatD DNase family protein [EC:3.1.21.-] |  |  |
| GC_00000361 | MIVYIVVLVLMGIFFLLLRNQEKENVHVMEKVLRSLKEKKLYDKIPPELKEQYIETLHKIIKQDLELDNSIEELREYRKELEVTYNALVDKSRKLEYSNQVLETRVTNLSNINSLSRTVLSIVELDHIINIILDAYFVLTGAKKVSLYLWEEGKLINKQTRGNVNFRGEVSYPIEILQKFTYQDYKRVYEELKKGFQISPDEVIVVSPLLVKGKELGVIFIIEDKNKFIGNDEETISALTIQVAIAINNAKIYADLRVKERISQELEVASRIQKTILPKNITNIYGLQIANYFEPAKEVGGDYYDYTVKDDENVSITIADVSGKGVPAAFLMALGRSILKTLERQGQSPADNLRNLNQIIYPDITEDMFITMLHSNFNNKTKILTYSNAGHNPLIVYRAKEDKVELHSVKGVAIGFMQGYNYKQGELKLEKGDIVLYYTDGITEAENPNKELFGFERLEKVIYDNRYNDVDTIKENILNEINIFRRDYPQVDDLTFVVIKNVE | K07315 | 145.23 | 271.8 | 1.10E-81 | phosphoserine phosphatase RsbU/P [EC:3.1.3.3] | SpoIIE family protein phosphatase | Effector Delivery System |
| GC_00000362 | MGLFNFNFGSGKGIGIDLGTANTLVYSKKQKKIVLNEPSVVAVEKETRKVLAVGNEAKEMIGKTPDSIVAIKPLSEGVIADYDVTEAMIKYFIKKVFGNTIFMPDVMICVPIDVTGVEKRAVLEAALSAGAKRAYLIEEARAAALGAGIDISAPTGNMIVDIGGGSTDVAVISFGGTVVSKTIRTASNNFDMDIIKYIKKTHNLLIGDKTAEEIKIKIGTAIPLEEEESLVVKGRDLIMGLPKSITITSEEVREAISDSLMEIVTCVKEVLEKTPPELAADIVDRGIVMAGGGSLIKNFPVLISQHTHLSVRLAENPLLSVVVGAGMAMDQLKVLKKIEKAER | K03569 | 115.27 | 501 | 3.30E-151 | rod shape-determining protein MreB and related proteins |  |  |
| GC_00000363 | MNEIMAKYGTFIMIAVWIAIIYFFMILPNKKKQKKQKEMMDSLKEGNEIVTIGGIKGTVSSVEDDYLQIRVDKGVNLTIRKSAVAVVLK | K03210 | 32.87 | 105.5 | 4.90E-31 | preprotein translocase subunit YajC |  |  |
| GC_00000364 | MEKIKIQTEYIKLDQFLKWTGVAESGVHAKDMILAGEVKVNGEVEERRGKKLYKGDKVEIAGKTFEVE | K14761 | 49.3 | 100.8 | 9.30E-30 | ribosome-associated protein |  |  |
| GC_00000365 | MDLALELAEKGRGYVNPNPMVGAVVVKDGEIVGKGWHKFYGGPHAEVYALDEAGAKAEGATIYVTLEPCSHFGKTPPCAEKIKKMKIKKCVIACLDPNPIVAGRGKKILEEAGIEVVVGVREKEAKELNKVFMKYITEKNPYLFLKCAITLDGKIATNERDSKWITNEKSREKVQFLRHEYMSIMVGINTLINDNPRLTARIENGVNPFRIVVDPHLRTPLESNFVNMADDNKSIVITSKENEKNNKITELENKNIKIIYMEGYDFSVHEILKKIGELKIDSVLLEGGSYLISKAFKENRIDGGEIFIAPKILGGGLPFIDGFDFKEIKDCFHLENVKFNVYDDNISVEFHK | K11752 | 196.53 | 427.6 | 1.30E-128 | diaminohydroxyphosphoribosylaminopyrimidine deaminase / 5-amino-6-(5-phosphoribosylamino)uracil reductase [EC:3.5.4.26 1.1.1.193] |  |  |
| GC_00000366 | MKRELALEFARVTEAAALAAQKWVGRGDKNLADGAAVEAMRNVLNRIKIDGEIVIGEGEIDEAPMLYIGEKVGLKYNPEKIEEFREDELSAVDIAVDPIEGTRMTAQGQPNAIAVLAAAQRGTFLKAPDMYMEKLVVGPEAKGAIDLDKSLEDNIRSVAKALKKDVKDMMIVVLDKPRHKKAMEQIRDLGAKLYALPDGDVAGSILTSIVDSDVDMLYGIGGAPEGVISAAIIKVLGGDMQGRLLLRSDVKGSDDKNDKISADEKRRCDEVGVEVGKKLLLDDLVKTDEIIFSATGITSGDLLEGVKRKGNIARTQTLVVRGSSKTVRYINSVHNLEYKDIYLEKLLKD | K02446 | 473.1 | 486.6 | 6.50E-147 | fructose-1,6-bisphosphatase II [EC:3.1.3.11] |  |  |
[truncated: 791,490 more chars]
